# Scoring reading parameters: An inter-rater reliability study using the MNREAD chart

**DOI:** 10.1371/journal.pone.0216775

**Published:** 2019-06-07

**Authors:** Karthikeyan Baskaran, Antonio Filipe Macedo, Yingchen He, Laura Hernandez-Moreno, Tatiana Queirós, J. Stephen Mansfield, Aurélie Calabrèse

**Affiliations:** 1 Department of Medicine and Optometry, Linnaeus University, Kalmar, Sweden; 2 Low Vision and Visual Rehabilitation Lab, Department and Center of Physics—Optometry and Vision Science, University of Minho Braga, Braga, Portugal; 3 Department of Ophthalmology & Visual Neurosciences, University of Minnesota, Minneapolis, United States of America; 4 Serviço de Oftalmologia, Hospital de Braga, Braga, Portugal; 5 Department of Psychology, SUNY College at Plattsburgh, Plattsburgh, New York, United States of America; 6 Aix-Marseille University, Marseille, France; 7 Laboratoire de Psychologie Cognitive, CNRS, Marseille, France; Marshall B. Ketchum University, UNITED STATES

## Abstract

**Purpose:**

First, to evaluate inter-rater reliability when human raters estimate the reading performance of visually impaired individuals using the MNREAD acuity chart. Second, to evaluate the agreement between computer-based scoring algorithms and compare them with human rating.

**Methods:**

Reading performance was measured for 101 individuals with low vision, using the Portuguese version of the MNREAD test. Seven raters estimated the maximum reading speed (MRS) and critical print size (CPS) of each individual MNREAD curve. MRS and CPS were also calculated automatically for each curve using two different algorithms: the original standard deviation method (SDev) and a non-linear mixed effects (NLME) modeling. Intra-class correlation coefficients (ICC) were used to estimate absolute agreement between raters and/or algorithms.

**Results:**

Absolute agreement between raters was ‘excellent’ for MRS (ICC = 0.97; 95%CI [0.96, 0.98]) and ‘moderate’ to ‘good’ for CPS (ICC = 0.77; 95%CI [0.69, 0.83]). For CPS, inter-rater reliability was poorer among less experienced raters (ICC = 0.70; 95%CI [0.57, 0.80]) when compared to experienced ones (ICC = 0.82; 95%CI [0.76, 0.88]). Absolute agreement between the two algorithms was ‘excellent’ for MRS (ICC = 0.96; 95%CI [0.91, 0.98]). For CPS, the best possible agreement was found for CPS defined as the print size sustaining 80% of MRS (ICC = 0.77; 95%CI [0.68, 0.84]). Absolute agreement between raters and automated methods was ‘excellent’ for MRS (ICC = 0.96; 95% CI [0.88, 0.98] for SDev; ICC = 0.97; 95% CI [0.95, 0.98] for NLME). For CPS, absolute agreement between raters and SDev ranged from ‘poor’ to ‘good’ (ICC = 0.66; 95% CI [0.3, 0.80]), while agreement between raters and NLME was ‘good’ (ICC = 0.83; 95% CI [0.76, 0.88]).

**Conclusion:**

For MRS, inter-rater reliability is excellent, even considering the possibility of noisy and/or incomplete data collected in low-vision individuals. For CPS, inter-rater reliability is lower. This may be problematic, for instance in the context of multisite investigations or follow-up examinations. The NLME method showed better agreement with the raters than the SDev method for both reading parameters. Setting up consensual guidelines to deal with ambiguous curves may help improve reliability. While the exact definition of CPS should be chosen on a case-by-case basis depending on the clinician or researcher’s motivations, evidence suggests that estimating CPS as the smallest print size sustaining about 80% of MRS would increase inter-rater reliability.

## Introduction

Reading difficulty is a major concern for patients referred to low-vision centers [1]. Therefore, most Quality-of-Life questionnaires assessing the severity of disability caused by vision loss contain one or more items on subjective reading difficulty [2–5]. However, substantial discrepancy has been observed between self-reported reading difficulty and measured reading speed [6]. For this reason, reading performance should be evaluated objectively to serve as a reliable outcome measure in clinical trials, multisite investigations or longitudinal studies. To assess, for instance, the success of vision rehabilitation, surgical procedures or ophthalmic treatments, measures of reading ability should be obtained using standardized tests with demonstrated high repeatability.

**Fig 1:**
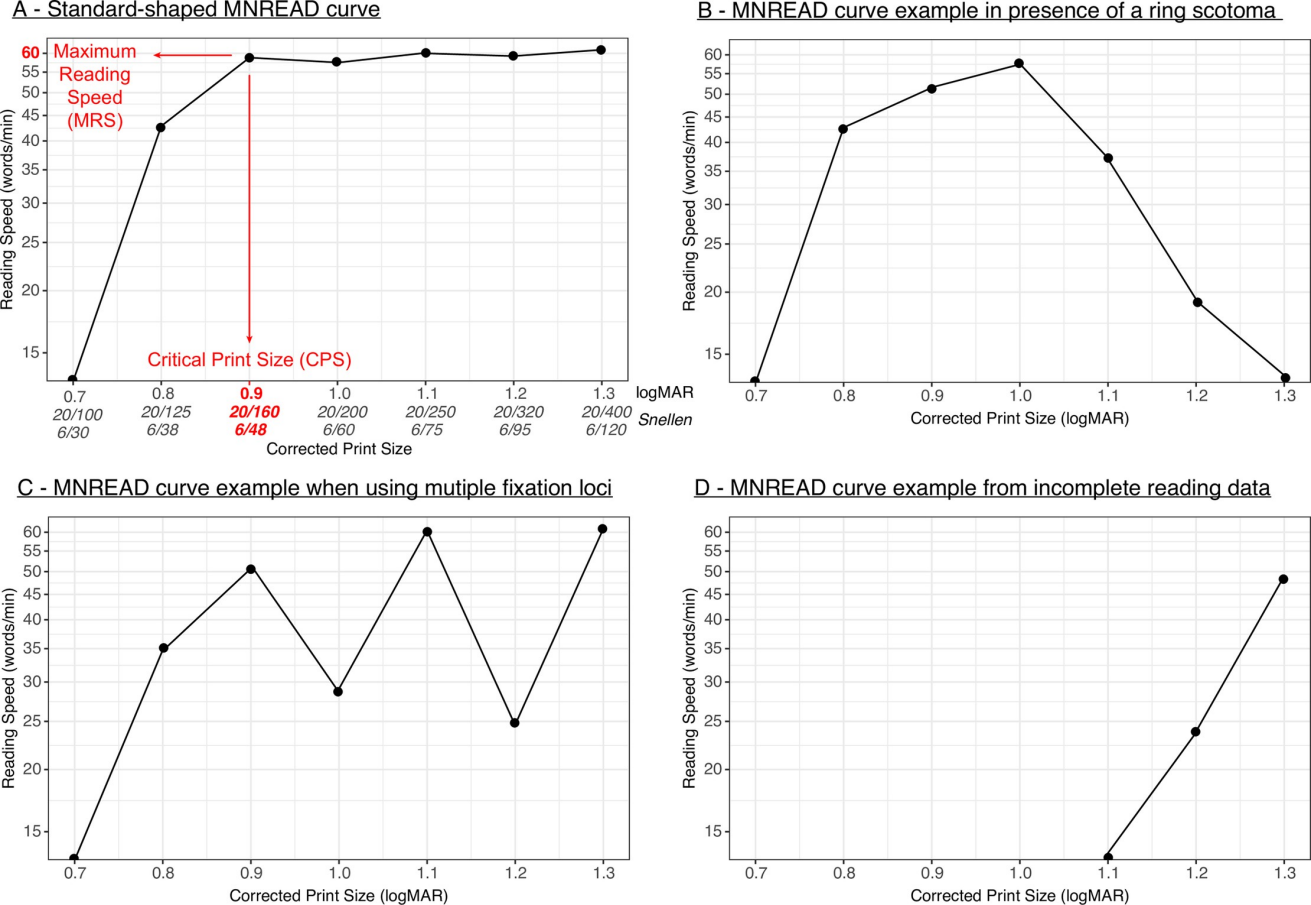
MNREAD curve examples.

Among the standardized tests available, the MNREAD acuity chart can be used to evaluate reading performance for people with normal or low vision in clinical and research environments [7]. The MNREAD test measures four parameters that characterize how reading performance changes when print size decreases: the maximum reading speed (MRS), the critical print size (CPS), the reading acuity (RA) and the reading accessibility index (ACC) [8]. The reading acuity and reading accessibility index are clearly defined by the number of reading errors made at small print sizes and the reading speeds for a range of larger sizes. In the original MNREAD manual provided with the chart, MRS and CPS are defined as follows: “The critical print size is the smallest print size at which patients can read with their maximum reading speed. […] Typically, reading time remains fairly constant for large print sizes. But as the acuity limit is approached there comes a print size where reading starts to slow down. This is the critical print size. The maximum reading speed with print larger than the critical print size is the maximum reading speed (MRS).” In short, values for MRS and CPS depend on the location of the flexion point in the curve of reading speed versus print size (Fig 1).

In normally sighted individuals, for whom the MNREAD curve usually exhibits a standard shape (Fig 1A), the above definitions may be sufficient to extract MRS and CPS confidently by inspecting the curve. However, they can be difficult to determine for readers with visual impairment who experience visual field defects (e.g. ring scotoma; Fig 1B) or use multiple fixation loci (i.e. Preferred Retinal Locations; Fig 1C) [9]. In such cases, variable visual function may lead to noisy and/or incomplete reading data (Fig 1D) and be inconsistent with the assumption that people will read at a fairly constant speed until font size compromises their ability to identify words. Because of atypical curves (Fig 1B–1D), subjective decisions (e.g. ignoring outliers) must be made by the individual analysing the data (referred to as the “rater” in the present work, as opposed to the “experimenter” who recorded the data). For this reason, MRS and CPS estimates may be considered highly sensitive to inter-rater variability.

In an attempt to reduce variability and unify the process of curve information extraction, alternative scoring methods have been proposed. According to these “simpler” scoring rules, MRS equals either the single largest reading speed [10] or the mean of the three largest reading speeds [11]. Nonetheless, a criterion must be chosen for the CPS (smallest print size supporting reading speed at either: 90% of MRS, 85%, 80%, etc.) but there is no general agreement on the appropriate criterion to use. Overall, open discussions on how to score MNREAD parameters optimally still persist in the literature [12] and the choice of scoring method constitutes an additional factor contributing to inter-rater variability. Another approach to reduce variability is to fit the MNREAD curve and estimate its parameters using automated algorithms [13]. In the present work, we will focus on two of these methods. The first one has been described by the MNREAD inventors [14,15] and is used in the MNREAD iPad app [16]; we will refer to it as the standard deviation method (SDev). The second method relies on smooth curve-fit using non-linear mixed effects (NLME) modeling [17]; we will refer to it as the NLME method.

In this study we have investigated the reliability of CPS and MRS estimates for MNREAD data curves obtained from participants with visual impairment. First, we evaluated the inter-rater reliability among raters (Analysis 1). Second, we evaluate agreement between the NLME and SDev algorithms (Analysis 2). Third, we evaluated agreement between raters and the two algorithms (Analysis 3).

## Methods

### Data source

Data from participants with visual impairment were obtained from a larger dataset, that included more than 500 participants recruited from 4 hospitals, originally collected to study the prevalence and costs of visual impairment in Portugal (PCVIP-study) [18–20]. In the original study, participants were recruited if their medical records indicated at least one of the inclusion criteria: 1) visual acuity in the better seeing eye less than 0.5 decimal (i.e. 6/12; 20/40; 0.3 logMAR); 2) visual field less than 20 degrees. In the present work, we included data collected in only two sites because reading was always tested in the same room. Luminance in each room was respectively 682±7 lux and 455±10 lux, measured at 1m above the floor and away from a direct effect of a ceiling light. Reading curves were obtained binocularly with participants’ “presenting reading glasses”. Reading data was excluded from analysis when the number of data-points corresponded to less than five sentences on the MNREAD test. We ended with a dataset of 101 reading curves. The study protocol was reviewed by the ethics committee for Life Sciences and Health of the University of Minho (REF: SECVS-084/2013) and was conducted in accordance with the principles of the Declaration of Helsinki. Written informed consent was obtained from all participants. The study was registered with the Portuguese data protection authority with the reference 9936/2013 and received approval number 5982/2014.

### MNREAD data

Reading performance was measured for each participant using the Portuguese version of the MNREAD acuity chart [21]. Reading distance was adjusted for each participant, to either 20 or 40 cm, according to his/her near visual acuity. Participants were asked to read the chart aloud as fast and accurately as possible, one sentence at a time, starting from the largest print size. For each sentence, reading time and number of misread words were recorded and reported on a score sheet by the experimenter. Data were then transferred into a digital file and further processed in R [22]. For each individual test, a corresponding MNREAD curve was plotted using the mnreadR package [23] to display log reading speed as a function of print size (see S1 Appendix for all 101 curves). Because the shape of the curve can influence visual estimation of the reading parameters, reading speed was plotted using a logarithmic scale so that reading speed variability (which is proportional to the overall measure of reading speed) was constant at all speeds [14].

### Raters’ visual scoring

Seven raters were recruited to estimate the MRS and CPS of each individual MNREAD curve. Since inter-rater reliability may be influenced by raters’ prior experience with the MNREAD chart, we included raters with different levels of expertise in MNREAD parameters estimation. Each rater gave a self-rated score of expertise (on a 5-point scale, from 0 = ‘no previous experience’ to 4 = ‘top expertise’), both before and after rating all the MNREAD curves, to account for the amount of practice gained during the study. Each rater was provided with S1 Appendix, containing the 101 MNREAD curves to score. Raters were instructed to follow the standard guidelines provided with the MNREAD chart instructions (see Introduction). However, coming from patients with impaired vision, many of the curves had noisy or incomplete data, which potentially made it difficult to estimate the MRS and CPS. In such cases, we provided more detailed instructions to the raters. These detailed instructions are available in S2 Appendix.

### Algorithms’ automated scoring

**Fig 2:**
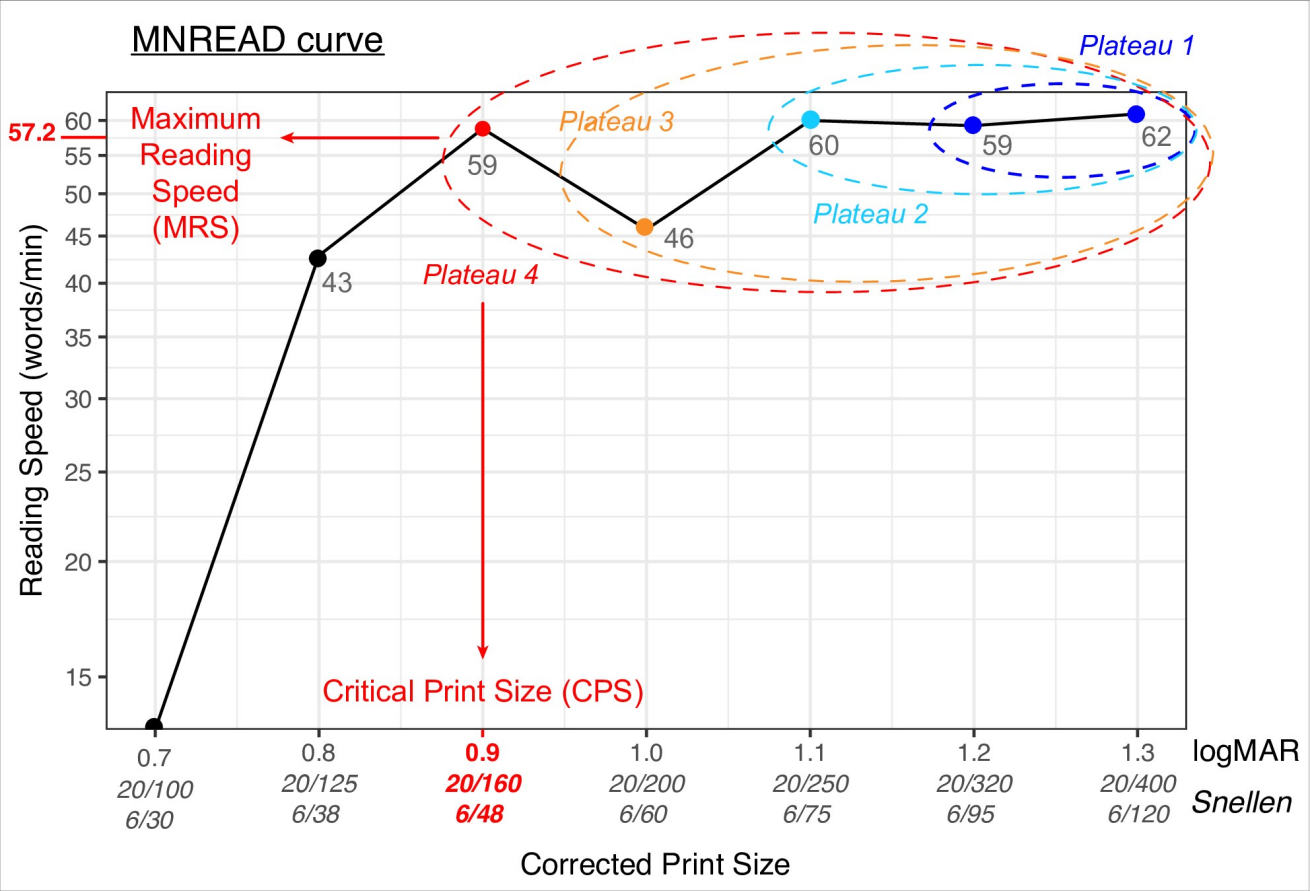
Example of the standard deviation algorithm calculation on a typical MNREAD curve. On *iteration 1* (dark blue), the algorithm selects the first two sentences as *plateau 1* (x-axis: 1.3 and 1.2 logMAR) and calculates a selection criterion for this plateau. Criterion _plateau 1_ = mean (reading speed _plateau 1_)– 1.96 x standard deviation (reading speed _plateau 1_) = 60.5–1.96 × 2.1 = 56.3 wpm. The point adjacent to *plateau 1* (x-axis: 1.1 logMAR) was read at 60 wpm, which is faster than criterion _plateau 1,_ indicating that this point belongs to the optimal plateau. A second iteration is then launched (light blue) with *plateau 2* now encompassing the first three sentences and a new criterion calculation. Criterion _plateau 2_ = 60.3–1.96 × 1.5 = 57.3 wpm. Among the points adjacent to *plateau 2*, there is still a value higher than this criterion (y-axis: 59 wpm at *x-axis*: 0.9 logMAR), so the algorithm continues to iterate one sentence at a time, including reading speeds at 1.0 logMAR in *plateau 3* and at 0.9 logMAR in *plateau 4*. The calculations stop with *plateau 4*, for which selection criterion is higher than any remaining points (criterion _plateau 4_ = 44.7 wpm). MRS is estimated as 57.2 wpm and a corresponding CPS of 0.9 logMAR.

MRS and CPS were also calculated automatically for each 101 datasets using two algorithm-based estimations: the ‘standard deviation’ method and NLME modeling. The standard deviation method (SDev) uses the original algorithm described in the literature to estimate the MNREAD parameters [14,15]. This algorithm iterates over the data searching for an optimal reading speed plateau, from which MRS and CPS will be derived. To be considered optimal, a plateau must encompass a range of print sizes that supports reading speed at a significantly faster rate (1.96 × standard deviation) than the print sizes smaller or larger than the plateau range (Fig 2). MRS is estimated as the mean reading speed for print sizes included in the plateau and CPS is defined as the smallest print size on the plateau. In most cases, several print-size ranges can qualify as an optimal plateau and the algorithm chooses the one with the fastest average reading speed. In the present work, the standard deviation method estimation was performed using the curveParam_RT () function from the mnreadR R package.

The NLME modeling method is particularly suited for incomplete datasets from individuals with reading or visual impairment as it uses parameter estimates from a larger group (101 datasets here) to allow suitable curve fits for individual datasets that contain few data points [17]. In the present work, we used an NLME model with a negative exponential decay function, as described in detail by Cheung et al. (2008) [17]. In this approach, a single estimate of MRS can yield several measures of CPS depending on the definition chosen (e.g. print size required to achieve 90% of MRS, 80% of MRS, etc.). Therefore, five values of CPS were estimated, i.e. 95%, 90%, 85%, 80% and 75% of MRS. NLME modeling and parameters estimation were performed using the nlmeModel () and nlmeParam () functions from mnreadR. The resulting curve fits are available in S3 Appendix.

### Statistical analysis

Intra-class correlation coefficient (ICC) was used in all the 3 analysis to assess absolute agreement between raters and/or algorithms [24]. The ICC reliability index (ranging from 0 to 1; 1 meaning perfect agreement) is widely used in the literature in test-retest, intra-rater, and inter-rater reliability analyses [25]. In this study ICC values estimate the variation between two or more methods (whether raters or algorithms) in scoring the same data by computing the absolute agreement between them. For each analysis, the appropriate ICC form (dependent on research design and assumptions) was chosen by selecting the correct combination of “model”, “type” and “definition”, as detailed in Table 1 [26]. ICC values were calculated using SPSS statistical package (IBM-SPSS, v24, Chicago, Illinois) and limits of agreement were visualized with Bland–Altman plots. Following guidelines from Koo & Li, 2016 [25], ICC values and their 95% confidence intervals (95% CI) were interpreted as showing: *“poor agreement”* if less than 0.5; *“moderate agreement”* if comprised between 0.5 and 0.75; *“good agreement”* if comprised between 0.75 and 0.9 and *“excellent agreement”* if greater than 0.9.

**Table 1:** Details of the ICC form chosen for analyses 1, 2 and 3.

	Intra-class correlation coefficient (ICC) form		
	Model	Type	Definition
Analysis 1—Agreement among the 7 raters	2-way random effects—*Both raters & curves are considered as selected randomly from a larger population*	Single rater—*Each rater is compared against all others*	Absolute agreement
Analysis 2—Agreement between the 2 automated algorithms	2-way mixed-effects—*Raters are fixed & curves are considered as selected randomly from a larger population*	Single measurement	Absolute agreement
Analysis 3—Agreement between raters and automated algorithms	2-way mixed effects	Mean of *7* raters	Absolute agreement

## Results

### Analysis 1: Agreement between raters (221 words)

**Fig 3:**
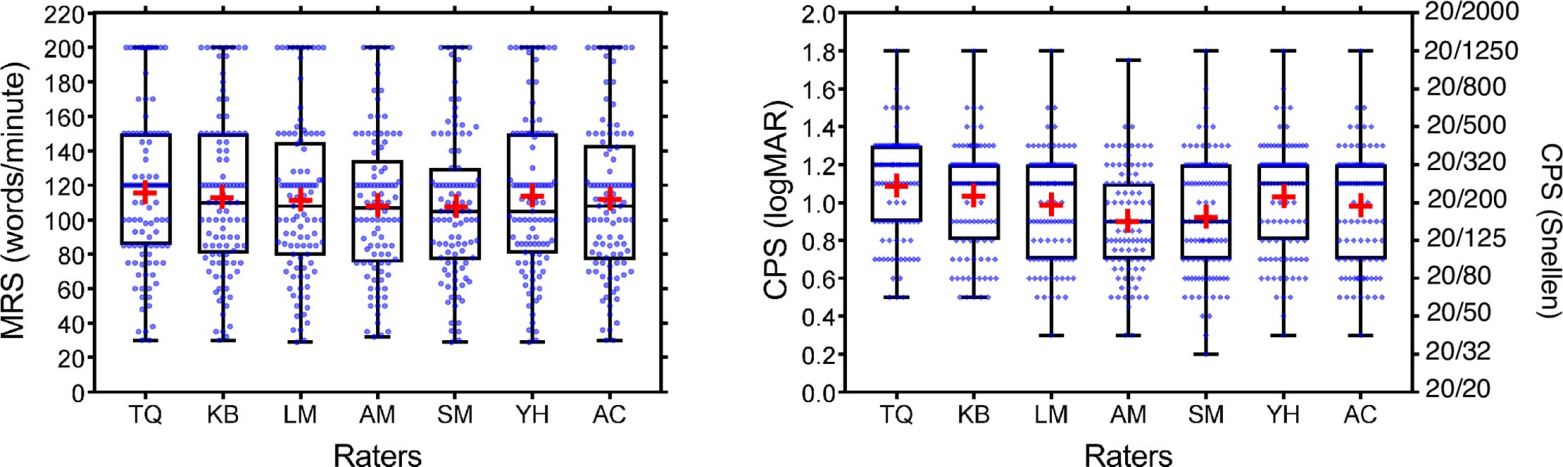
Box and whisker plots of estimated MRS (left) and CPS (right), grouped by raters and sorted in ascending order of expertise level (from 0 to 4). Boxes represent the 25th to 75th percentiles and whiskers range from min to max values. Medians (lines) and means (red cross) are also represented.

For MRS ICC value was 0.97 (95% CI [0.96, 0.98]), indicating excellent agreement between raters (Fig 3). The ICC value for CPS was 0.77 (95% CI [0.69, 0.83]), suggesting ‘moderate’ to ‘good’ agreement between raters. We hypothesized that the weaker agreement for CPS could be attributed to the difference in raters’ expertise level. These scores, both before and after evaluating the 101 MNREAD curves, are reported in Table 2. Prior to rating, one rater had no previous experience in rating MNREAD curves (TQ), three raters considered themselves intermediate raters (LM, AM and KB), two raters scored themselves as advanced raters (SM and YH) and one rater reported to be an expert rater (AC). Among the less experienced raters (score 0–2), CPS estimation reliability was only ‘moderate’ to ‘good’ (ICC = 0.70; 95% CI [0.57, 0.80]). Among the most experienced raters (score 3–4), it was good **(**ICC = 0.82; 95% CI [0.76, 0.88]).

**Table 2:** Self-reported score of expertise for our 7 raters.

Raters		TQ	LM	AM	KB	SM	YH	AC
**Self-reported score of expertise**	Prior rating	0	2	2	2	3	3	4
	After rating	1	3	3	2	3	3	4

Score of expertise in extracting MNREAD parameters before and after rating 101 curves (0 –no prior experience, 1 –novice, 2 –intermediate, 3 –Advance, 4 –Expert).

### Analysis 2: Agreement between automated algorithms (245 words)

**Fig 4:**
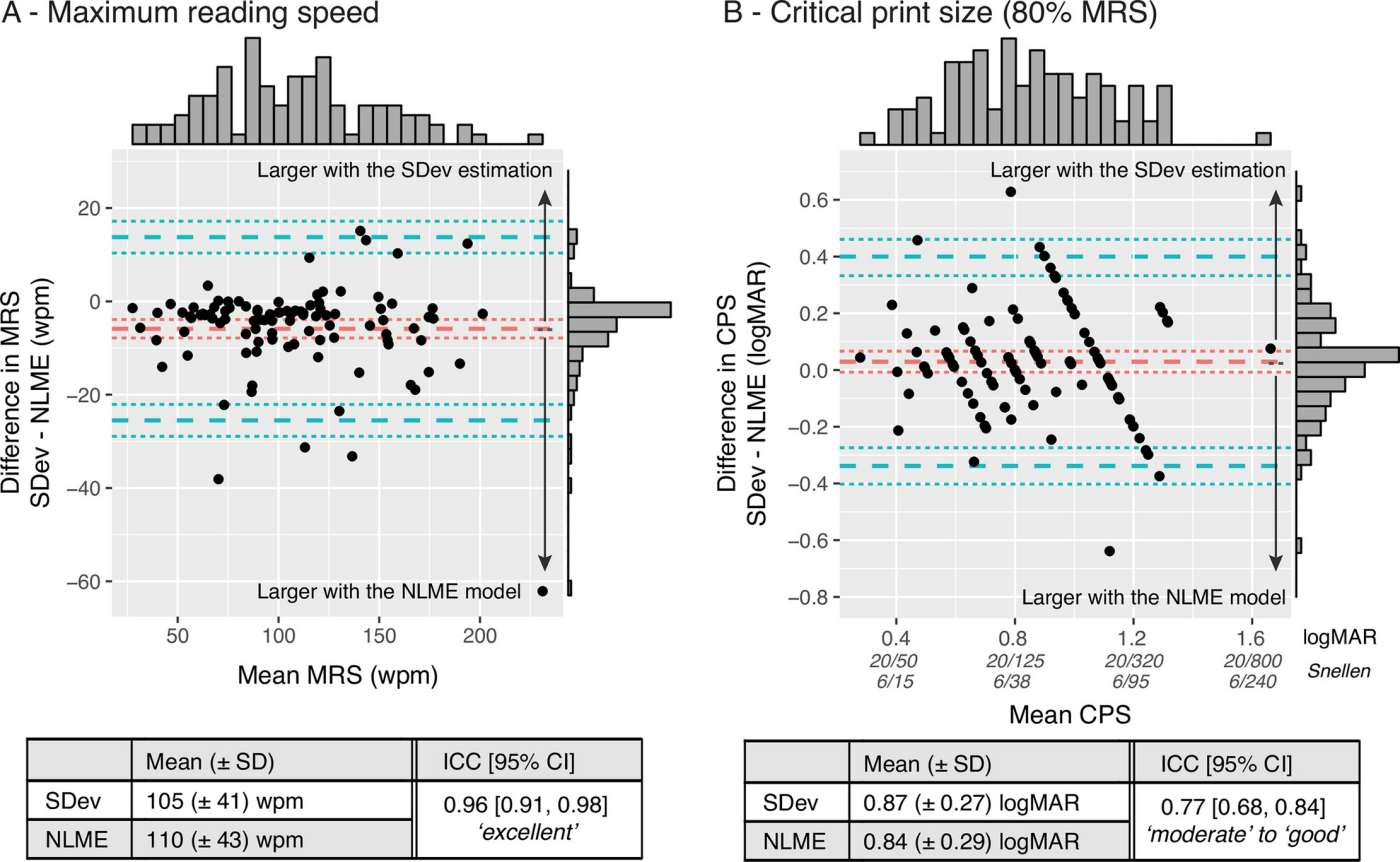
Bland–Altman plots showing agreement between SDev and NLME methods for both MRS (left) and CPS (right). x-axes represent the mean estimate for both methods; y-axes represent the estimate difference between the two methods (SDev—NLME). In both subplots, the red dashed lines represent the mean difference (i.e. bias) and the blue dashed lines represent the agreement limits (±1.96 SD). The dotted lines show the 95%CI of the limits; top and right histograms show the data distribution along the x- and y-axes respectively. Tables summarize the SDev and NLME average values as well as their ICC values of absolute agreement.

For MRS, the ICC value of absolute agreement between SDev and NLME methods was 0.96 (95% CI [0.91, 0.98]), showing excellent agreement. Contrary to the SDdev method, for which a single MNREAD test yields only one estimate for MRS and one estimate for CPS, the NLME method can generate several measures of CPS depending on the reading-speed criterion chosen to define the CPS (e.g. print size required to achieve 90% of MRS, 80% of MRS, etc.). Therefore, for each of the 101 MNREAD curves, we estimated five values of CPS with NLME (corresponding to: 95%, 90%, 85%, 80% and 75% of MRS) and measured agreement between SDev and NLME for each of them. The results are reported in Table 3. The strongest agreement between the two automated methods was obtained for the 80% criterion, and was ‘moderate’ to ‘good’, with an ICC value of 0.77 (95% CI [0.68, 0.84]). In addition, limits of agreement between the two algorithms were estimated using Bland–Altman plots for both MRS and CPS (Fig 4). For MRS, the average difference (i.e. bias) between the SDev method and the NLME model was −5.8 wpm (i.e. 4.5%), with 95% limits of agreement of ranging from -26 to 14 wpm. A post-hoc paired sample *t*-test revealed that this difference was significant (t(7) = -2.5, p = 0.04, 95% CI [-44.4, -1.1]). For CPS (defined as 80% of MRS, which showed the best agreement between methods), bias was 0.031 logMAR with 95% limits of agreement ranging from -0.34 to 0.40 logMAR (1 step unit being 0.1 logMAR). A post-hoc paired sample *t*-test revealed that this difference was not significant (t(7) = -0.9, p = 0.4, 95% CI [-0.5, 0.2]).

**Table 3:** Absolute agreement (ICC values and their 95% confidence intervals) between CPS values estimated with the SDev and the NLME methods for five different definitions of CPS.

	ICC value	95% CI	Absolute agreement
95% CPS	0.56	[0.10, 0.77]	Poor to good
90% CPS	0.70	[0.53, 0.81]	Moderate to good
85% CPS	0.76	[0.66, 0.83]	
**80% CPS**	**0.77**	**[0.68, 0.84]**	
75% CPS	0.76	[0.62, 0.84]	

Best agreement is highlighted in grey.

### Analysis 3: Agreement between raters and automated algorithms (139 words)

**Fig 5:**
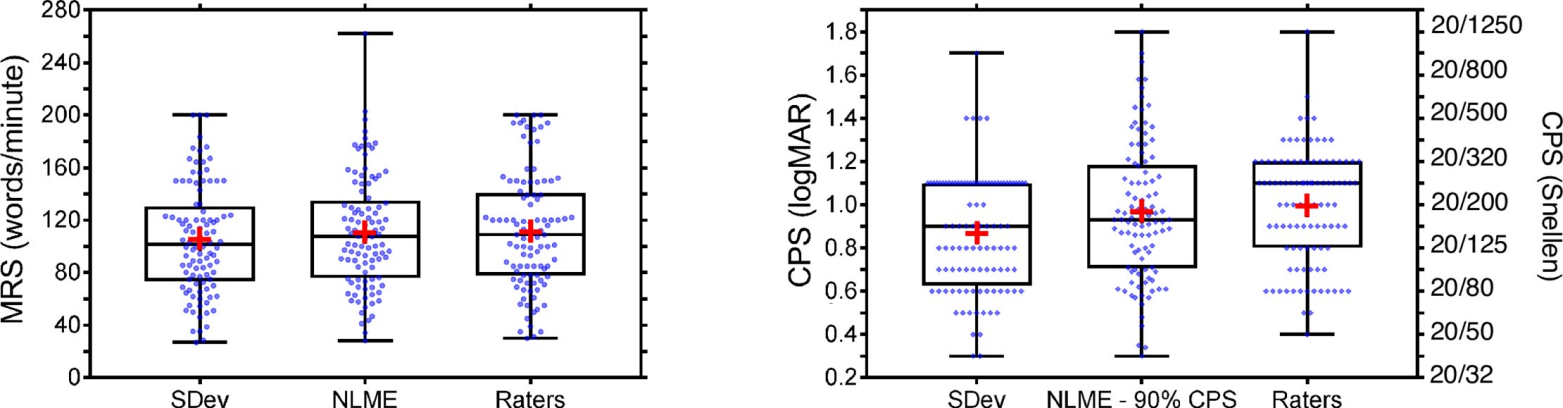
Box and whisker plots showing MRS (left panel) and CPS (right panel) obtained with the two algorithms and the mean for all raters. The box represents 25th to 75th percentile with median line, the red + sign represents the mean and the whiskers represent minimum to maximum.

For MRS, the absolute agreement between raters (k = 7) and automated algorithms was excellent for both the SDev method (ICC = 0.96; 95% CI [0.88, 0.98] and the NLME method (ICC = 0.97; 95% CI [0.95, 0.98]). For CPS, the absolute agreement between raters and the SDev method ranged from ‘poor’ to ‘good’ (ICC = 0.66; 95% CI [0.3, 0.80]), whereas agreement between raters and the NLME method was ‘good’ for CPS defined as 90% of MRS (ICC = 0.83; 95% CI [0.76, 0.88]). Table 4 summarizes the ICC values for each of the five CPS definitions. The NLME method showed better agreement with the raters than the SDev method for both reading parameters. Fig 5 shows the MRS and CPS obtained by the automated algorithms and the 7 raters.

**Table 4:** Absolute agreement (ICC values and their 95% confidence intervals) between CPS values estimated by the raters and with the NLME method for five different cut-off values of CPS.

	ICC value	95% CI	Absolute agreement
95% CPS	0.78	[0.61, 0.87]	Moderate to good
**90% CPS**	**0.83**	**[0.76, 0.88]**	**Good**
85% CPS	0.79	[0.55, 0.71]	Moderate
80% CPS	0.72	[0.18, 0.88]	Poor to good
75% CPS	0.66	[0.02, 0.87]	

Best agreement is highlighted in grey.

## Discussion

Repeatability of the MNREAD chart measures has been assessed before in low-vision populations. Overall, studies have reported good intra and inter-session reliability [11,27–29], as well as good repeatability across multiple testing sites and experimenters [30]. However, to our knowledge, the variability of the MNREAD estimates scored by different raters and/or methods has never been evaluated before. This question of inter-rater variability is especially relevant (1) in the context of multisite investigations, in which data may be scored by different raters with different levels of expertise, (2) when comparing results from different studies performed by different groups, or (3) when looking at follow-up data involving different raters. With the aim to increase knowledge on inter-rater reliability of reading measures, we have chosen to study this question using the MNREAD acuity chart in order to remain consistent with the existing literature, allowing reliable comparisons with previous results of intra and inter-session reliability. In the present work we investigated *i)* the agreement between raters for MNREAD parameters extracted from reading curves (Analysis 1), *ii)* the agreement between SDev and NLME automated methods extracting reading parameters from raw data (Analysis 2) and *iii)* the agreement between raters and automated methods (Analysis 3).

Our first main result was that inter-rater reliability can be classified as ‘excellent’ for MRS (ICC of 0.97) and ‘moderate’ to ‘good’ for CPS (ICC of 0.77). Because they are lower than 1, these agreement indexes reveal the existence of discrepancies when extracting MNREAD parameters visually from reading curves. Whilst the variability for MRS can be considered residual, the CPS estimation may be questionable. On average, the range of difference in CPS estimates was 0.19 logMAR (i.e. almost 2 lines on a logMAR chart), implying that the variability among raters can be considered clinically significant and potentially problematic, for example, when CPS is used to prescribe optimal magnifying power. To identify the underlying factors of the discrepancies observed in CPS rating, we considered whether the data itself could be involved, hypothesizing that the modest ICC value that we found (0.77) was largely due to the presence of highly noisy data. To confirm this hypothesis, we identified extreme outliers for which CPS values were three times larger than the standard deviation of the mean. A total of five curves (5%) were identified as extreme outliers (#2, #31, #58, #70 and #89 in S1 Appendix). What these curves have in common is the lack of a clear plateau and/or the lack of a clear drop point. After removing these five outliers, inter-rater reliability for CPS improved from ‘*moderate to good’* to ‘*good*’, with an increased ICC value of 0.82 (95%CI [0.76, 0.87]). It is worth noting that this new measure of agreement (including the ICC value and its 95% CI) was virtually identical to the agreement measured among experienced raters. Despite modest, this increase suggests that to improve inter-rater reliability, ambiguous cases of noisy data should be discussed before final estimates of CPS are reached. Therefore, the advice for our fellow clinicians and researchers is to inspect our 5 ambiguous samples and define how to deal with such cases on an individual basis whilst maintaining consistency in data extraction. The tips provided in S2 Appendix on how to score ambiguous data can serve as a starting point. When possible, measurements should be repeated to help with the interpretation of problematic data.

We also found that the inter-rater reliability for CPS was marginally poorer among less experienced raters (where it was found to be ‘moderate’ to ‘good’) compared to experienced ones (where it was found to be ‘good’). We speculate that this tendency may be related to both the lack of experience in administrating and rating the test that would lead more naïve raters to follow strictly the definitions of CPS and MRS. Taking the example of curve #2 (see S1 Appendix), both raters SM and AC (self-reported expertise scores of 3 and 4) estimated CPS to be 0.7 logMAR (20/100; 6/30) with MRS = 68 wpm, whilst TQ and KB (self-reported expertise score of 0 and 2) estimated CPS to be 1.3 logMAR (20/400; 6/120) with MRS = 85 wpm; and 1.1 logMAR (20/250; 6/75) with MRS = 75 wpm, respectively. In this case, the more experienced raters (SM and AC) may have decided to ignore the outlier initial data point, assuming that this measure resulted from experimental noise.

Our second main result is the ‘excellent’ agreement between the two automated methods for MRS. It is worth noting that we found a relatively small advantage for NLME (5.8 wpm, i.e. 4.5%). This difference was statistically significant, but may be considered non clinically relevant as it falls within the 8.5 wpm test-retest coefficient of repeatability reported in normal readers [29]. Regarding CPS estimation, the NLME method provides more flexibility over the SDev method because it allows us to determine CPS for different levels of MRS. For instance a higher, more conservative criterion, can be chosen for fluent reading while a lower criterion would be preferred for spot reading. However, there is no rule yet on how to set this criterion optimally to increase reliability. Our results show that the reading speed cut-off to determine CPS yielding the best reliability between methods is 80% of the MRS. This result resonates with conclusions from Cheung et al. 2008, who showed that agreement between NLME method using a two-limb function and an exponential decay function was greater if CPS was set at 80% MRS [17]. On the question of test-retest reliability, Patel et al. 2011 also reported that using a criterion of 80% yield improved repeatability of the CPS (when compared to 90%) [11]. While an optimal criterion should be chosen on a case-by-case basis depending on the clinician or researcher’s motivations, the evidence suggests that a criterion close to 80% would increase both inter-rater and test-retest variability.

Our third result is that raters and automated methods show ‘excellent’ agreement for MRS values (ICC of 0.96 and 0.97 for the SDev and NLME methods, respectively). The agreement for CPS was more variable. It was ‘poor’ to ‘moderate’ for the SDev (ICC of 0.66) and ‘good’ for the NLME (ICC of 0.83 with a CPS criterion set to 90% MRS). It is worth noting that ICC values were almost identical when measuring agreement between raters and agreement between algorithms for both MRS and CPS. This comparison is important because it indicates somehow the robustness and efficacy of human visual inspection of MNREAD curves when compared to automated methods. This observation is especially relevant considering that algorithms present two major drawbacks: (1) they may not be easily accessible in clinical environments, (2) they may fail to provide satisfactory measures with noisy data points or small and incomplete datasets, which require human inspection of the curves for validation.

Overall, these results are likely applicable to researchers and clinicians, whether they use computational methods, or the traditional pen-and-paper scoring technique. Indeed, our findings allow to extend the currently available MNREAD instructions, by focusing on extraction of test results from atypical curves. First, we point that these atypical reading profiles are mainly driven by clinical factors such as the presence of a ring scotoma or unpredictable preferred retinal loci, which is fundamentally relevant for clinical use. Second, the examples and comments provided in our detailed instructions (S2 Appendix) should support clinicians to extract meaningful information from these atypical reading profiles and score them optimally. By extension, this should increase reproducibility and confidence in test results amongst clinicians and researchers. Finally, our results are also relevant for clinicians currently using the hardcopy MNREAD chart and willing to use the MNREAD iPad app, by showing that the automated scoring provided by the app is consistent with manual scoring, given a routine visual inspection of the automatically generated curves.

This work presents some limitations. First, despite the relatively large sample of MNREAD data considered in the present work, it is hard to predict to what extent the different shaped curves are representative of all possible curves. Second, it is likely that the new instructions helped to reduce inter-rater variability, but there is no data to support this conclusion. Despite all raters used the new set of extended instructions the ICC value for CPS was still suboptimal, suggesting that additional fixes should be considered to help increase reliability. Whenever possible repeated measures should be performed, using the different tests version available [16,31]. Repeated measures would make it easier for the rater to determine whether a measure should be considered as noise or not. Another possibility might be to pool estimates from multiple raters or in combination with curve fits. Third, the finding that 80% MRS yields the most reliable CPS using the NLME method is convenient to parameterize the curve in research studies using curve fitting but is only moderately relevant in the context of low-vision rehabilitation, where the goal is to enlarge text so that it can be read at the reader’s MRS, not at the 80% of the reader’s MRS.

## Conclusions

In summary, our study shows that extraction of the maximum reading speed from MNREAD data is consistent across methods and researchers. It also reveals that for curves obtained from readers with low vision it is difficult to obtain excellent inter-rater reliability for CPS estimates. Future studies, such as rehabilitation interventions aiming at improving reading ability in people with low vision, can now follow the advice and instructions resulting from our investigation. Using a standard set of instructions and criteria to analyze reading curves may help increase the reliability of the results. Additional ways to improve inter-rater reliability should also be considered, e.g. use the curve fits, collect multiple runs per participant or combine the estimates of multiple raters.

## Supporting information

S1 Appendix Individual MNREAD curves from the 101 MNREAD measurements. (PDF)

S2 Appendix Detailed scoring instructions provided to the raters. (PDF)

S3 Appendix 101 MNREAD curves fitted by the NLME model. (PDF)

## References

[1] Brown JC, Goldstein JE, Chan TL, Massof R, Ramulu P, Low Vision Research Network Study Group (2014) Characterizing functional complaints in patients seeking outpatient low-vision services in the United States. Ophthalmology 121: 1655–62.e1. 10.1016/j.ophtha.2014.02.030 24768243 PMC6746569

[2] Frost NA, Sparrow JM, Durant JS, Donovan JL, Peters TJ, Brookes ST (1998) Development of a questionnaire for measurement of vision-related quality of life. Ophthalmic Epidemiol 5: 185–210. 9894804 10.1076/opep.5.4.185.4191

[3] Hart PM, Chakravarthy U, Stevenson MR, Jamison JQ (1999) A vision specific functional index for use in patients with age related macular degeneration. Br J Ophthalmol 83: 1115–20. 10.1136/bjo.83.10.1115 10502569 PMC1722820

[4] Mangione CM, Lee PP, Gutierrez PR, Spritzer K, Berry S, Hays R (2001) Development of the 25-list-item national eye institute visual function questionnaire. Archives of Ophthalmology 119: 1050–1058. 11448327 10.1001/archopht.119.7.1050

[5] Massof RW, Hsu CT, Baker FH, Barnett GD, Park WL, Deremeik JT, et al (2005) Visual Disability Variables. I: The Importance and Difficulty of Activity Goals for a Sample of Low-Vision Patients. Archives of Physical Medicine and Rehabilitation 86: 946–953. 10.1016/j.apmr.2004.09.016 15895341

[6] Friedman SM, Munoz B, Rubin GS, West SK, Bandeen-Roche K, Fried LP (1999) Characteristics of discrepancies between self-reported visual function and measured reading speed. Salisbury Eye Evaluation Project Team. Invest Ophthalmol Vis Sci 40: 858–64. 10102282

[7] Mansfield JS, Ahn SJ, Legge GE, Luebker A (1993) A new reading-acuity chart for normal and low vision. Ophthalmic and Visual Optics/Noninvasive Assessment of the Visual System Technical Digest, (Optical Society of America, Washington, DC., 1993) 3: 232–235.

[8] Calabrèse A, Owsley C, McGwin G, Legge GE (2016a) Development of a Reading Accessibility Index Using the MNREAD Acuity Chart. JAMA Ophthalmol 134: 398–405. 26868760 10.1001/jamaophthalmol.2015.6097 PMC5369600

[9] Macedo AF, Nascimento SMC, Gomes AOS, Puga AT (2007) Fixation in patients with juvenile macular disease. Optom Vis Sci 84: 852–8. 10.1097/OPX.0b013e3181559beb 17873770

[10] Finger RP, Charbel Issa P, Fimmers R, Holz FG, Rubin GS, Scholl HPN (2009) Reading performance is reduced by parafoveal scotomas in patients with macular telangiectasia type 2. Invest Ophthalmol Vis Sci 50: 1366–70. 10.1167/iovs.08-2032 18997085

[11] Patel PJ, Chen FK, Da Cruz L, Rubin GS, Tufail A (2011) Test-retest variability of reading performance metrics using MNREAD in patients with age-related macular degeneration. Invest Ophthalmol Vis Sci 52: 3854–9. 10.1167/iovs.10-6601 21421873

[12] Rubin GS (2013) Measuring reading performance. Vision Res 90: 43–51. 10.1016/j.visres.2013.02.015 23506967

[13] Cudeck R, Harring R Jeffrey (2010) Developing a random coefficient model for nonlinear repeated measures data In Chow S.-M., Ferrer E., & Hsieh F. (Eds.). The Notre Dame series on quantitative methodology. Statistical methods for modeling human dynamics: An interdisciplinary dialogue (pp. 289–318). New York, NY, US: Routledge/Taylor & Francis Group.

[14] Legge GE (2007) Psychophysics of reading in normal and low vision. Mahwah: NJ & London: Lawrence Erlbaum Associates.

[15] Mansfield JS, Legge GE, Bane MC (1996) Psychophysics of reading XV—Font effects in normal and low vision. Invest Ophthalmol Vis Sci 37: 1492–501. 8675391

[16] Calabrèse A, To L, He Y, Berkholtz E, Rafian P, Legge GE (2018a) Comparing performance on the MNREAD iPad application with the MNREAD acuity chart. J Vis 18: 8. 10.1167/18.1.8 PMC5774869 29351351

[17] Cheung SH, Kallie CS, Legge GE, Cheong AM (2008) Nonlinear Mixed-Effects Modeling of MNREAD Data. Invest Ophthalmol Vis Sci 49: 828–35. 10.1167/iovs.07-0555 18235034

[18] Macedo AF, Ramos PL, Hernandez-Moreno L, Cima J, Baptista AMG, Marques AP, et al (2017) Visual and health outcomes, measured with the activity inventory and the EQ-5D, in visual impairment. Acta Ophthalmol 95: e783–e791. 10.1111/aos.13430 28371261

[19] Ramos PL, Santana R, Moreno LH, Marques AP, Freitas C, Rocha-Sousa A, et al (2018) Predicting participation of people with impaired vision in epidemiological studies. BMC Ophthalmol 18: 236 10.1186/s12886-018-0889-9 30180834 PMC6123934

[20] Marques AP, Macedo AF, Hernandez-Moreno L, Ramos PL, Butt T, Rubin G, et al (2018) The use of informal care by people with vision impairment. PLoS One 13: e0198631 10.1371/journal.pone.0198631 29879193 PMC5991749

[21] Tamaki Monteiro de Castro C, Kallie CS, Salomão SR (2005) [Development and validation of the MNREAD reading acuity chart in Portuguese]. Arq Bras Oftalmol 68: 777–83. 17344979 10.1590/s0004-27492005000600013

[22] R Core Team (2018) R: A Language and Environment for Statistical Computing. Vienna, Austria: R Foundation for Statistical Computing https://www.R-project.org/.

[23] Calabrèse A, Mansfield JS, Legge GE. (2017) mnreadR, an R package to analyze MNREAD data.

[24] Shrout PE, Fleiss JL (1979) Intraclass correlations: uses in assessing rater reliability. Psychol Bull 86: 420–8. 18839484 10.1037//0033-2909.86.2.420

[25] Koo TK, Li MY (2016) A Guideline of Selecting and Reporting Intraclass Correlation Coefficients for Reliability Research. J Chiropr Med 15: 155–63. 10.1016/j.jcm.2016.02.012 27330520 PMC4913118

[26] McGraw KO, Wong SP (1996) Forming inferences about some intraclass correlation coefficients. Psychological Methods 1: 30–46.

[27] Virgili G, Cordaro C, Bigoni A, Crovato S, Cecchini P, Menchini U (2004) Reading acuity in children: evaluation and reliability using MNREAD charts. Invest Ophthalmol Vis Sci 45: 3349–54. 10.1167/iovs.03-1304 15326160

[28] Subramanian A, Pardhan S (2009) Repeatability of reading ability indices in subjects with impaired vision. Invest Ophthalmol Vis Sci 50: 3643–7. 10.1167/iovs.08-2823 19339738

[29] Subramanian A, Pardhan S (2006) The repeatability of MNREAD acuity charts and variability at different test distances. Optom Vis Sci 83: 572–6. 10.1097/01.opx.0000232225.00311.53 16909082

[30] Calabrèse A, Cheong AMY, Cheung S, He Y, Kwon M, Mansfield JS, et al (2016c) Baseline MNREAD Measures for Normally Sighted Subjects From Childhood to Old Age. Invest Ophthalmol Vis Sci 57: 3836–43. 27442222 10.1167/iovs.16-19580 PMC4961000

[31] Mansfield JS, Atilgan N, Lewis A, Legge GE (2019) Extending the MNREAD sentence corpus: Computer-generated sentences for measuring vision for reading. Vision Res 158: 11–18 10.1016/j.visres.2019.01.010 30731097 PMC6538455

