# Supplementary material for: Scoring reading parameters: An inter-rater reliability study using the MNREAD chart
Source: PLoS One. 2019 Jun 7;14(6):e0216775. doi: 10.1371/journal.pone.0216775 (PMC6555504; doi:10.1371/journal.pone.0216775)
Supplement: S1 Appendix — (PDF) [file pone.0216775.s001.pdf]

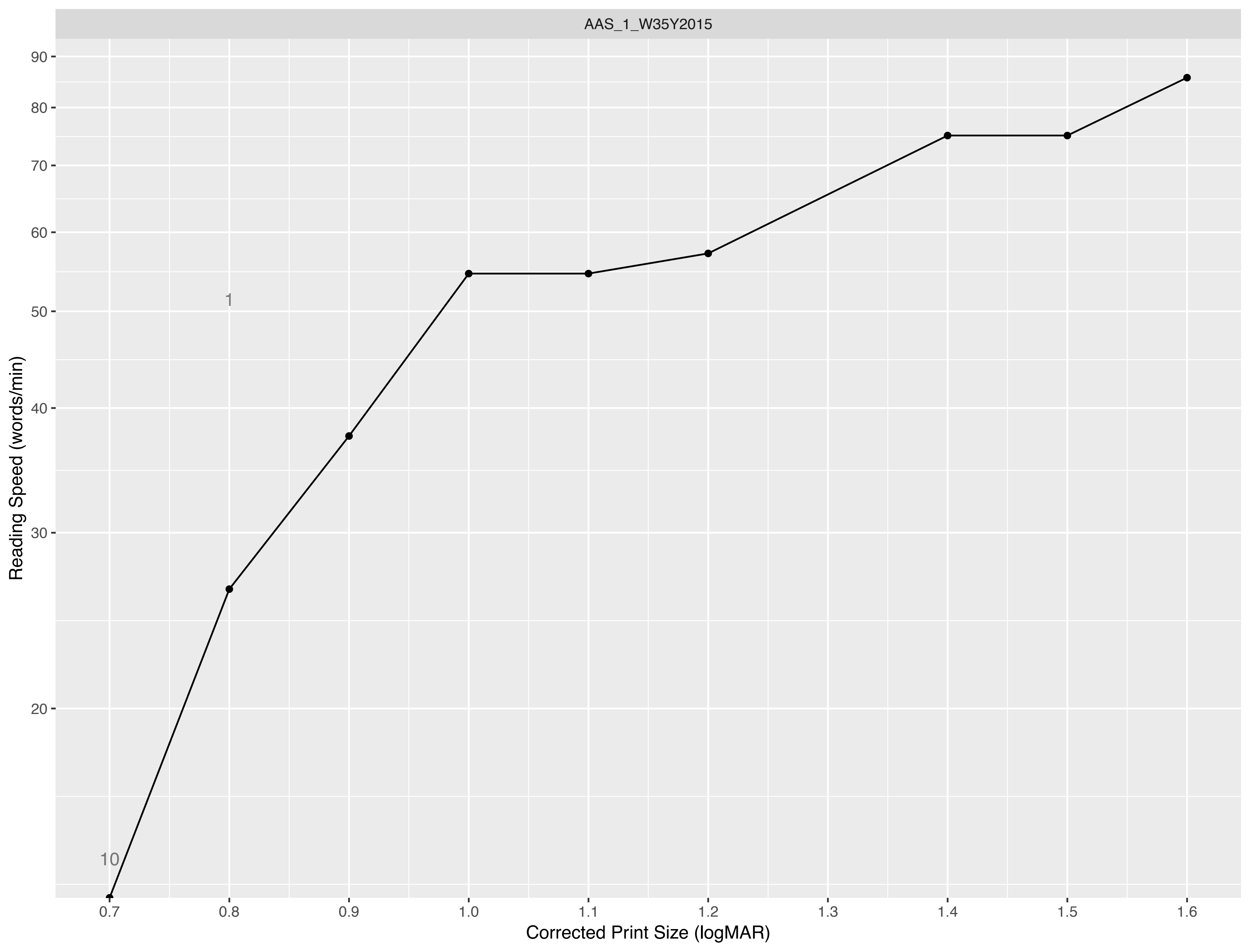

AAS_1_W35Y2015
Reading Speed (words/min)
90
80
70
60
50
40
30
20
Corrected Print Size (logMAR)
0.7
0.8
0.9
1.0
1.1
1.2
1.3
1.4
1.5
1.6
1
10

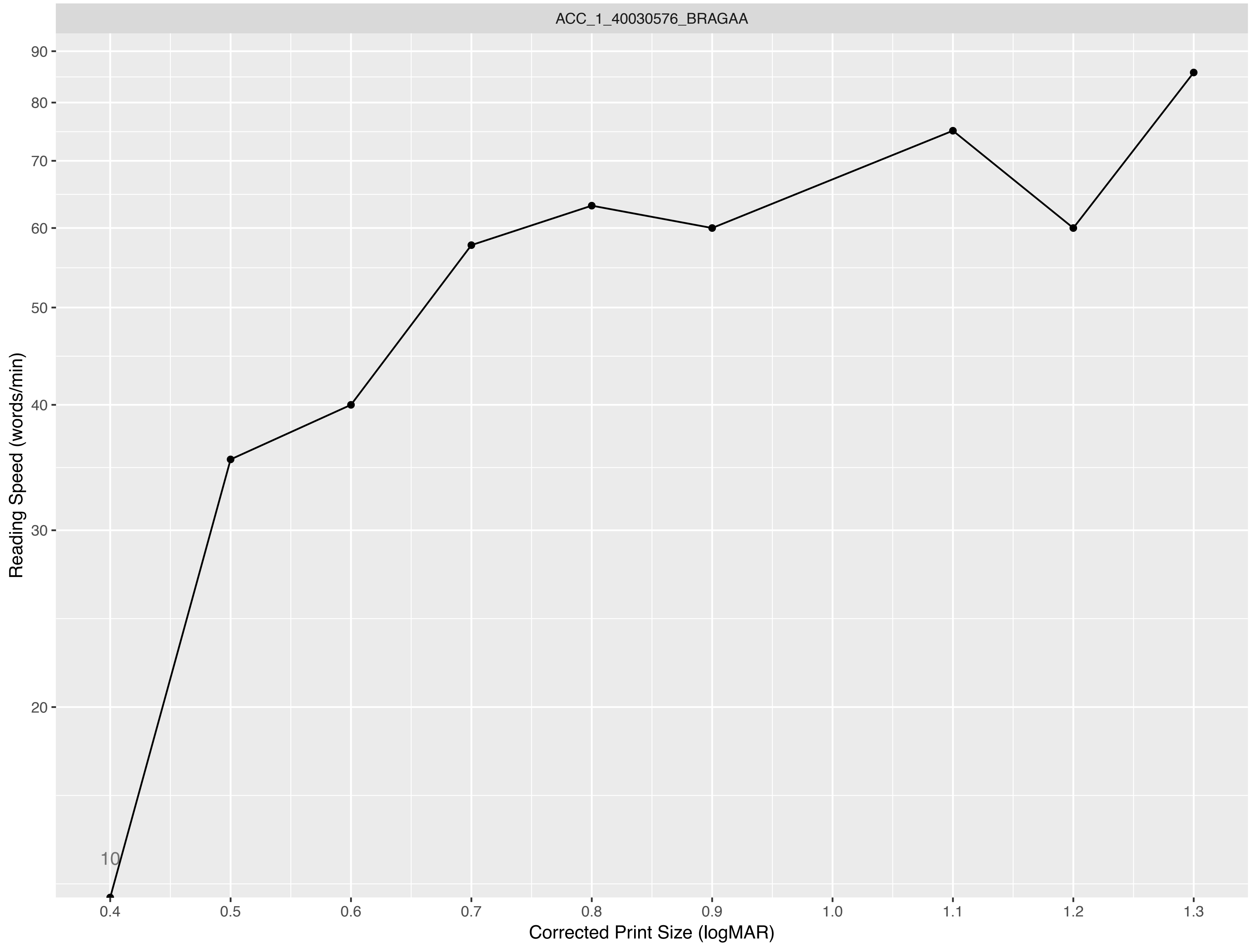
ACC_1_40030576_BRAGAA
Reading Speed (words/min)
90
80
70
60
50
40
30
20
10
0.4
0.5
0.6
0.7
0.8
0.9
1.0
1.1
1.2
1.3
Corrected Print Size (logMAR)

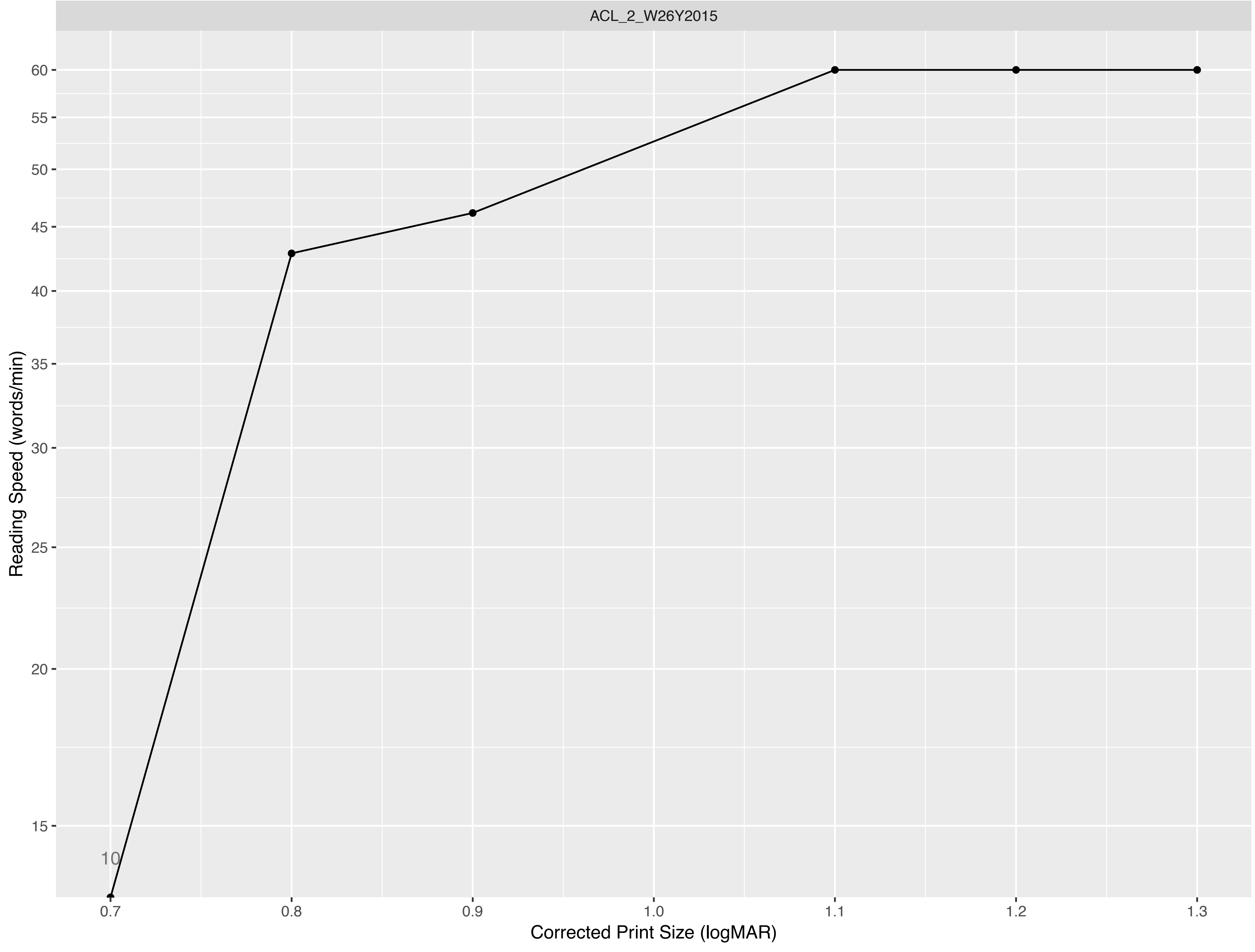

ACL_2_W26Y2015
Reading Speed (words/min)
60
55
50
45
40
35
30
25
20
15
10
0.7
0.8
0.9
1.0
1.1
1.2
1.3
Corrected Print Size (logMAR)

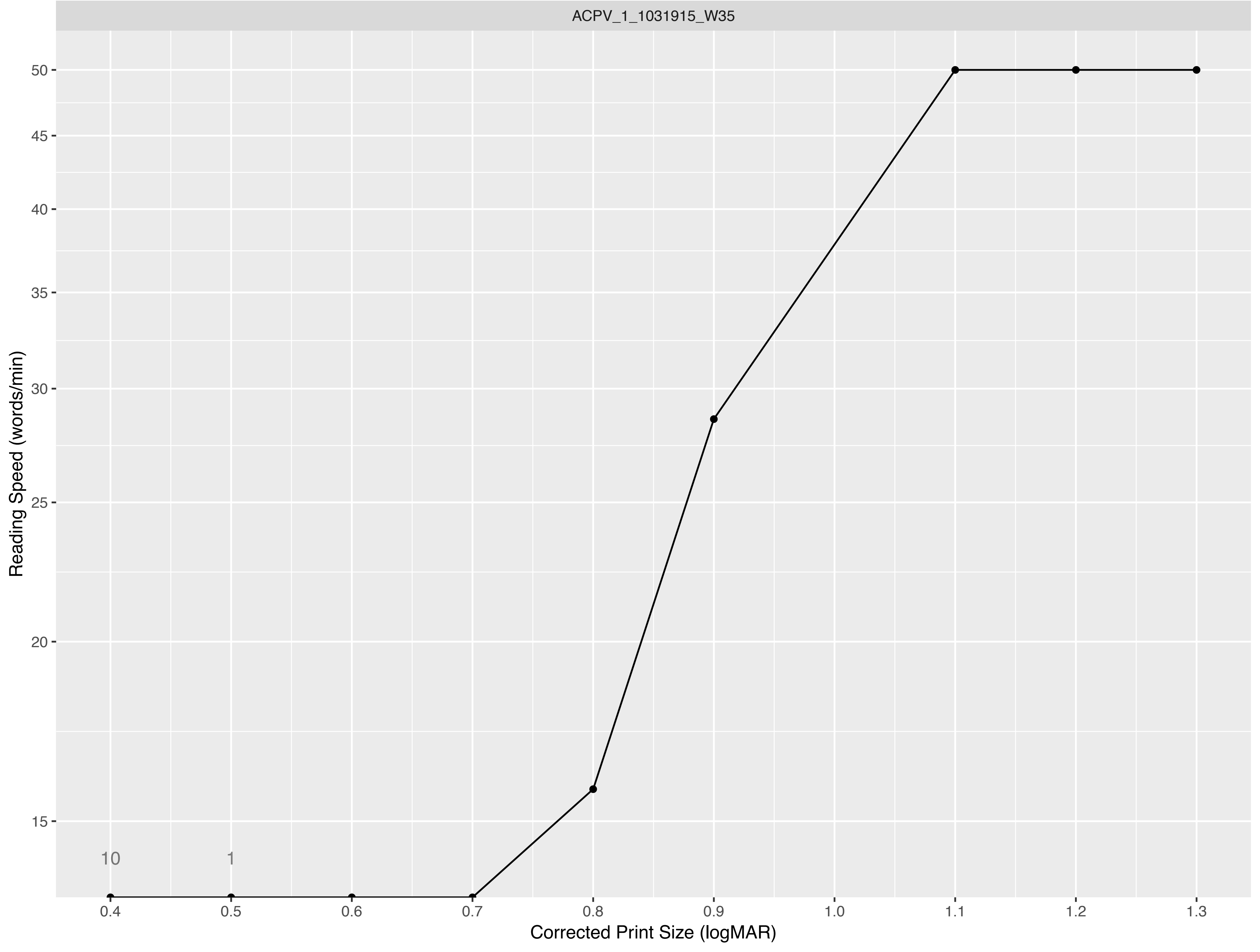

ACPV_1_1031915_W35
Reading Speed (words/min)
15
20
25
30
35
40
45
50
10
1
0.4
0.5
0.6
0.7
0.8
0.9
1.0
1.1
1.2
1.3
Corrected Print Size (logMAR)

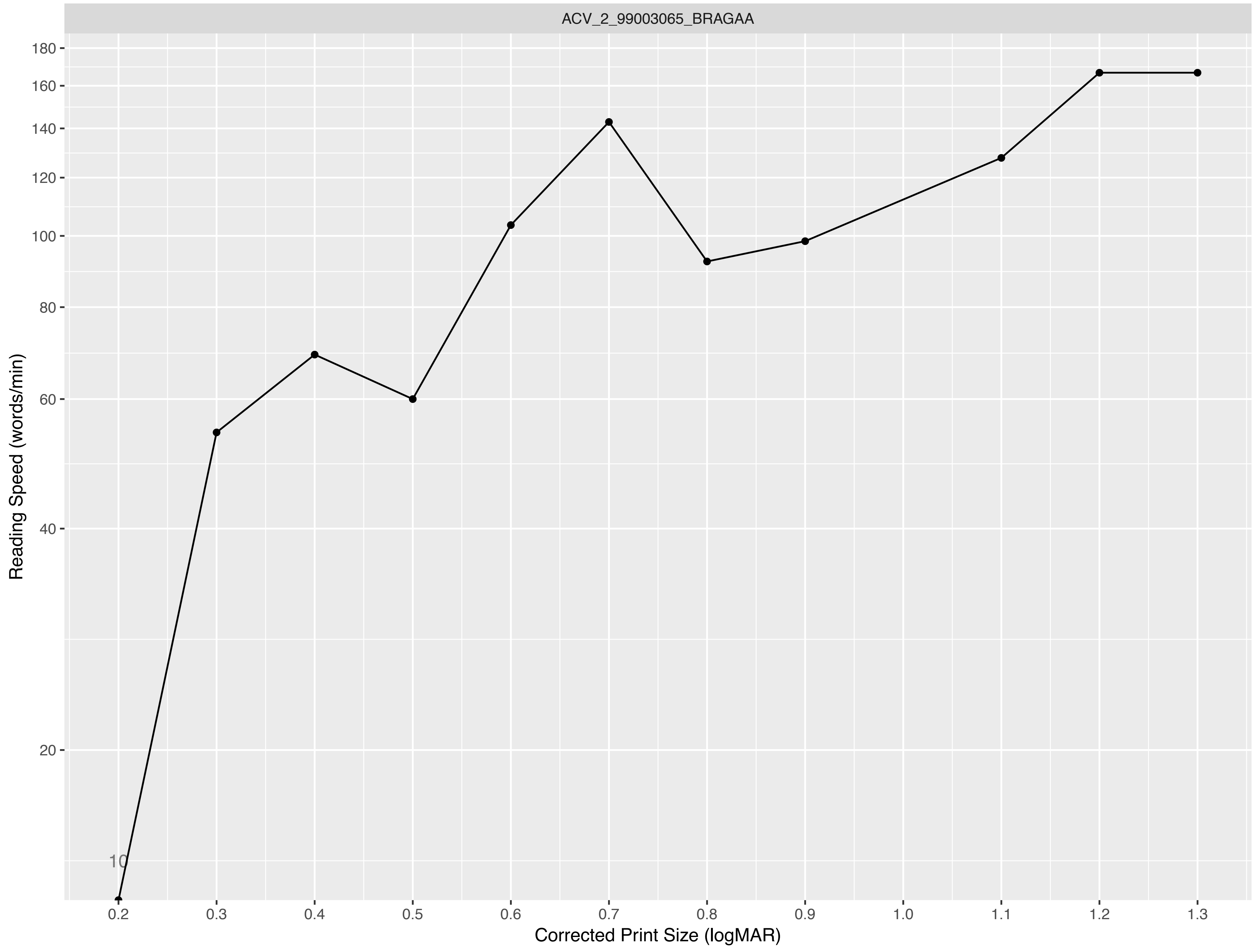

ACV_2_99003065_BRAGAA
Reading Speed (words/min)
180
160
140
120
100
80
60
40
20
10
0.2
0.3
0.4
0.5
0.6
0.7
0.8
0.9
1.0
1.1
1.2
1.3
Corrected Print Size (logMAR)

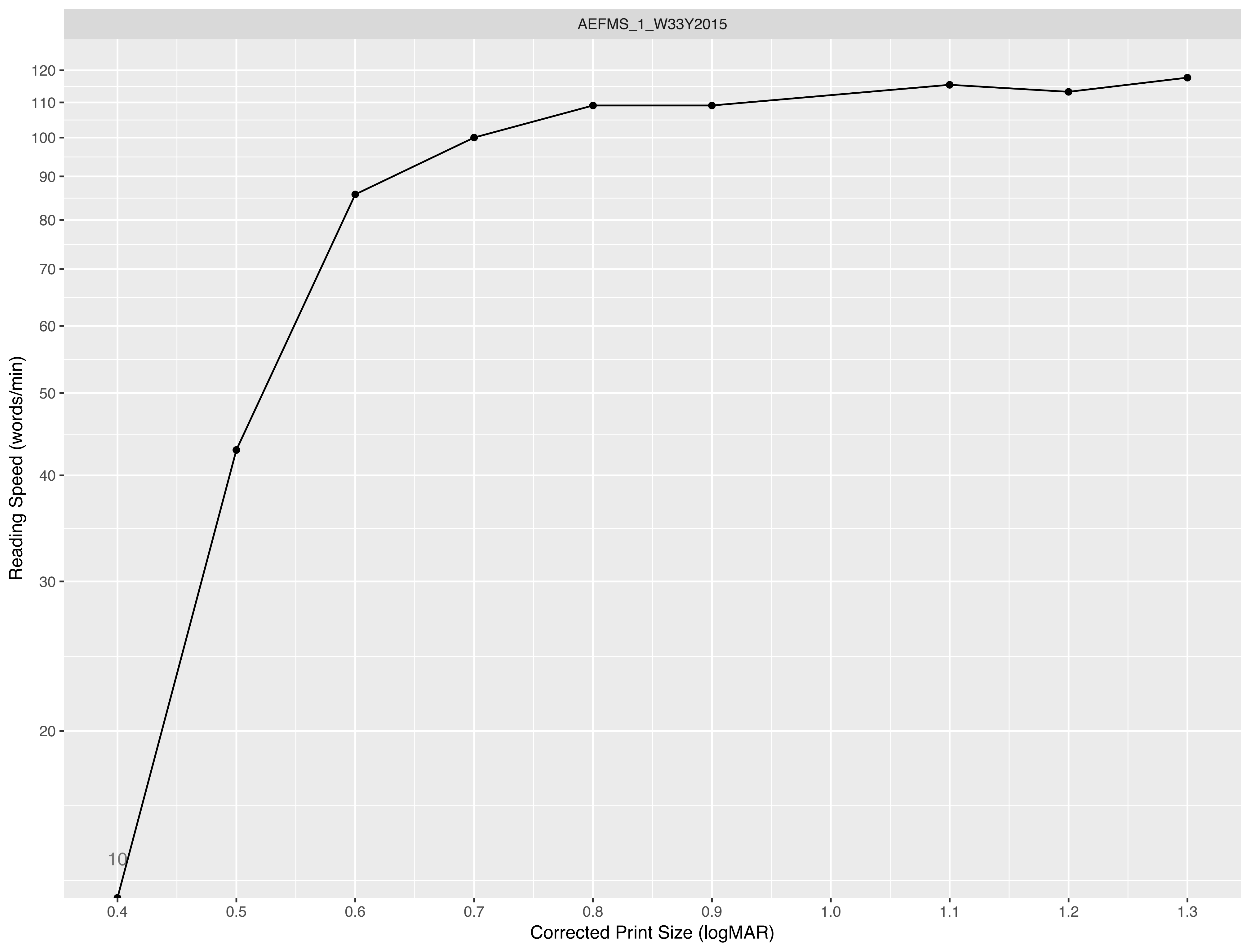

AEFMS_1_W33Y2015
Reading Speed (words/min)
120
110
100
90
80
70
60
50
40
30
20
10
0.4
0.5
0.6
0.7
0.8
0.9
1.0
1.1
1.2
1.3
Corrected Print Size (logMAR)

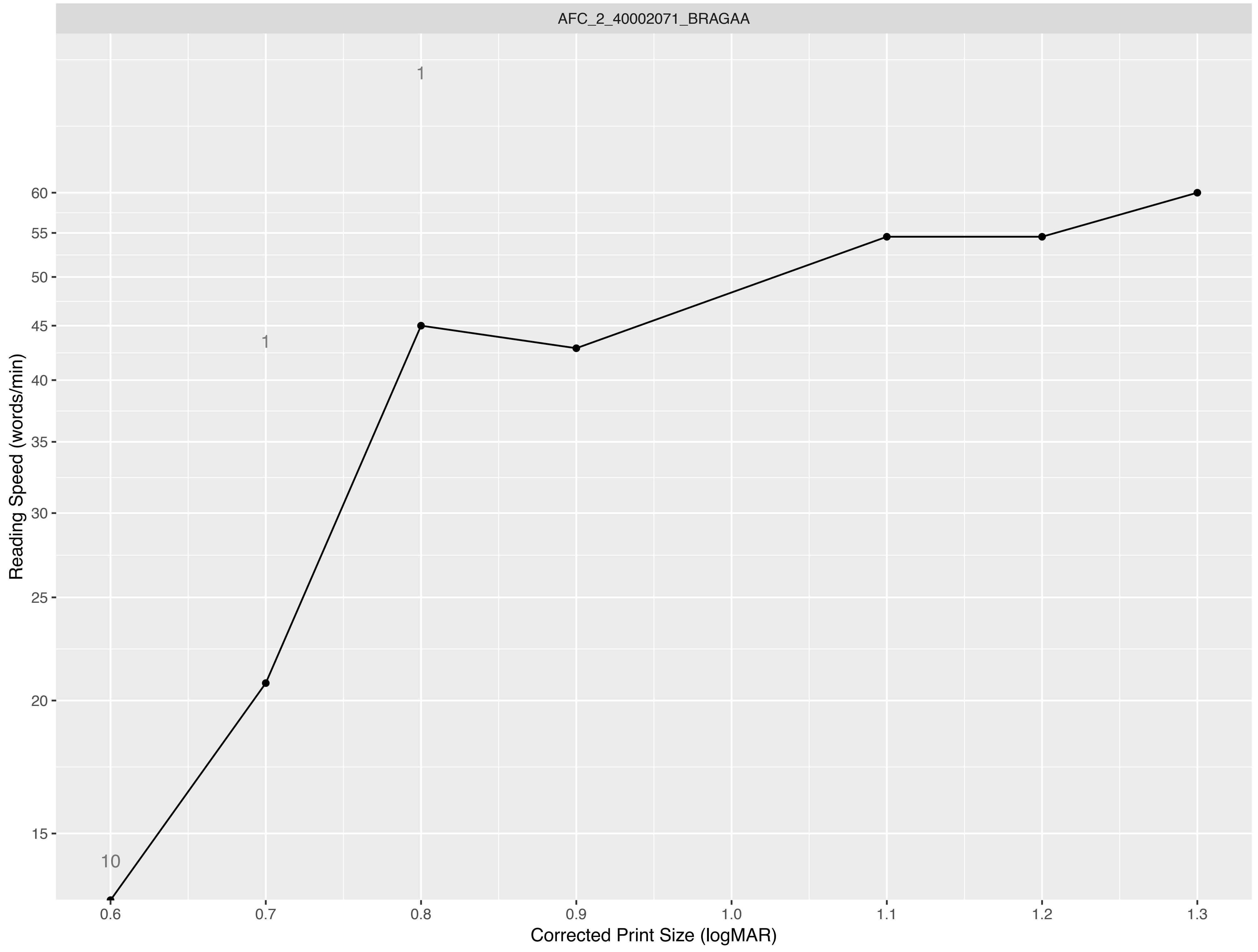

AFC_2_40002071_BRAGAA
Reading Speed (words/min)
60
55
50
45
40
35
30
25
20
15
1
1
10
0.6
0.7
0.8
0.9
1.0
1.1
1.2
1.3
Corrected Print Size (logMAR)

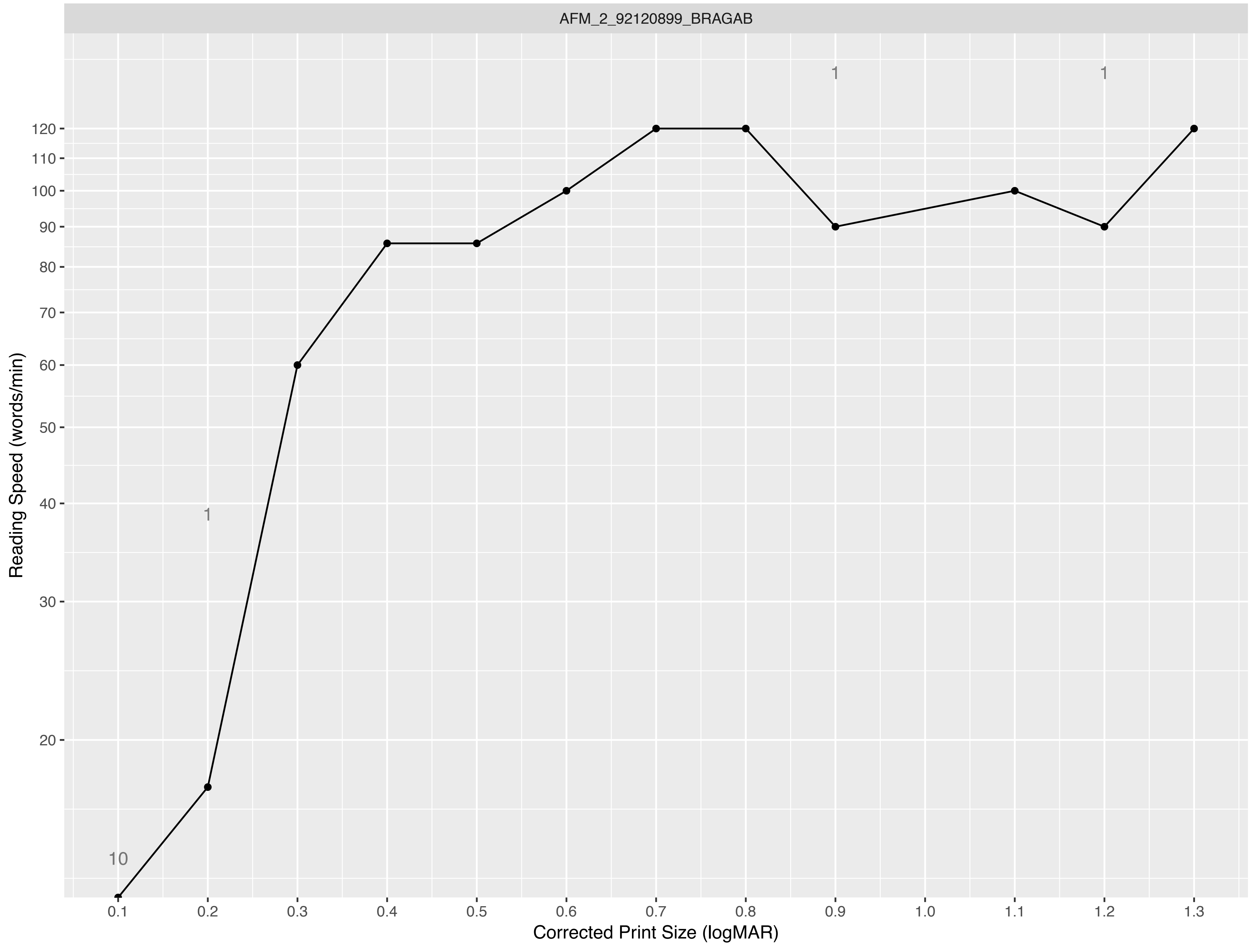
AFM_2_92120899_BRAGAB
Reading Speed (words/min)
Corrected Print Size (logMAR)
120
110
100
90
80
70
60
50
40
30
20
0.1
0.2
0.3
0.4
0.5
0.6
0.7
0.8
0.9
1.0
1.1
1.2
1.3
1
1
1
10

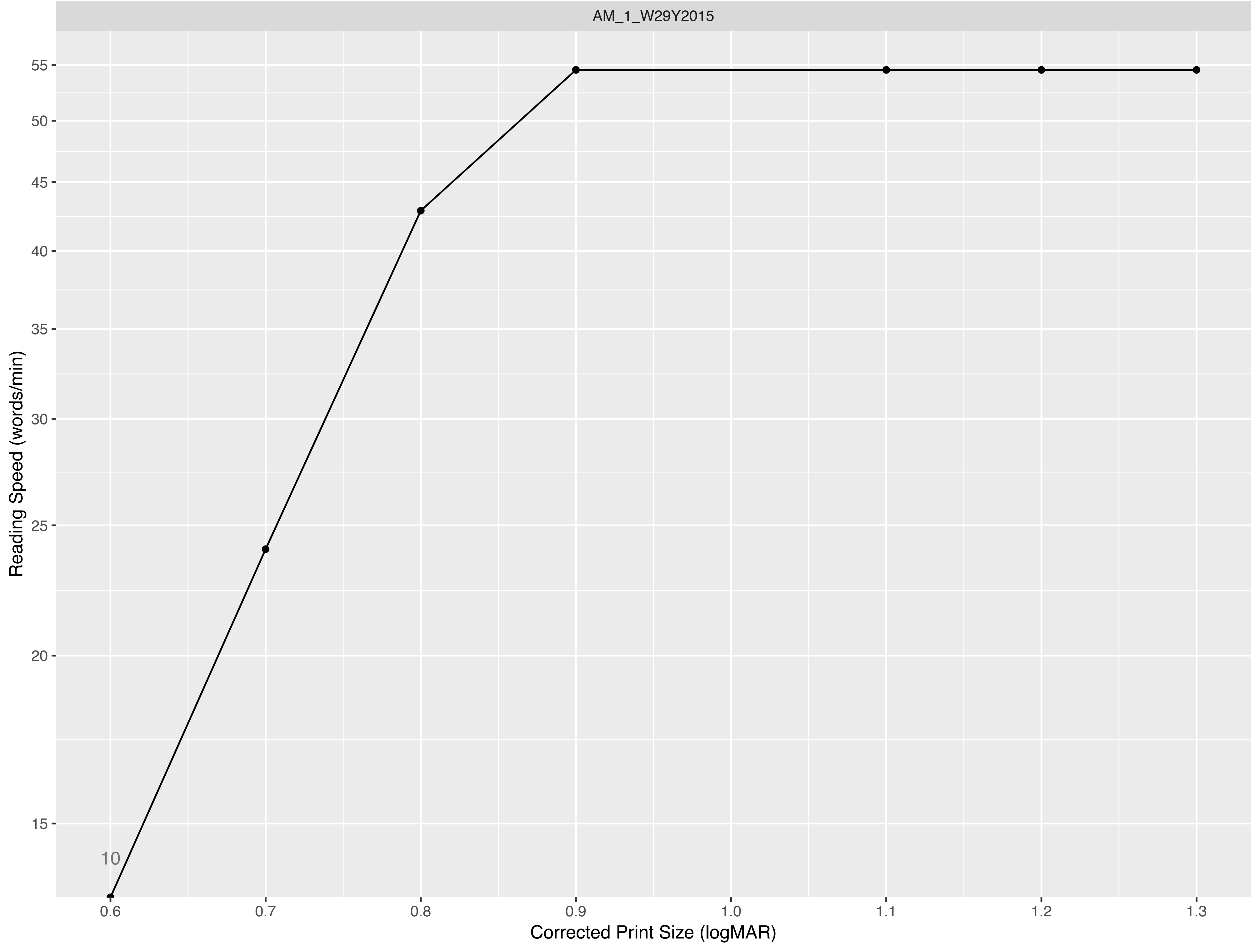
AM_1_W29Y2015
Reading Speed (words/min)
55
50
45
40
35
30
25
20
15
10
0.6
0.7
0.8
0.9
1.0
1.1
1.2
1.3
Corrected Print Size (logMAR)

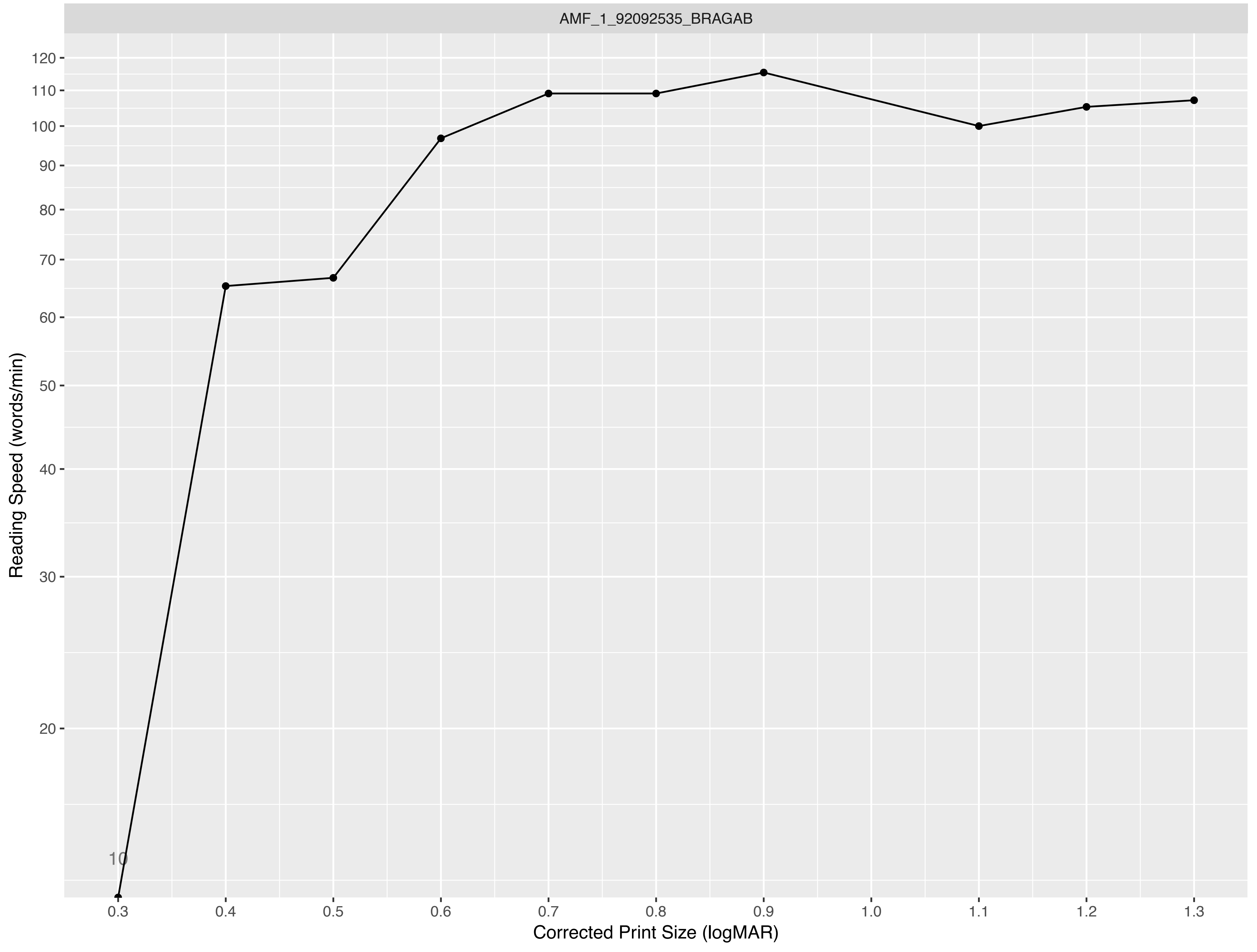

AMF_1_92092535_BRAGAB
Reading Speed (words/min)
120
110
100
90
80
70
60
50
40
30
20
10
0.3
0.4
0.5
0.6
0.7
0.8
0.9
1.0
1.1
1.2
1.3
Corrected Print Size (logMAR)

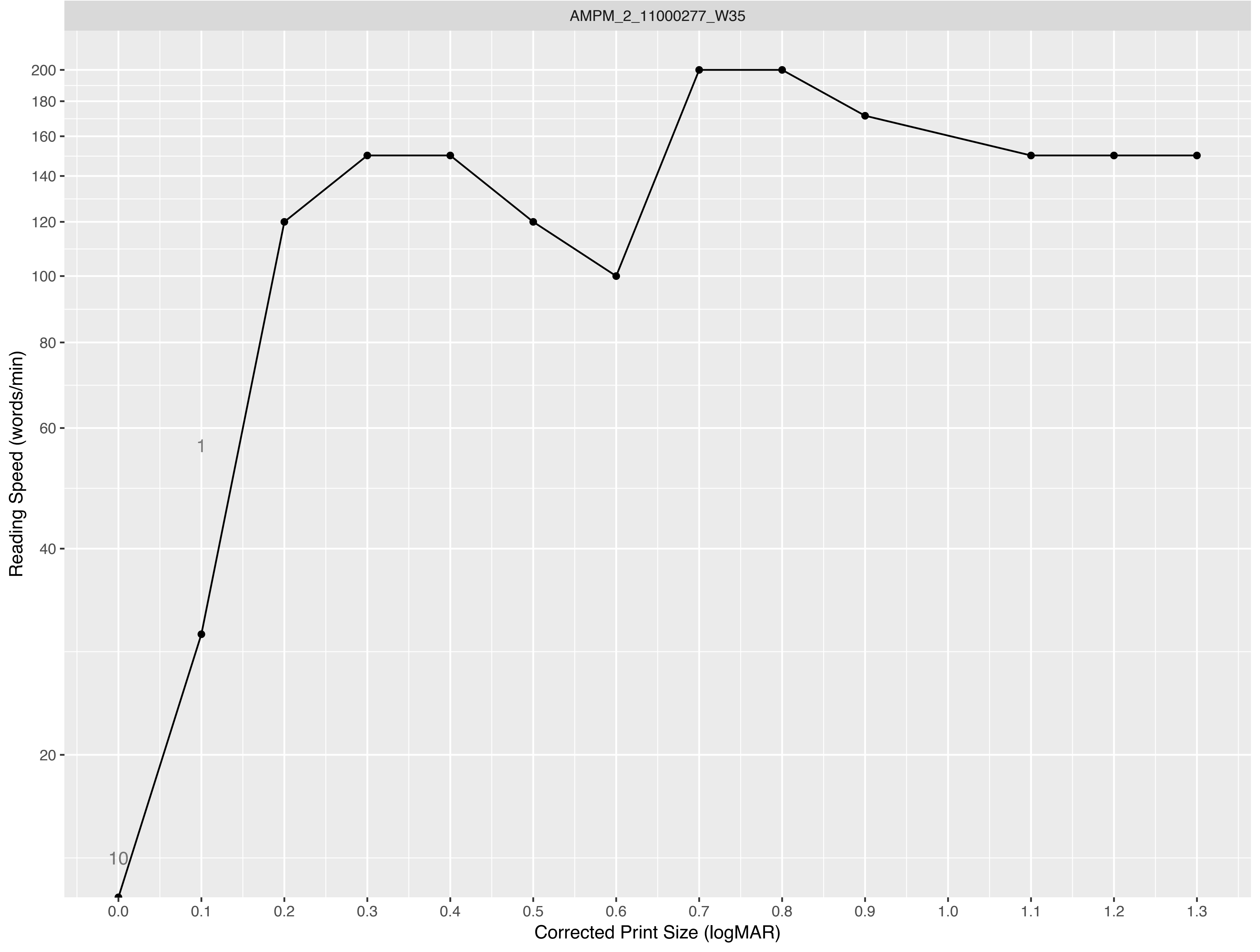
AMPM_2_11000277_W35
Reading Speed (words/min)
200
180
160
140
120
100
80
60
40
20
Corrected Print Size (logMAR)
0.0
0.1
0.2
0.3
0.4
0.5
0.6
0.7
0.8
0.9
1.0
1.1
1.2
1.3
10
1

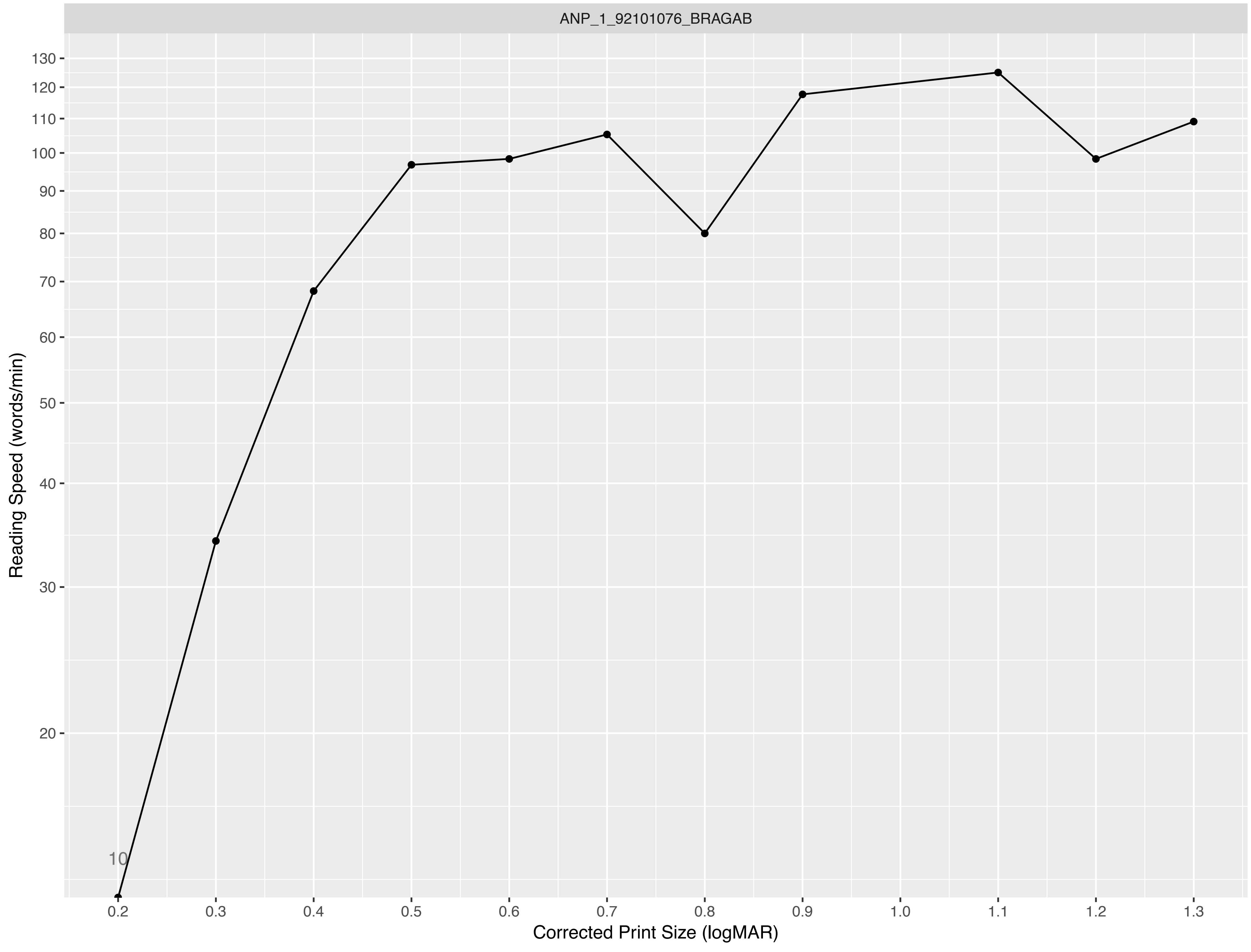
ANP_1_92101076_BRAGAB
Reading Speed (words/min)
130
120
110
100
90
80
70
60
50
40
30
20
10
0.2
0.3
0.4
0.5
0.6
0.7
0.8
0.9
1.0
1.1
1.2
1.3
Corrected Print Size (logMAR)

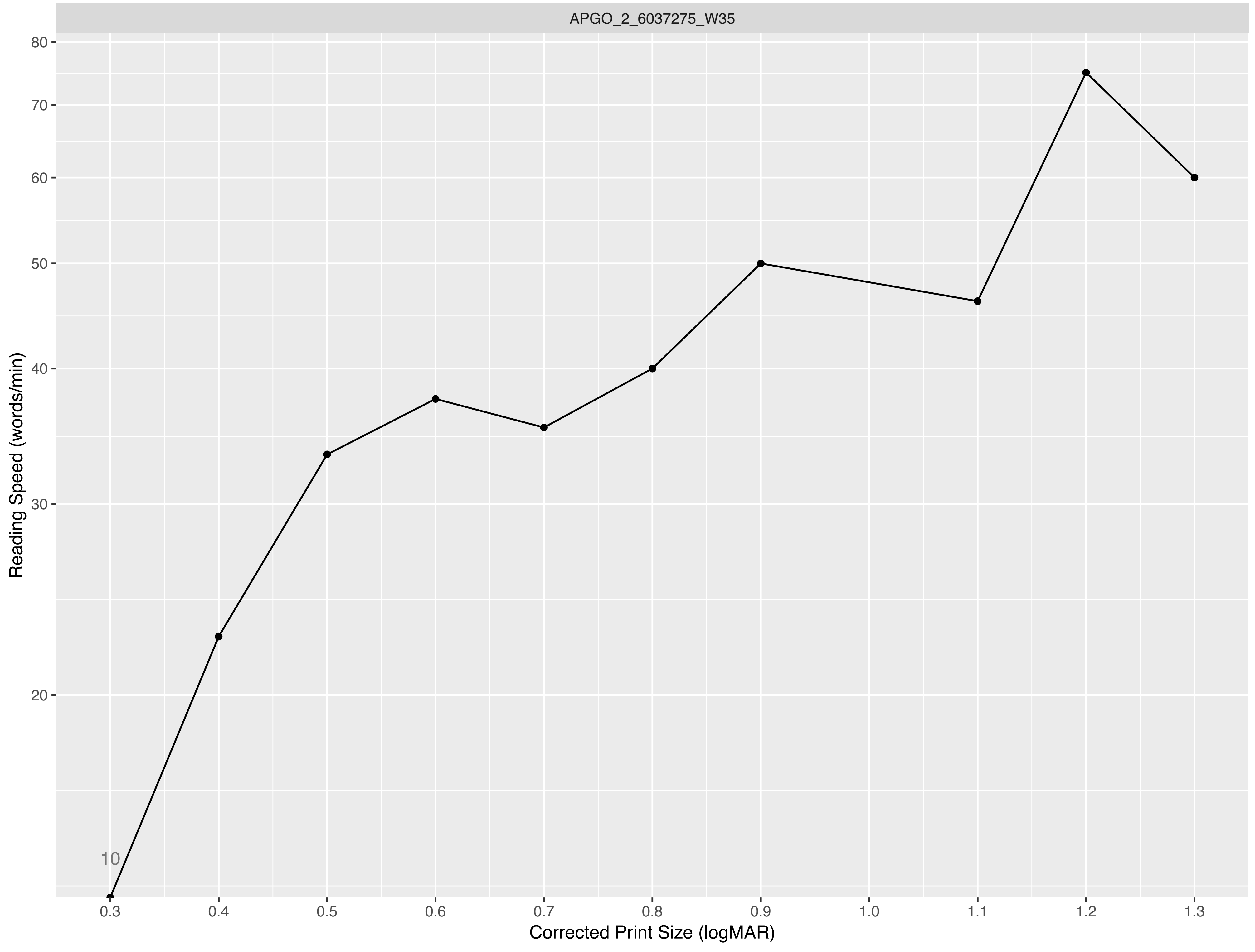
APGO_2_6037275_W35
Reading Speed (words/min)
80
70
60
50
40
30
20
10
0.3
0.4
0.5
0.6
0.7
0.8
0.9
1.0
1.1
1.2
1.3
Corrected Print Size (logMAR)

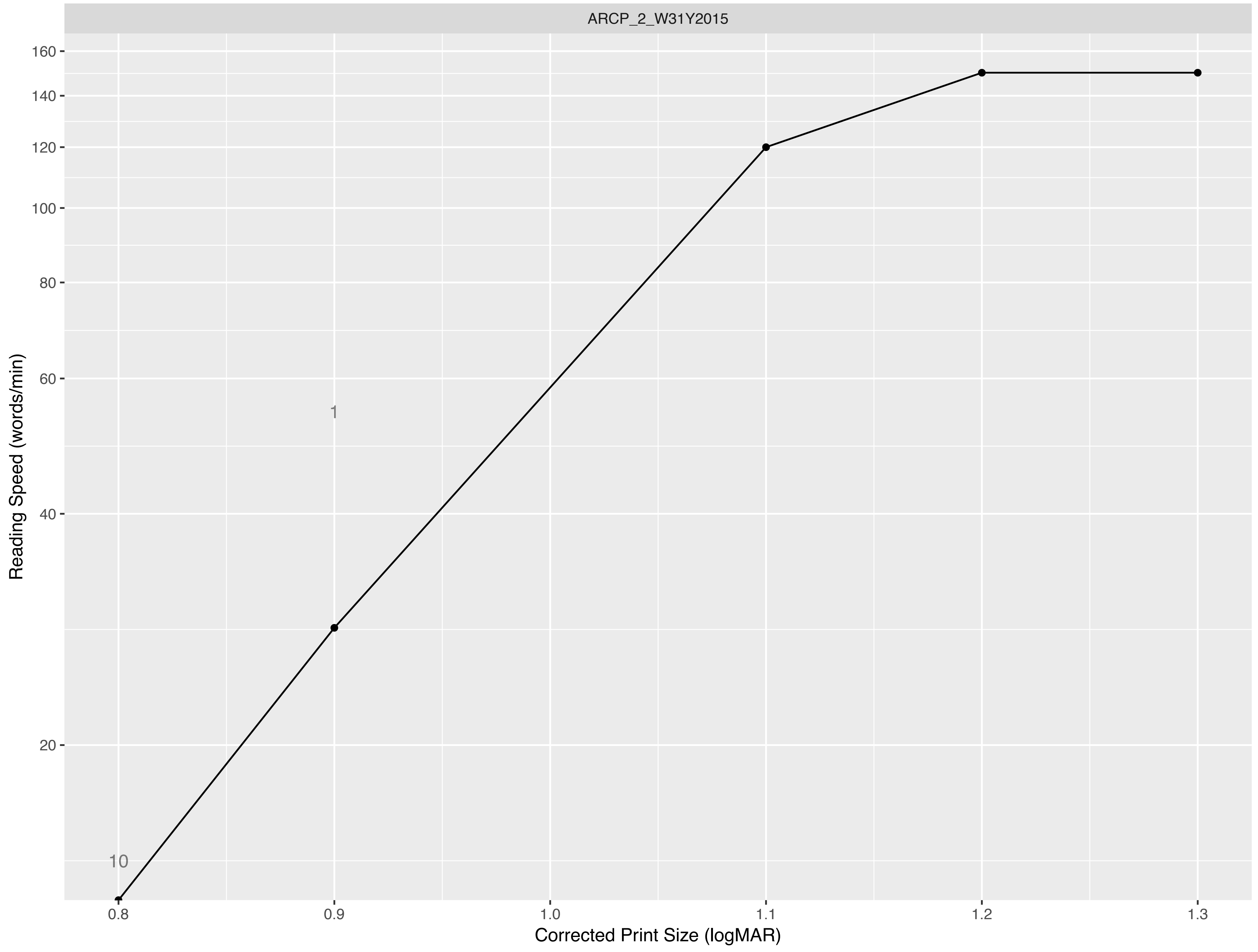
ARCP_2_W31Y2015
160
140
120
100
80
60
40
20
1
10
0.8
0.9
1.0
1.1
1.2
1.3
Reading Speed (words/min)
Corrected Print Size (logMAR)

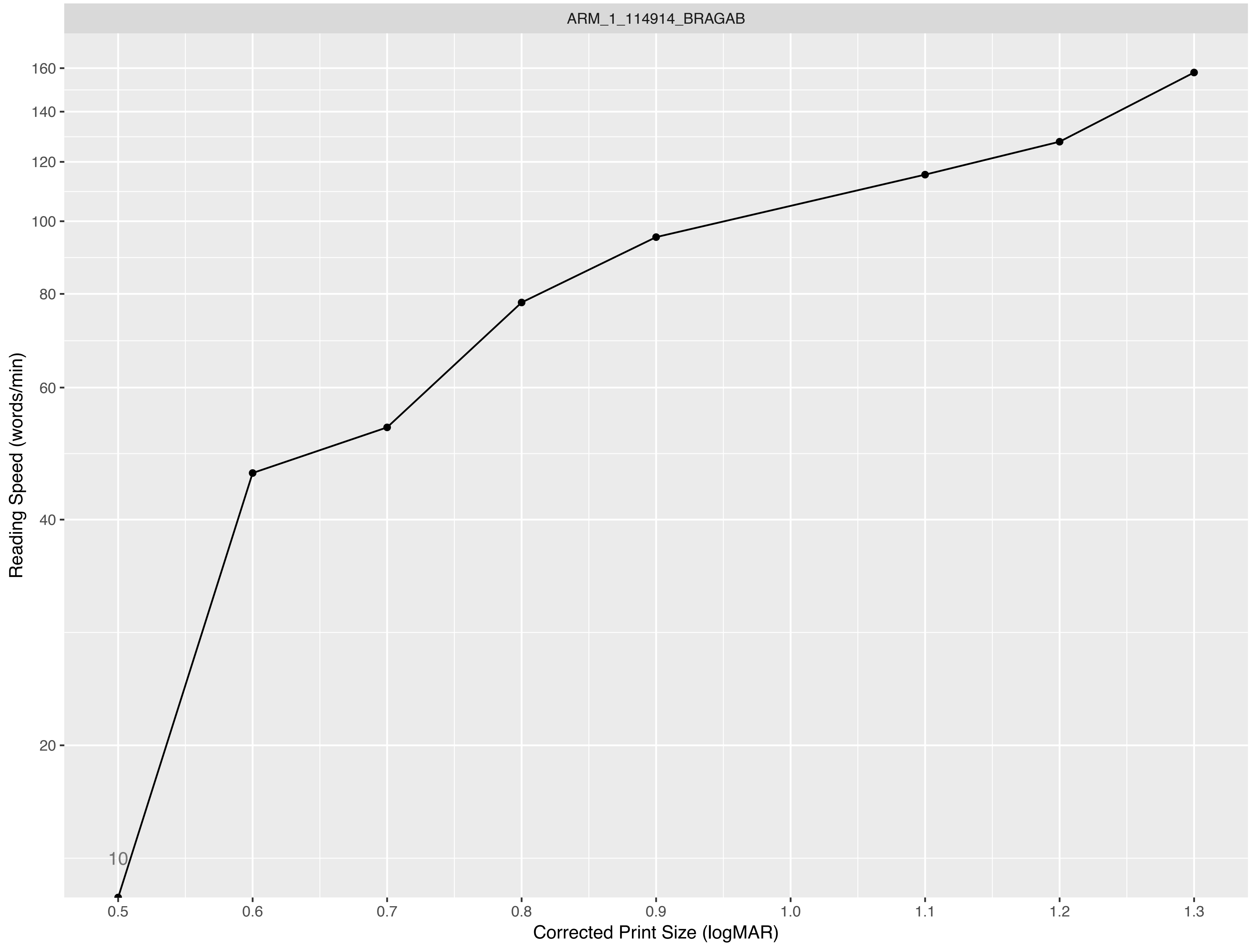

ARM_1_114914_BRAGAB
Reading Speed (words/min)
160
140
120
100
80
60
40
20
10
0.5
0.6
0.7
0.8
0.9
1.0
1.1
1.2
1.3
Corrected Print Size (logMAR)

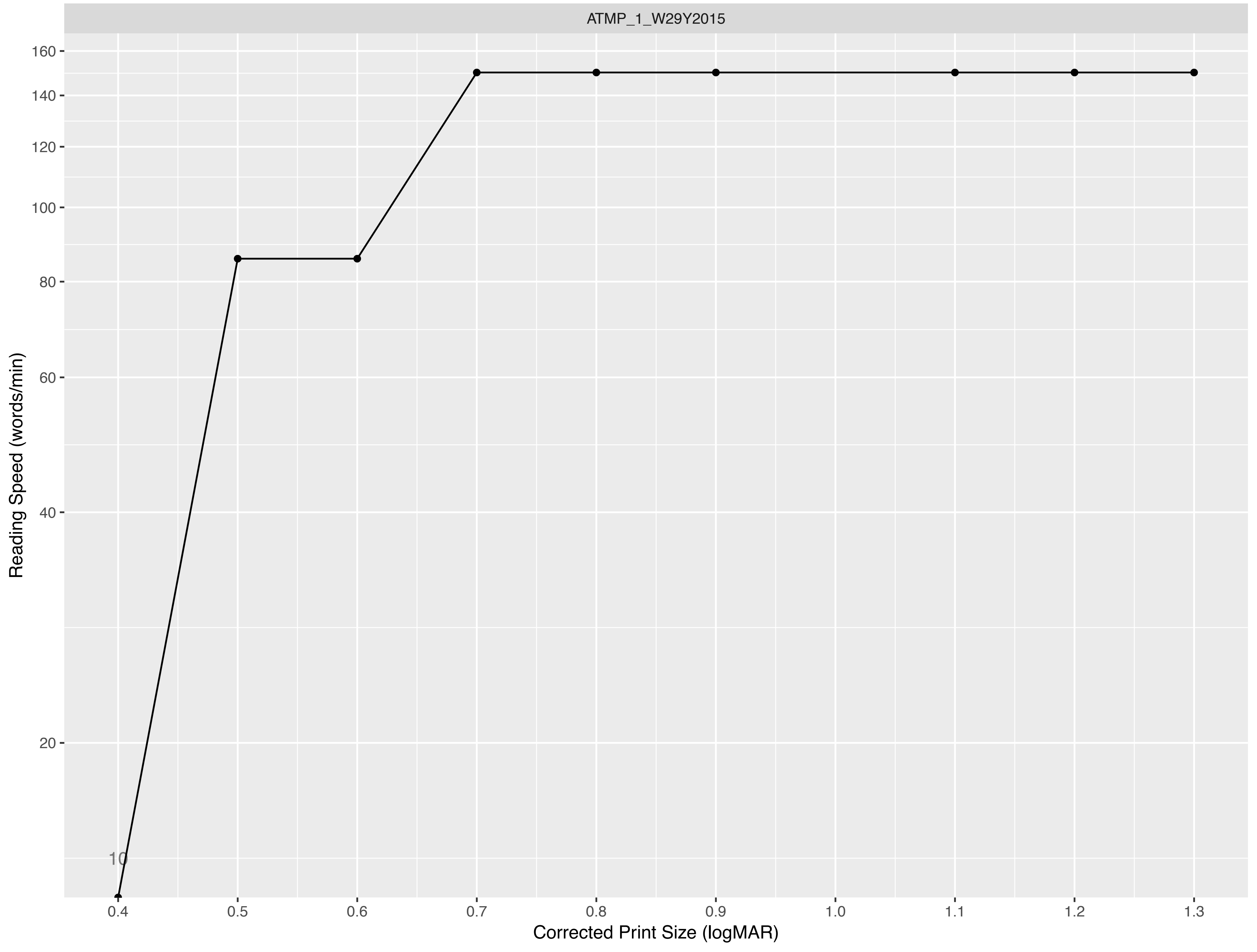
ATMP_1_W29Y2015
Reading Speed (words/min)
160
140
120
100
80
60
40
20
10
0.4
0.5
0.6
0.7
0.8
0.9
1.0
1.1
1.2
1.3
Corrected Print Size (logMAR)

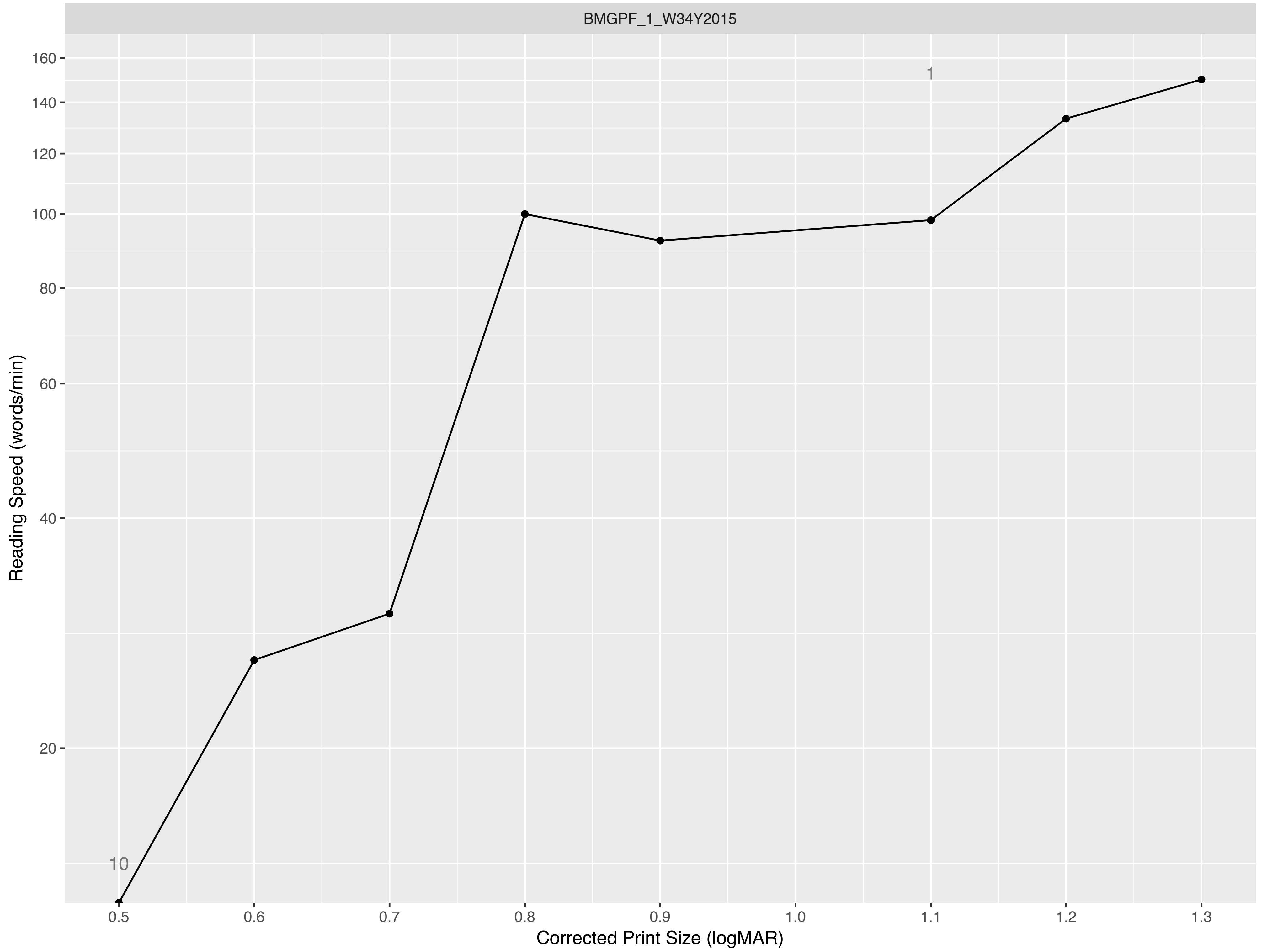

BMGPF_1_W34Y2015
Reading Speed (words/min)
160
140
120
100
80
60
40
20
1
10
0.5
0.6
0.7
0.8
0.9
1.0
1.1
1.2
1.3
Corrected Print Size (logMAR)

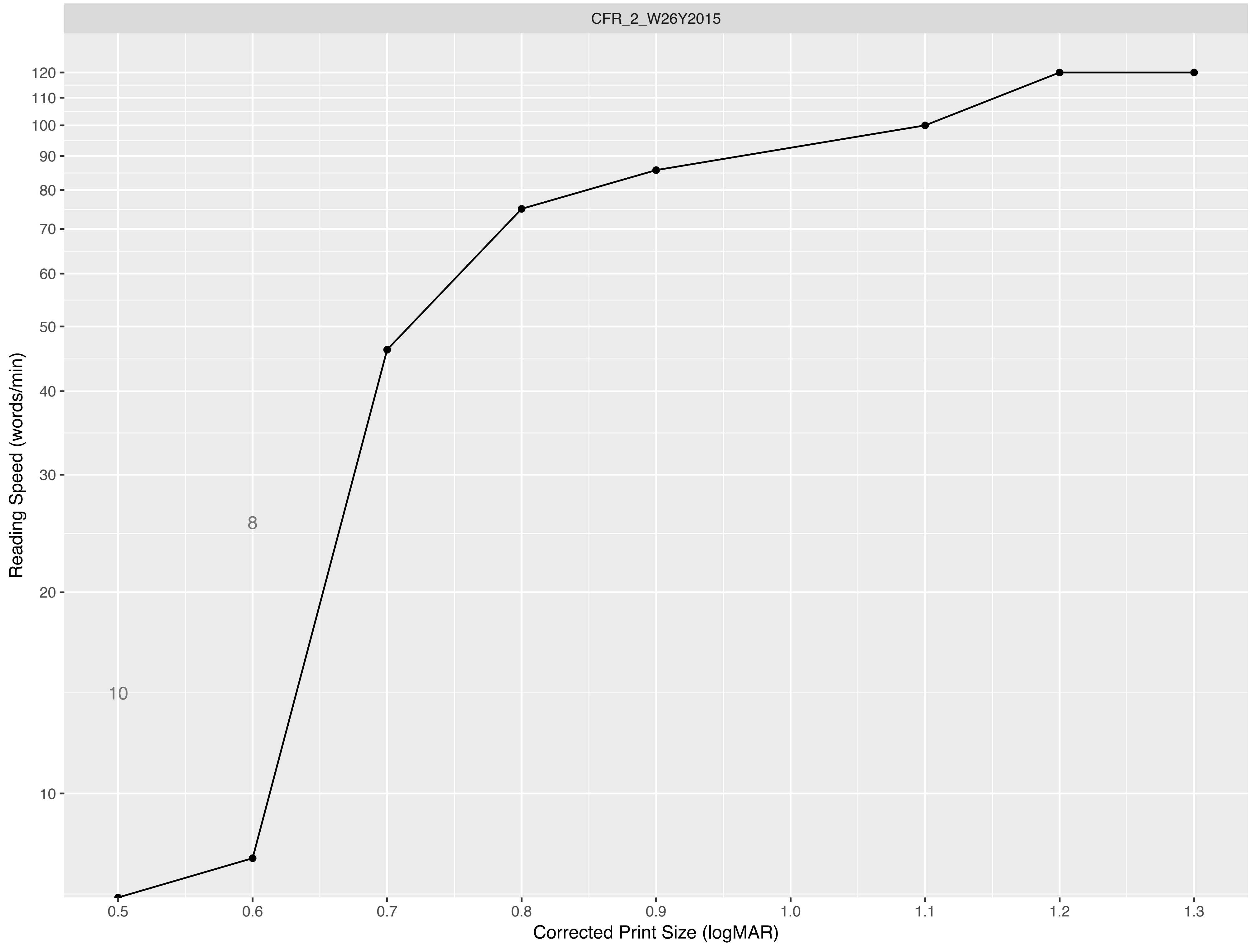

CFR_2_W26Y2015
Reading Speed (words/min)
120
110
100
90
80
70
60
50
40
30
20
10
10
8
0.5
0.6
0.7
0.8
0.9
1.0
1.1
1.2
1.3
Corrected Print Size (logMAR)

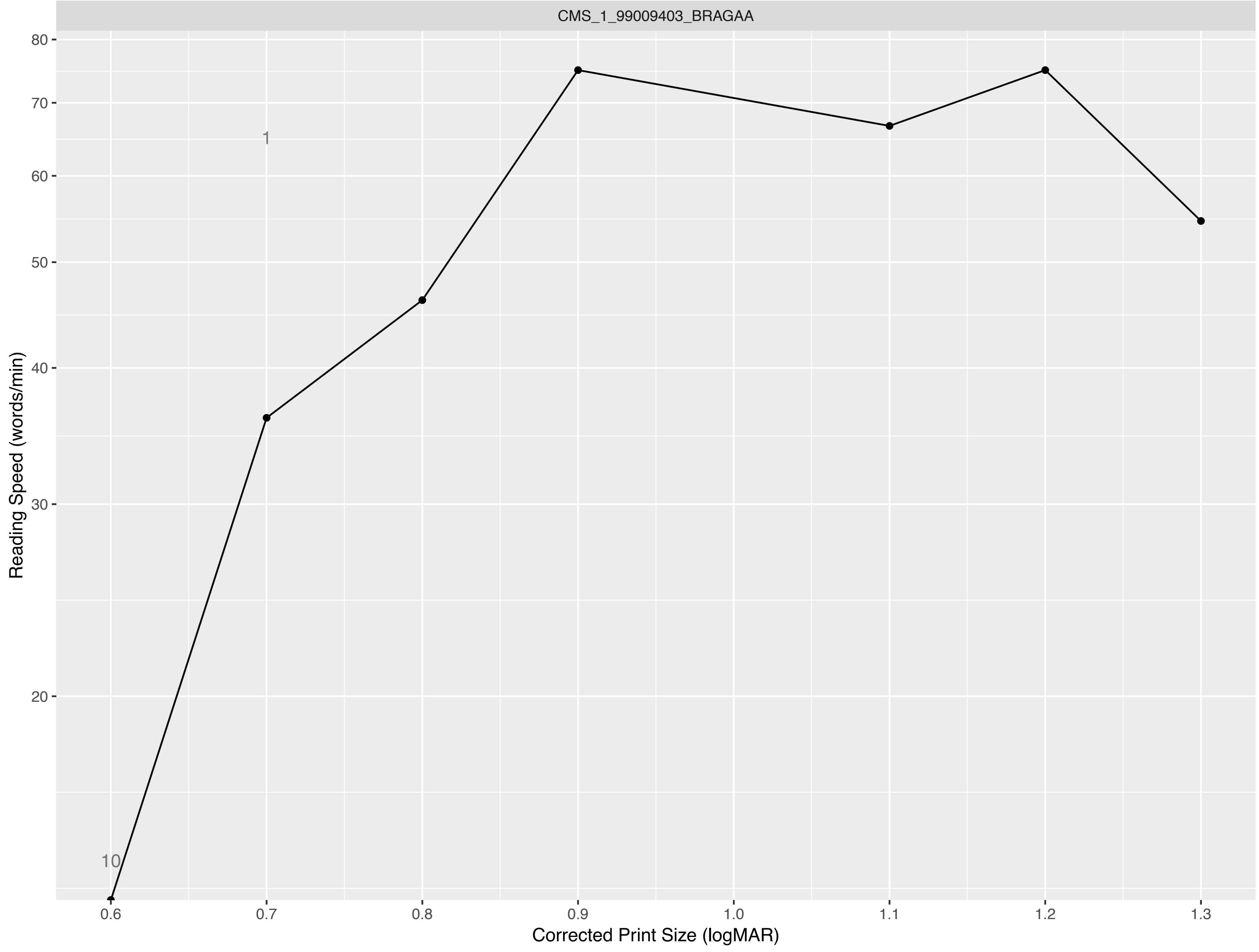

CMS_1_99009403_BRAGAA
Reading Speed (words/min)
80
70
60
50
40
30
20
1
10
0.6
0.7
0.8
0.9
1.0
1.1
1.2
1.3
Corrected Print Size (logMAR)

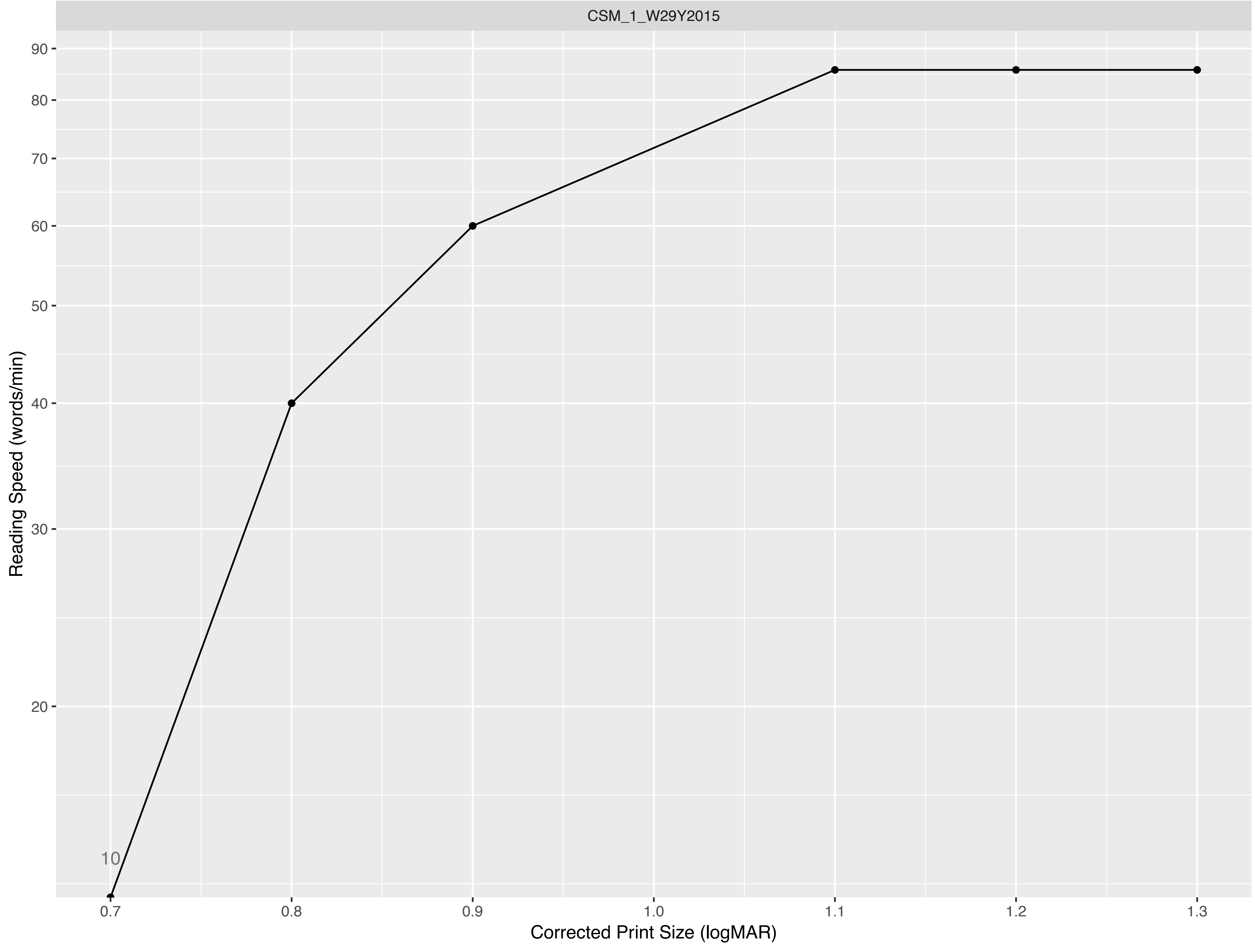
CSM_1_W29Y2015
Reading Speed (words/min)
Corrected Print Size (logMAR)
90
80
70
60
50
40
30
20
10
0.7
0.8
0.9
1.0
1.1
1.2
1.3

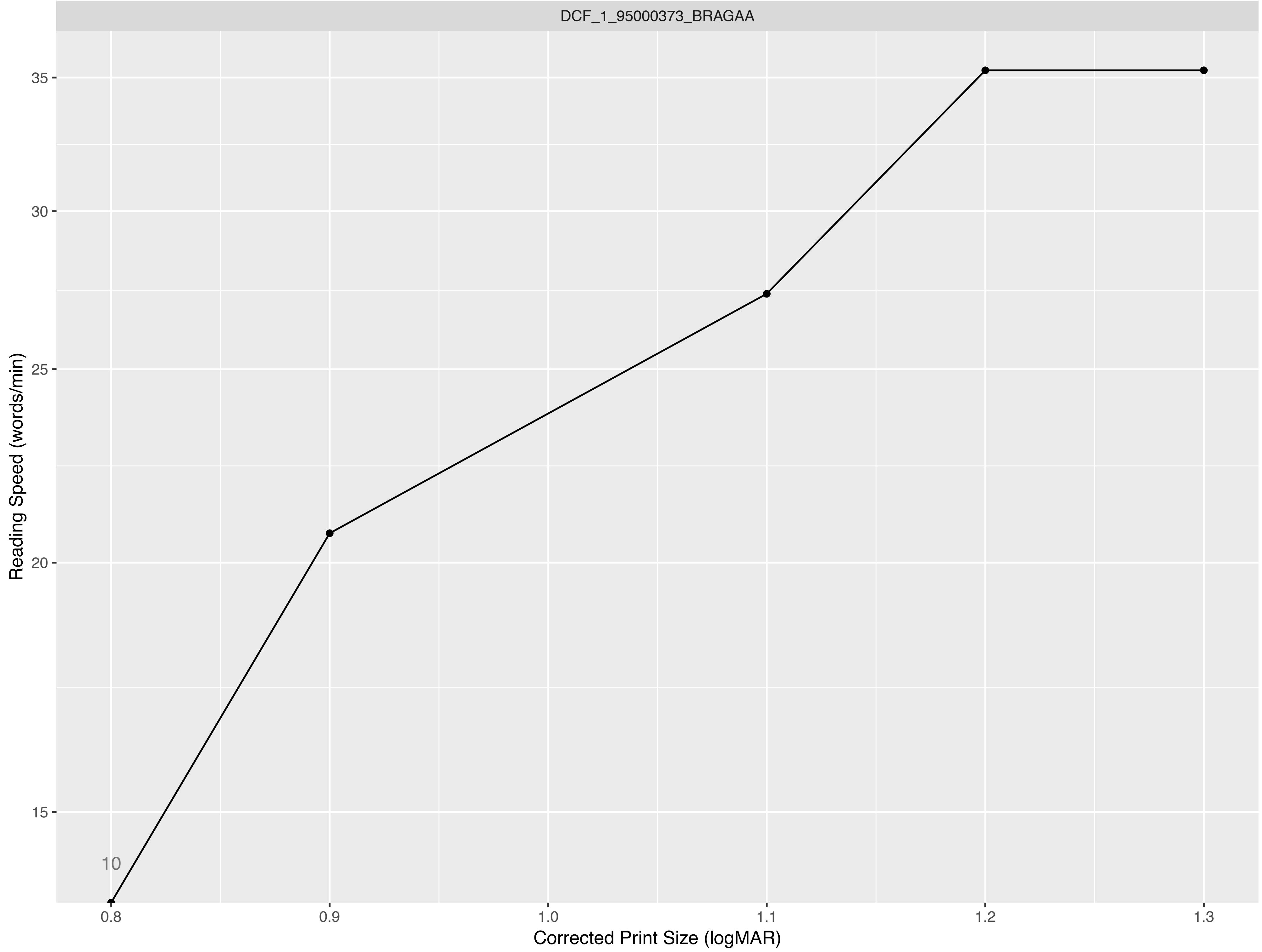
DCF_1_95000373_BRAGAA
Reading Speed (words/min)
35
30
25
20
15
10
0.8
0.9
1.0
1.1
1.2
1.3
Corrected Print Size (logMAR)

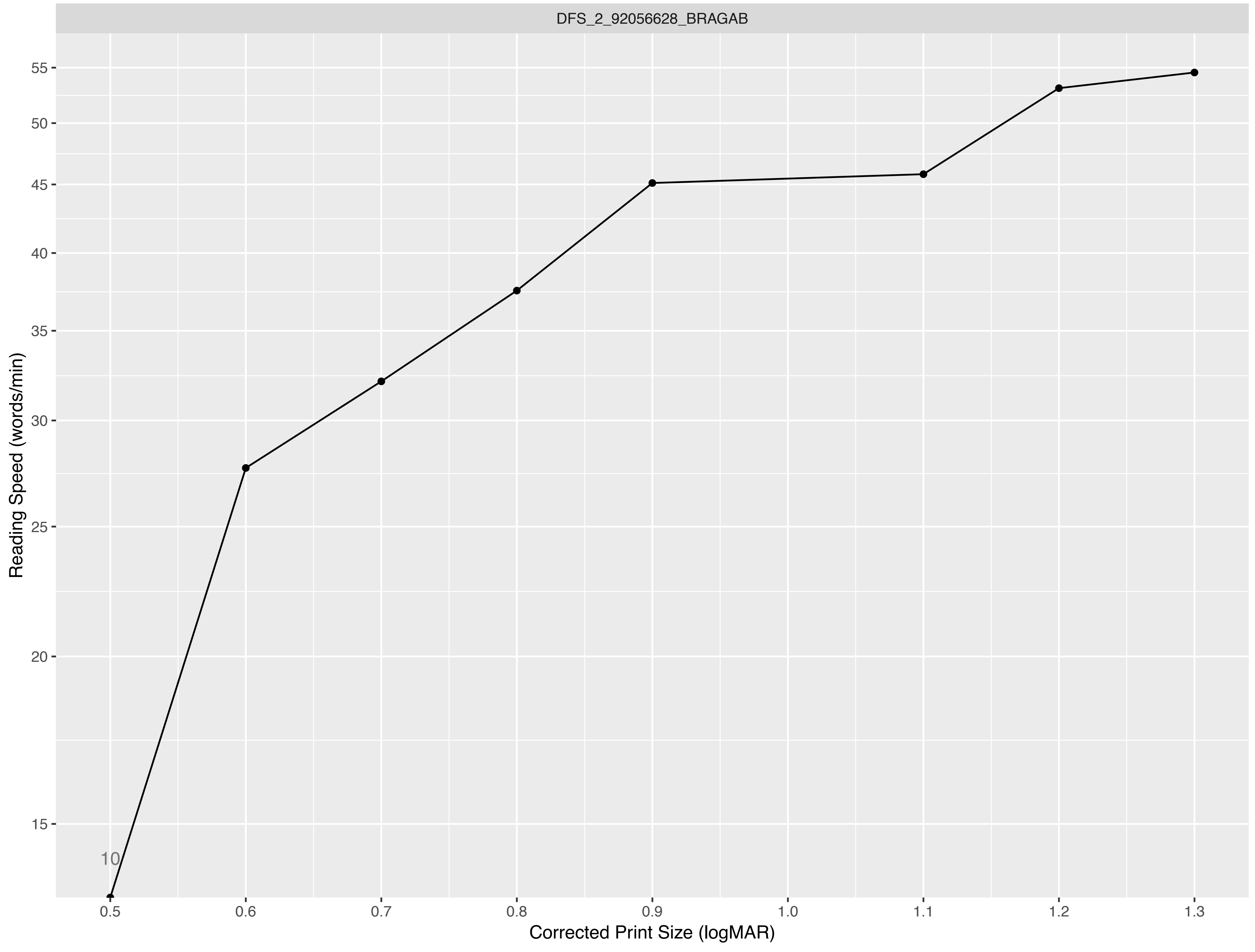
DFS_2_92056628_BRAGAB
Reading Speed (words/min)
55
50
45
40
35
30
25
20
15
10
0.5
0.6
0.7
0.8
0.9
1.0
1.1
1.2
1.3
Corrected Print Size (logMAR)

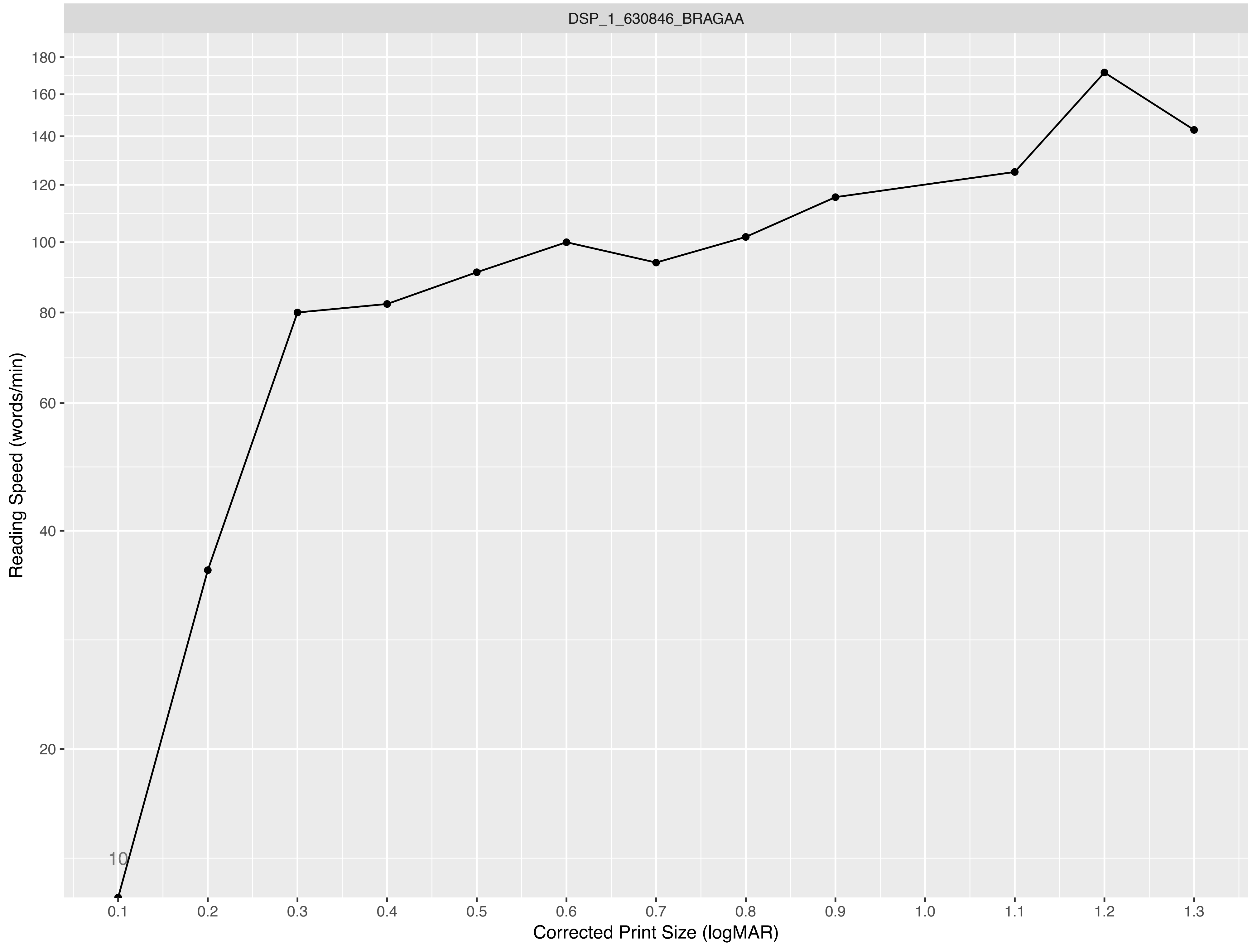
DSP_1_630846_BRAGAA
Reading Speed (words/min)
180
160
140
120
100
80
60
40
20
10
0.1
0.2
0.3
0.4
0.5
0.6
0.7
0.8
0.9
1.0
1.1
1.2
1.3
Corrected Print Size (logMAR)

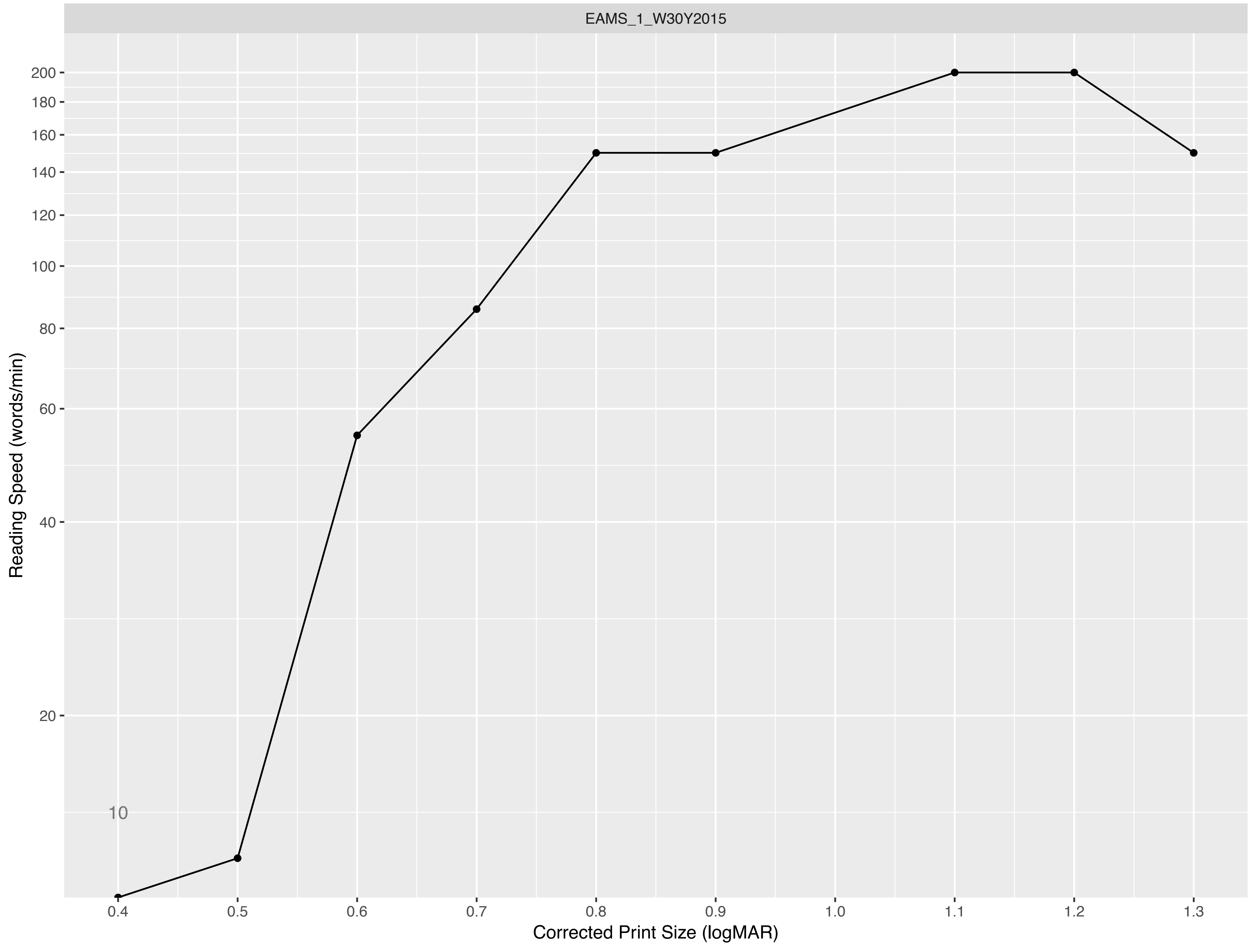
EAMS_1_W30Y2015
Reading Speed (words/min)
200
180
160
140
120
100
80
60
40
20
10
0.4
0.5
0.6
0.7
0.8
0.9
1.0
1.1
1.2
1.3
Corrected Print Size (logMAR)

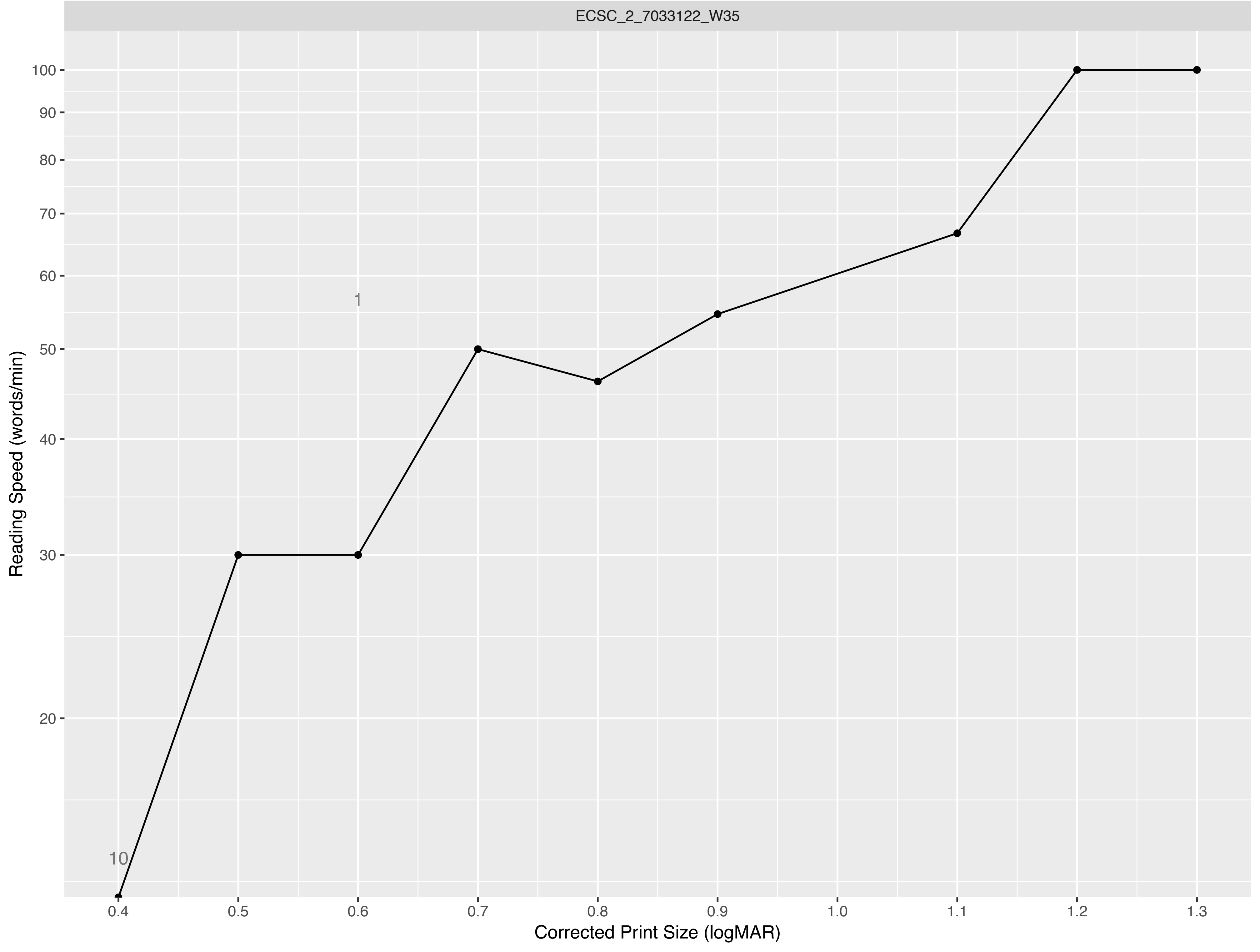
ECSC_2_7033122_W35
Reading Speed (words/min)
100
90
80
70
60
50
40
30
20
1
10
0.4
0.5
0.6
0.7
0.8
0.9
1.0
1.1
1.2
1.3
Corrected Print Size (logMAR)

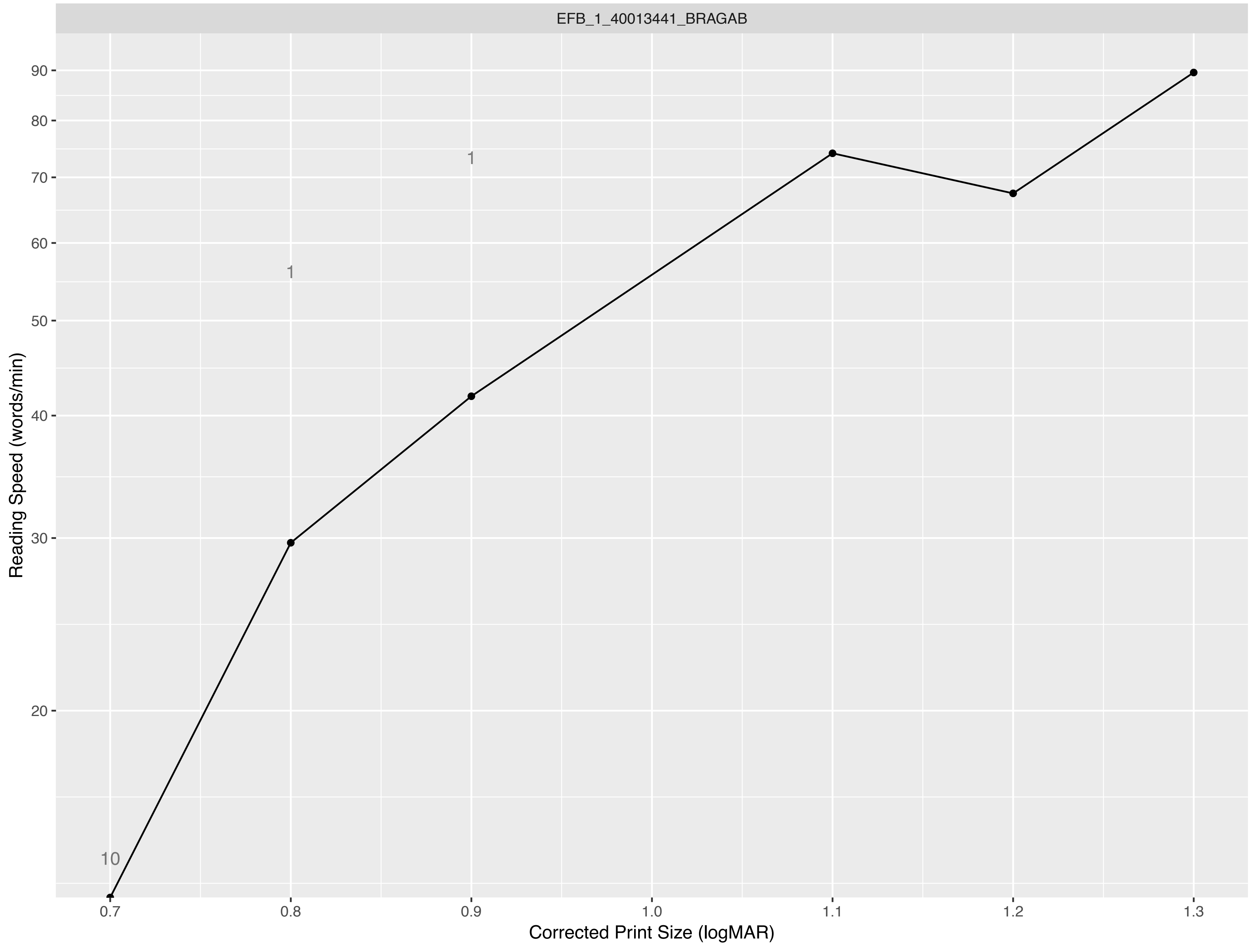
EFB_1_40013441_BRAGAB
Reading Speed (words/min)
90
80
70
60
50
40
30
20
1
1
10
0.7
0.8
0.9
1.0
1.1
1.2
1.3
Corrected Print Size (logMAR)

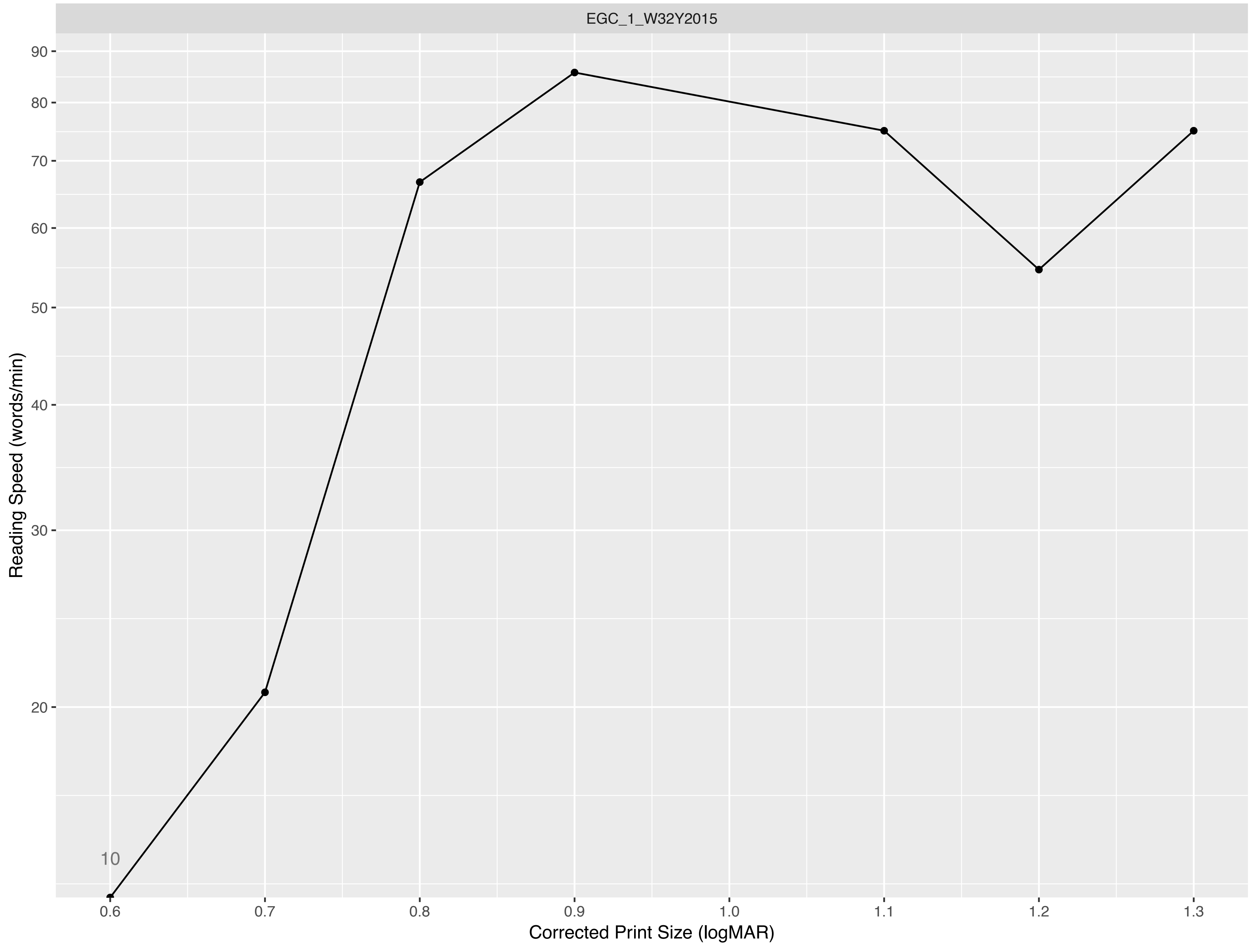
EGC_1_W32Y2015
Reading Speed (words/min)
90
80
70
60
50
40
30
20
10
0.6
0.7
0.8
0.9
1.0
1.1
1.2
1.3
Corrected Print Size (logMAR)

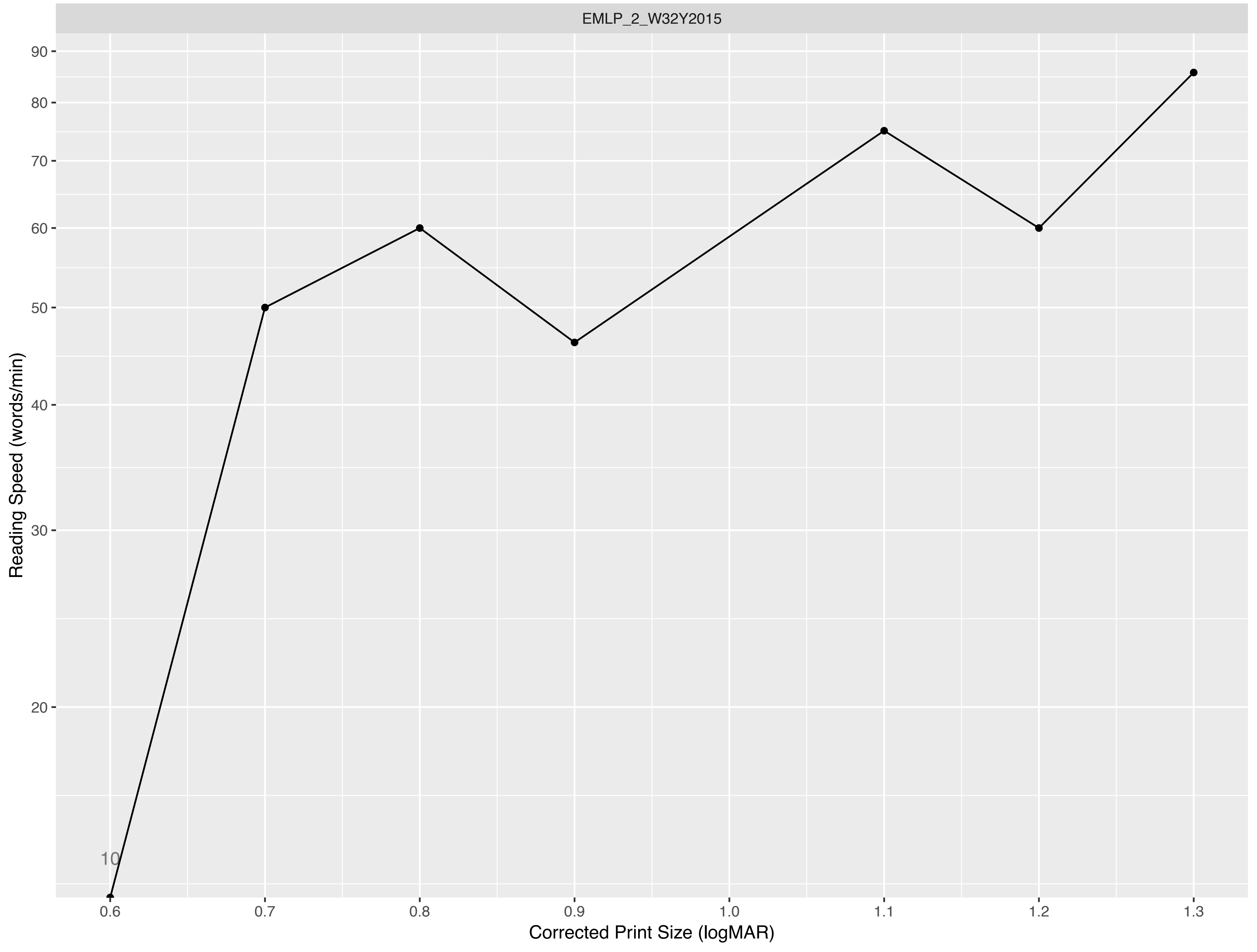
EMLP_2_W32Y2015
Reading Speed (words/min)
90
80
70
60
50
40
30
20
10
0.6
0.7
0.8
0.9
1.0
1.1
1.2
1.3
Corrected Print Size (logMAR)

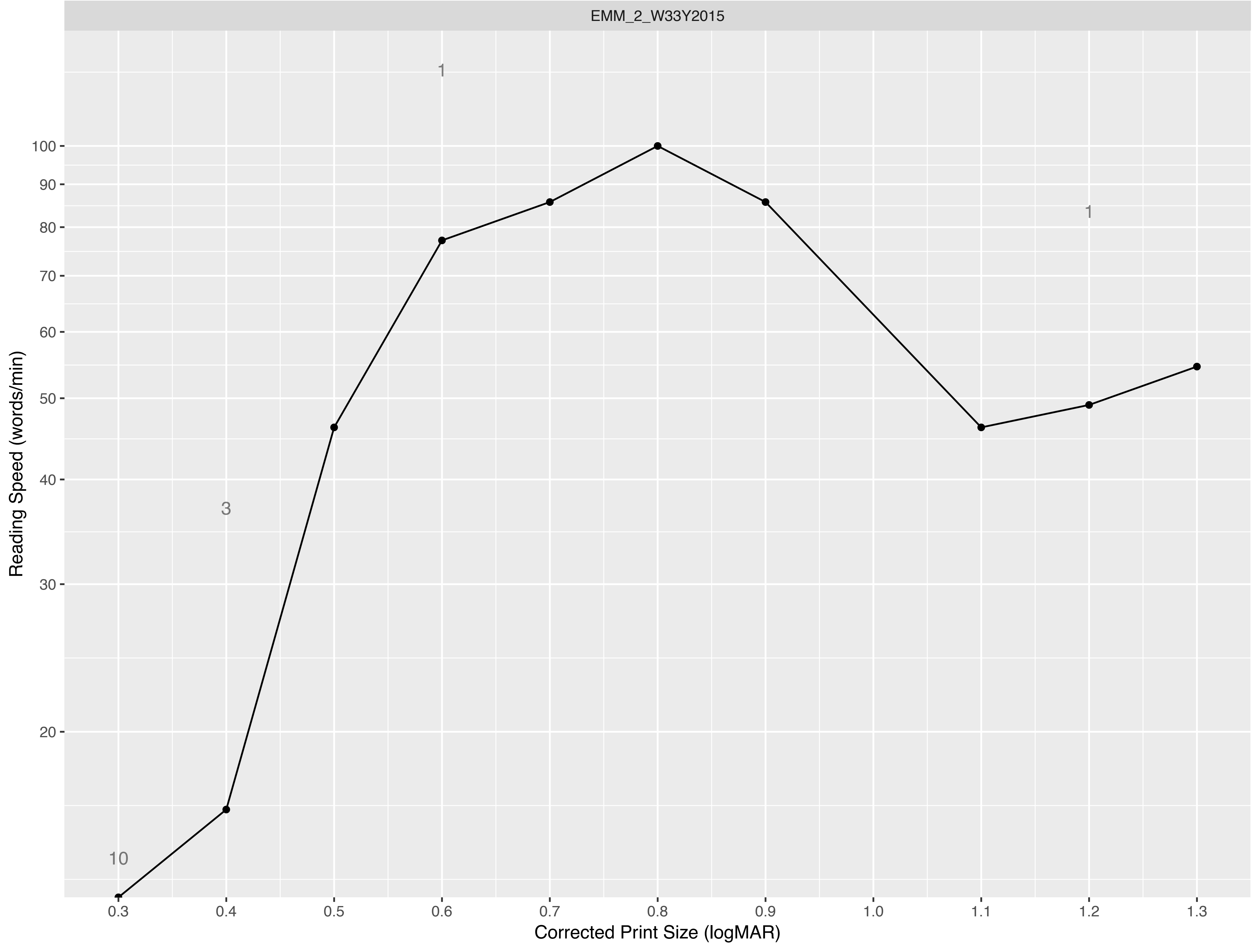

EMM_2_W33Y2015
Reading Speed (words/min)
100
90
80
70
60
50
40
30
20
1
1
3
10
0.3
0.4
0.5
0.6
0.7
0.8
0.9
1.0
1.1
1.2
1.3
Corrected Print Size (logMAR)

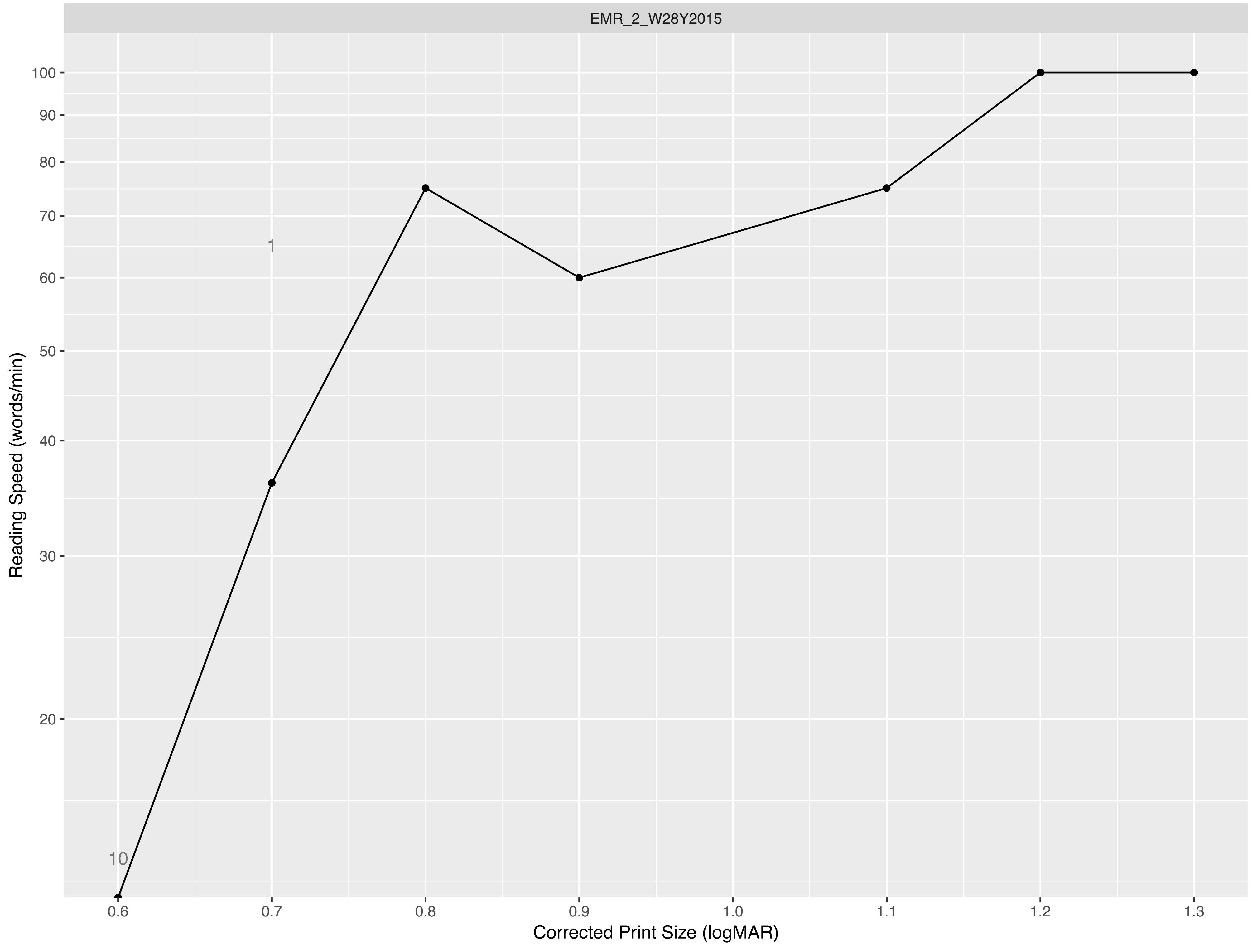
EMR_2_W28Y2015
Reading Speed (words/min)
100
90
80
70
60
50
40
30
20
1
10
0.6
0.7
0.8
0.9
1.0
1.1
1.2
1.3
Corrected Print Size (logMAR)

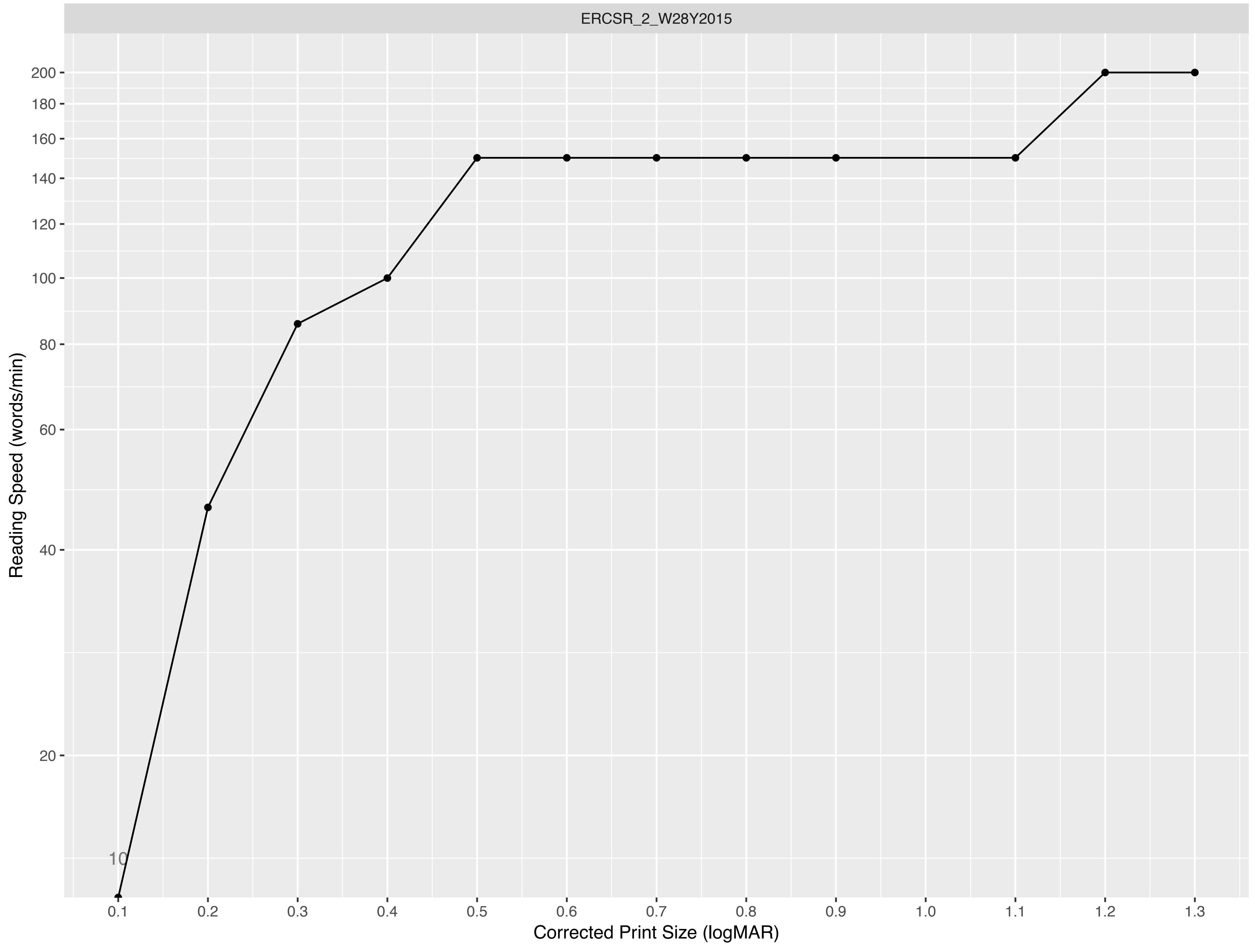

ERCSR_2_W28Y2015
Reading Speed (words/min)
200
180
160
140
120
100
80
60
40
20
10
0.1
0.2
0.3
0.4
0.5
0.6
0.7
0.8
0.9
1.0
1.1
1.2
1.3
Corrected Print Size (logMAR)

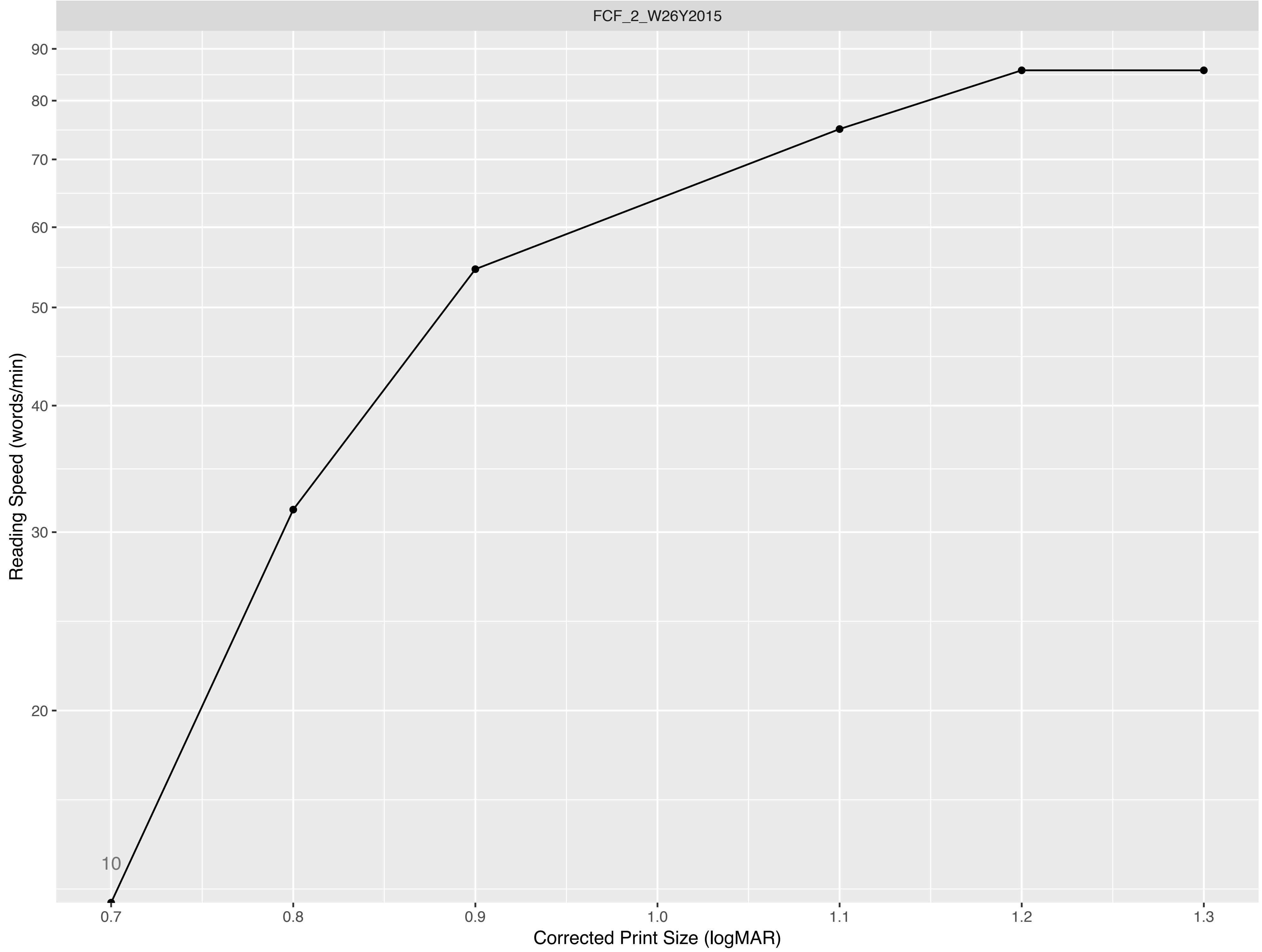
FCF_2_W26Y2015
Reading Speed (words/min)
90
80
70
60
50
40
30
20
10
0.7
0.8
0.9
1.0
1.1
1.2
1.3
Corrected Print Size (logMAR)

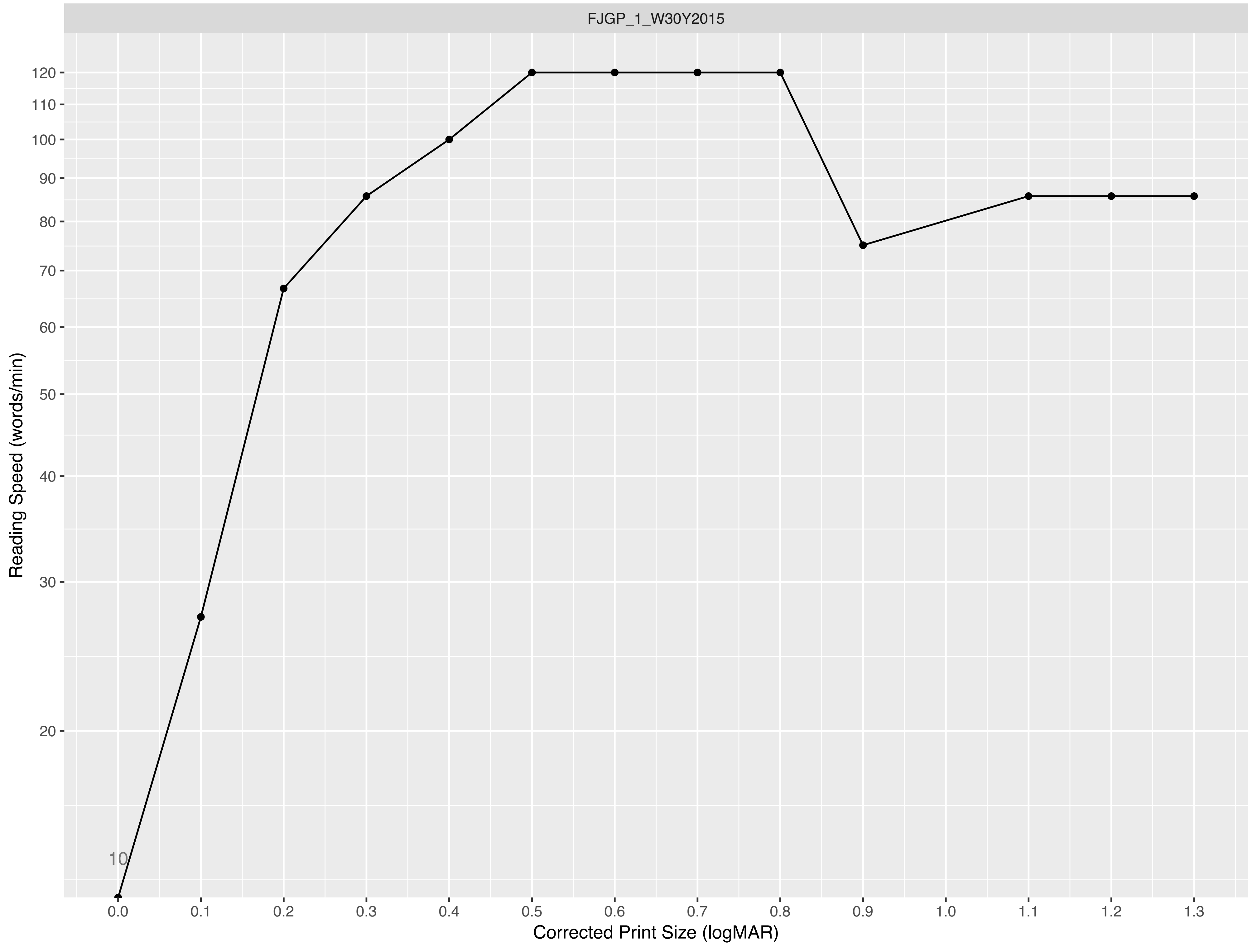

FJGP_1_W30Y2015
Reading Speed (words/min)
120
110
100
90
80
70
60
50
40
30
20
10
0.0
0.1
0.2
0.3
0.4
0.5
0.6
0.7
0.8
0.9
1.0
1.1
1.2
1.3
Corrected Print Size (logMAR)

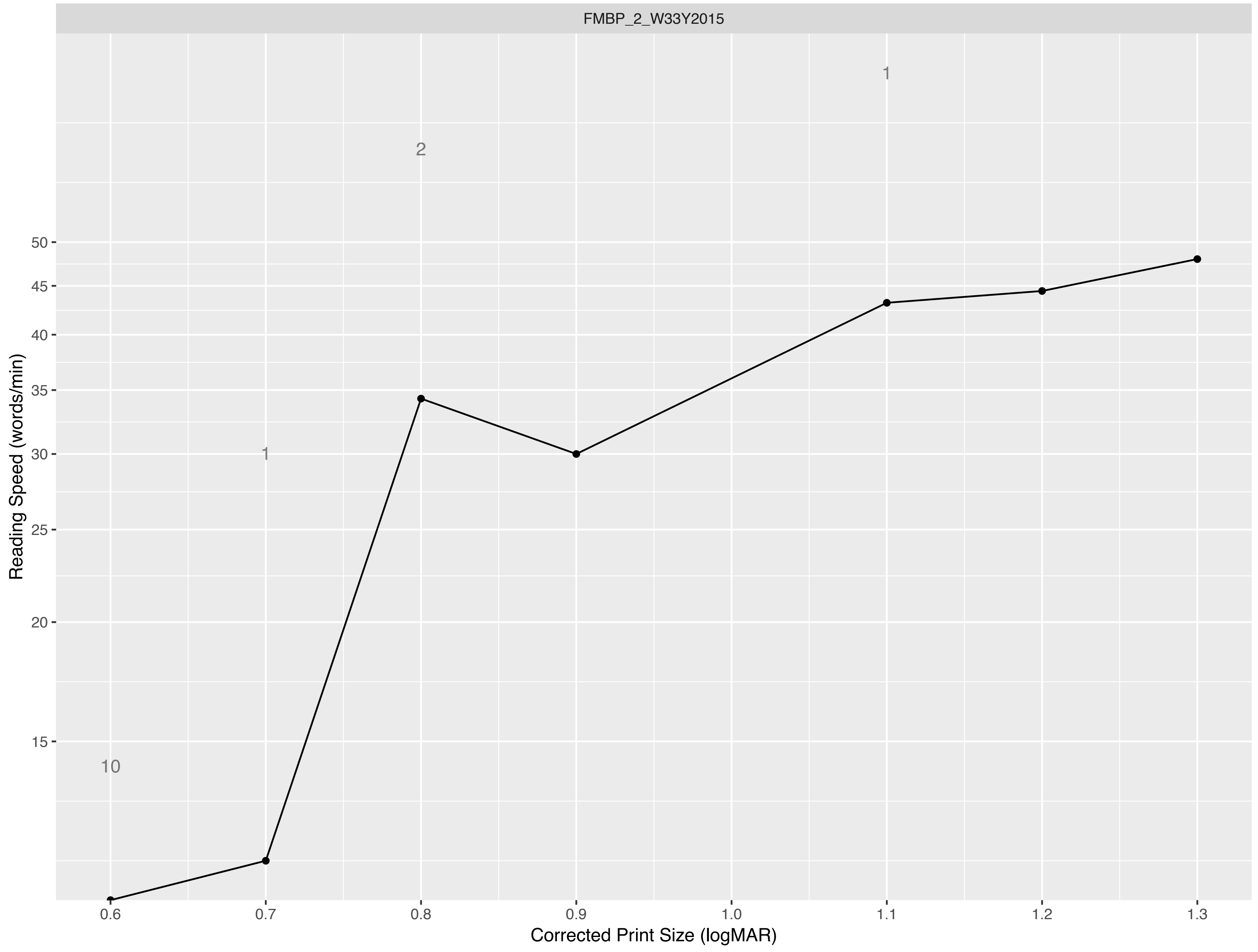

FMBP_2_W33Y2015
Reading Speed (words/min)
50
45
40
35
30
25
20
15
1
2
1
10
0.6
0.7
0.8
0.9
1.0
1.1
1.2
1.3
Corrected Print Size (logMAR)

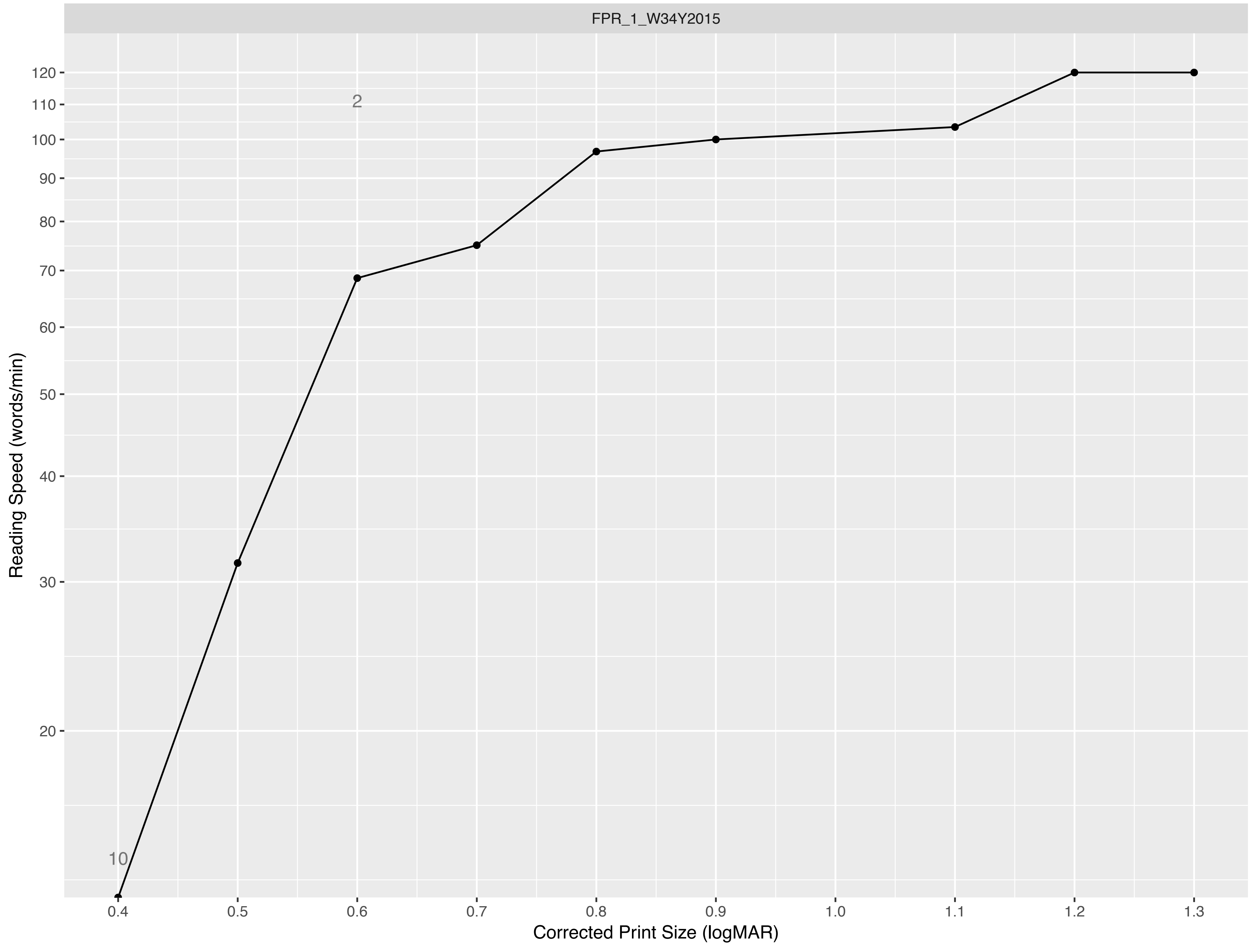
FPR_1_W34Y2015
Reading Speed (words/min)
120
110
100
90
80
70
60
50
40
30
20
2
10
0.4
0.5
0.6
0.7
0.8
0.9
1.0
1.1
1.2
1.3
Corrected Print Size (logMAR)

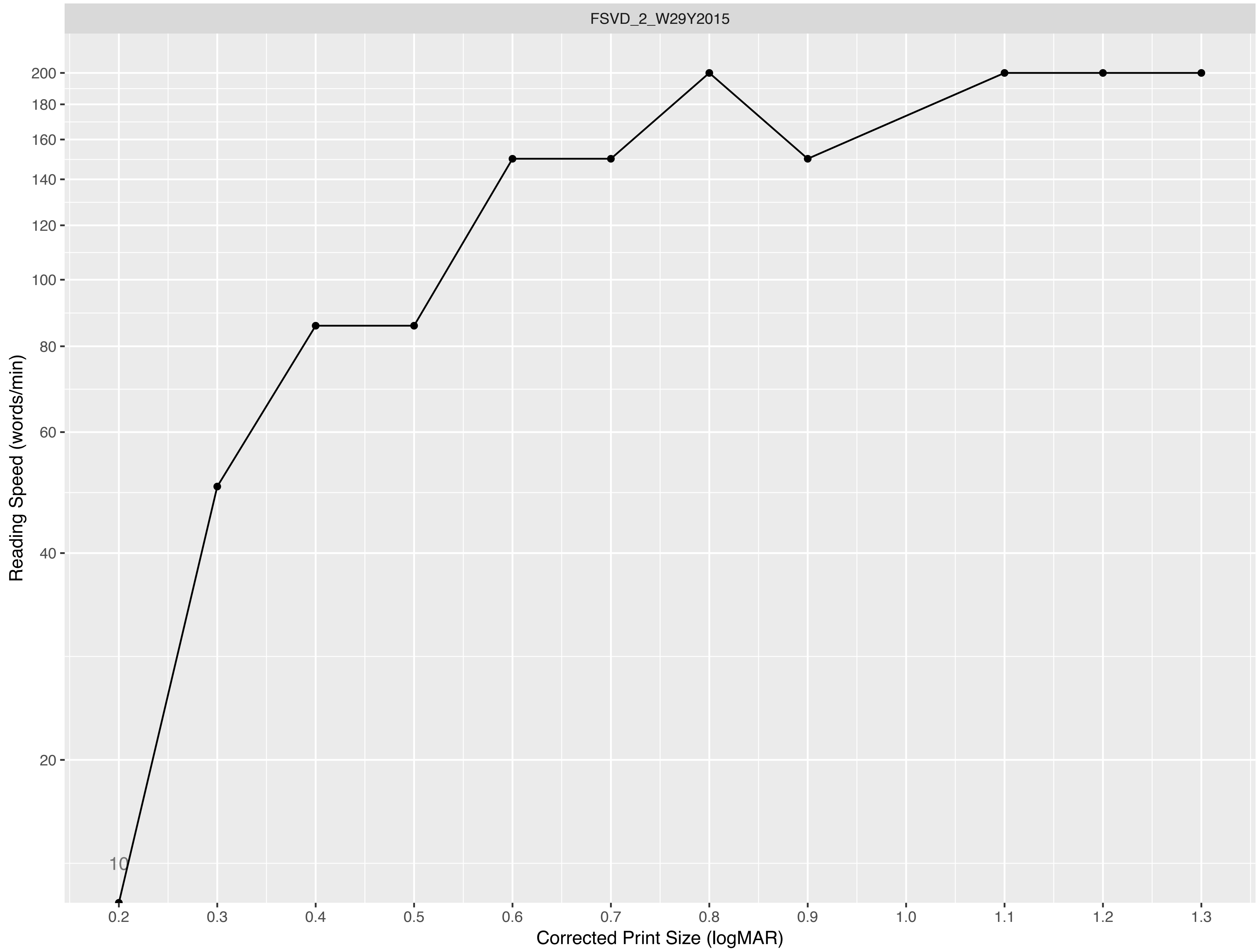

FSVD_2_W29Y2015
Reading Speed (words/min)
200
180
160
140
120
100
80
60
40
20
10
Corrected Print Size (logMAR)
0.2
0.3
0.4
0.5
0.6
0.7
0.8
0.9
1.0
1.1
1.2
1.3

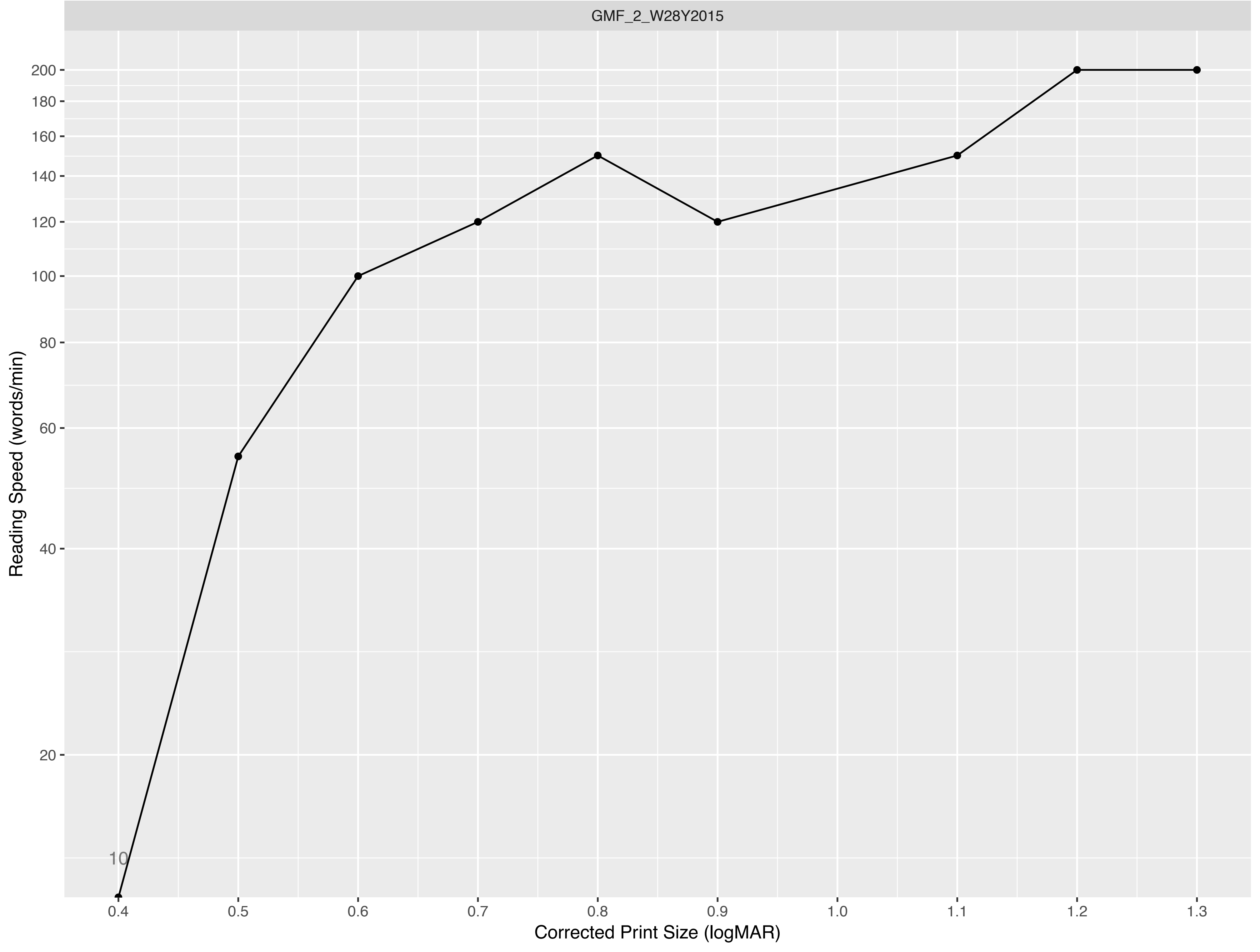
GMF_2_W28Y2015
Reading Speed (words/min)
200
180
160
140
120
100
80
60
40
20
10
0.4
0.5
0.6
0.7
0.8
0.9
1.0
1.1
1.2
1.3
Corrected Print Size (logMAR)

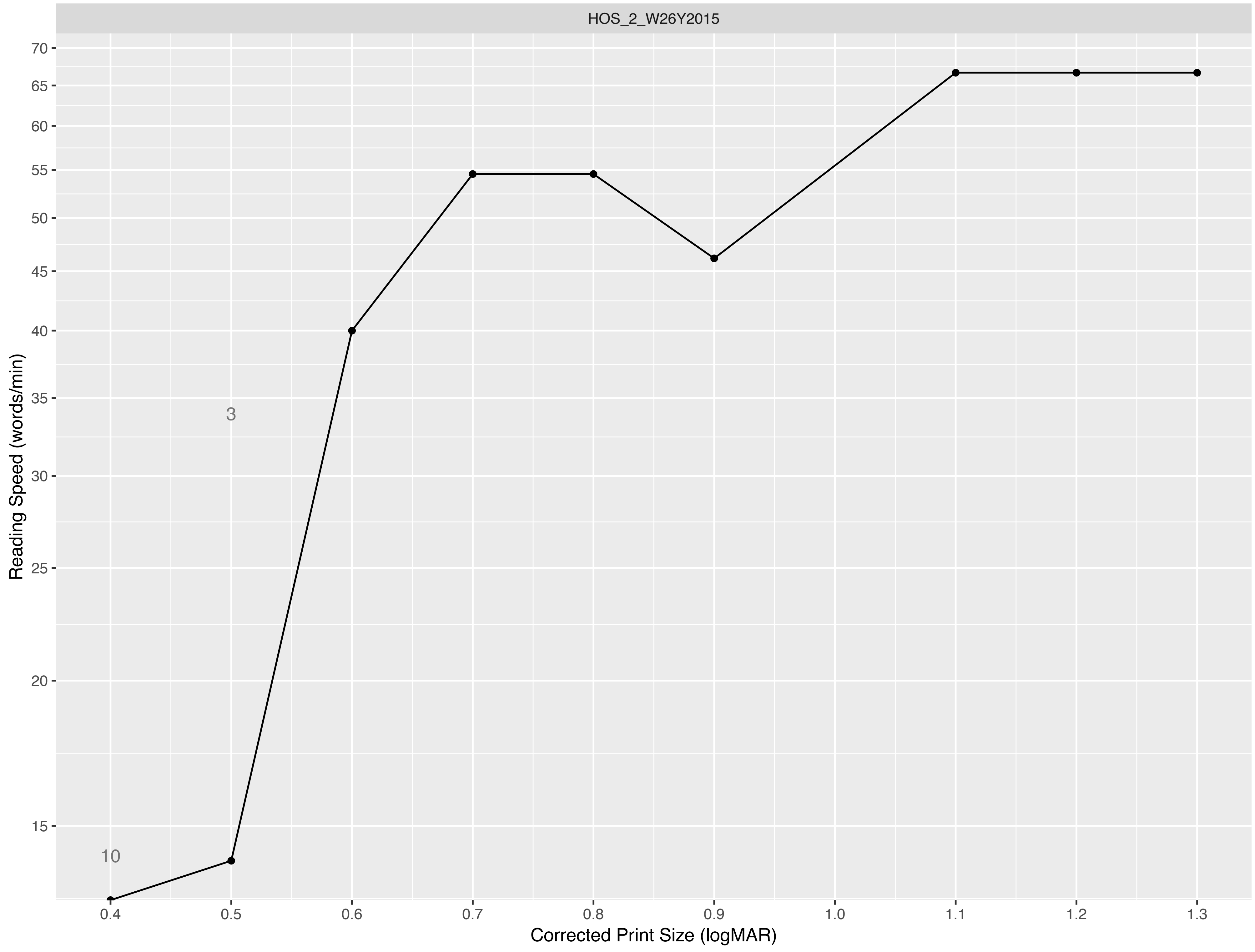

HOS_2_W26Y2015
Reading Speed (words/min)
70
65
60
55
50
45
40
35
30
25
20
15
3
10
0.4
0.5
0.6
0.7
0.8
0.9
1.0
1.1
1.2
1.3
Corrected Print Size (logMAR)

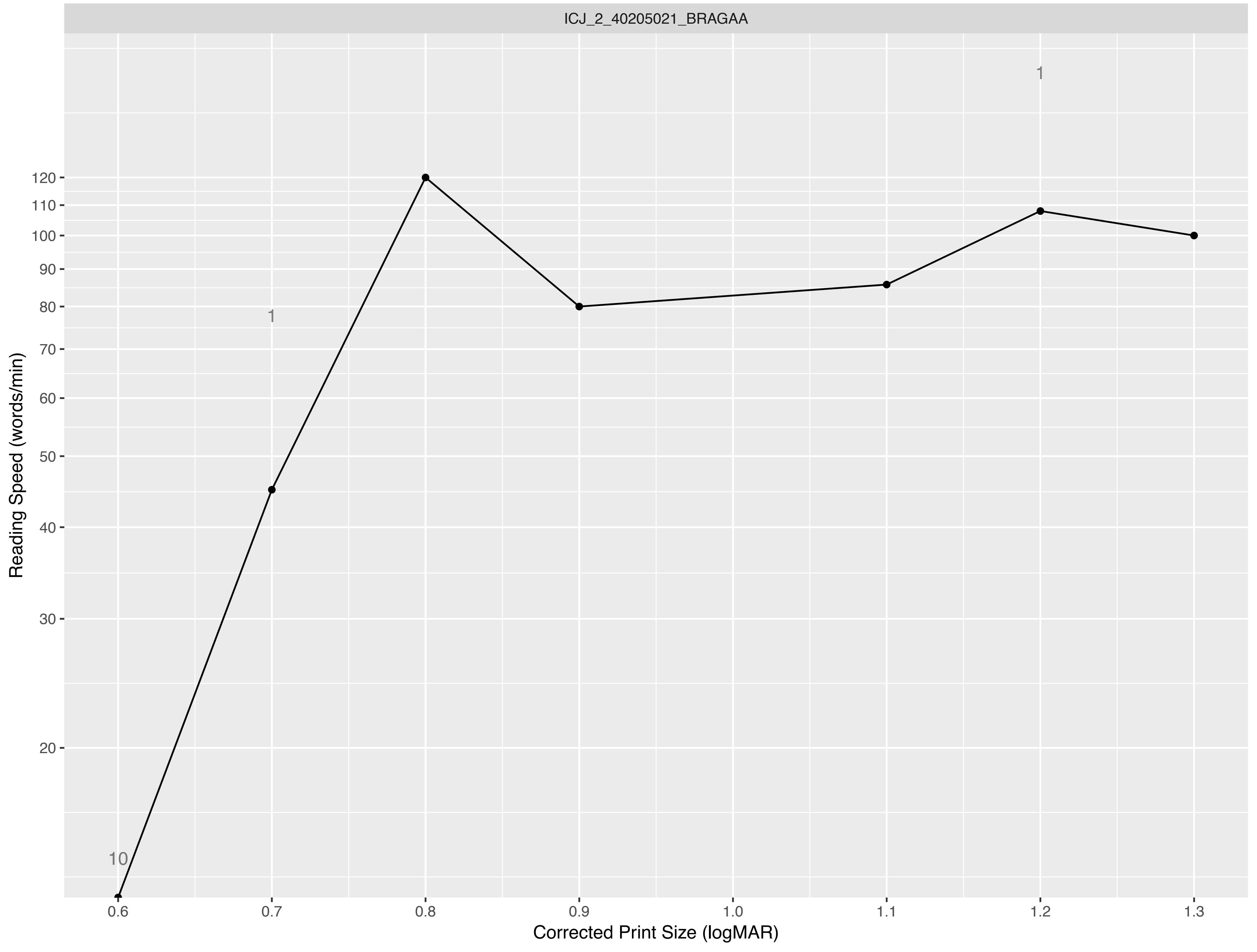

ICJ_2_40205021_BRAGAA
Reading Speed (words/min)
Corrected Print Size (logMAR)
120
110
100
90
80
70
60
50
40
30
20
0.6
0.7
0.8
0.9
1.0
1.1
1.2
1.3
10
1
1

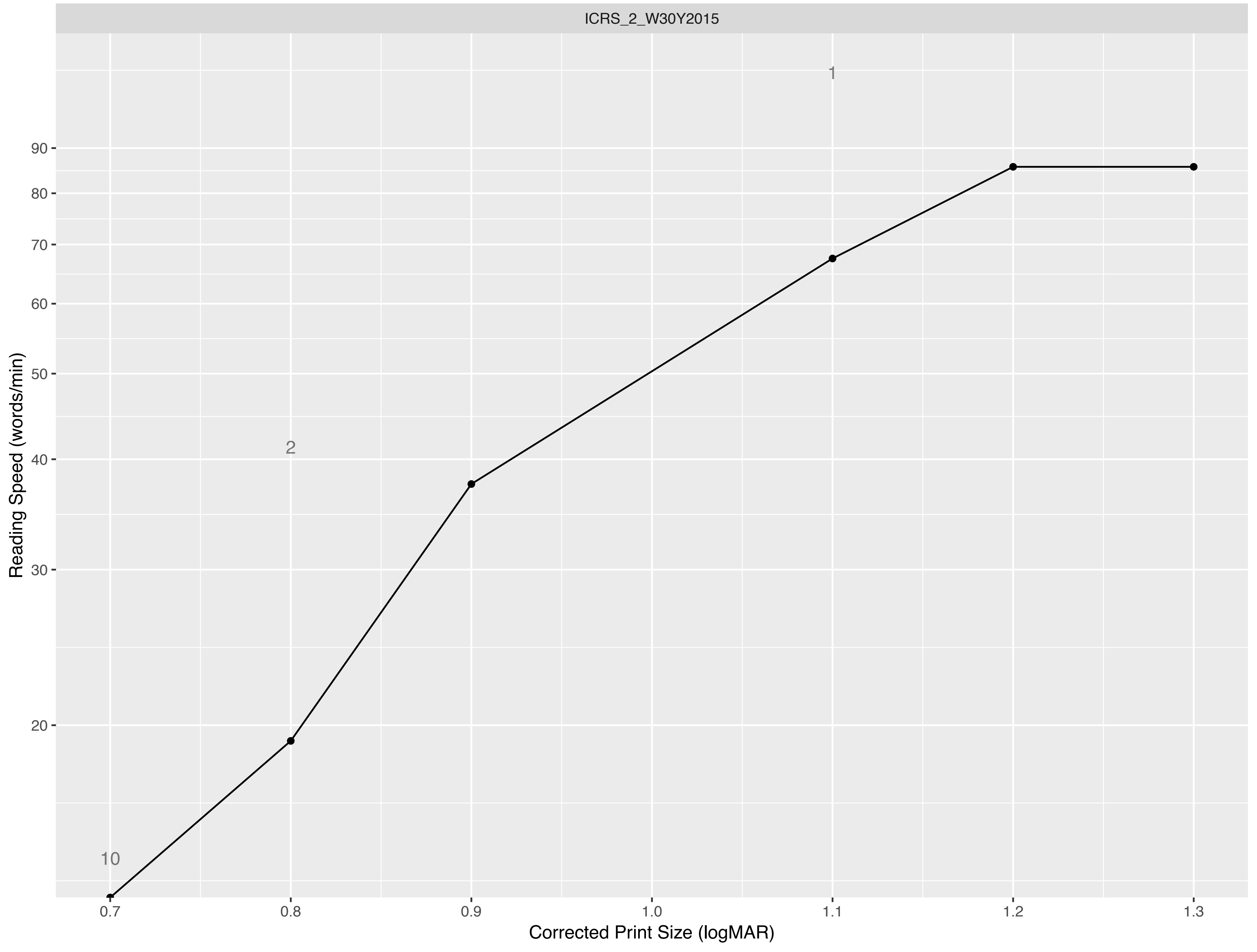

ICRS_2_W30Y2015
Reading Speed (words/min)
90
80
70
60
50
40
30
20
1
2
10
0.7
0.8
0.9
1.0
1.1
1.2
1.3
Corrected Print Size (logMAR)

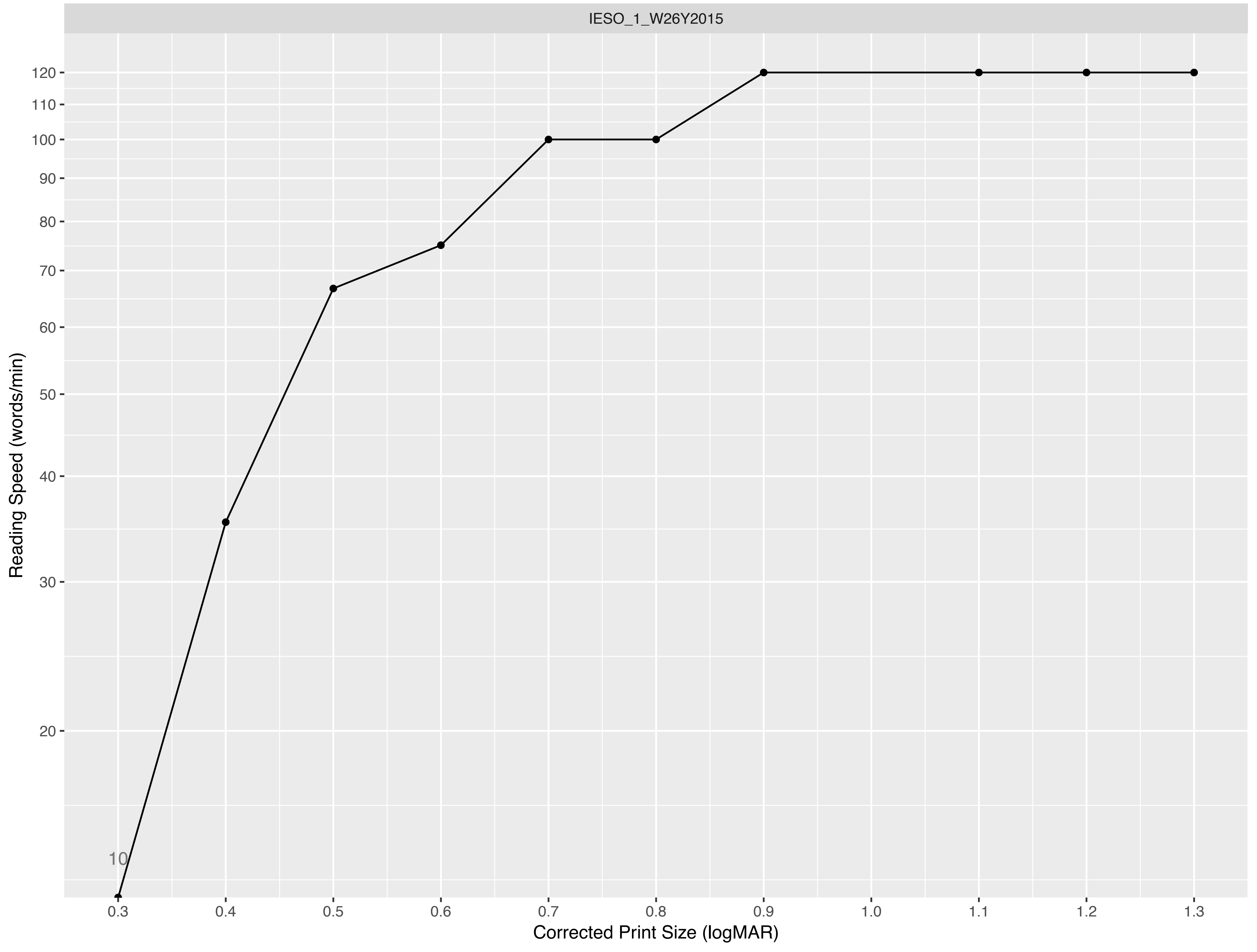

IESO_1_W26Y2015
Reading Speed (words/min)
120
110
100
90
80
70
60
50
40
30
20
10
0.3
0.4
0.5
0.6
0.7
0.8
0.9
1.0
1.1
1.2
1.3
Corrected Print Size (logMAR)

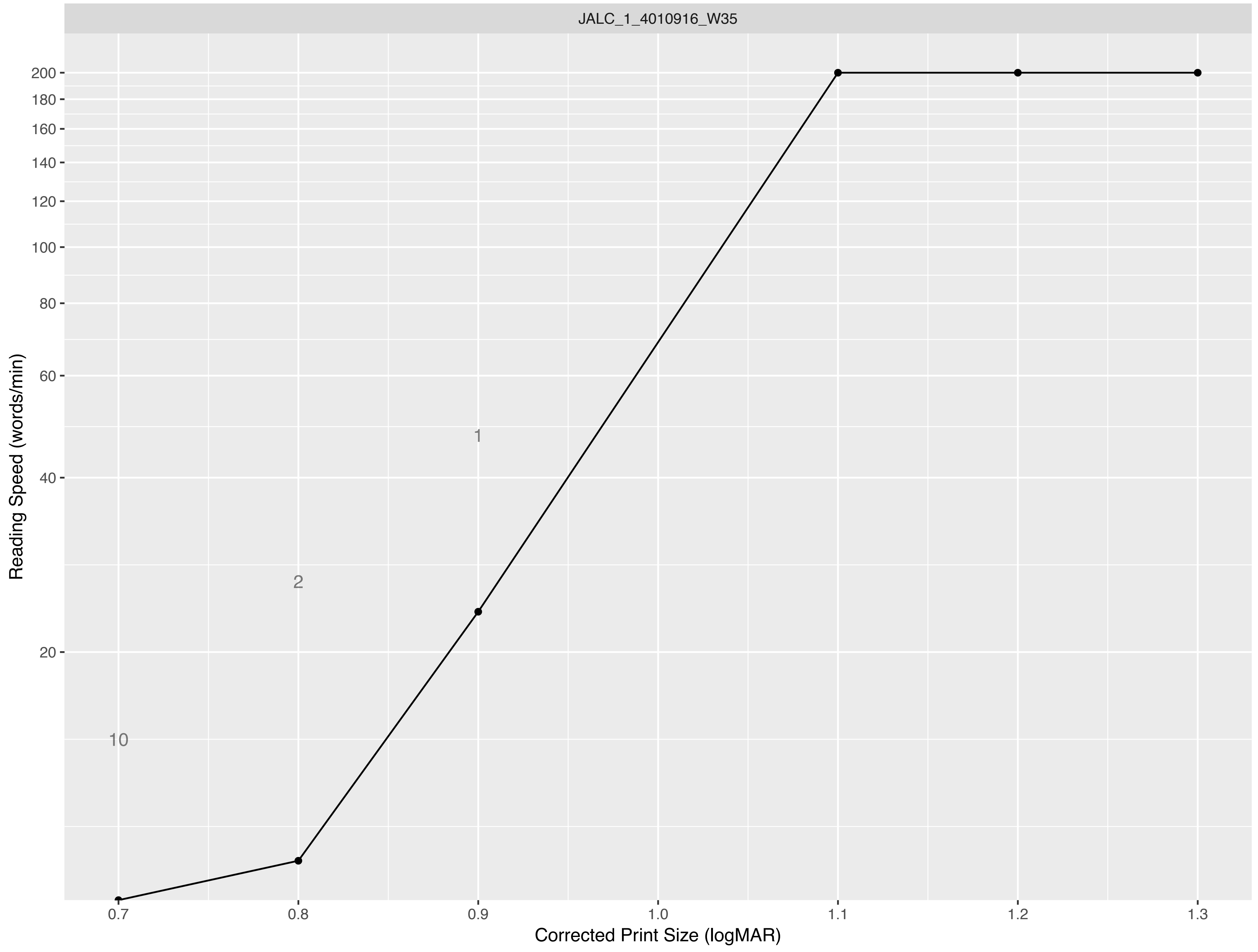
JALC_1_4010916_W35
Reading Speed (words/min)
200
180
160
140
120
100
80
60
40
20
1
2
10
0.7
0.8
0.9
1.0
1.1
1.2
1.3
Corrected Print Size (logMAR)

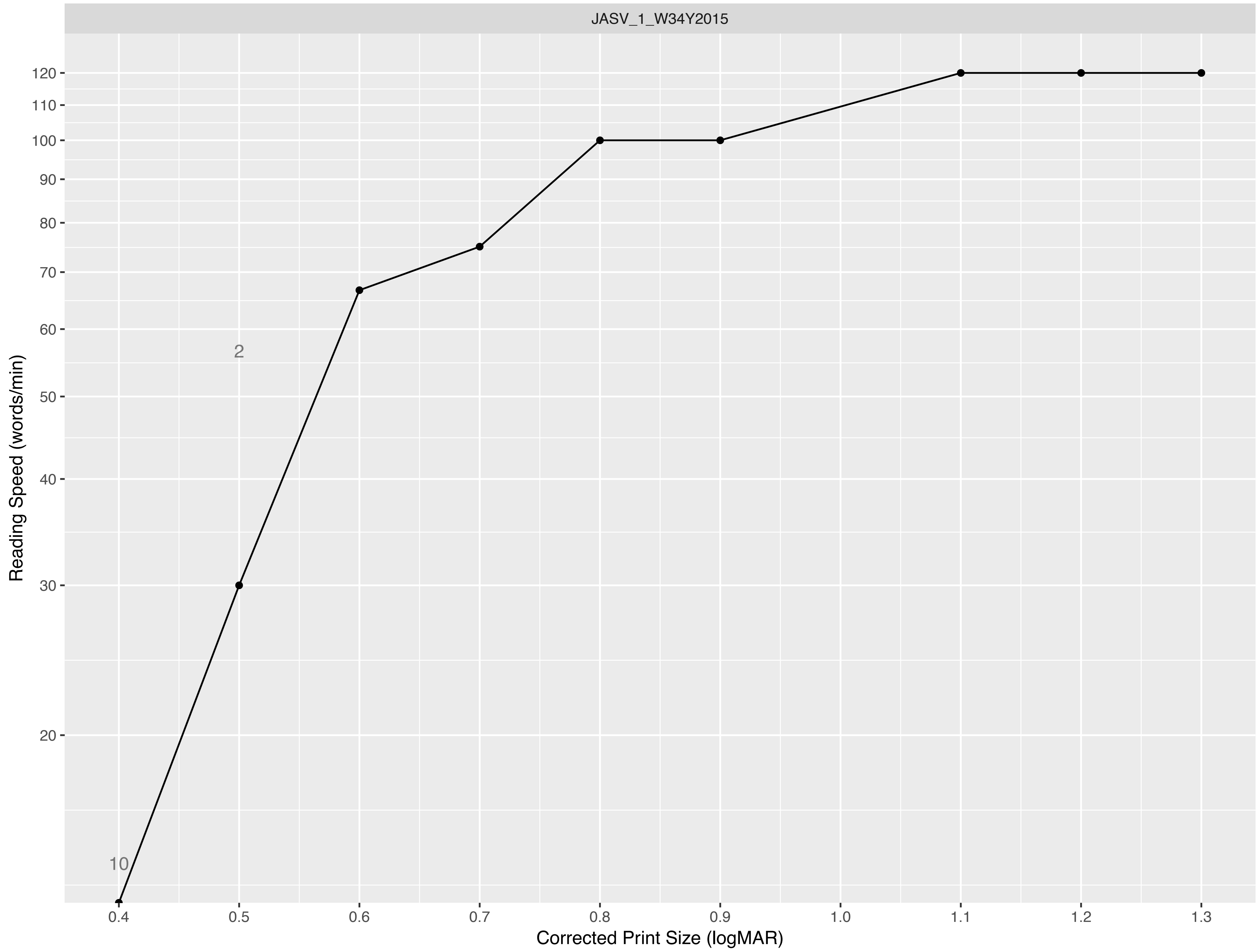
JASV_1_W34Y2015
Reading Speed (words/min)
120
110
100
90
80
70
60
50
40
30
20
2
10
0.4
0.5
0.6
0.7
0.8
0.9
1.0
1.1
1.2
1.3
Corrected Print Size (logMAR)

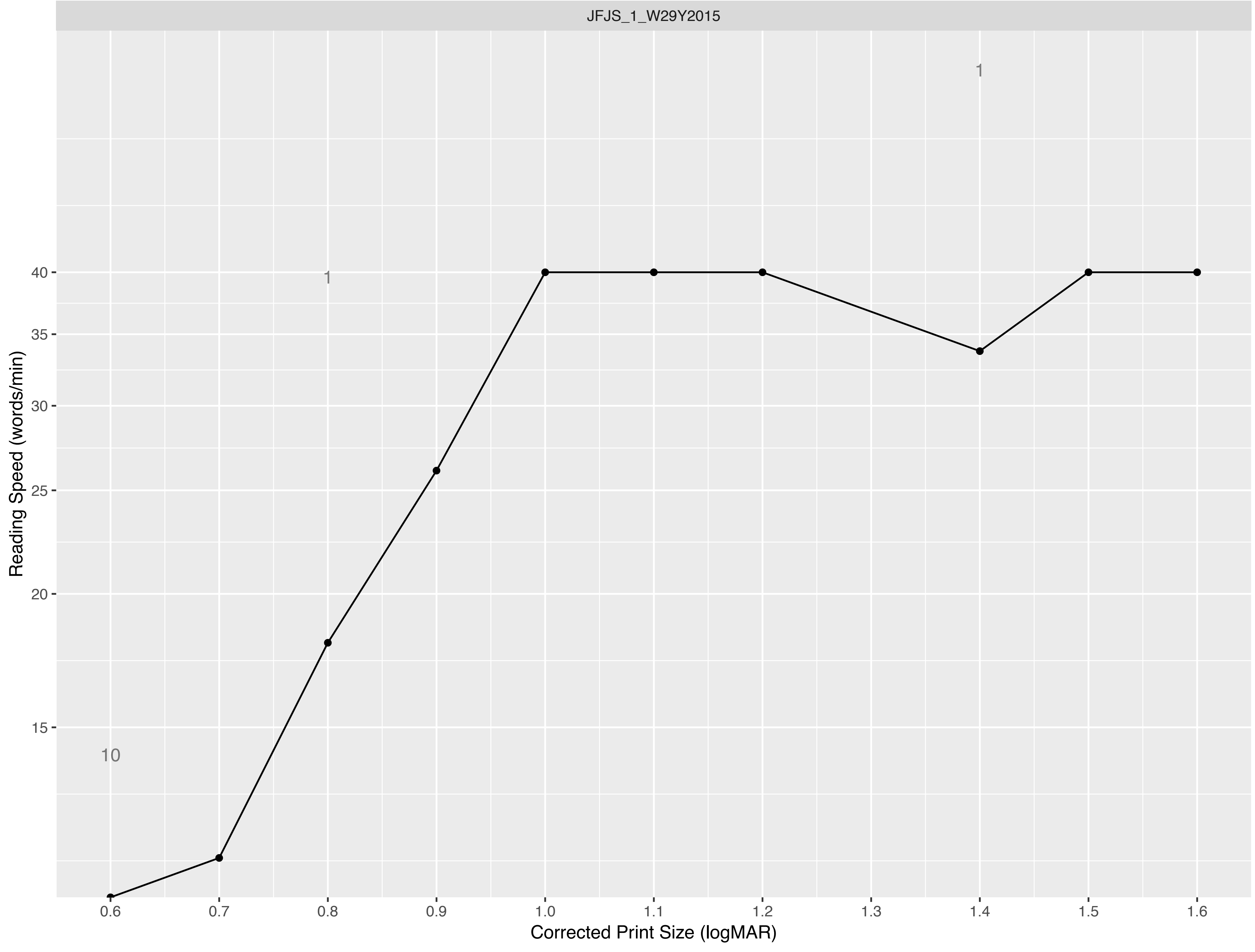

JFJS_1_W29Y2015
Reading Speed (words/min)
40
35
30
25
20
15
1
1
10
0.6
0.7
0.8
0.9
1.0
1.1
1.2
1.3
1.4
1.5
1.6
Corrected Print Size (logMAR)

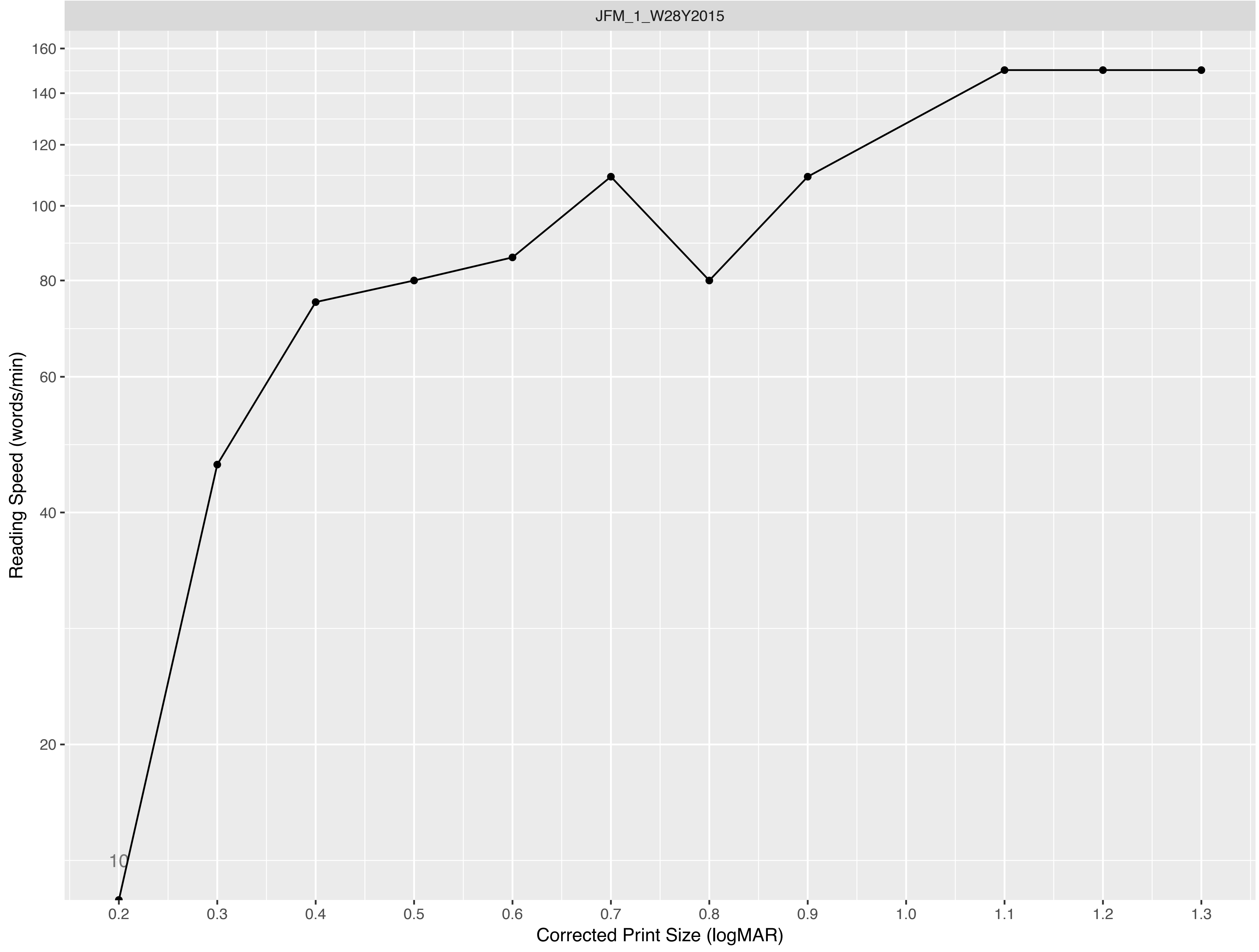

JFM_1_W28Y2015
Reading Speed (words/min)
160
140
120
100
80
60
40
20
10
0.2
0.3
0.4
0.5
0.6
0.7
0.8
0.9
1.0
1.1
1.2
1.3
Corrected Print Size (logMAR)

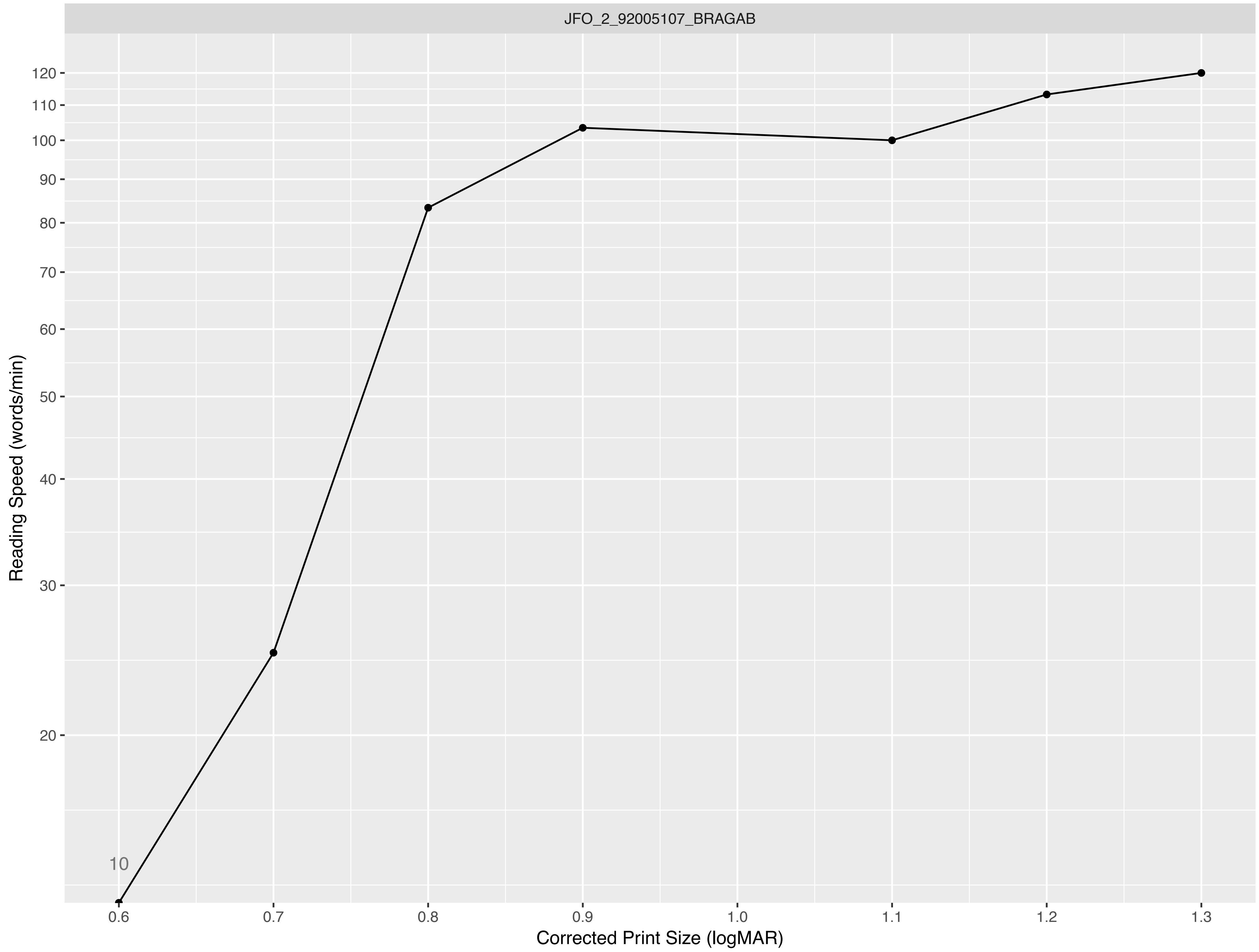

JFO_2_92005107_BRAGAB
Reading Speed (words/min)
120
110
100
90
80
70
60
50
40
30
20
10
0.6
0.7
0.8
0.9
1.0
1.1
1.2
1.3
Corrected Print Size (logMAR)

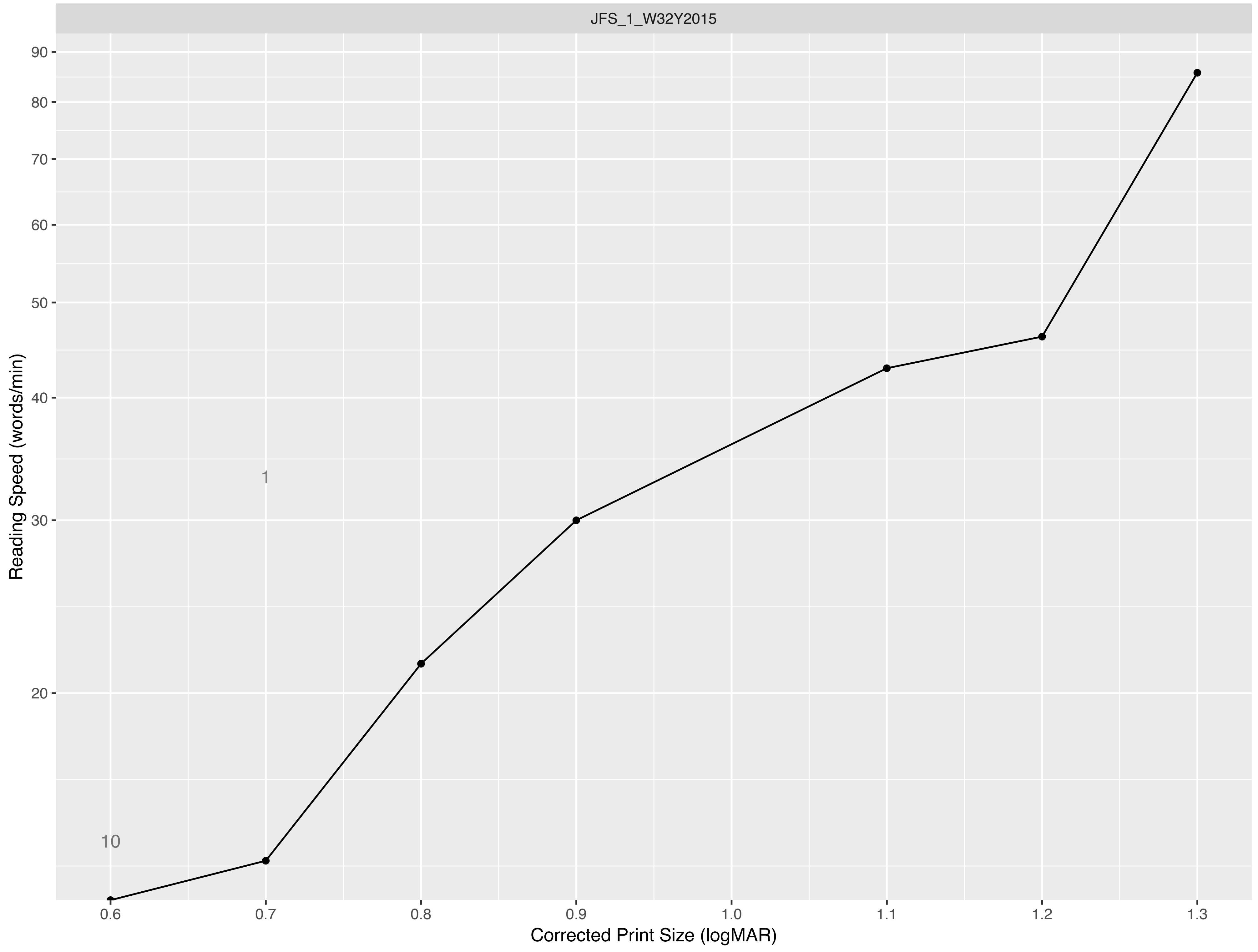

JFS_1_W32Y2015
Reading Speed (words/min)
90
80
70
60
50
40
30
20
1
10
0.6
0.7
0.8
0.9
1.0
1.1
1.2
1.3
Corrected Print Size (logMAR)

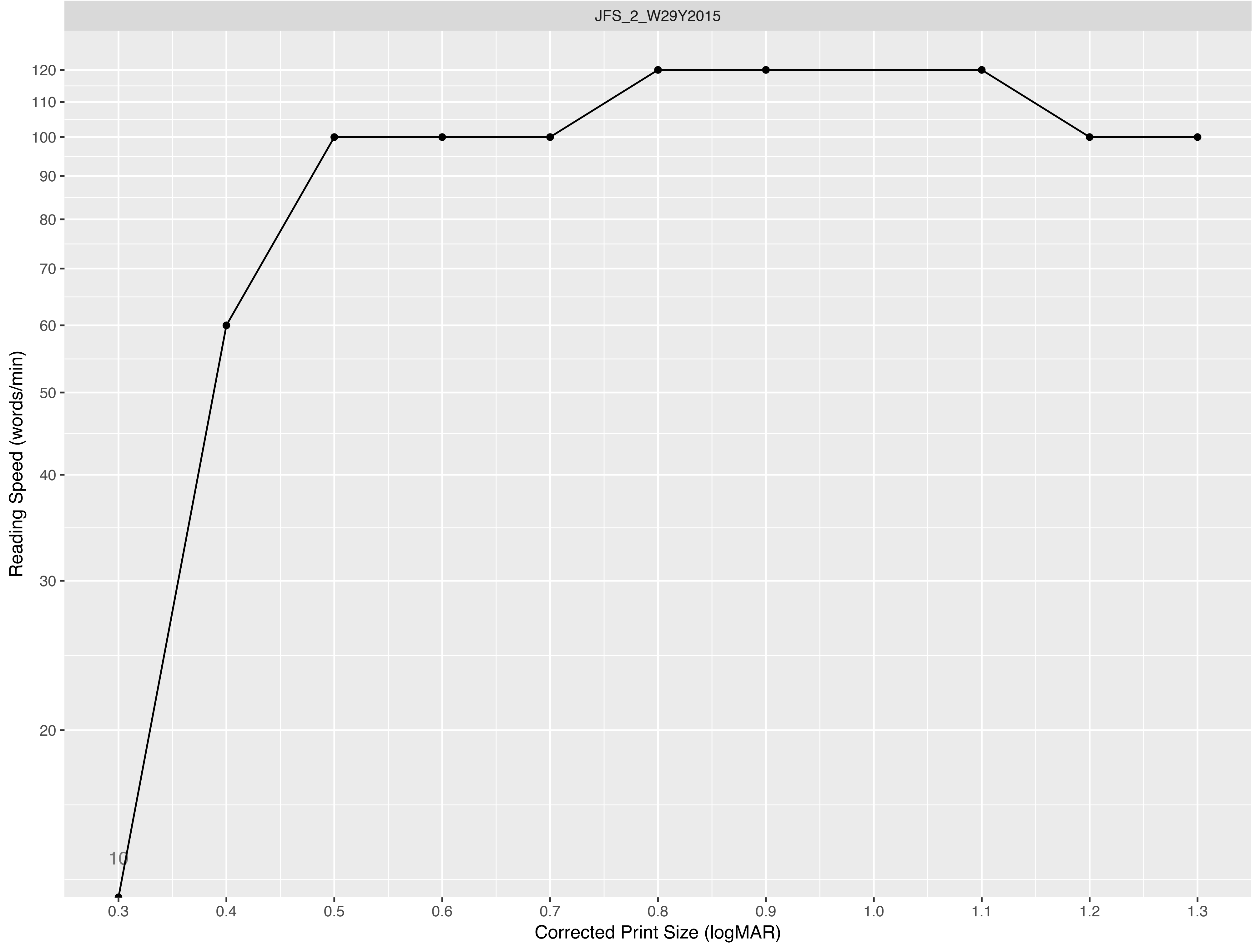

JFS_2_W29Y2015
Reading Speed (words/min)
120
110
100
90
80
70
60
50
40
30
20
10
0.3
0.4
0.5
0.6
0.7
0.8
0.9
1.0
1.1
1.2
1.3
Corrected Print Size (logMAR)

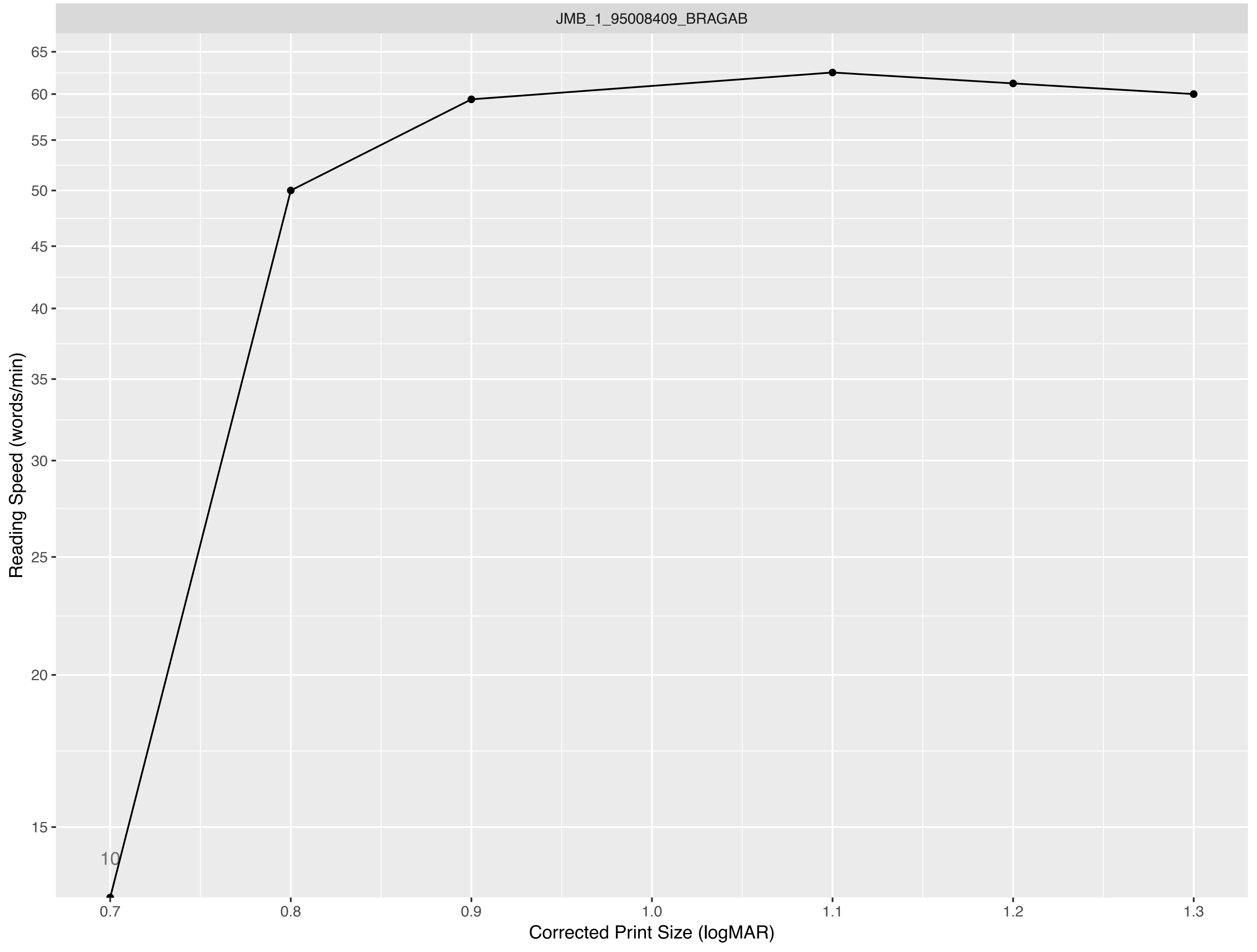

JMB_1_95008409_BRAGAB
Reading Speed (words/min)
Corrected Print Size (logMAR)
65
60
55
50
45
40
35
30
25
20
15
10
0.7
0.8
0.9
1.0
1.1
1.2
1.3

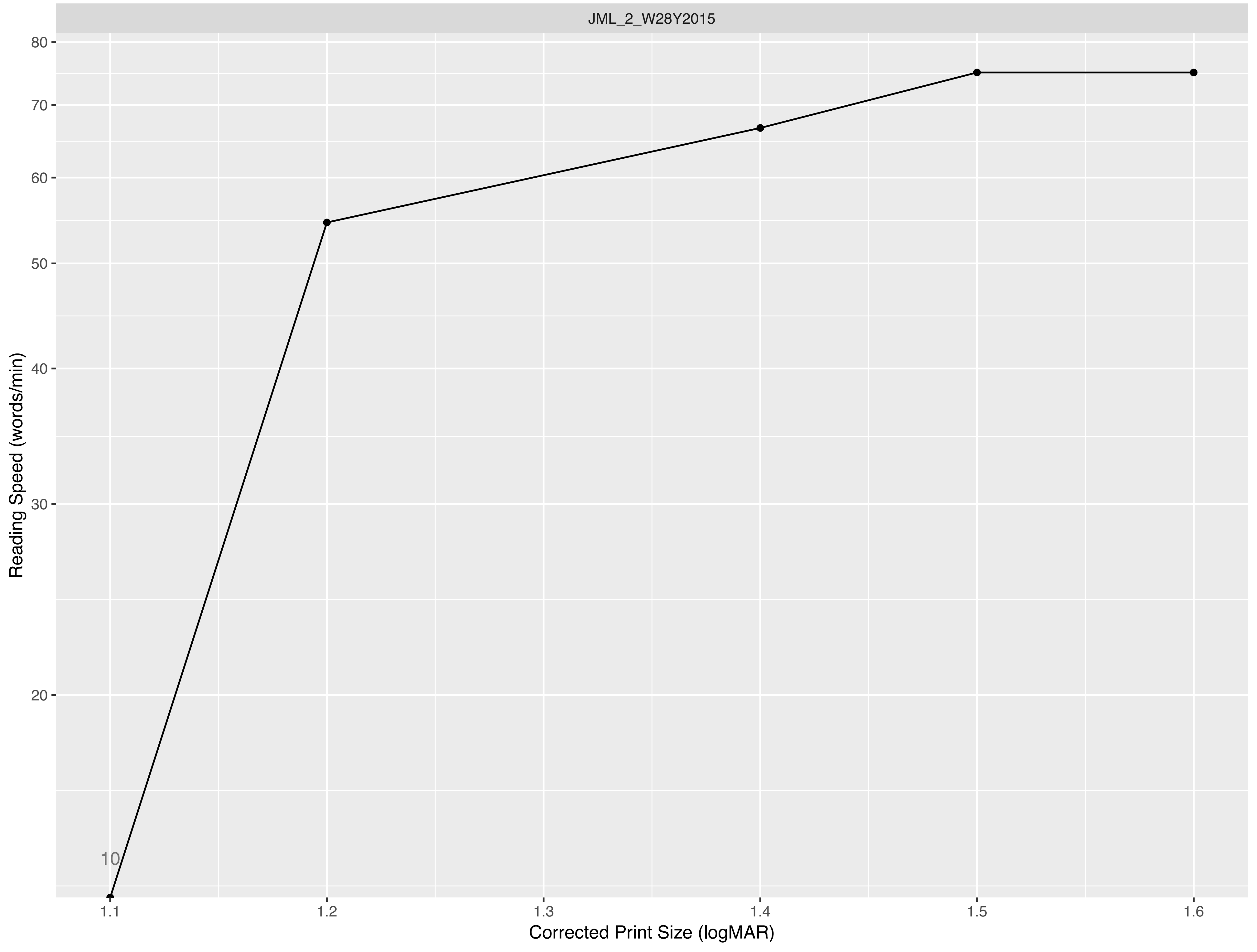

JML_2_W28Y2015
80
70
60
50
40
30
20
10
Reading Speed (words/min)
1.1
1.2
1.3
1.4
1.5
1.6
Corrected Print Size (logMAR)

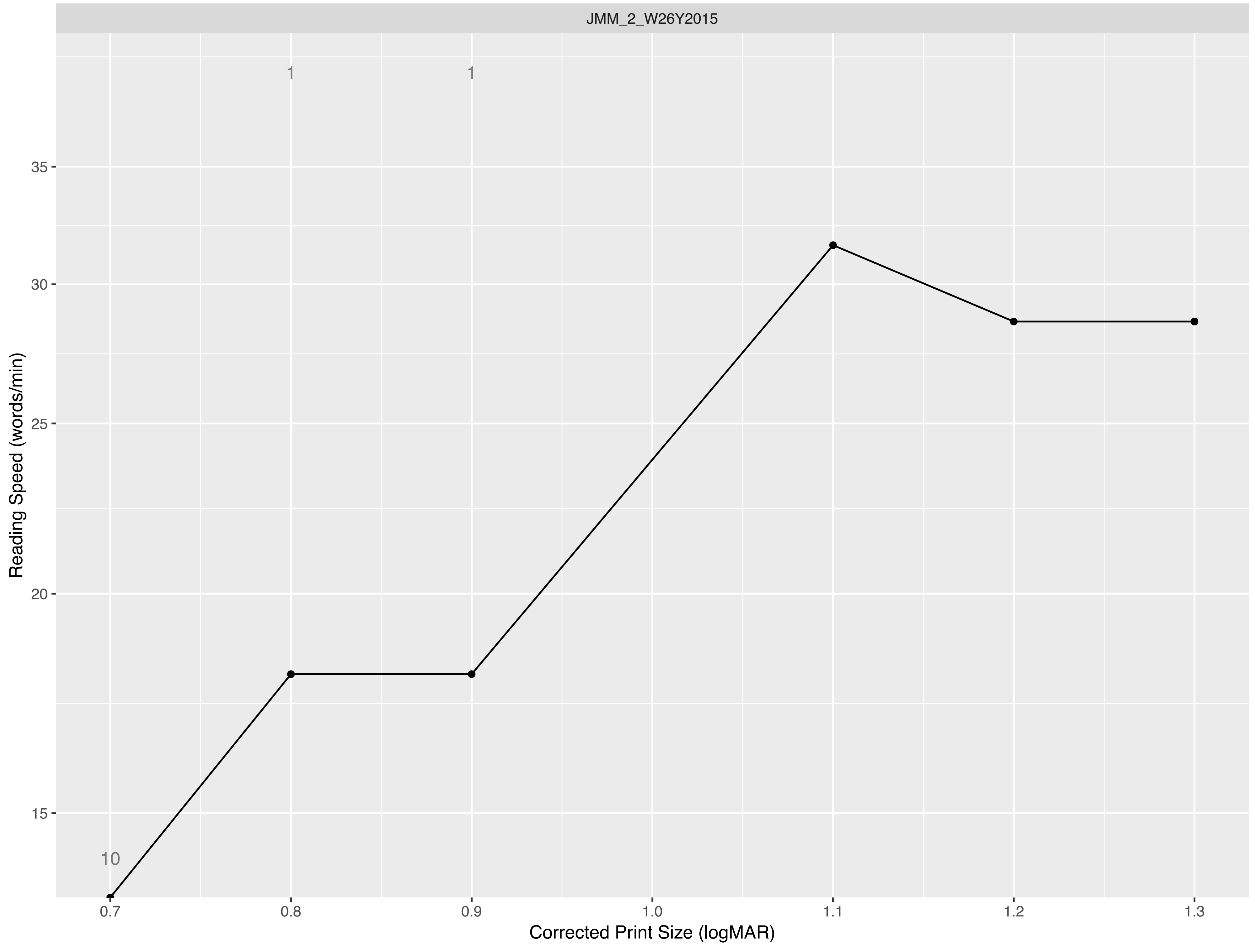
JMM_2_W26Y2015
1
1
10
35
30
25
20
15
Reading Speed (words/min)
0.7
0.8
0.9
1.0
1.1
1.2
1.3
Corrected Print Size (logMAR)

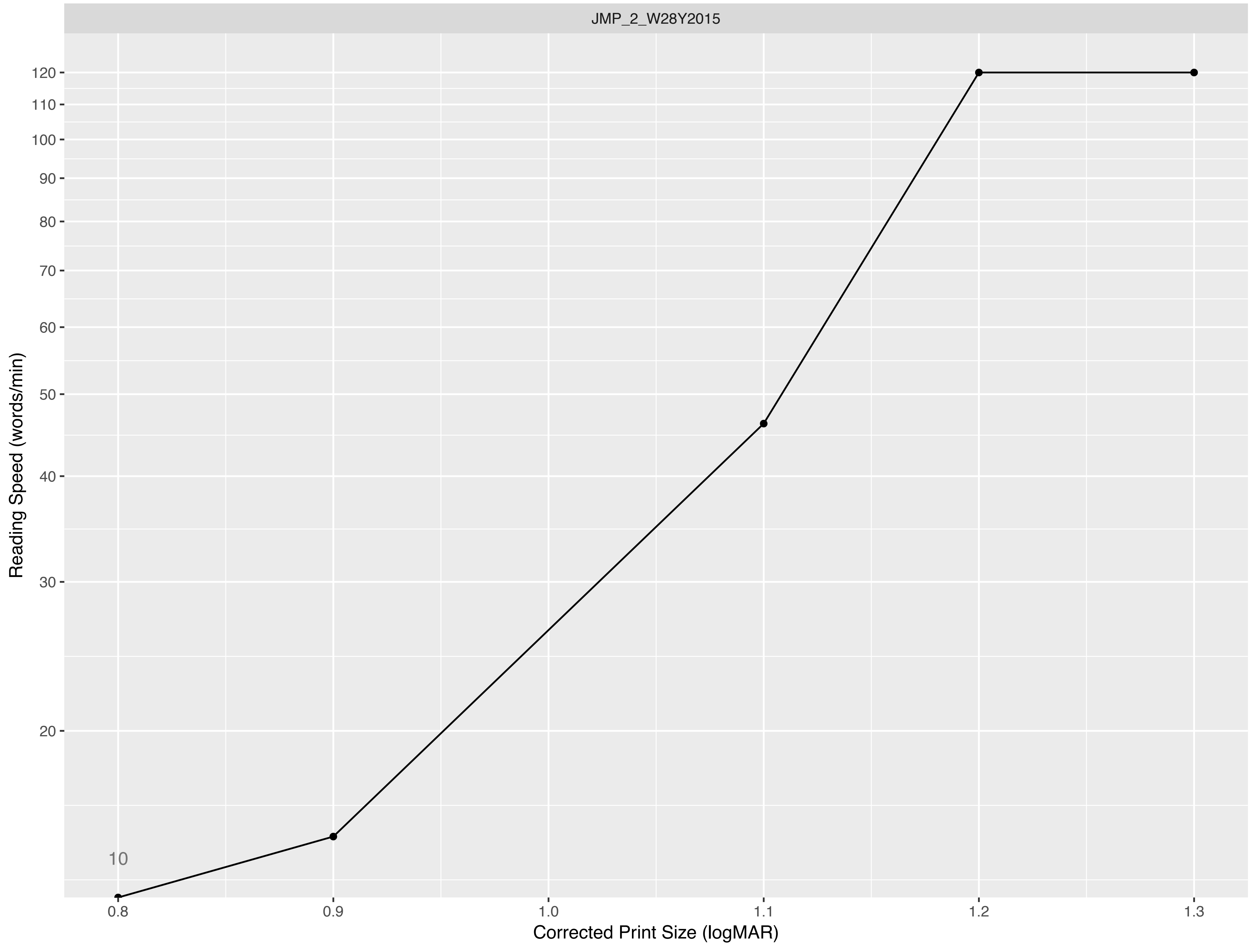

JMP_2_W28Y2015
Reading Speed (words/min)
120
110
100
90
80
70
60
50
40
30
20
10
0.8
0.9
1.0
1.1
1.2
1.3
Corrected Print Size (logMAR)

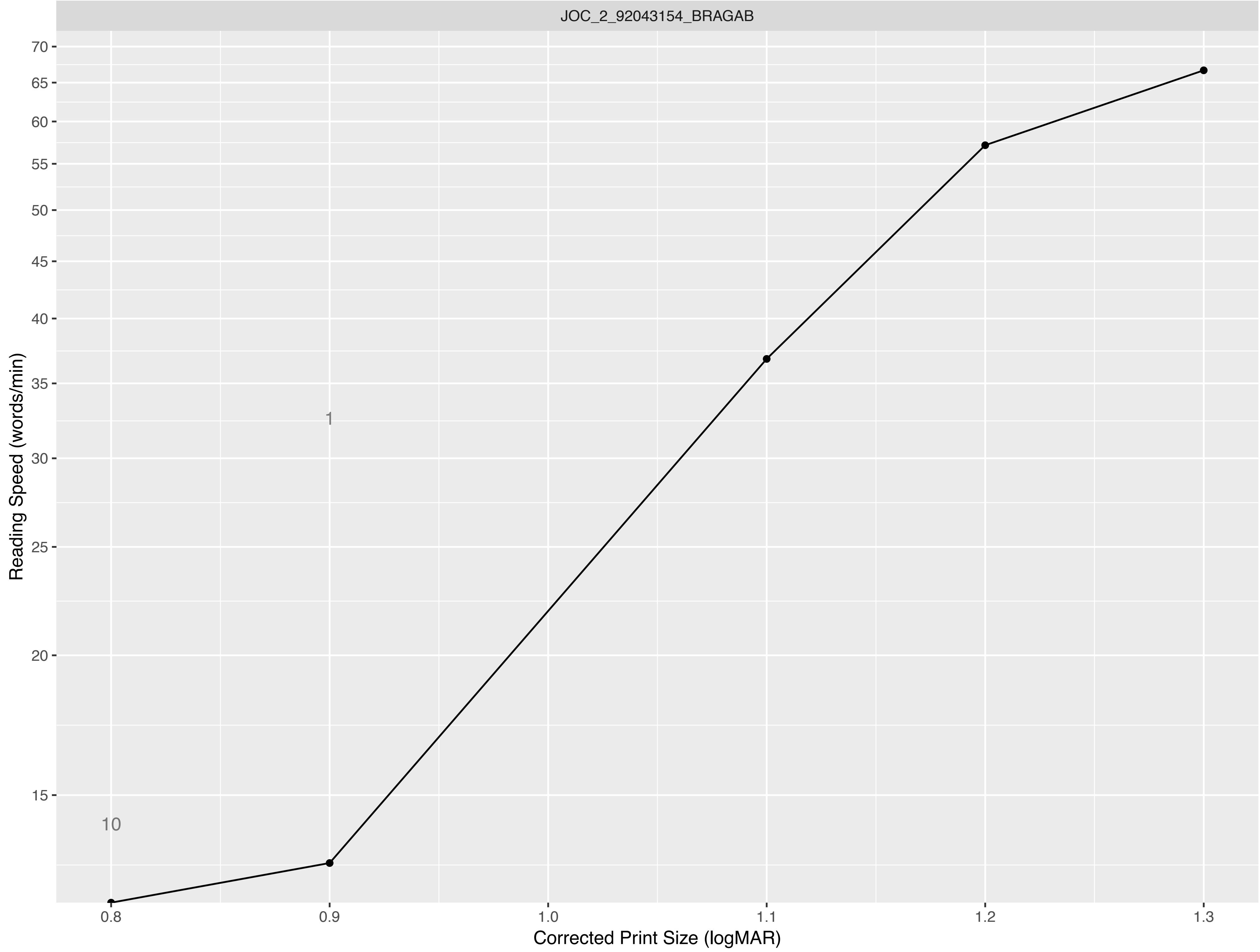
JOC_2_92043154_BRAGAB
Reading Speed (words/min)
70
65
60
55
50
45
40
35
30
25
20
15
1
10
0.8
0.9
1.0
1.1
1.2
1.3
Corrected Print Size (logMAR)

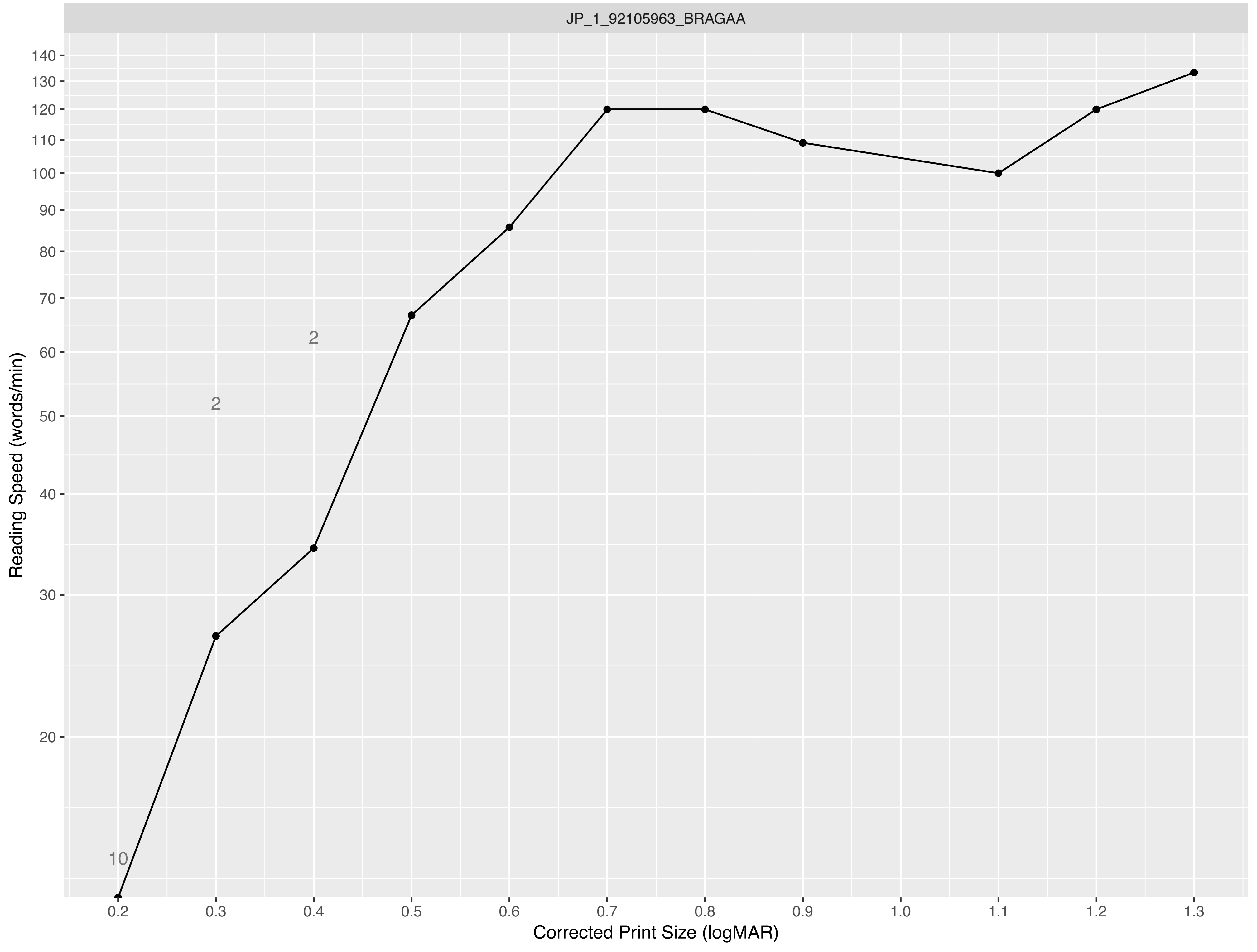
JP_1_92105963_BRAGAA
Reading Speed (words/min)
140
130
120
110
100
90
80
70
60
50
40
30
20
10
2
2
0.2
0.3
0.4
0.5
0.6
0.7
0.8
0.9
1.0
1.1
1.2
1.3
Corrected Print Size (logMAR)

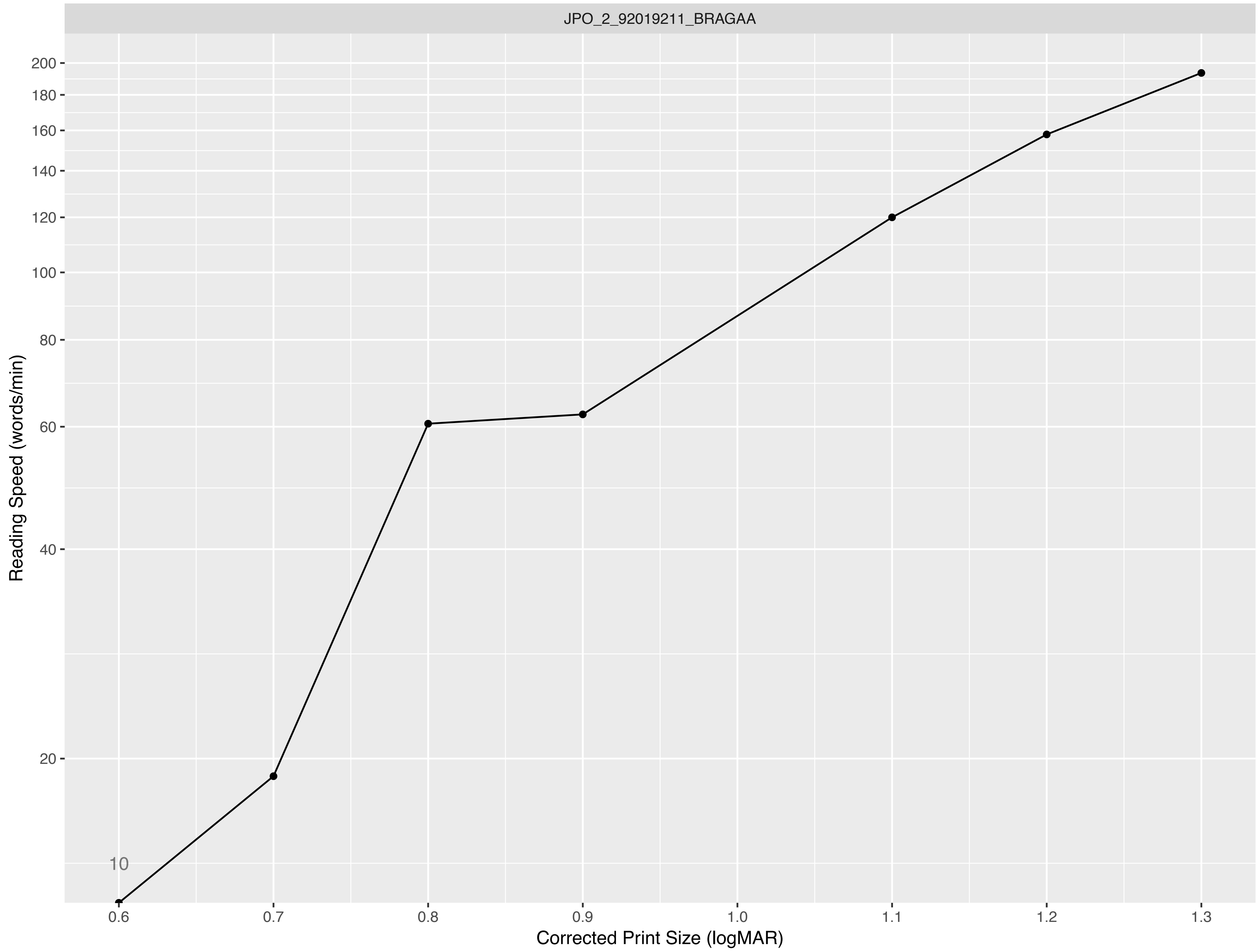
JPO_2_92019211_BRAGAA
Reading Speed (words/min)
200
180
160
140
120
100
80
60
40
20
10
0.6
0.7
0.8
0.9
1.0
1.1
1.2
1.3
Corrected Print Size (logMAR)

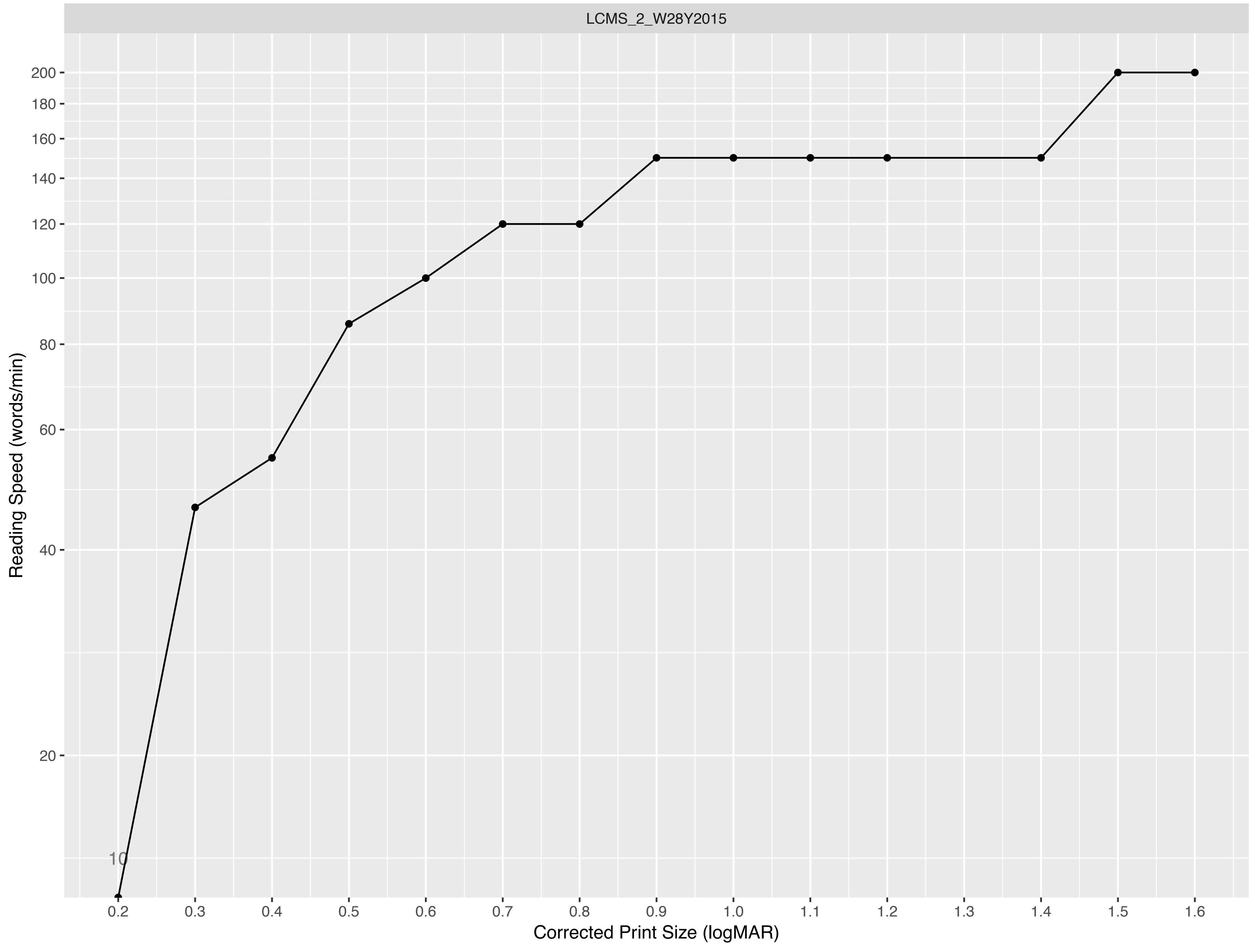
LCMS_2_W28Y2015
Reading Speed (words/min)
200
180
160
140
120
100
80
60
40
20
10
0.2
0.3
0.4
0.5
0.6
0.7
0.8
0.9
1.0
1.1
1.2
1.3
1.4
1.5
1.6
Corrected Print Size (logMAR)

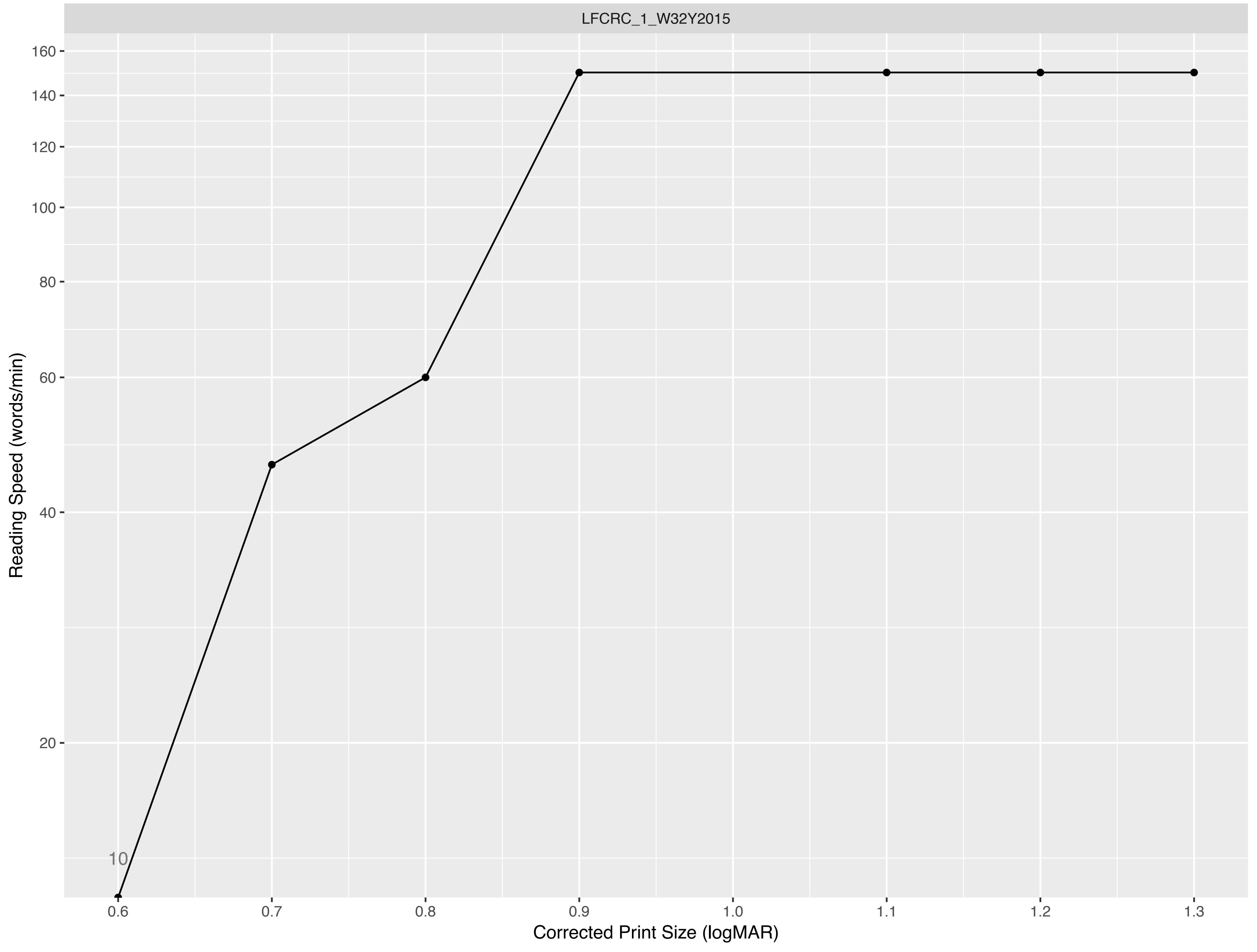

LFCRC_1_W32Y2015
Reading Speed (words/min)
160
140
120
100
80
60
40
20
10
0.6
0.7
0.8
0.9
1.0
1.1
1.2
1.3
Corrected Print Size (logMAR)

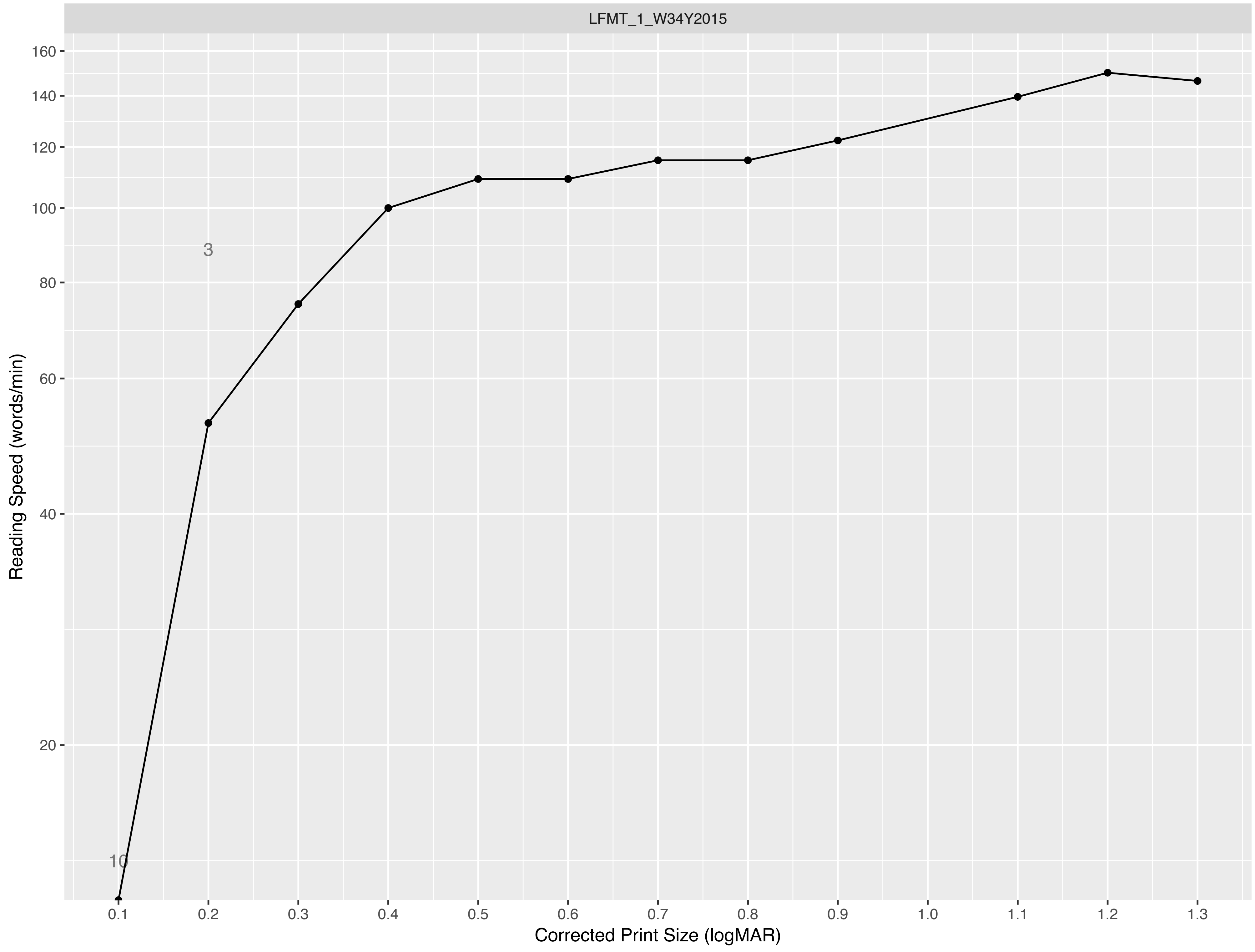
LFMT_1_W34Y2015
Reading Speed (words/min)
Corrected Print Size (logMAR)
160
140
120
100
80
60
40
20
3
10
0.1
0.2
0.3
0.4
0.5
0.6
0.7
0.8
0.9
1.0
1.1
1.2
1.3

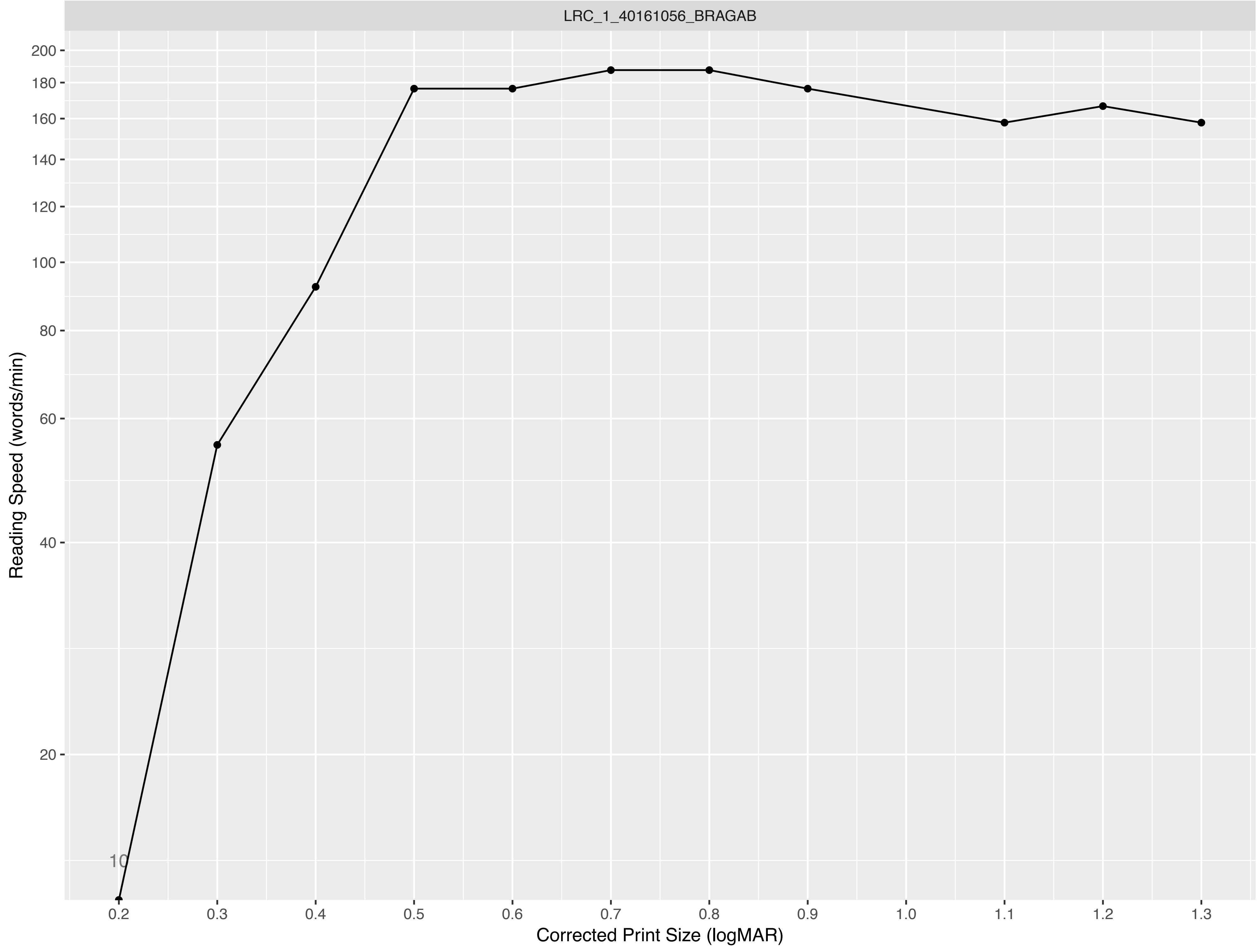
LRC_1_40161056_BRAGAB
Reading Speed (words/min)
200
180
160
140
120
100
80
60
40
20
10
0.2
0.3
0.4
0.5
0.6
0.7
0.8
0.9
1.0
1.1
1.2
1.3
Corrected Print Size (logMAR)

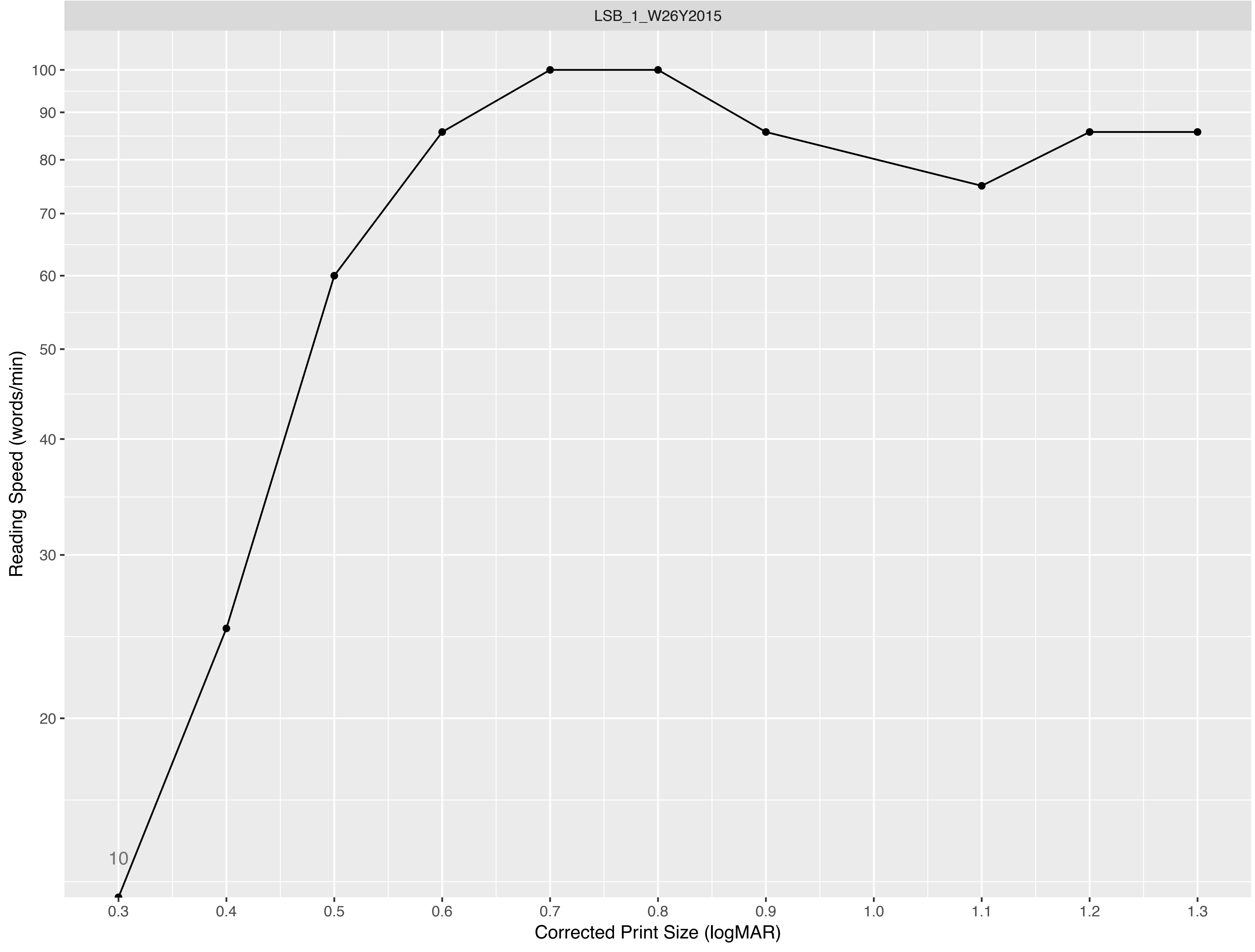
LSB_1_W26Y2015
Reading Speed (words/min)
100
90
80
70
60
50
40
30
20
10
0.3
0.4
0.5
0.6
0.7
0.8
0.9
1.0
1.1
1.2
1.3
Corrected Print Size (logMAR)

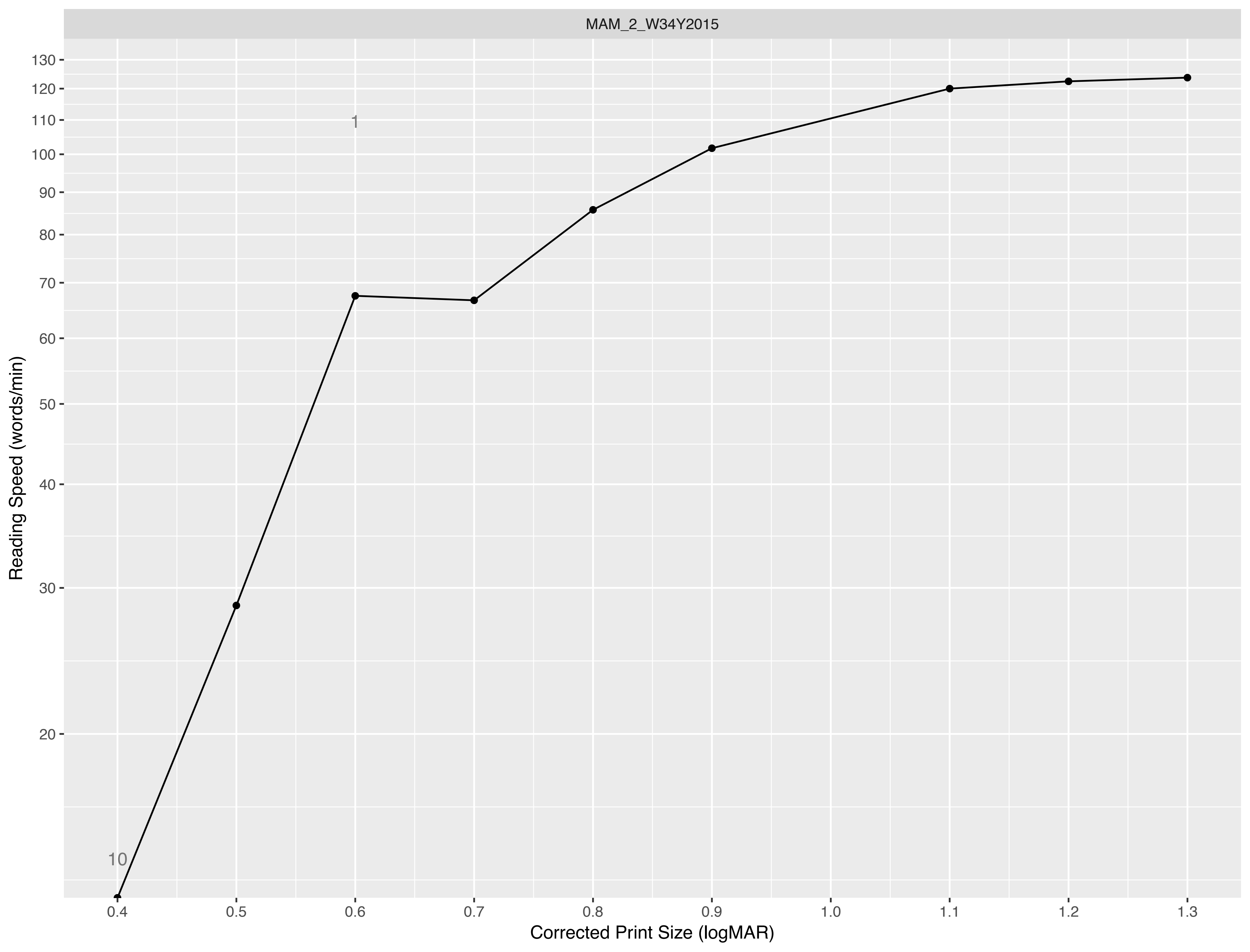

MAM_2_W34Y2015
Reading Speed (words/min)
130
120
110
100
90
80
70
60
50
40
30
20
1
10
0.4
0.5
0.6
0.7
0.8
0.9
1.0
1.1
1.2
1.3
Corrected Print Size (logMAR)

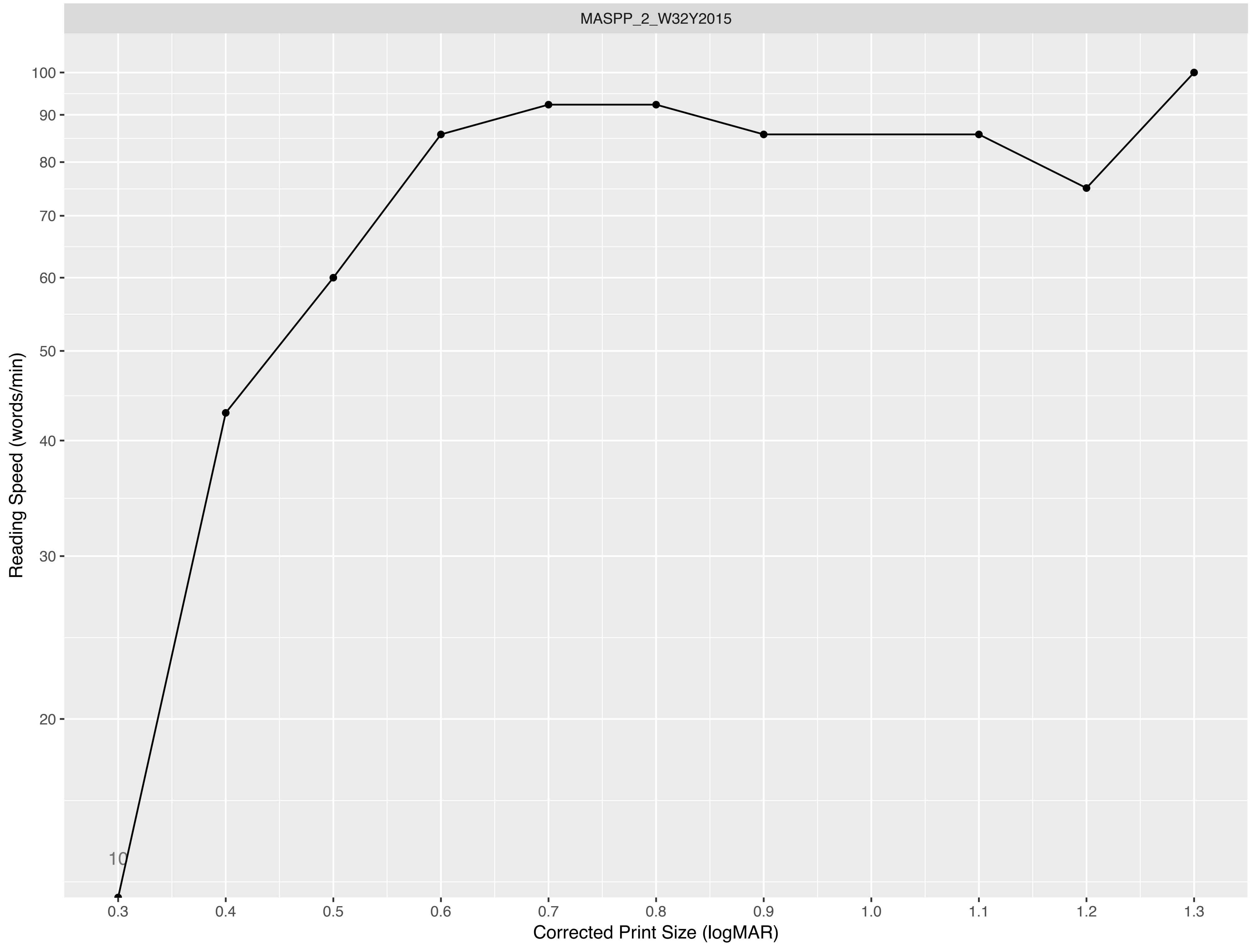

MASPP_2_W32Y2015
Reading Speed (words/min)
100
90
80
70
60
50
40
30
20
10
0.3
0.4
0.5
0.6
0.7
0.8
0.9
1.0
1.1
1.2
1.3
Corrected Print Size (logMAR)

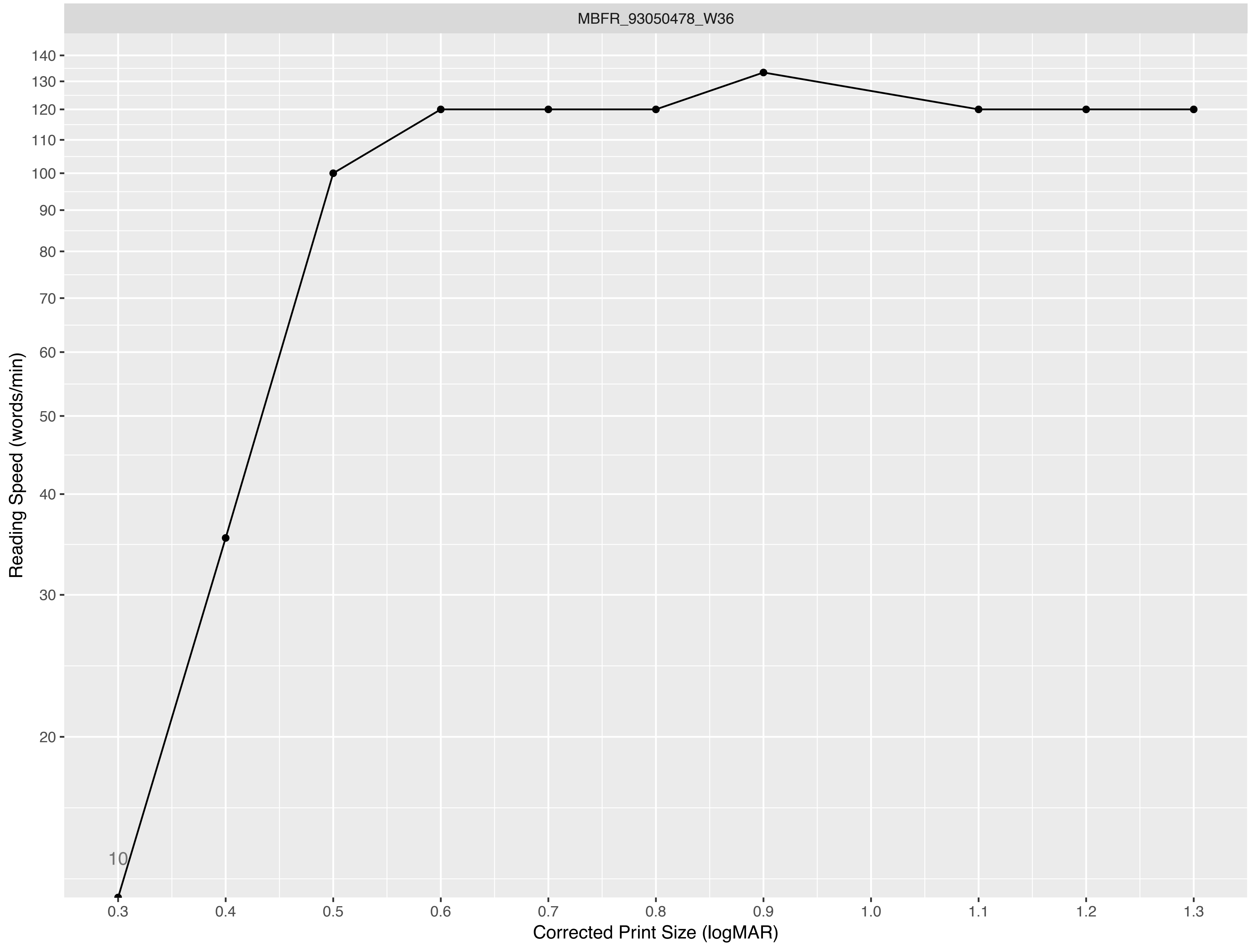
MBFR_93050478_W36
Reading Speed (words/min)
140
130
120
110
100
90
80
70
60
50
40
30
20
10
Corrected Print Size (logMAR)
0.3
0.4
0.5
0.6
0.7
0.8
0.9
1.0
1.1
1.2
1.3

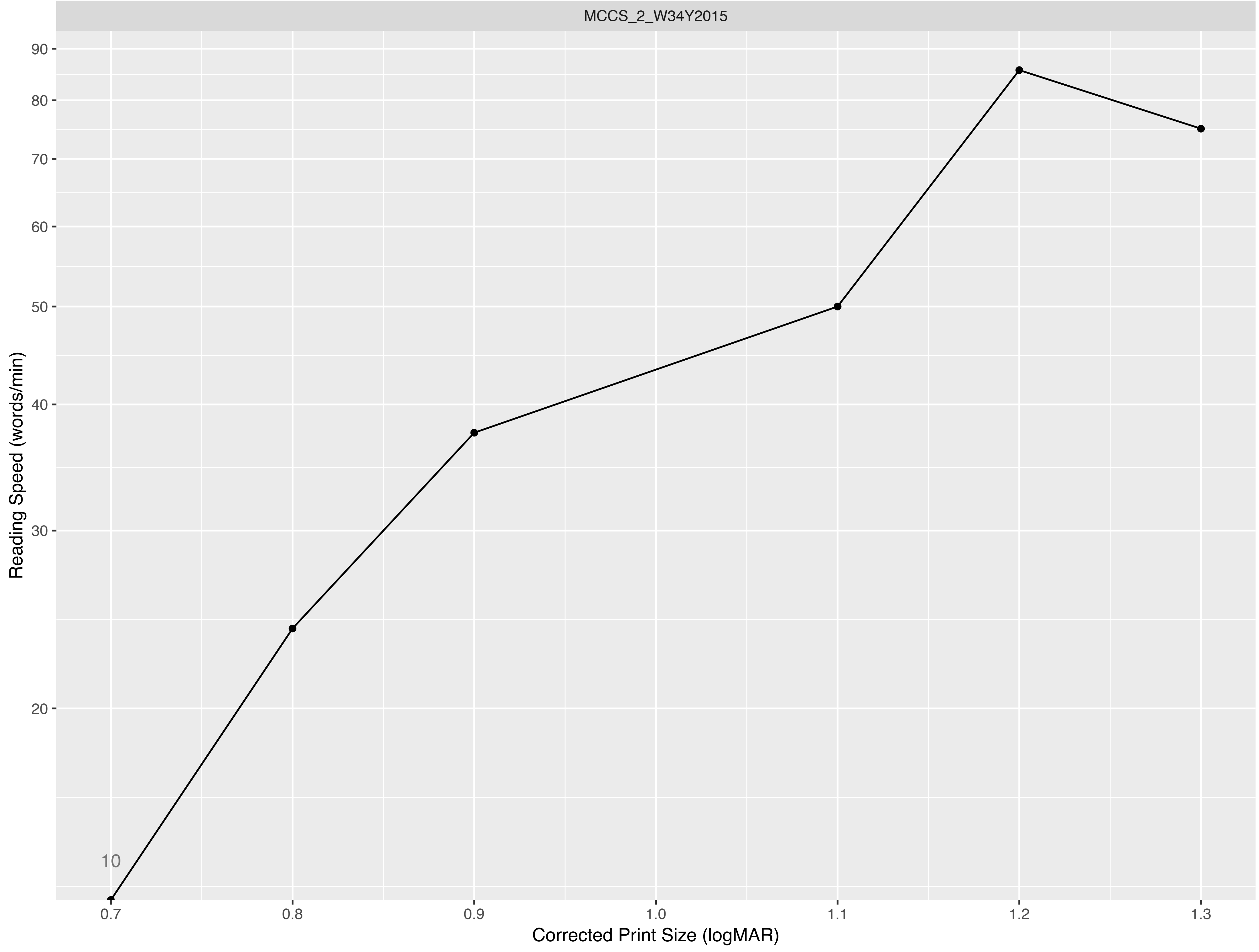
MCCS_2_W34Y2015
Reading Speed (words/min)
90
80
70
60
50
40
30
20
10
0.7
0.8
0.9
1.0
1.1
1.2
1.3
Corrected Print Size (logMAR)

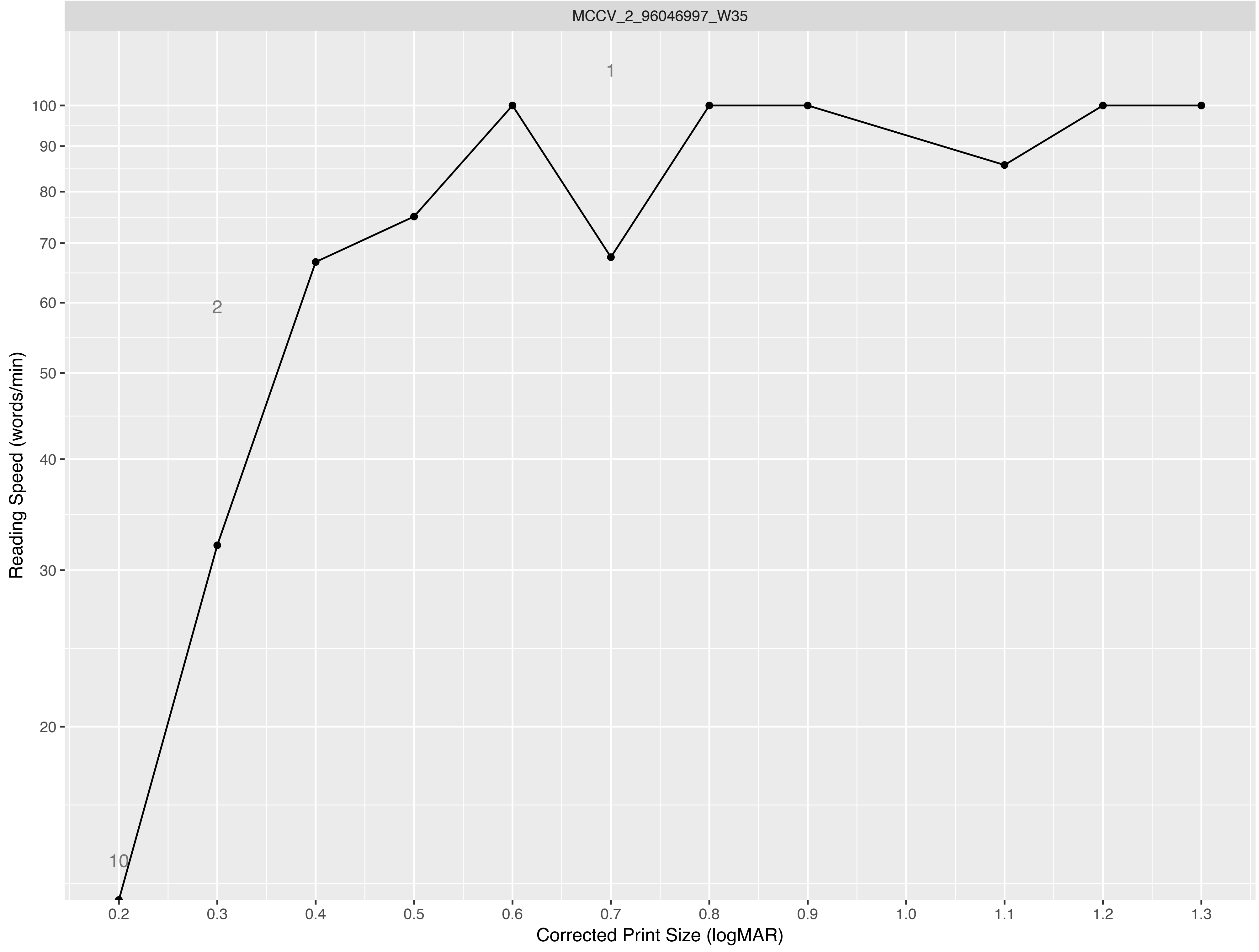

MCCV_2_96046997_W35
1
2
10
100
90
80
70
60
50
40
30
20
Reading Speed (words/min)
0.2
0.3
0.4
0.5
0.6
0.7
0.8
0.9
1.0
1.1
1.2
1.3
Corrected Print Size (logMAR)

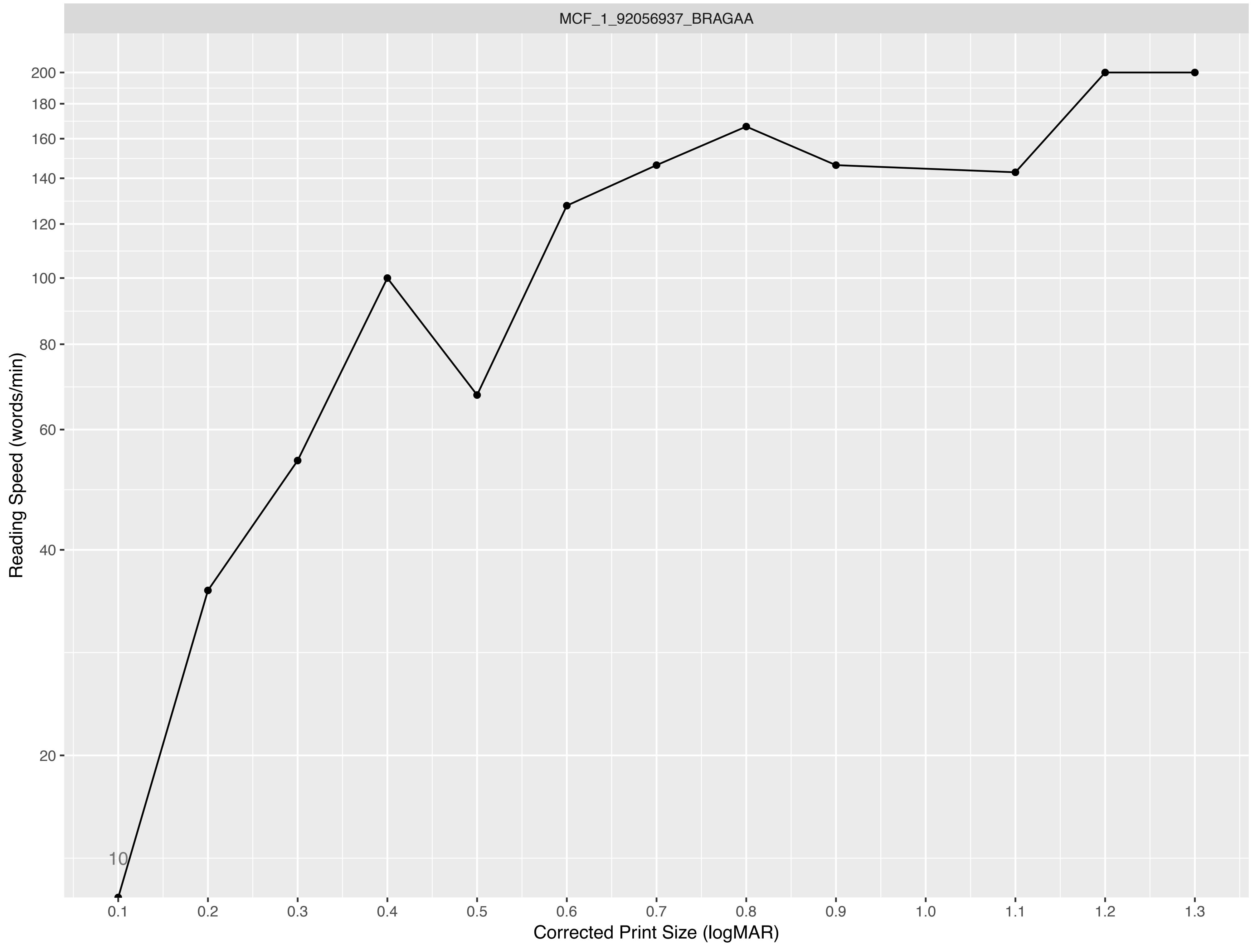
MCF_1_92056937_BRAGAA
Reading Speed (words/min)
200
180
160
140
120
100
80
60
40
20
10
0.1
0.2
0.3
0.4
0.5
0.6
0.7
0.8
0.9
1.0
1.1
1.2
1.3
Corrected Print Size (logMAR)

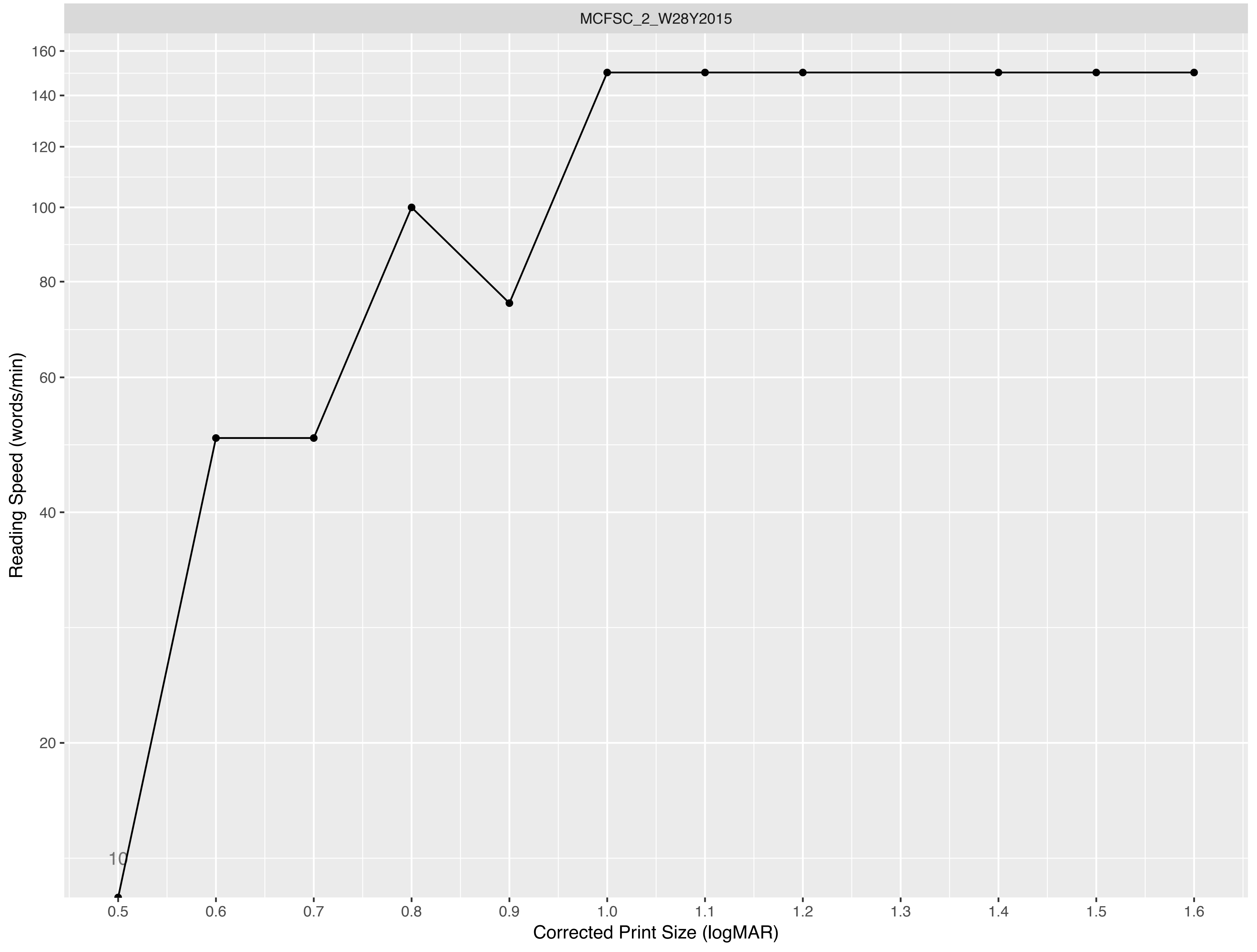
MCFSC_2_W28Y2015
Reading Speed (words/min)
160
140
120
100
80
60
40
20
10
Corrected Print Size (logMAR)
0.5
0.6
0.7
0.8
0.9
1.0
1.1
1.2
1.3
1.4
1.5
1.6

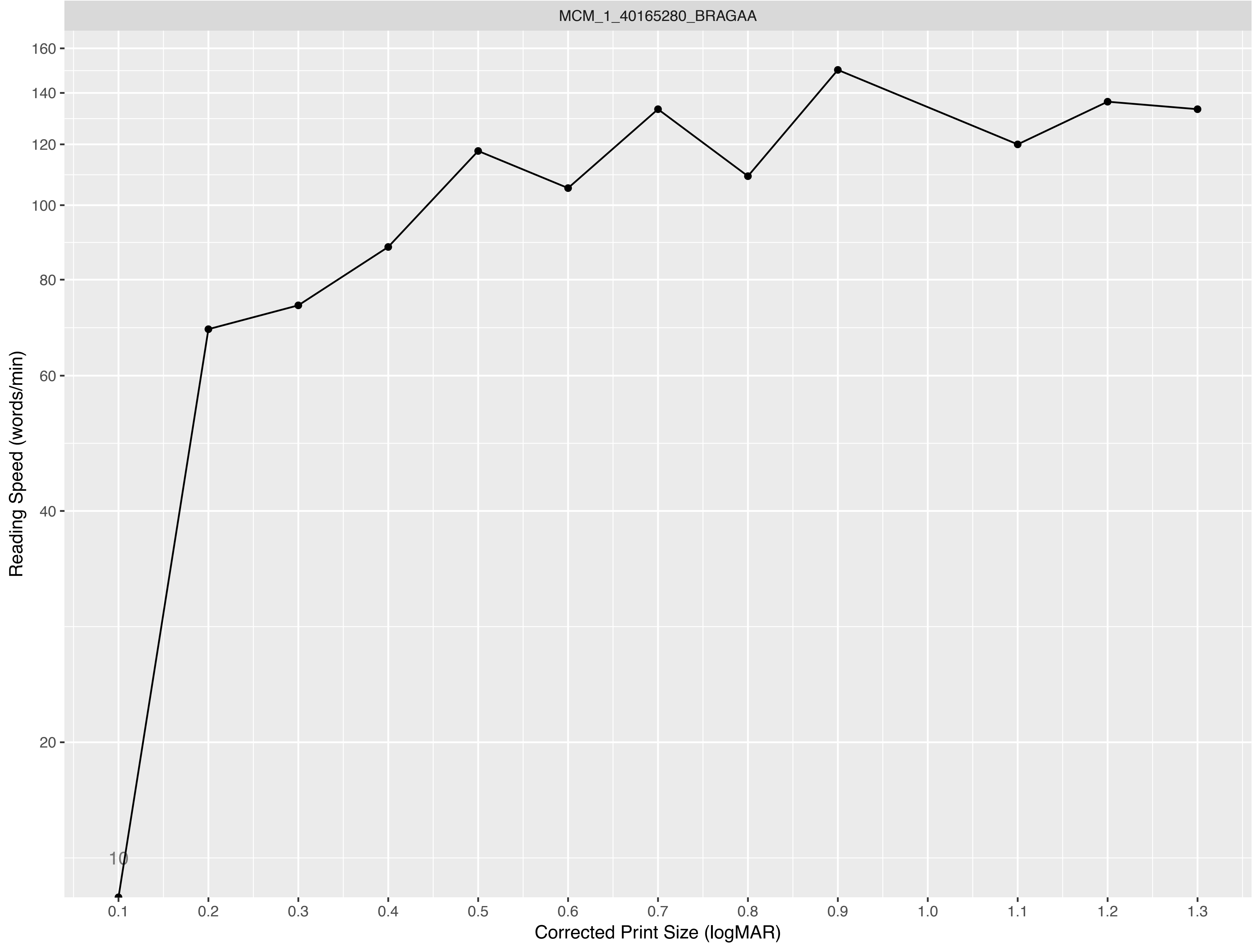

MCM_1_40165280_BRAGAA
Reading Speed (words/min)
160
140
120
100
80
60
40
20
10
0.1
0.2
0.3
0.4
0.5
0.6
0.7
0.8
0.9
1.0
1.1
1.2
1.3
Corrected Print Size (logMAR)

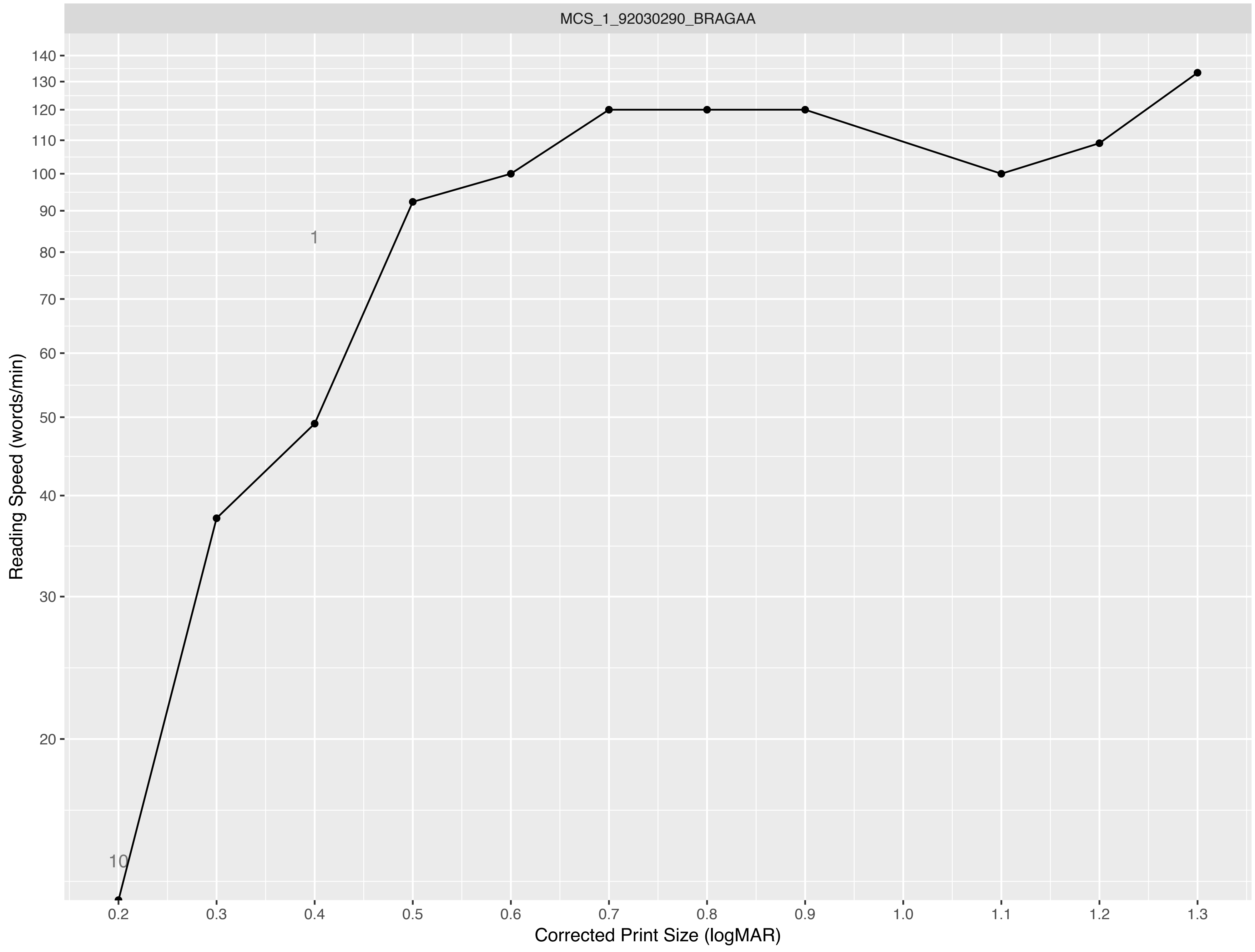

MCS_1_92030290_BRAGAA
Reading Speed (words/min)
140
130
120
110
100
90
80
70
60
50
40
30
20
1
10
0.2
0.3
0.4
0.5
0.6
0.7
0.8
0.9
1.0
1.1
1.2
1.3
Corrected Print Size (logMAR)

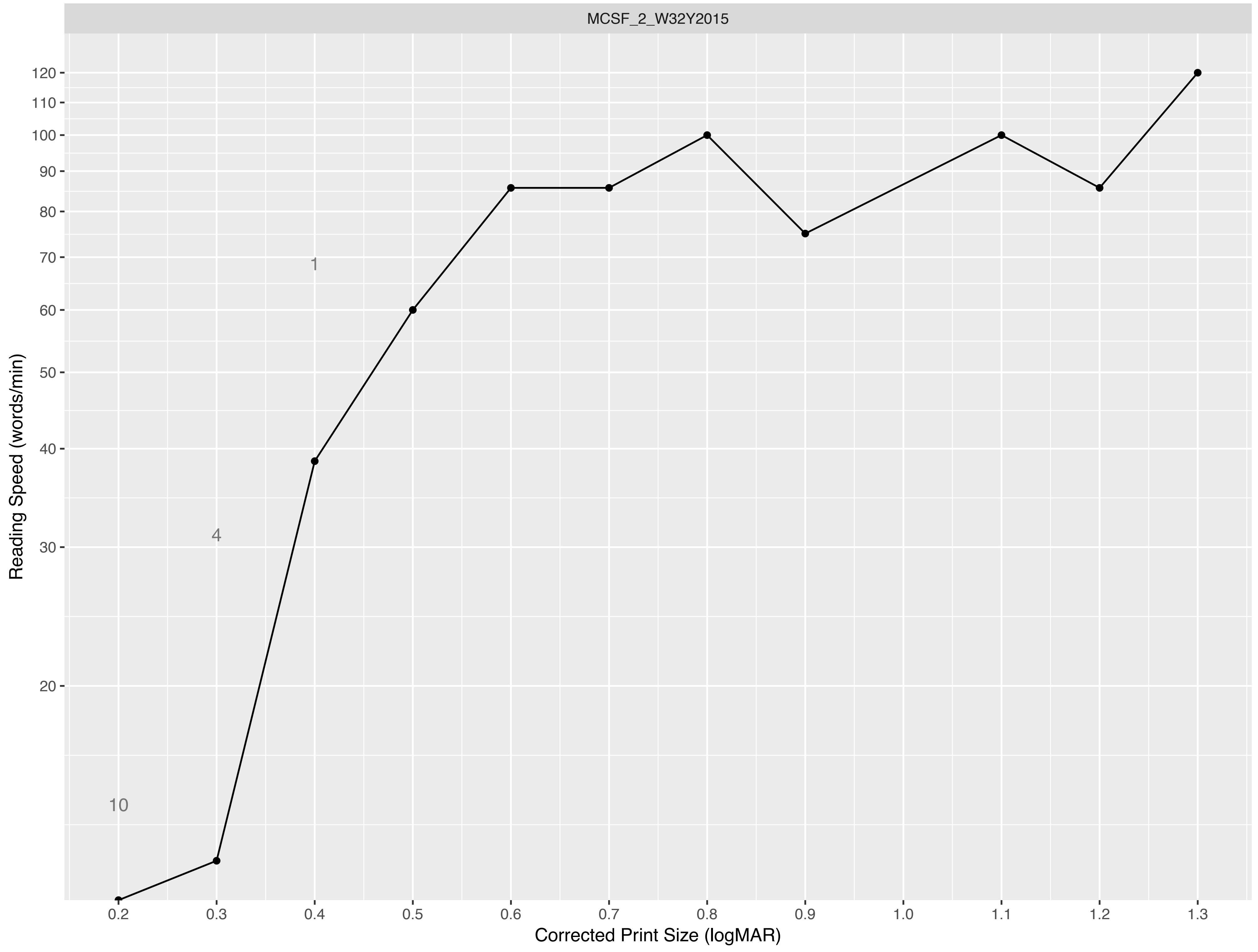
MCSF_2_W32Y2015
Reading Speed (words/min)
120
110
100
90
80
70
60
50
40
30
20
1
4
10
0.2
0.3
0.4
0.5
0.6
0.7
0.8
0.9
1.0
1.1
1.2
1.3
Corrected Print Size (logMAR)

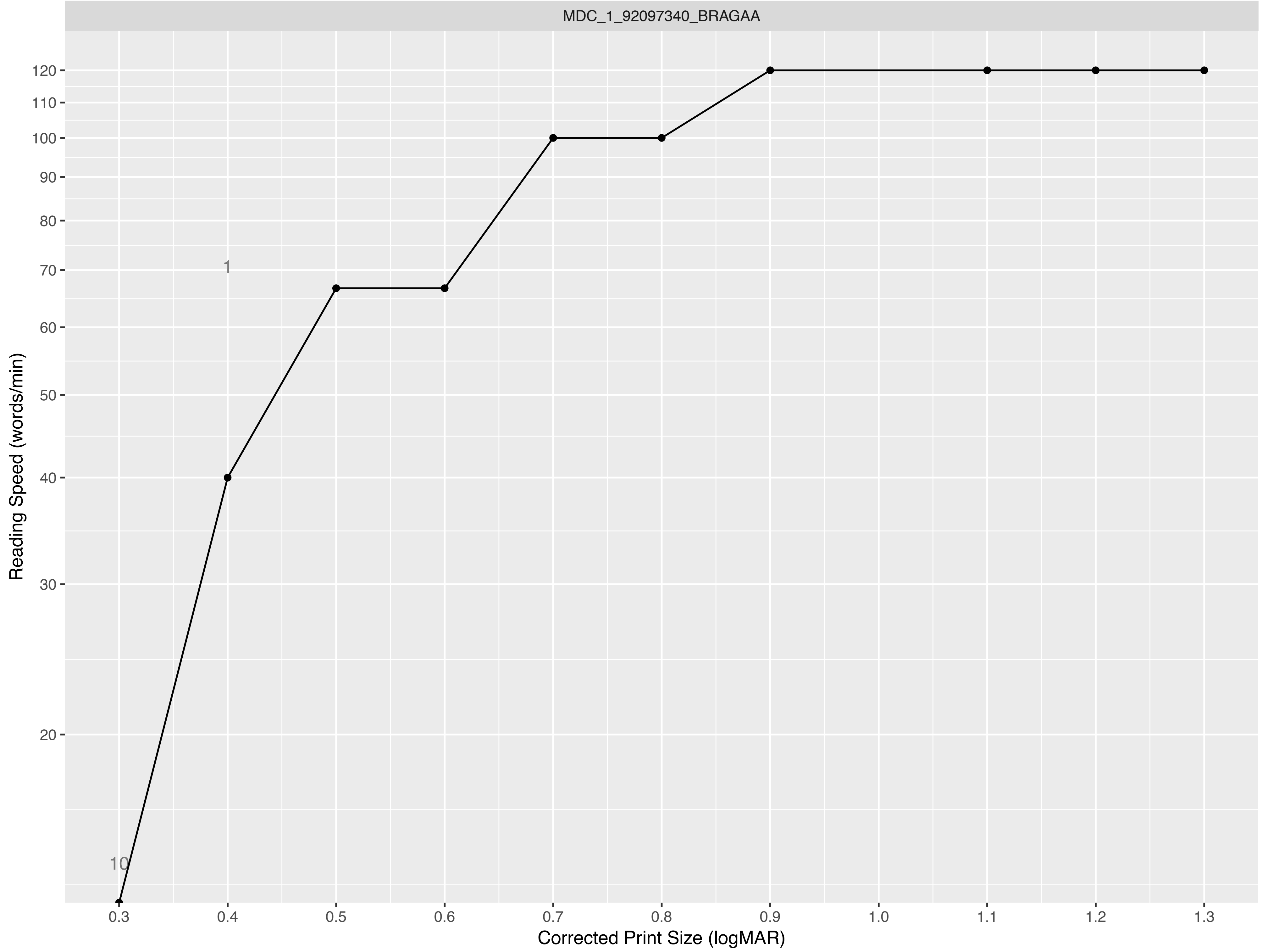

MDC_1_92097340_BRAGAA
Reading Speed (words/min)
120
110
100
90
80
70
60
50
40
30
20
1
10
0.3
0.4
0.5
0.6
0.7
0.8
0.9
1.0
1.1
1.2
1.3
Corrected Print Size (logMAR)

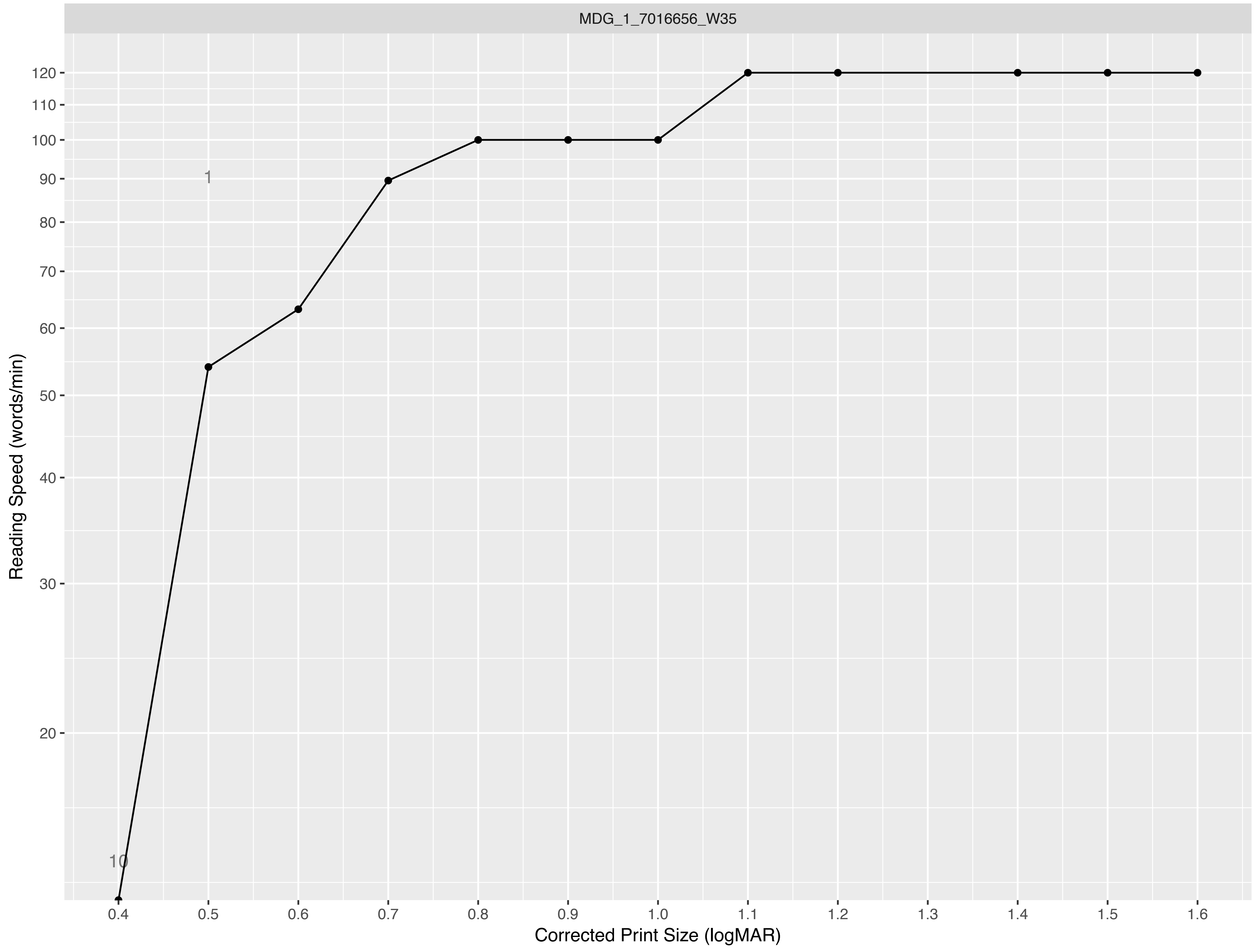
MDG_1_7016656_W35
Reading Speed (words/min)
120
110
100
90
80
70
60
50
40
30
20
1
10
0.4
0.5
0.6
0.7
0.8
0.9
1.0
1.1
1.2
1.3
1.4
1.5
1.6
Corrected Print Size (logMAR)

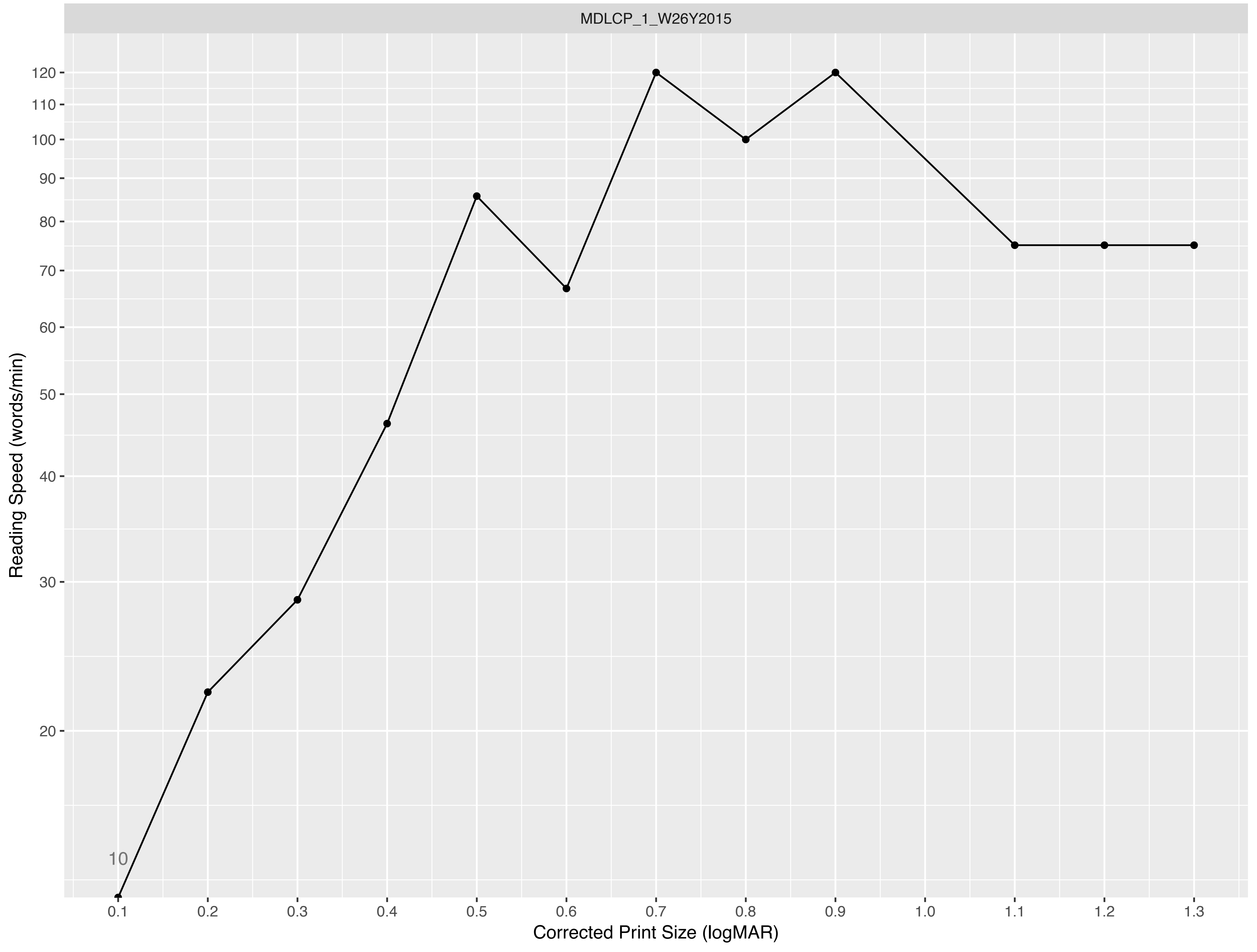
MDLCP_1_W26Y2015
Reading Speed (words/min)
Corrected Print Size (logMAR)
10
20
30
40
50
60
70
80
90
100
110
120
0.1
0.2
0.3
0.4
0.5
0.6
0.7
0.8
0.9
1.0
1.1
1.2
1.3

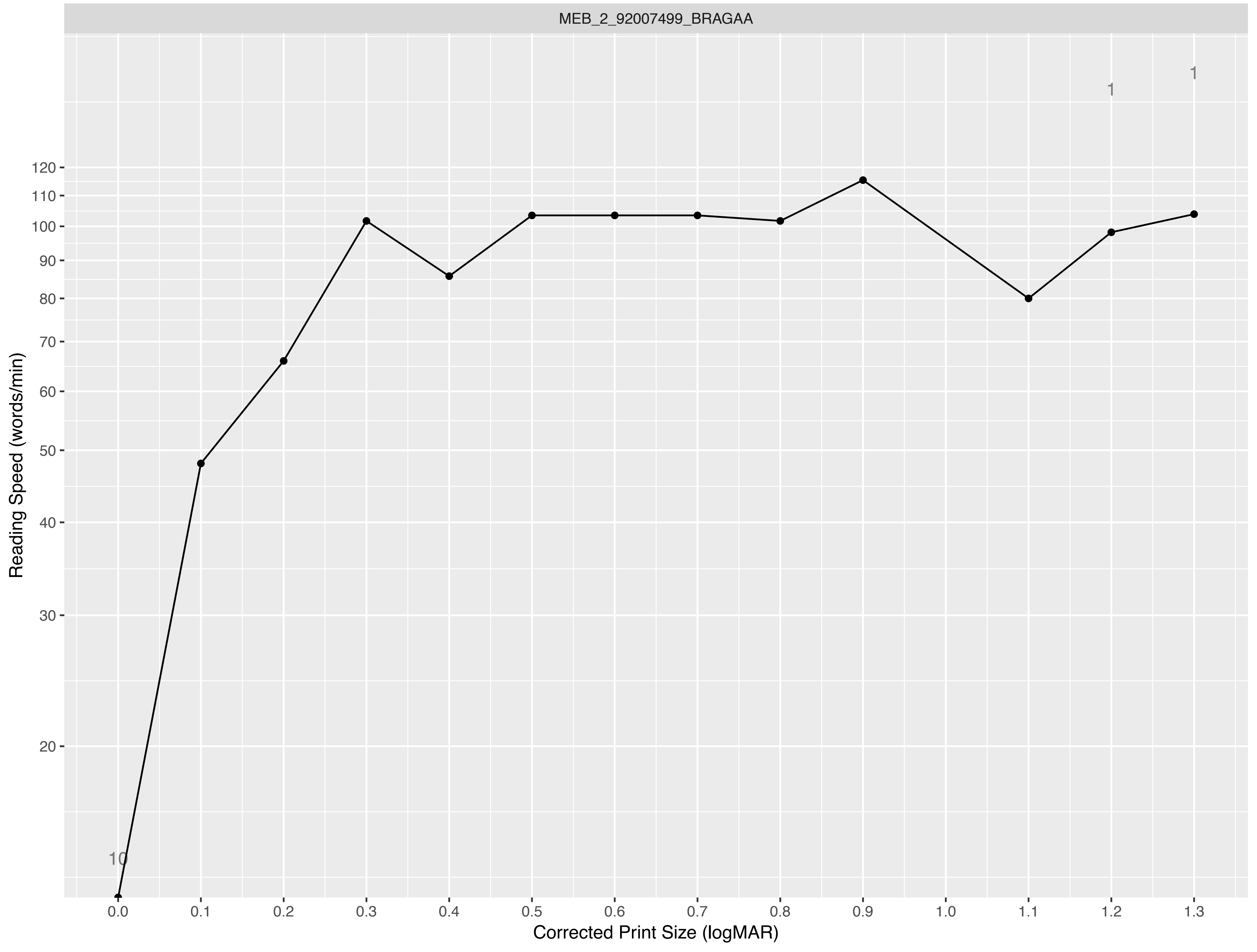
MEB_2_92007499_BRAGAA
Reading Speed (words/min)
120
110
100
90
80
70
60
50
40
30
20
10
1
1
0.0
0.1
0.2
0.3
0.4
0.5
0.6
0.7
0.8
0.9
1.0
1.1
1.2
1.3
Corrected Print Size (logMAR)

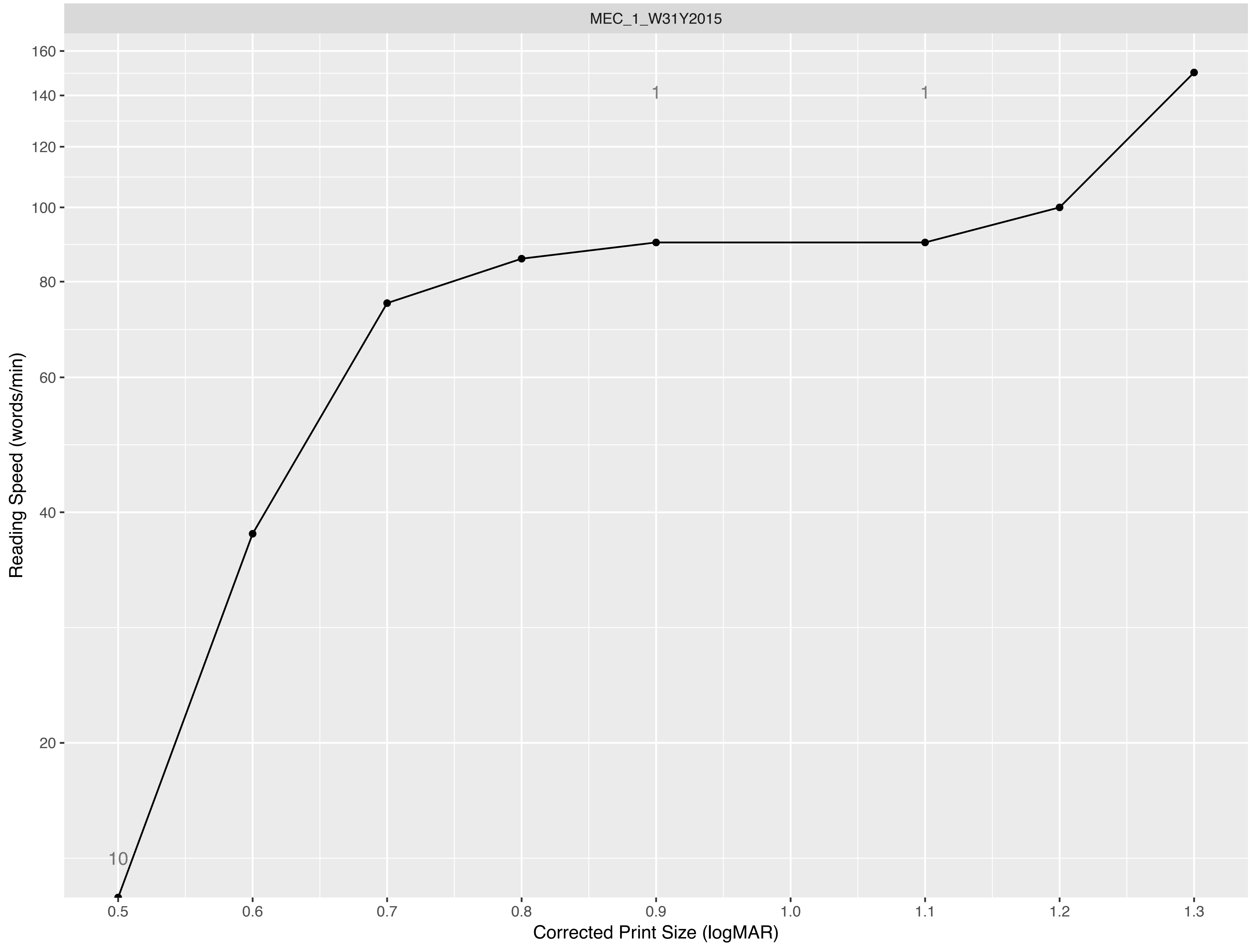

MEC_1_W31Y2015
Reading Speed (words/min)
160
140
120
100
80
60
40
20
1
1
10
0.5
0.6
0.7
0.8
0.9
1.0
1.1
1.2
1.3
Corrected Print Size (logMAR)

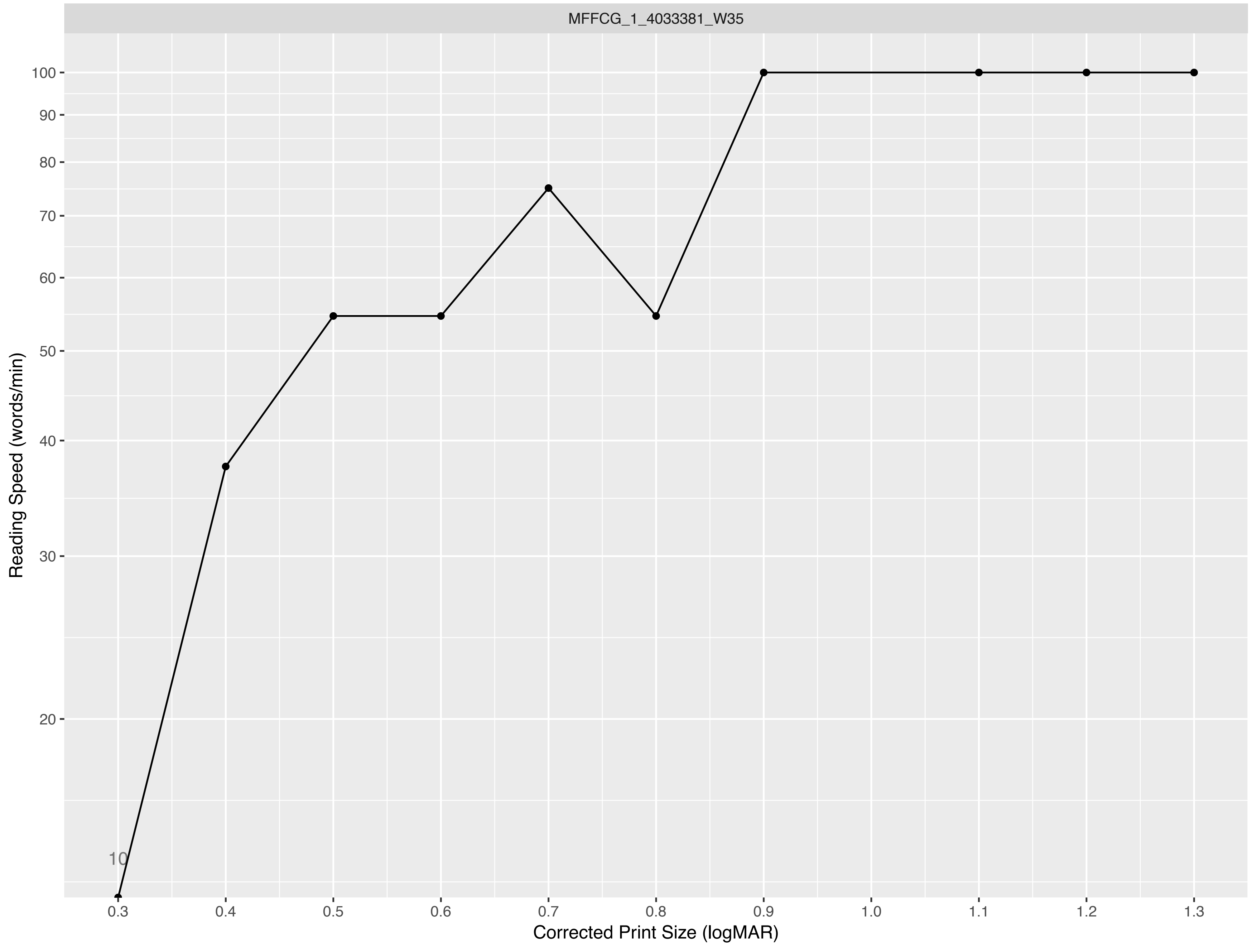

MFFCG_1_4033381_W35
Reading Speed (words/min)
100
90
80
70
60
50
40
30
20
10
0.3
0.4
0.5
0.6
0.7
0.8
0.9
1.0
1.1
1.2
1.3
Corrected Print Size (logMAR)

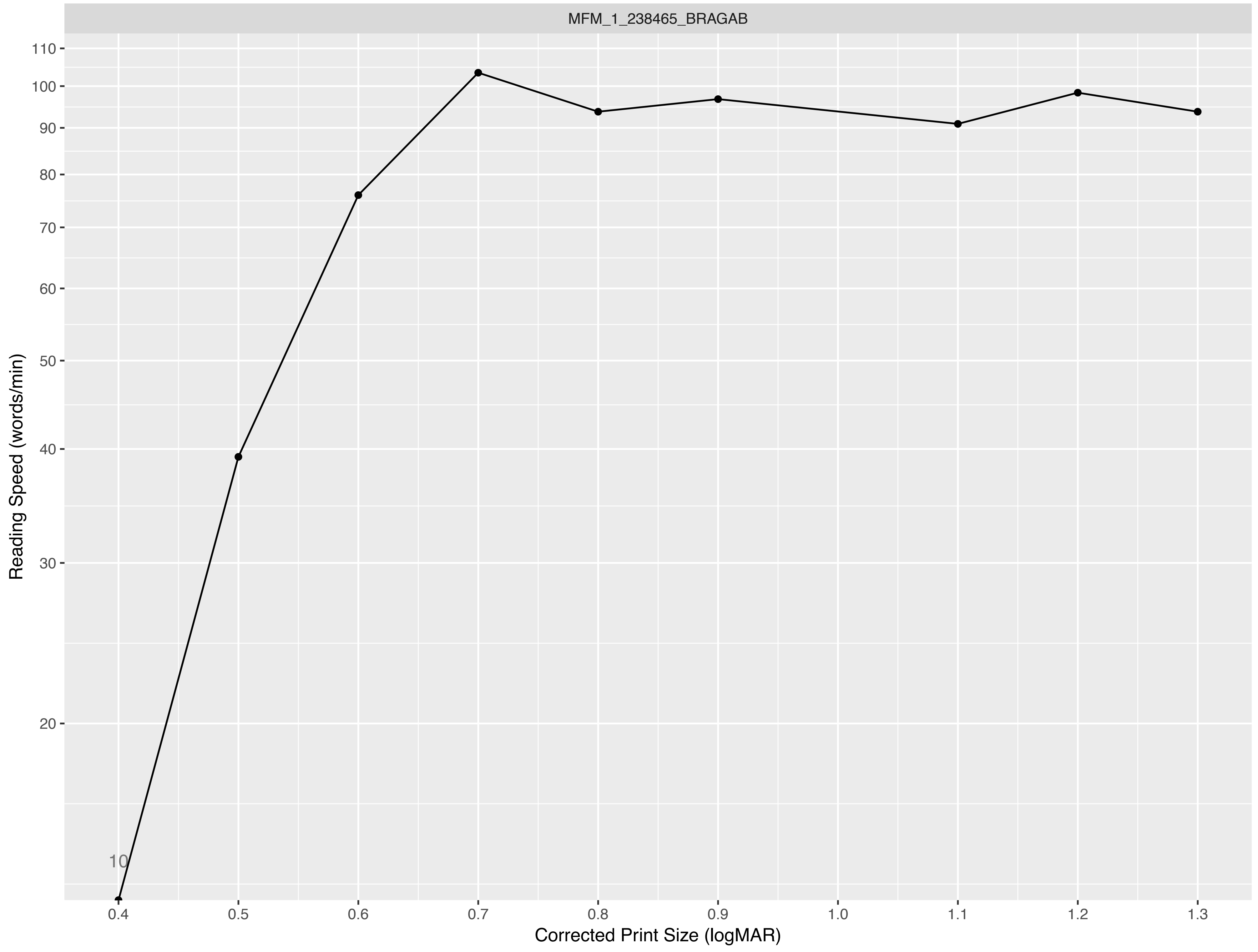
MFM_1_238465_BRAGAB
Reading Speed (words/min)
110
100
90
80
70
60
50
40
30
20
10
0.4
0.5
0.6
0.7
0.8
0.9
1.0
1.1
1.2
1.3
Corrected Print Size (logMAR)

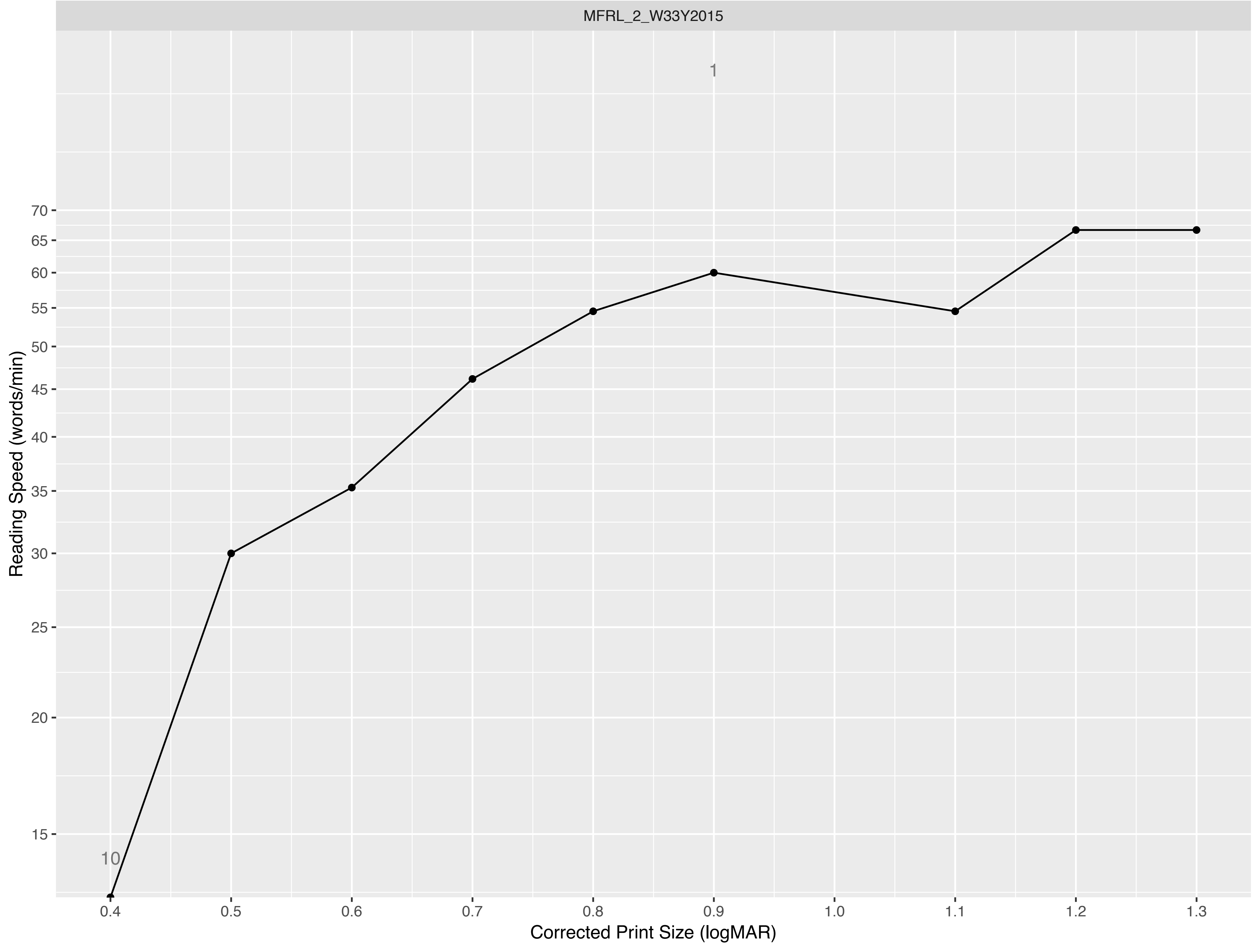

MFRL_2_W33Y2015
1
10
70
65
60
55
50
45
40
35
30
25
20
15
Reading Speed (words/min)
0.4
0.5
0.6
0.7
0.8
0.9
1.0
1.1
1.2
1.3
Corrected Print Size (logMAR)

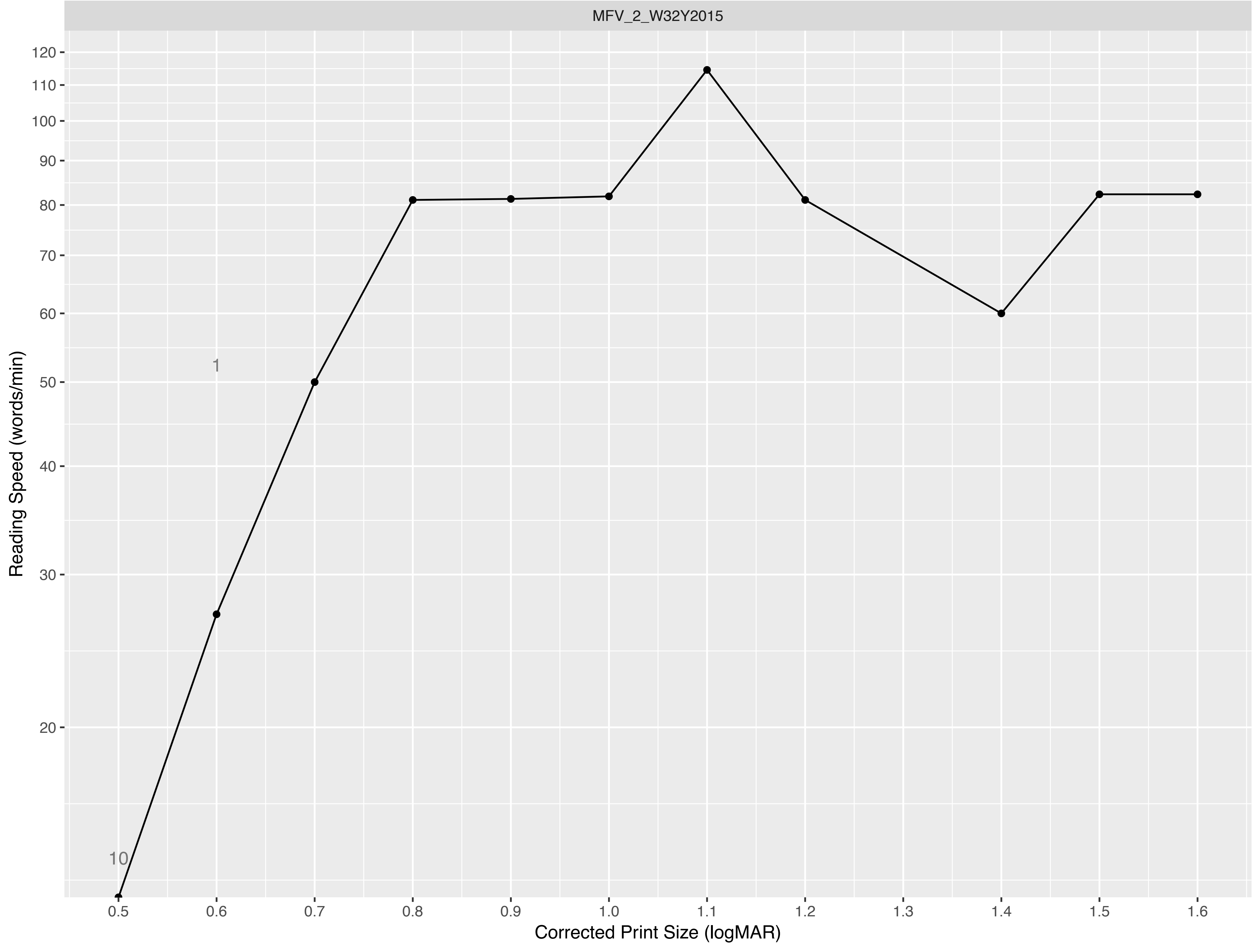

MFV_2_W32Y2015
Reading Speed (words/min)
120
110
100
90
80
70
60
50
40
30
20
1
10
0.5
0.6
0.7
0.8
0.9
1.0
1.1
1.2
1.3
1.4
1.5
1.6
Corrected Print Size (logMAR)

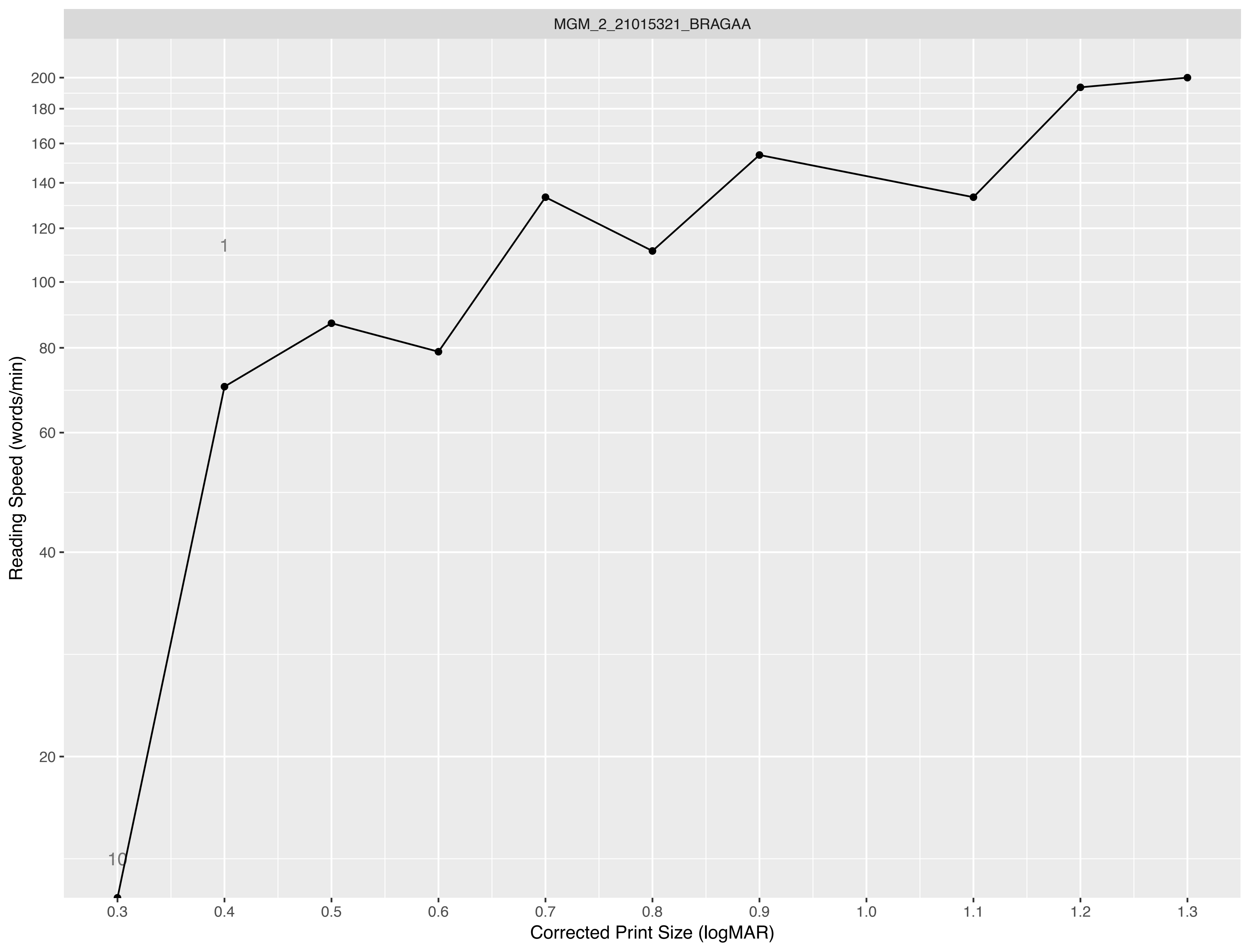

MGM_2_21015321_BRAGAA
Reading Speed (words/min)
200
180
160
140
120
100
80
60
40
20
1
10
0.3
0.4
0.5
0.6
0.7
0.8
0.9
1.0
1.1
1.2
1.3
Corrected Print Size (logMAR)

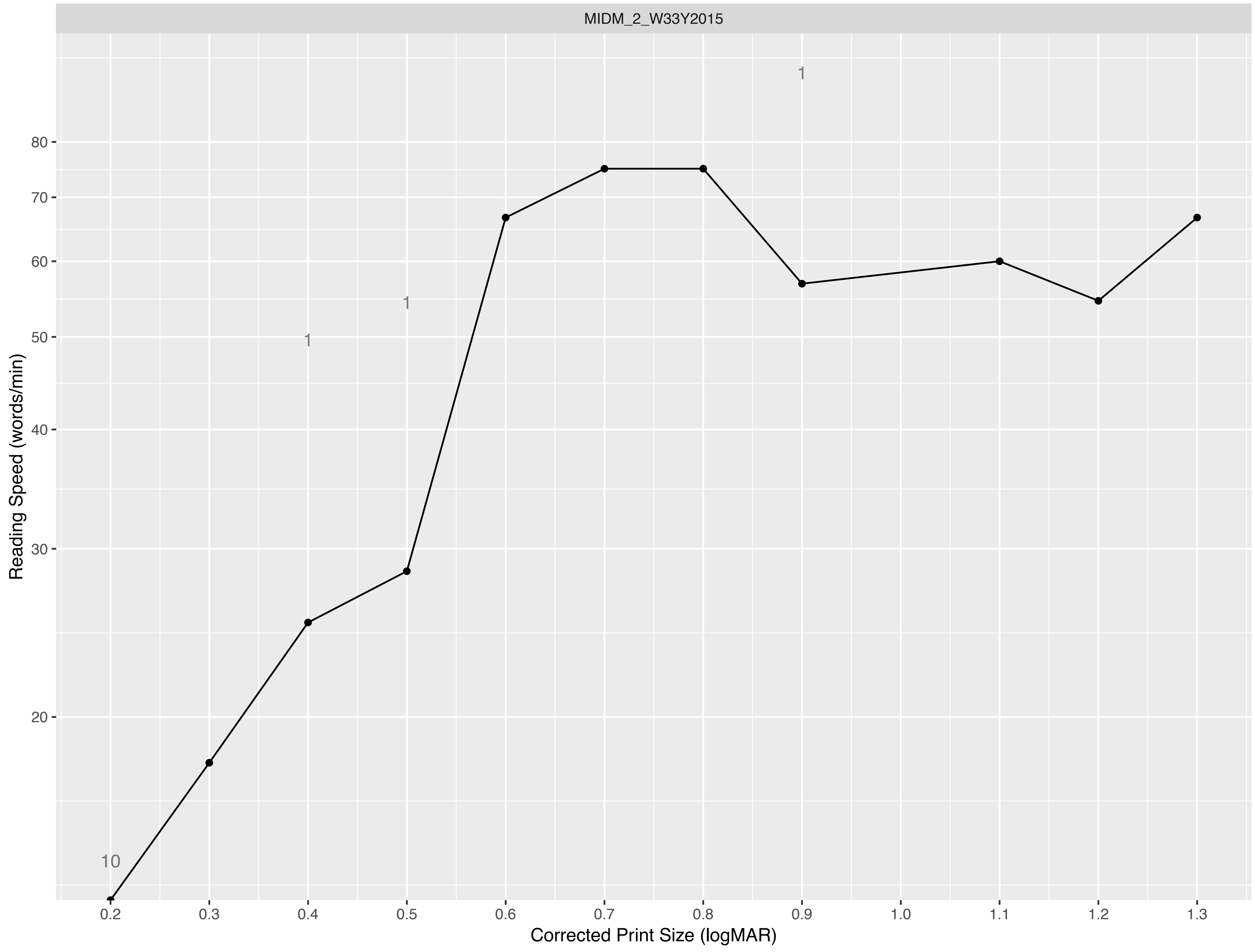
MIDM_2_W33Y2015
Reading Speed (words/min)
80
70
60
50
40
30
20
10
1
1
1
1
0.2
0.3
0.4
0.5
0.6
0.7
0.8
0.9
1.0
1.1
1.2
1.3
Corrected Print Size (logMAR)

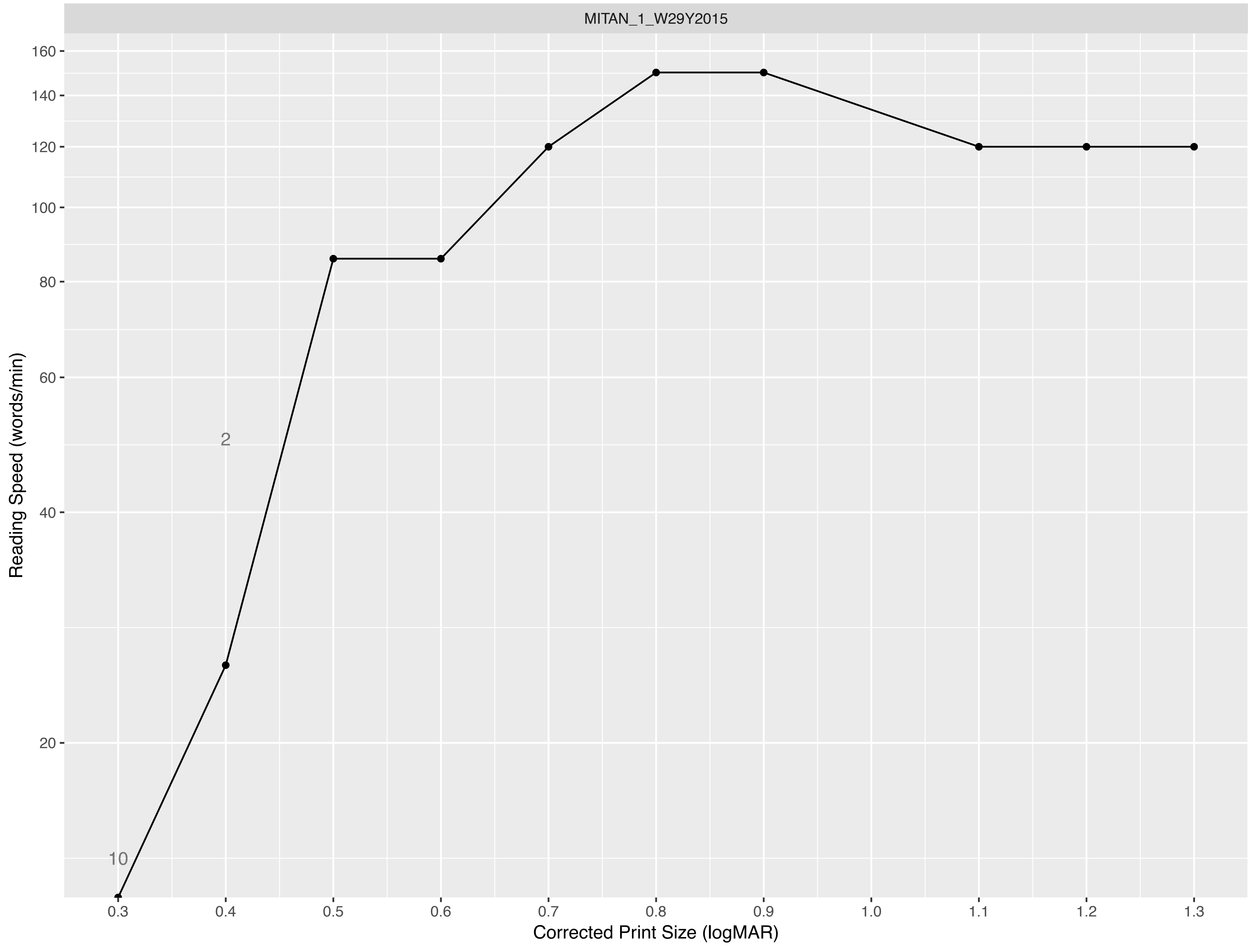

MITAN_1_W29Y2015
Reading Speed (words/min)
160
140
120
100
80
60
40
20
2
10
0.3
0.4
0.5
0.6
0.7
0.8
0.9
1.0
1.1
1.2
1.3
Corrected Print Size (logMAR)

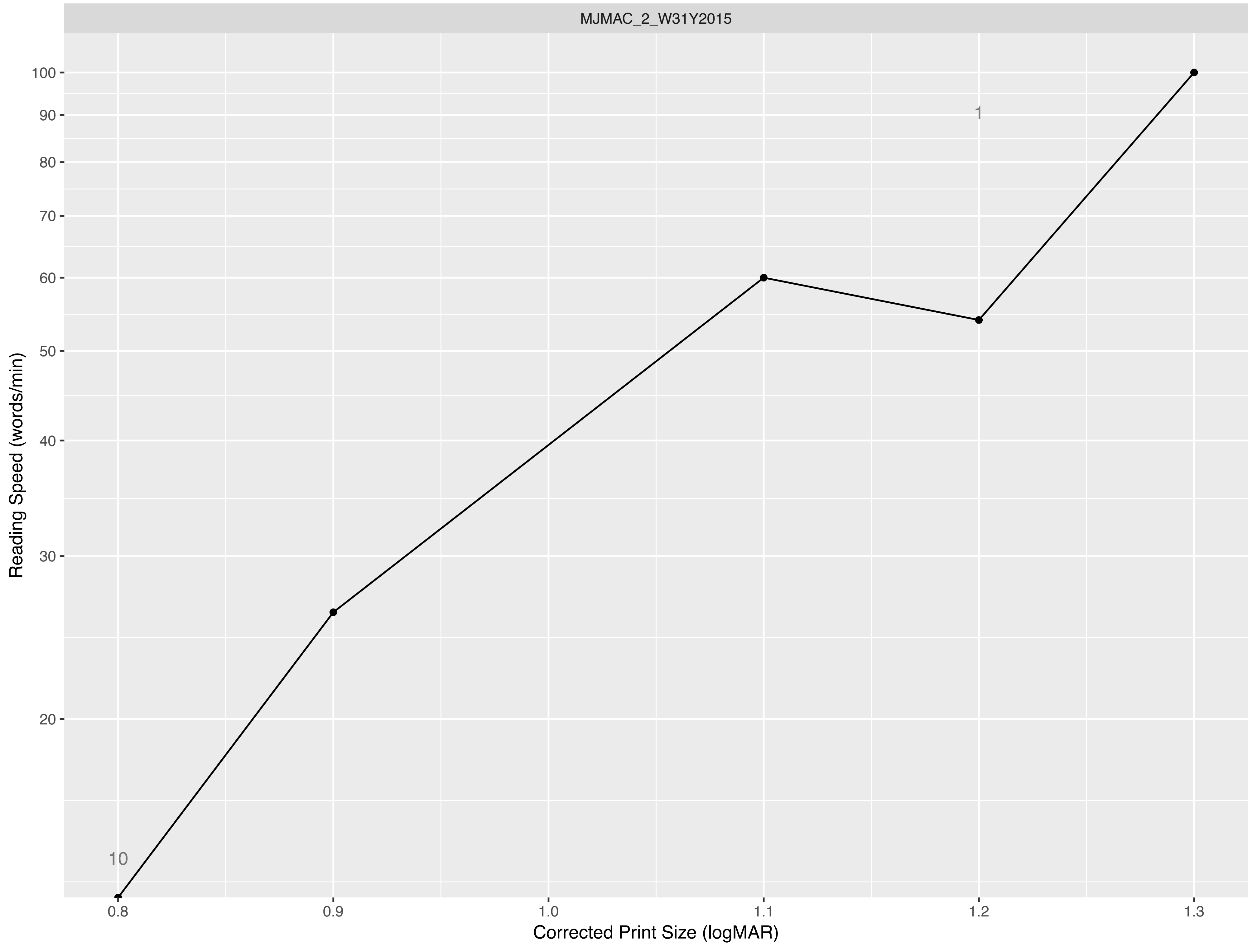

MJMAC_2_W31Y2015
Reading Speed (words/min)
100
90
80
70
60
50
40
30
20
1
10
0.8
0.9
1.0
1.1
1.2
1.3
Corrected Print Size (logMAR)

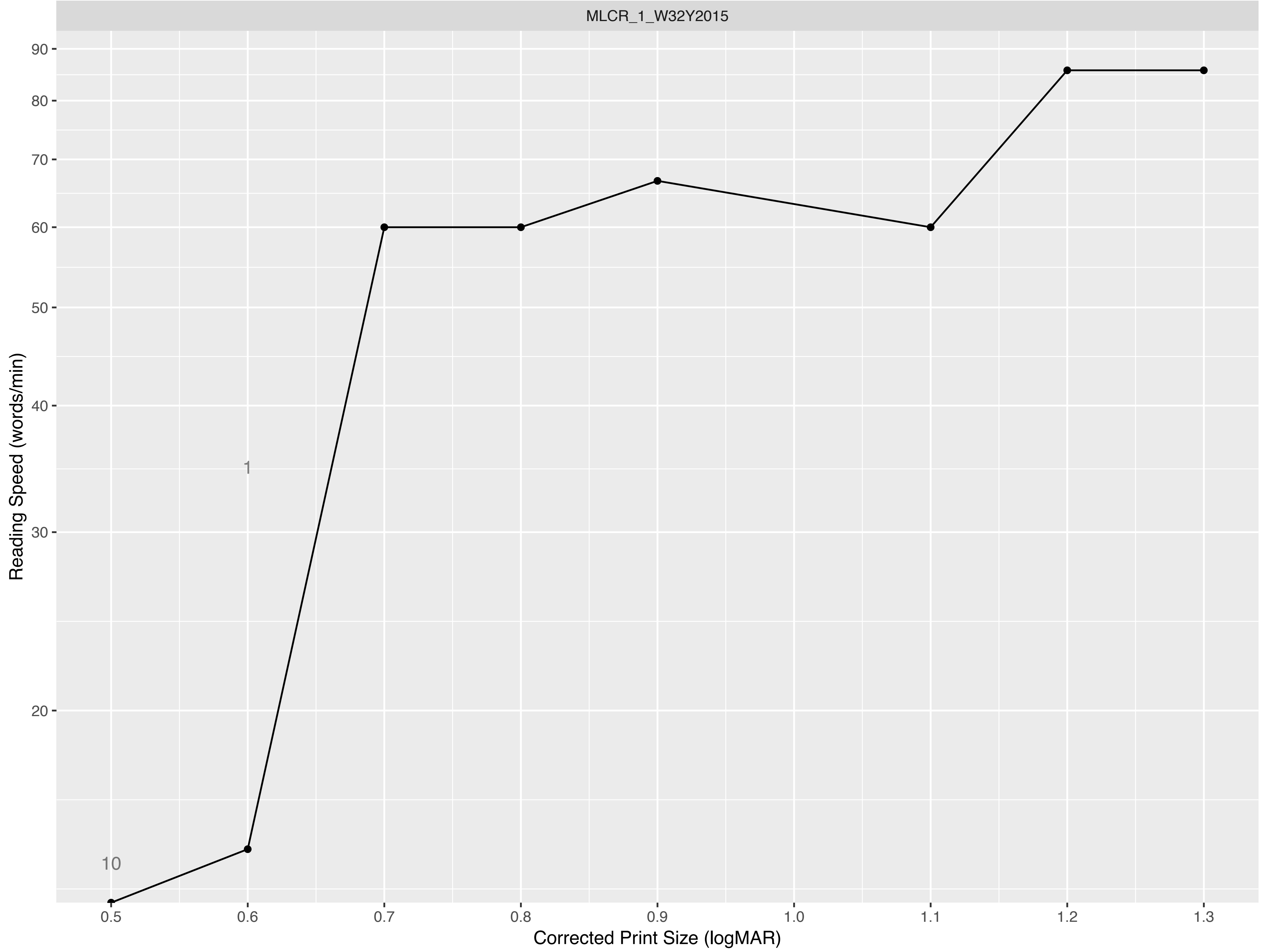

MLCR_1_W32Y2015
Reading Speed (words/min)
Corrected Print Size (logMAR)
90
80
70
60
50
40
30
20
1
10
0.5
0.6
0.7
0.8
0.9
1.0
1.1
1.2
1.3

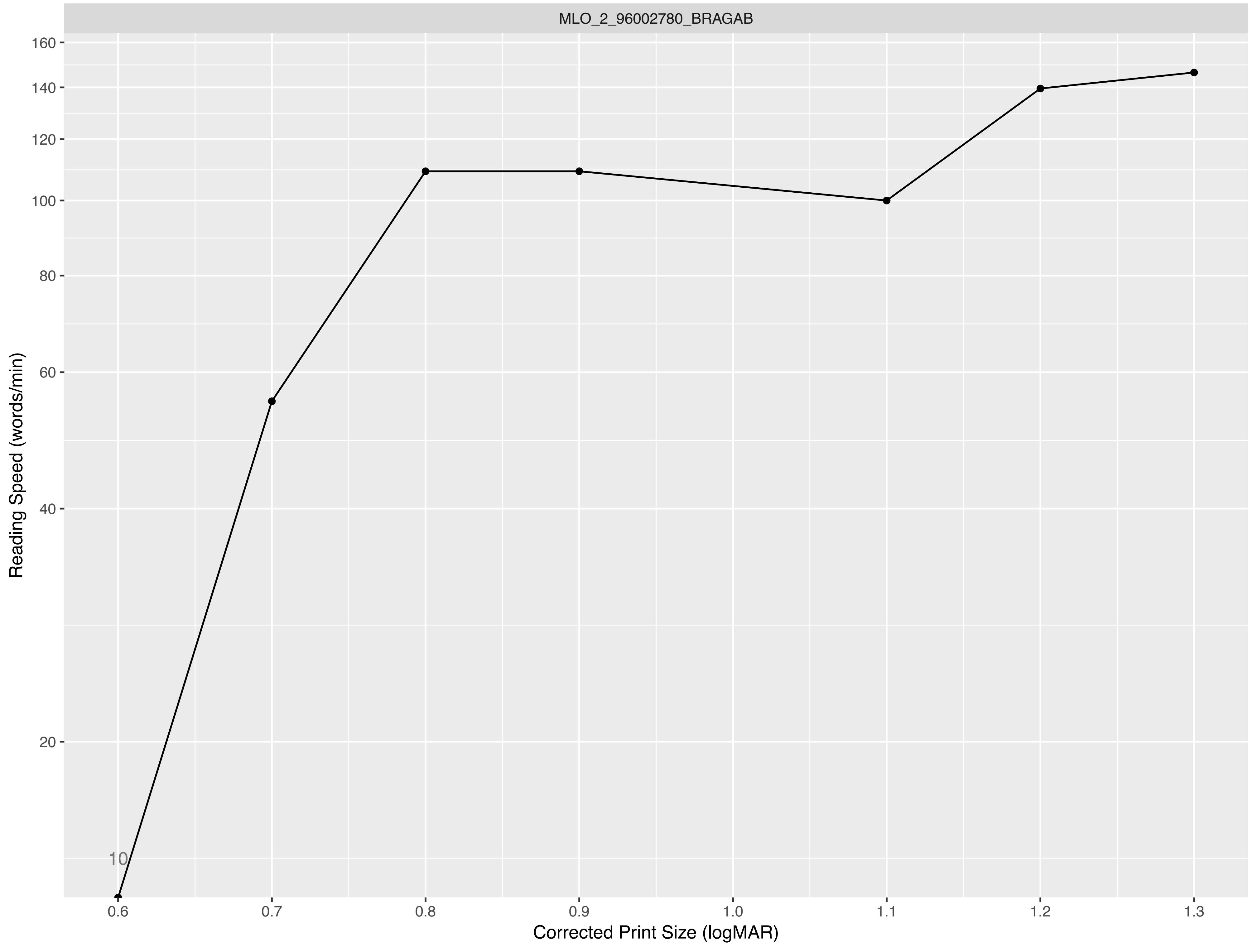

MLO_2_96002780_BRAGAB
160
140
120
100
80
60
40
20
10
0.6
0.7
0.8
0.9
1.0
1.1
1.2
1.3
Reading Speed (words/min)
Corrected Print Size (logMAR)

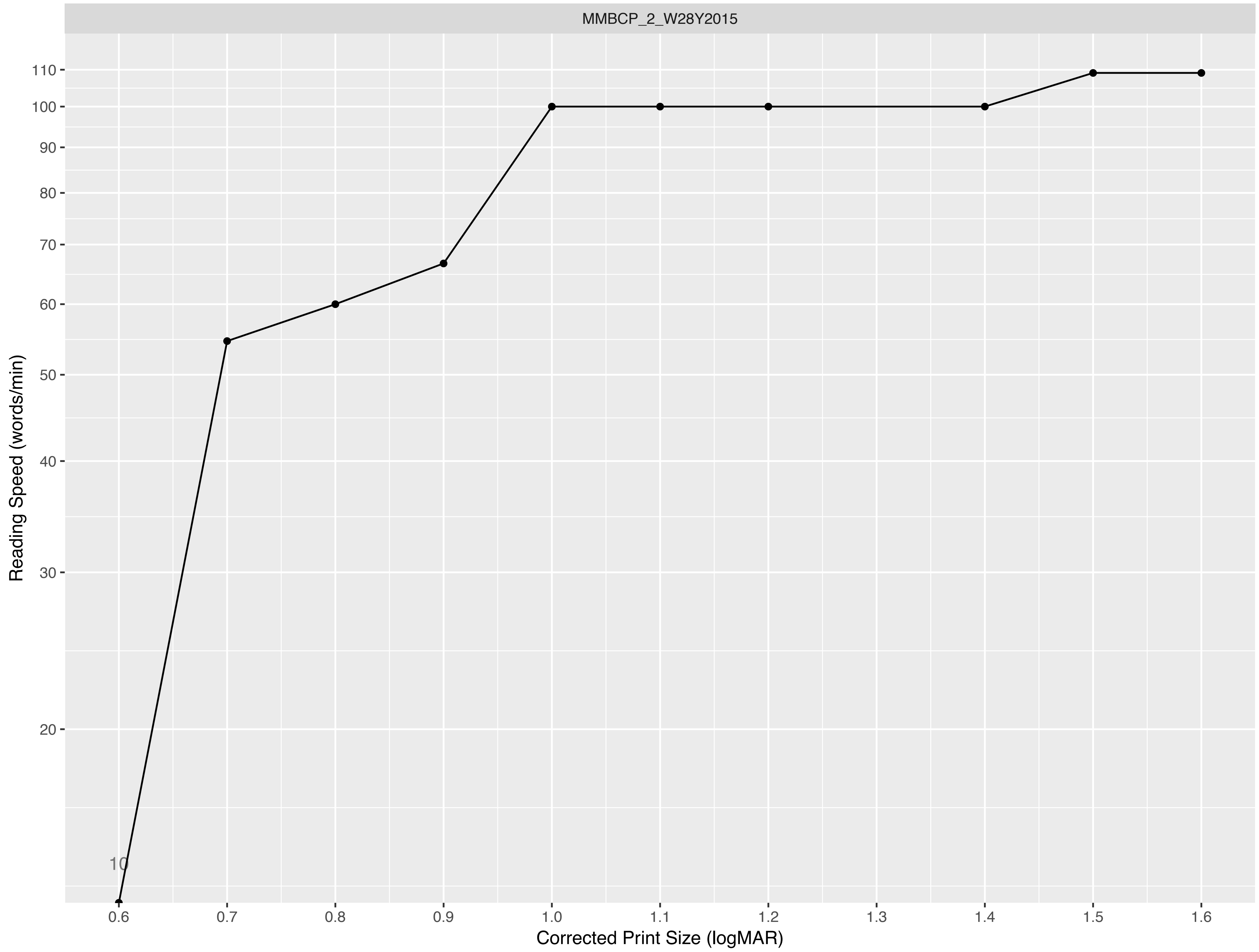

MMBCP_2_W28Y2015
Reading Speed (words/min)
110
100
90
80
70
60
50
40
30
20
10
0.6
0.7
0.8
0.9
1.0
1.1
1.2
1.3
1.4
1.5
1.6
Corrected Print Size (logMAR)

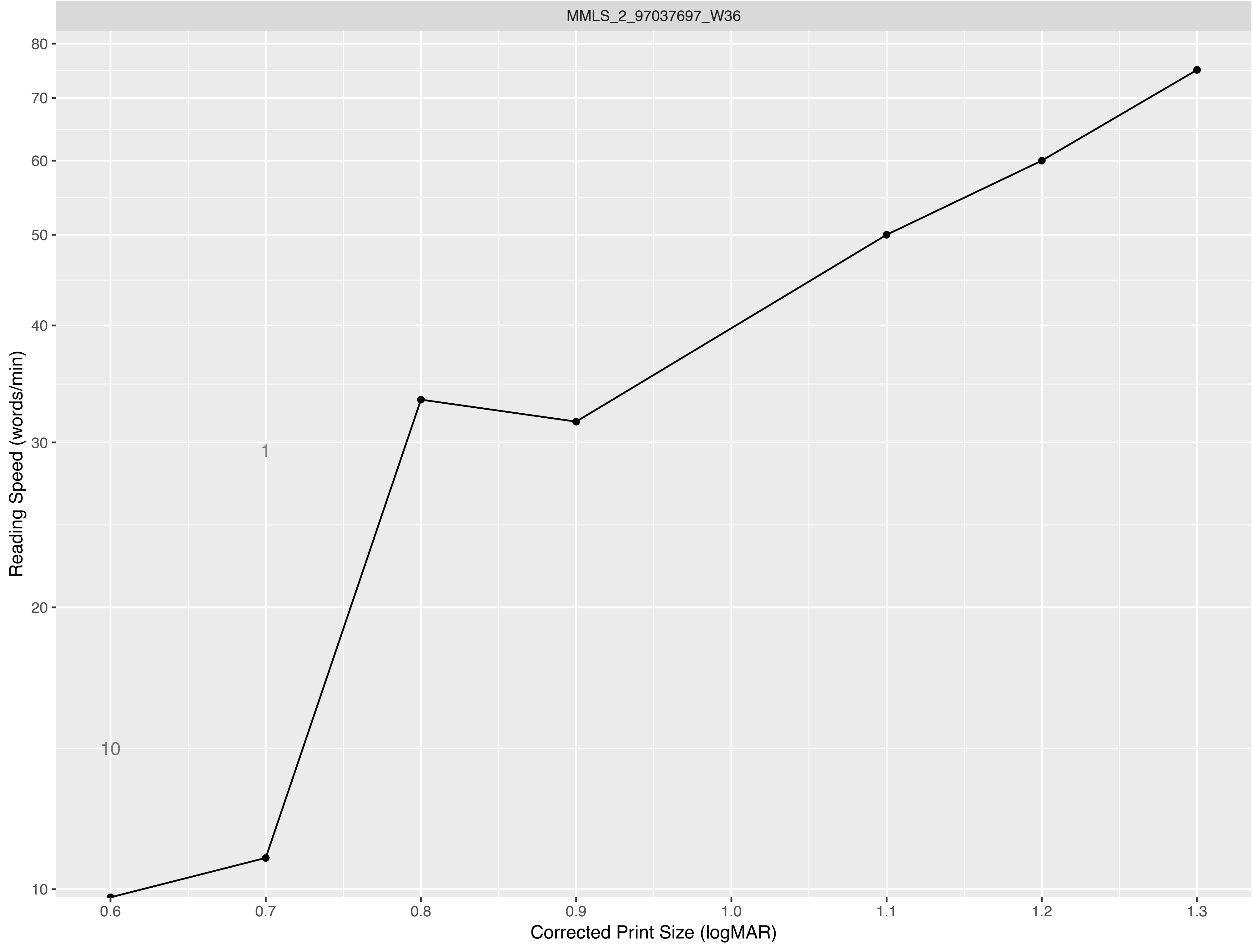
MMLS_2_97037697_W36
Reading Speed (words/min)
80
70
60
50
40
30
20
10
1
10
0.6
0.7
0.8
0.9
1.0
1.1
1.2
1.3
Corrected Print Size (logMAR)

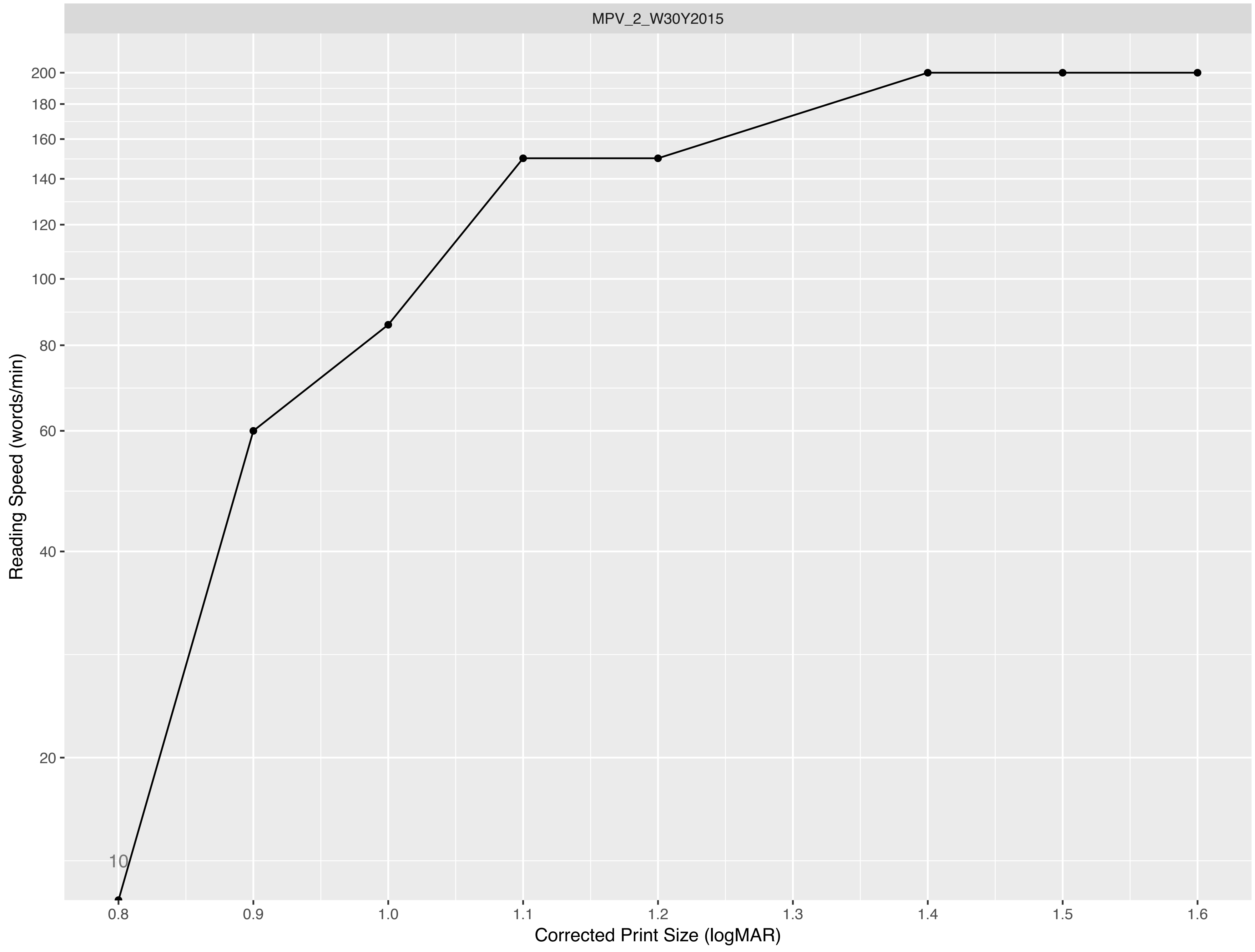

MPV_2_W30Y2015
Reading Speed (words/min)
200
180
160
140
120
100
80
60
40
20
10
0.8
0.9
1.0
1.1
1.2
1.3
1.4
1.5
1.6
Corrected Print Size (logMAR)

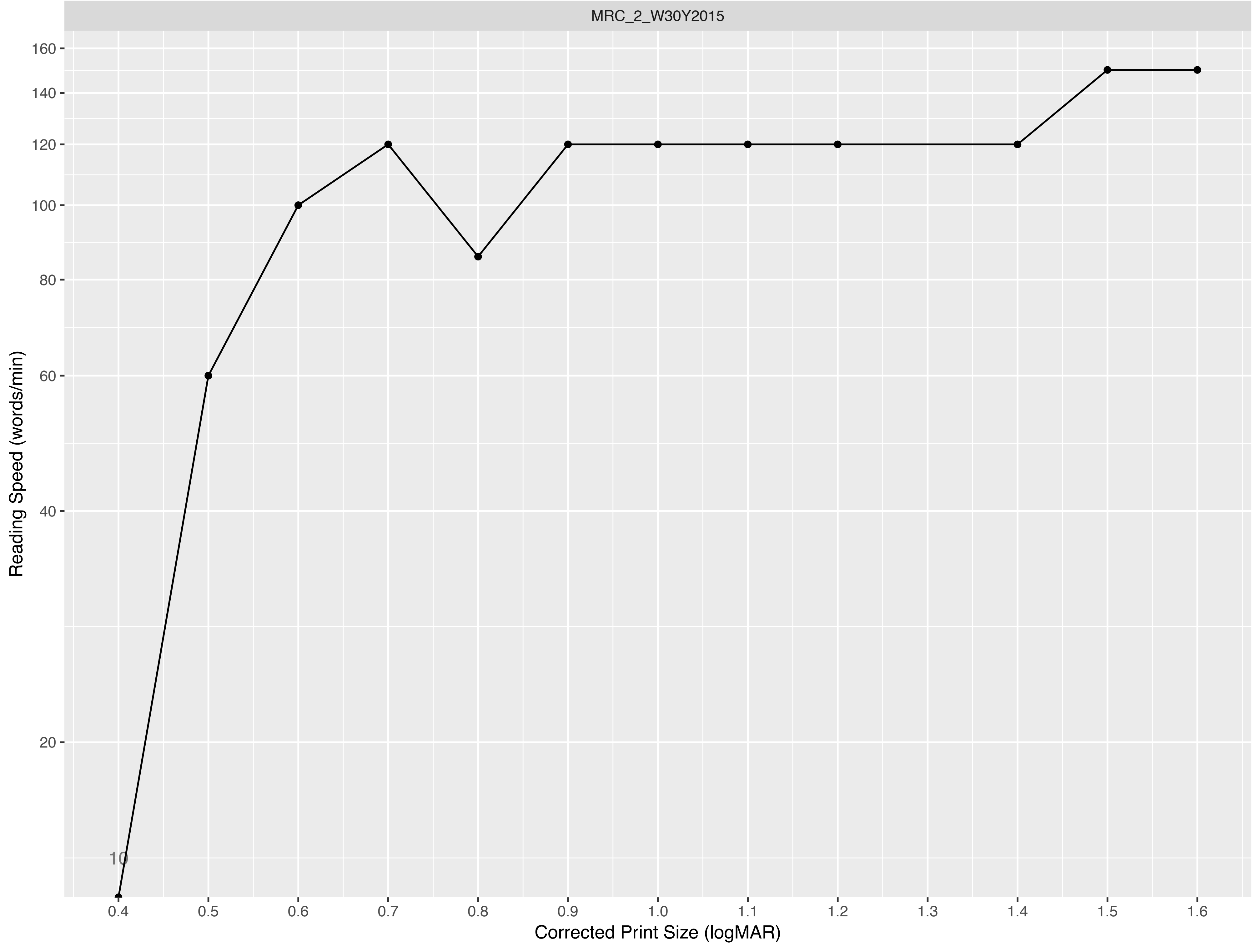
MRC_2_W30Y2015
Reading Speed (words/min)
160
140
120
100
80
60
40
20
10
0.4
0.5
0.6
0.7
0.8
0.9
1.0
1.1
1.2
1.3
1.4
1.5
1.6
Corrected Print Size (logMAR)

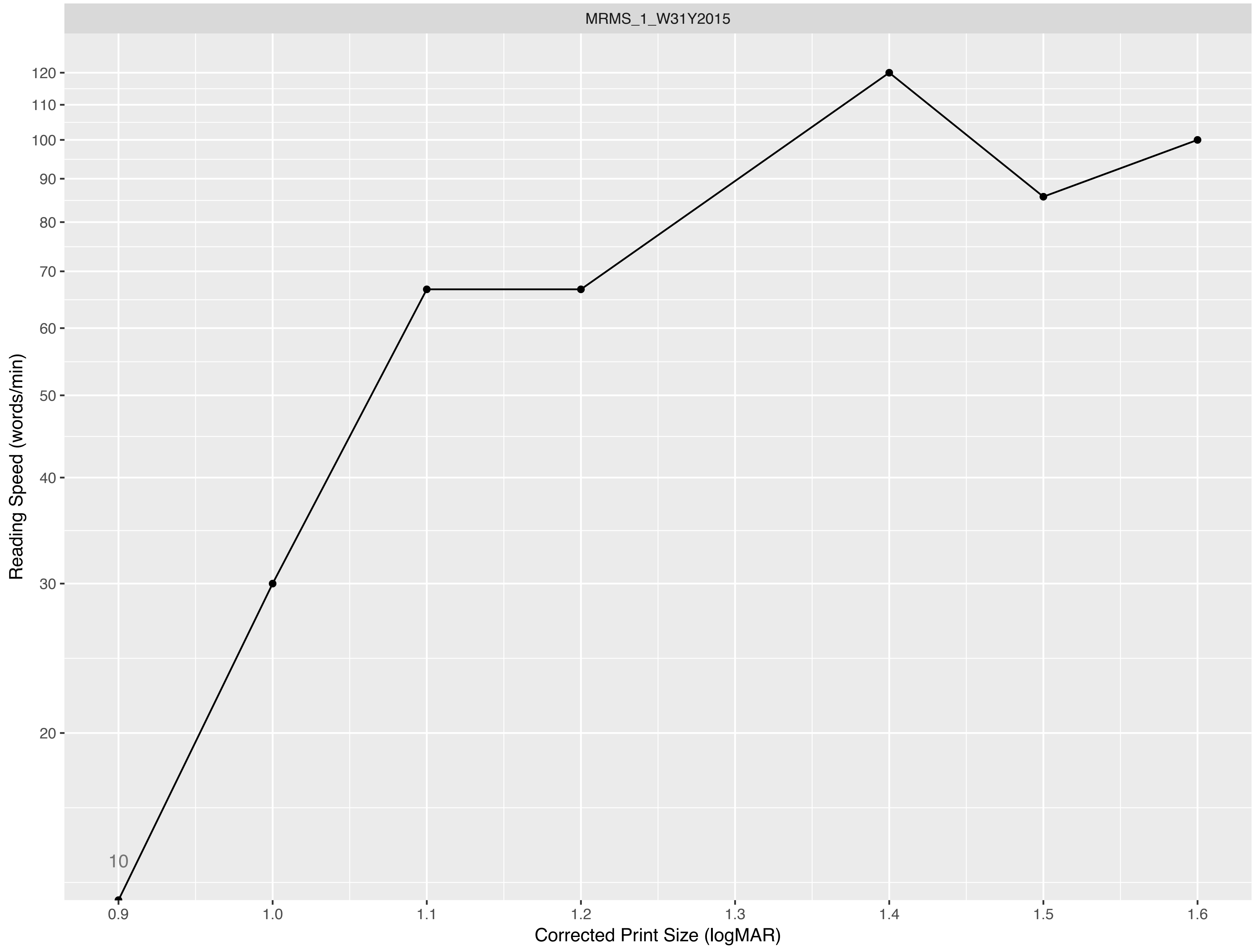

MRMS_1_W31Y2015
Reading Speed (words/min)
120
110
100
90
80
70
60
50
40
30
20
10
0.9
1.0
1.1
1.2
1.3
1.4
1.5
1.6
Corrected Print Size (logMAR)

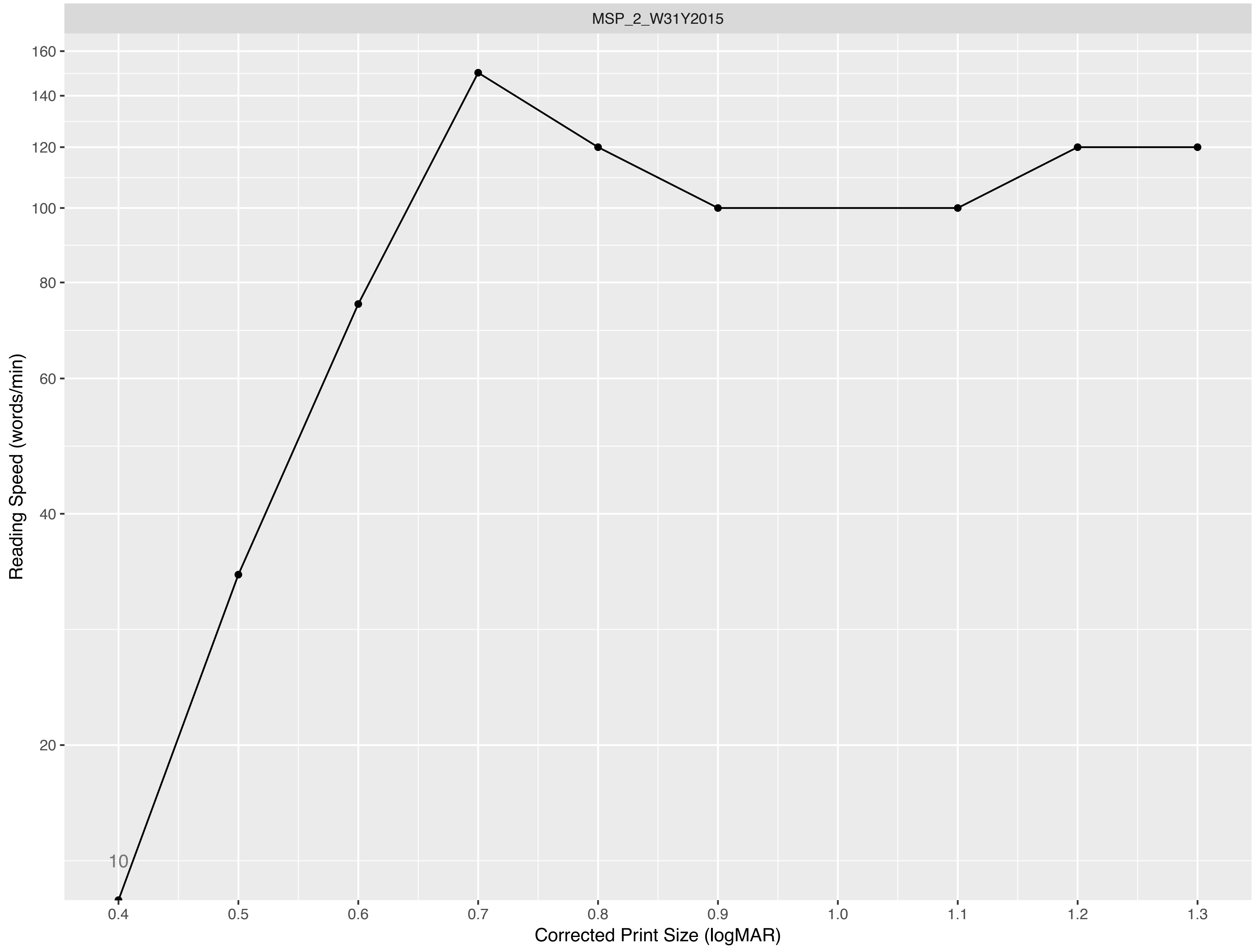

MSP_2_W31Y2015
Reading Speed (words/min)
160
140
120
100
80
60
40
20
10
0.4
0.5
0.6
0.7
0.8
0.9
1.0
1.1
1.2
1.3
Corrected Print Size (logMAR)

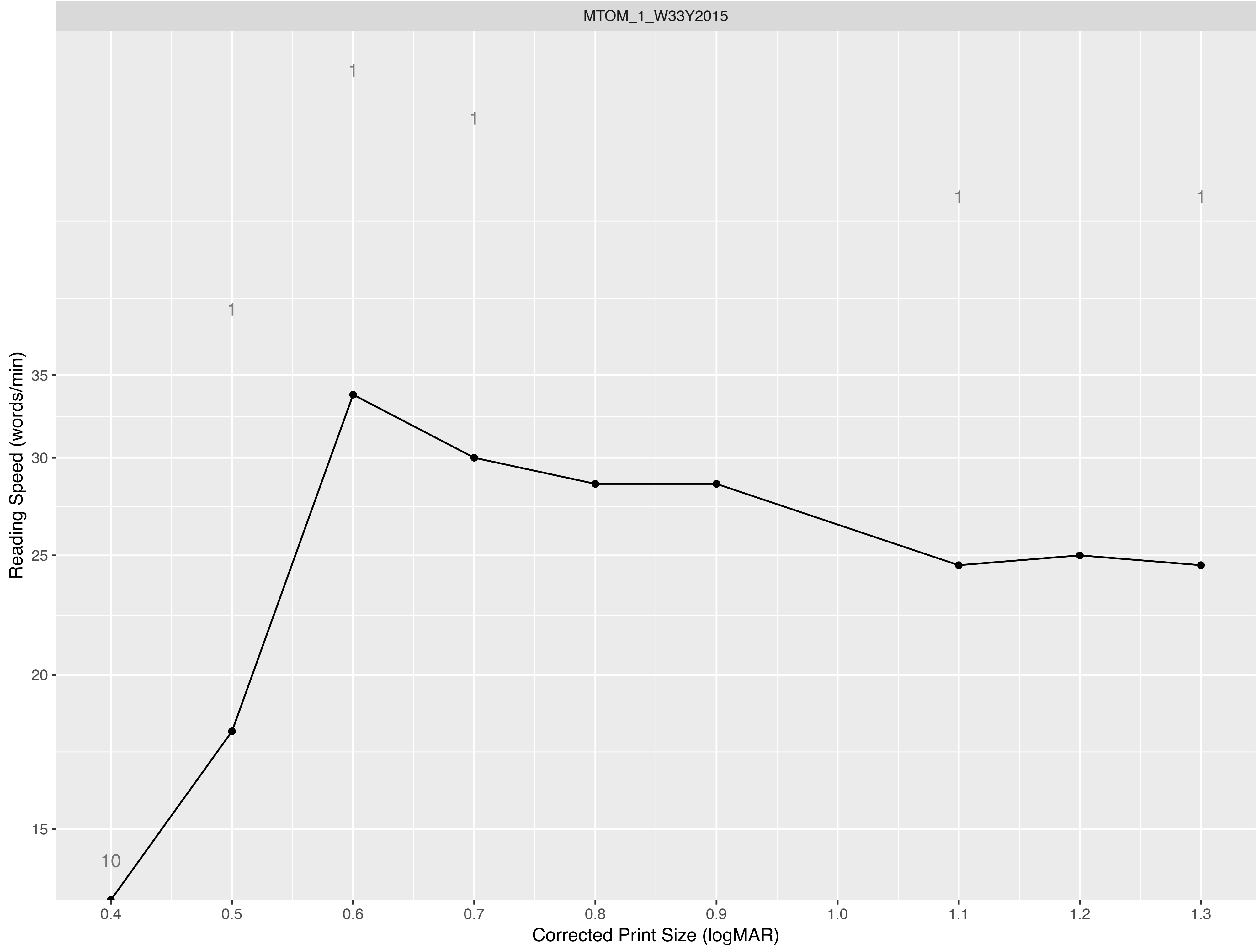

MTOM_1_W33Y2015
Reading Speed (words/min)
Corrected Print Size (logMAR)
35
30
25
20
15
0.4
0.5
0.6
0.7
0.8
0.9
1.0
1.1
1.2
1.3
1
1
1
1
1
10

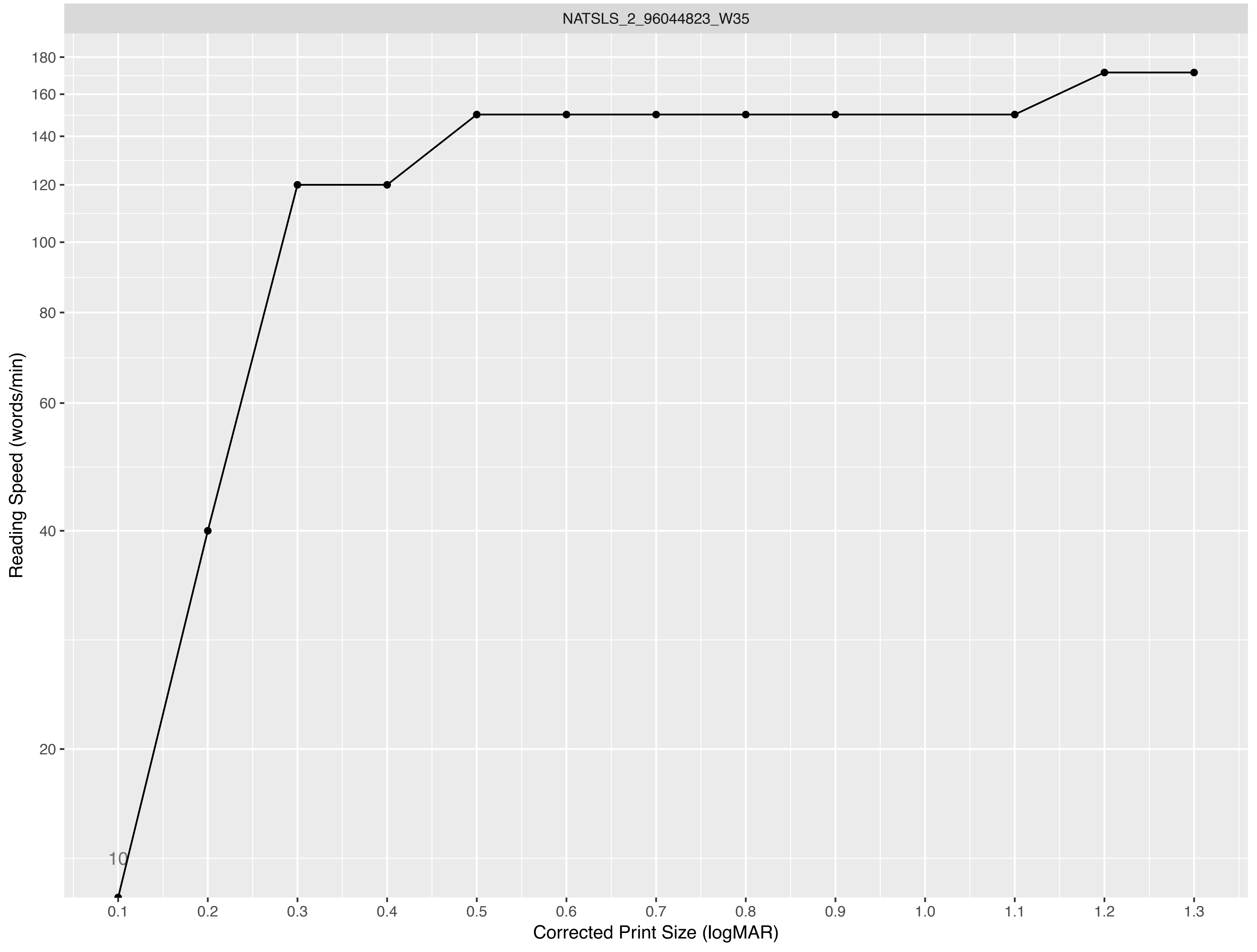

NATSLS_2_96044823_W35
Reading Speed (words/min)
180
160
140
120
100
80
60
40
20
10
0.1
0.2
0.3
0.4
0.5
0.6
0.7
0.8
0.9
1.0
1.1
1.2
1.3
Corrected Print Size (logMAR)

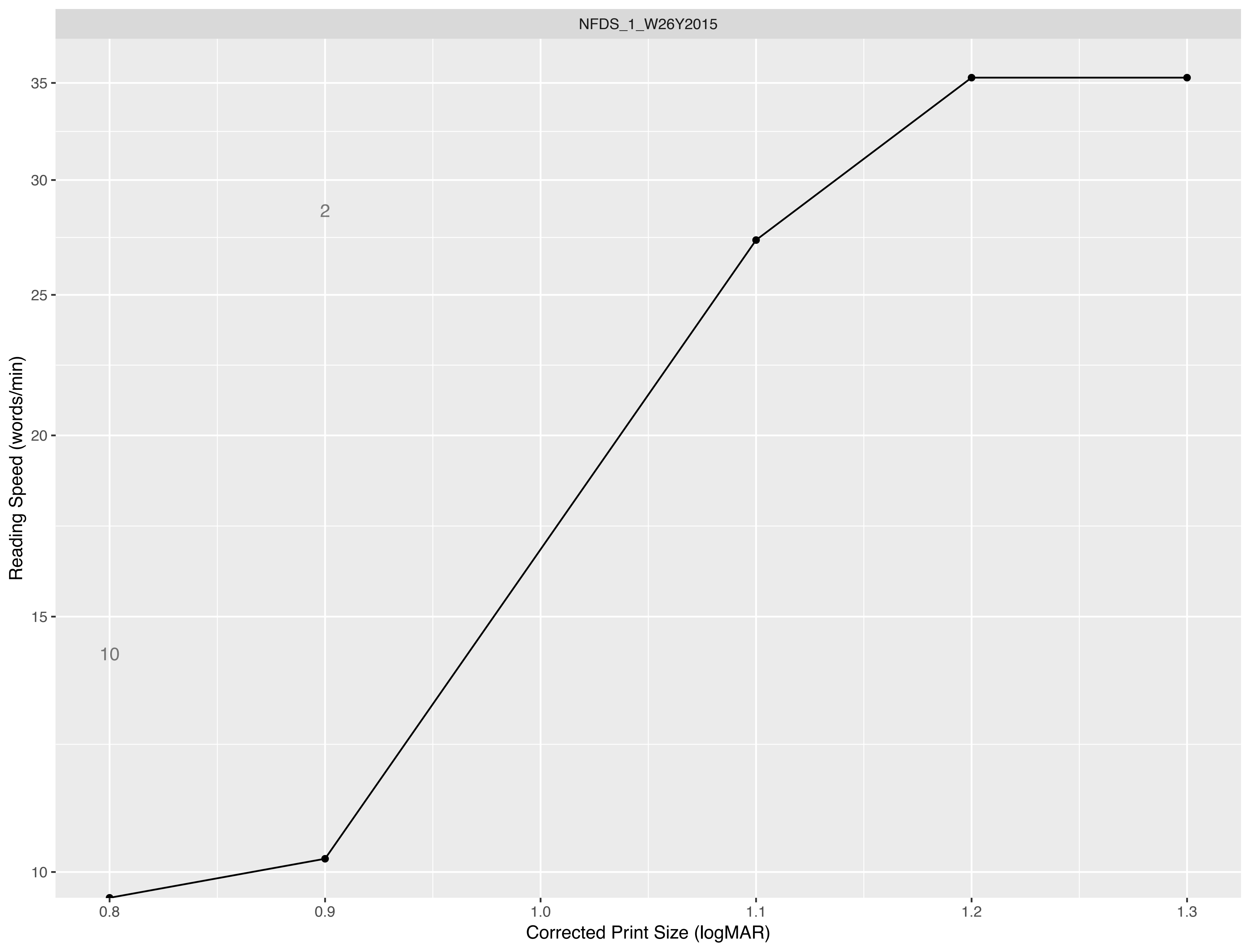

NFDS_1_W26Y2015
Reading Speed (words/min)
35
30
25
20
15
10
2
10
0.8
0.9
1.0
1.1
1.2
1.3
Corrected Print Size (logMAR)

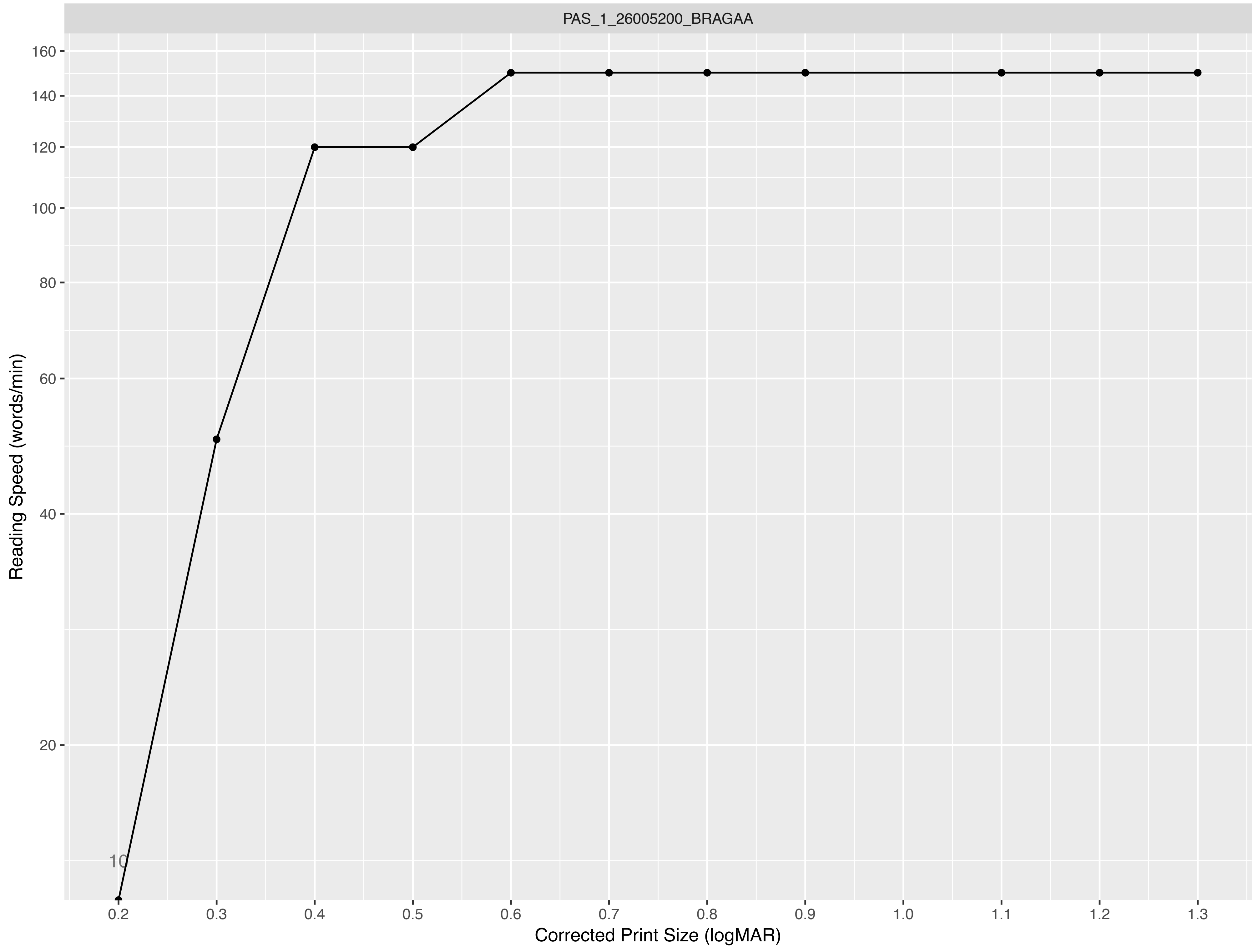
PAS_1_26005200_BRAGAA
Reading Speed (words/min)
160
140
120
100
80
60
40
20
10
0.2
0.3
0.4
0.5
0.6
0.7
0.8
0.9
1.0
1.1
1.2
1.3
Corrected Print Size (logMAR)

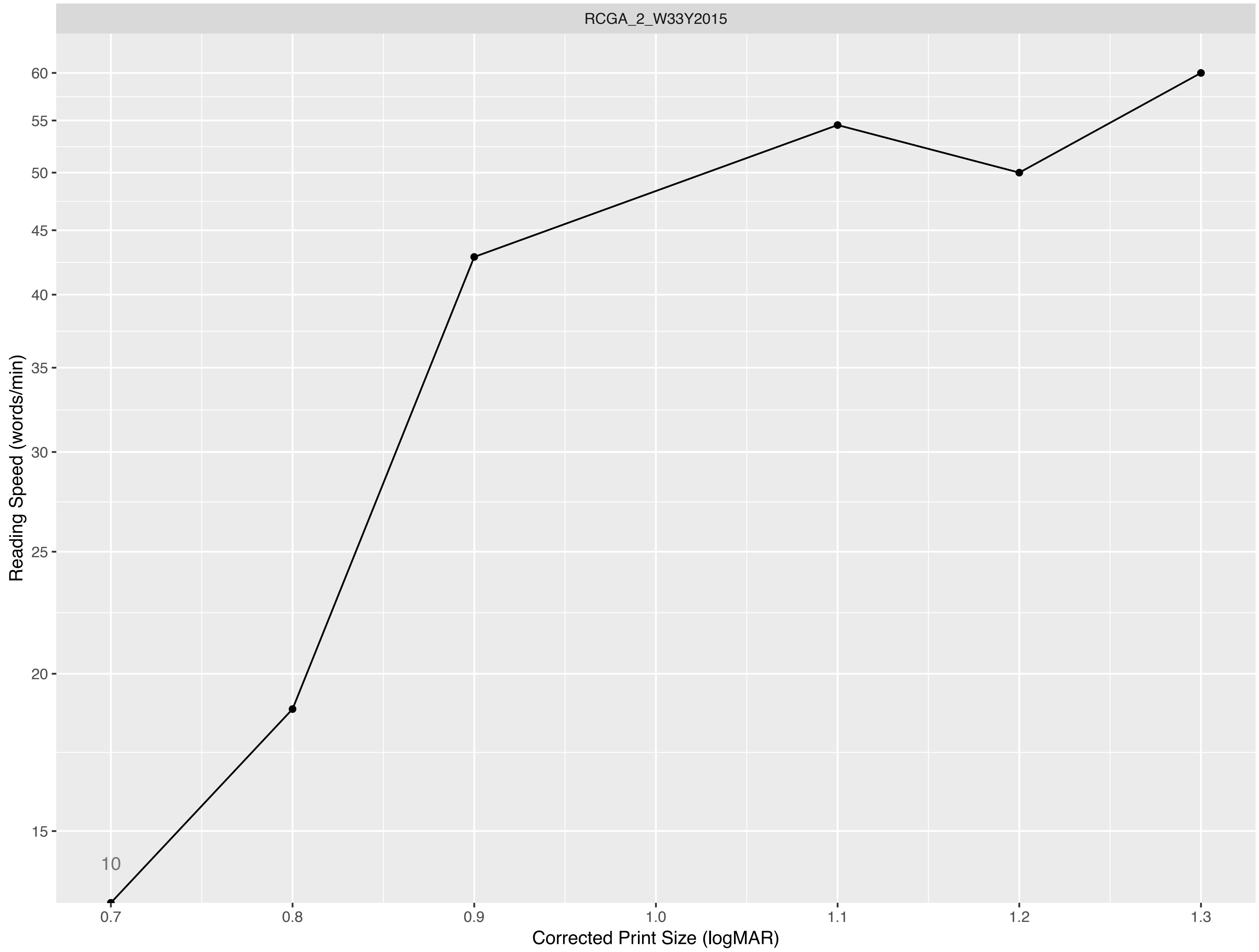

RCGA_2_W33Y2015
Reading Speed (words/min)
60
55
50
45
40
35
30
25
20
15
10
0.7
0.8
0.9
1.0
1.1
1.2
1.3
Corrected Print Size (logMAR)

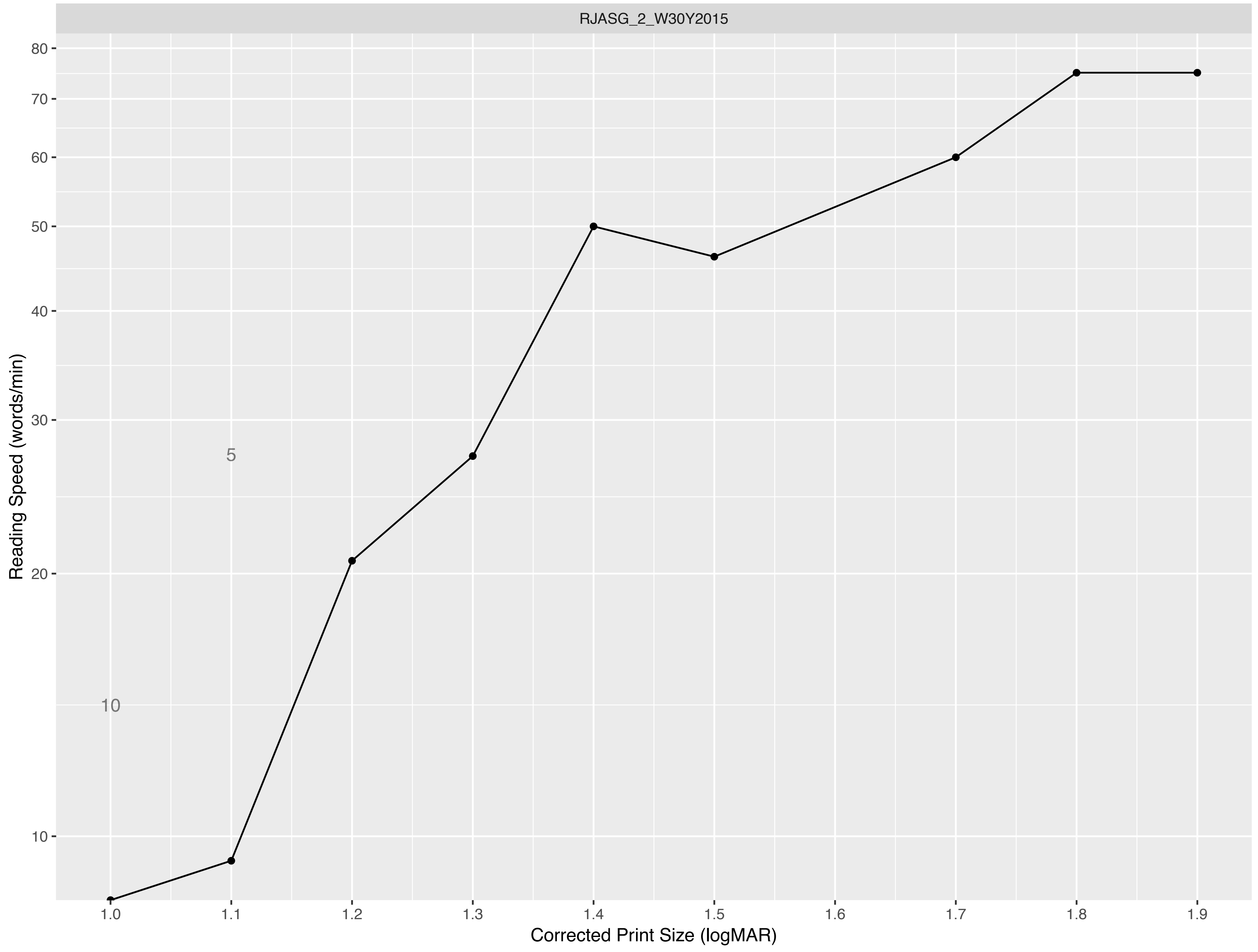

RJASG_2_W30Y2015
Reading Speed (words/min)
80
70
60
50
40
30
20
10
5
10
1.0
1.1
1.2
1.3
1.4
1.5
1.6
1.7
1.8
1.9
Corrected Print Size (logMAR)

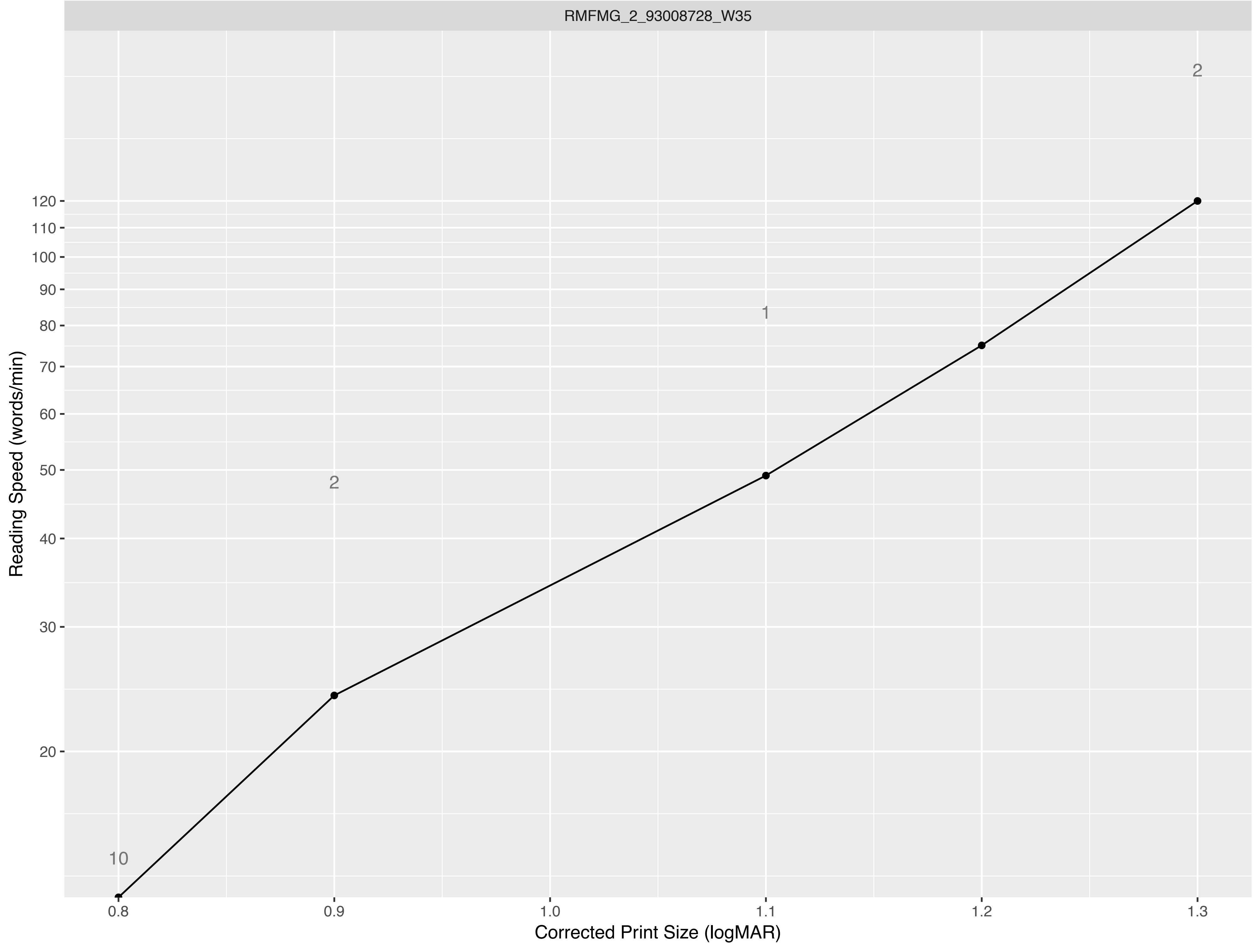
RMFMG_2_93008728_W35
Reading Speed (words/min)
120
110
100
90
80
70
60
50
40
30
20
2
1
2
10
0.8
0.9
1.0
1.1
1.2
1.3
Corrected Print Size (logMAR)

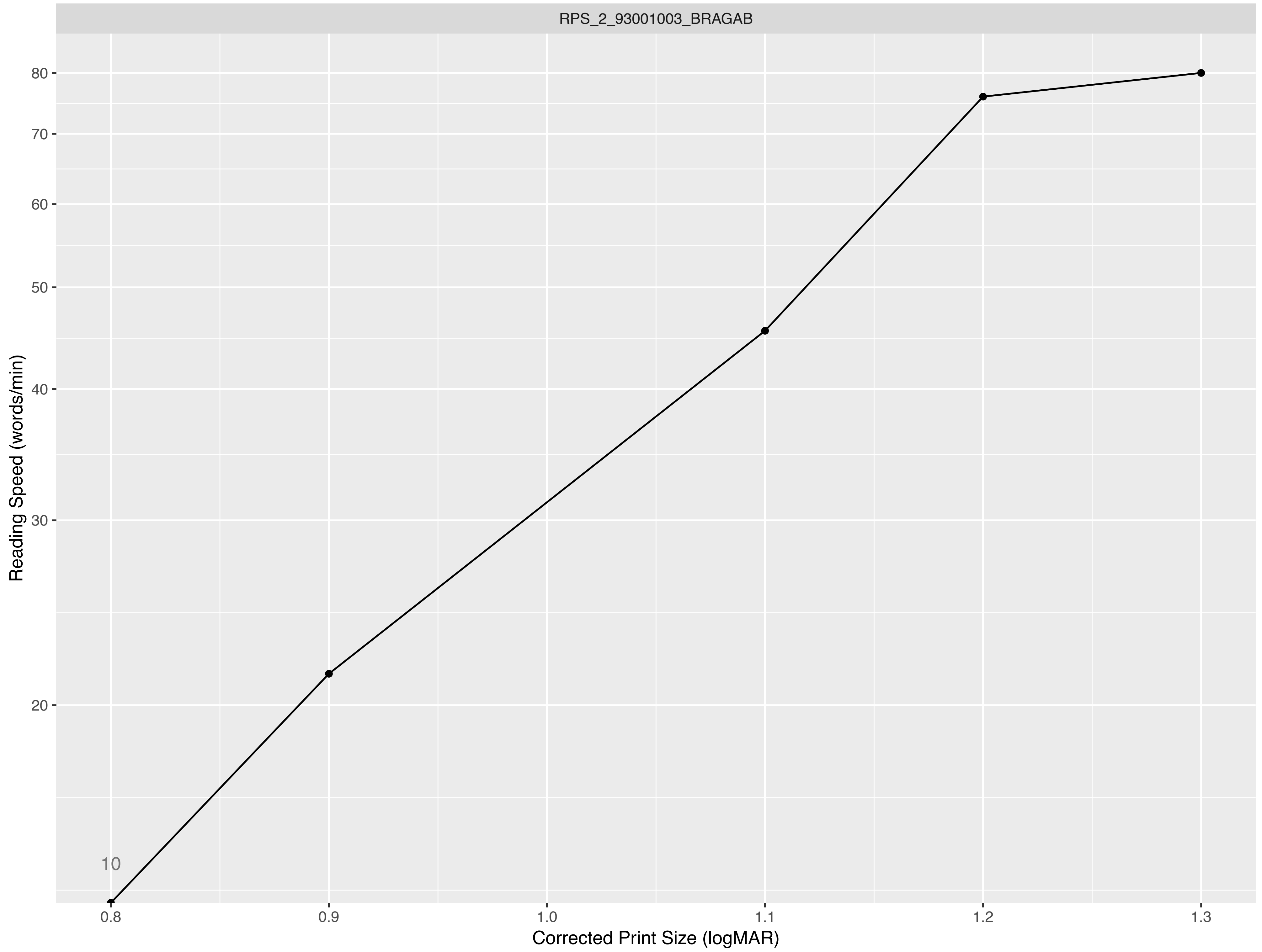

RPS_2_93001003_BRAGAB
80
70
60
50
40
30
20
10
Reading Speed (words/min)
0.8
0.9
1.0
1.1
1.2
1.3
Corrected Print Size (logMAR)

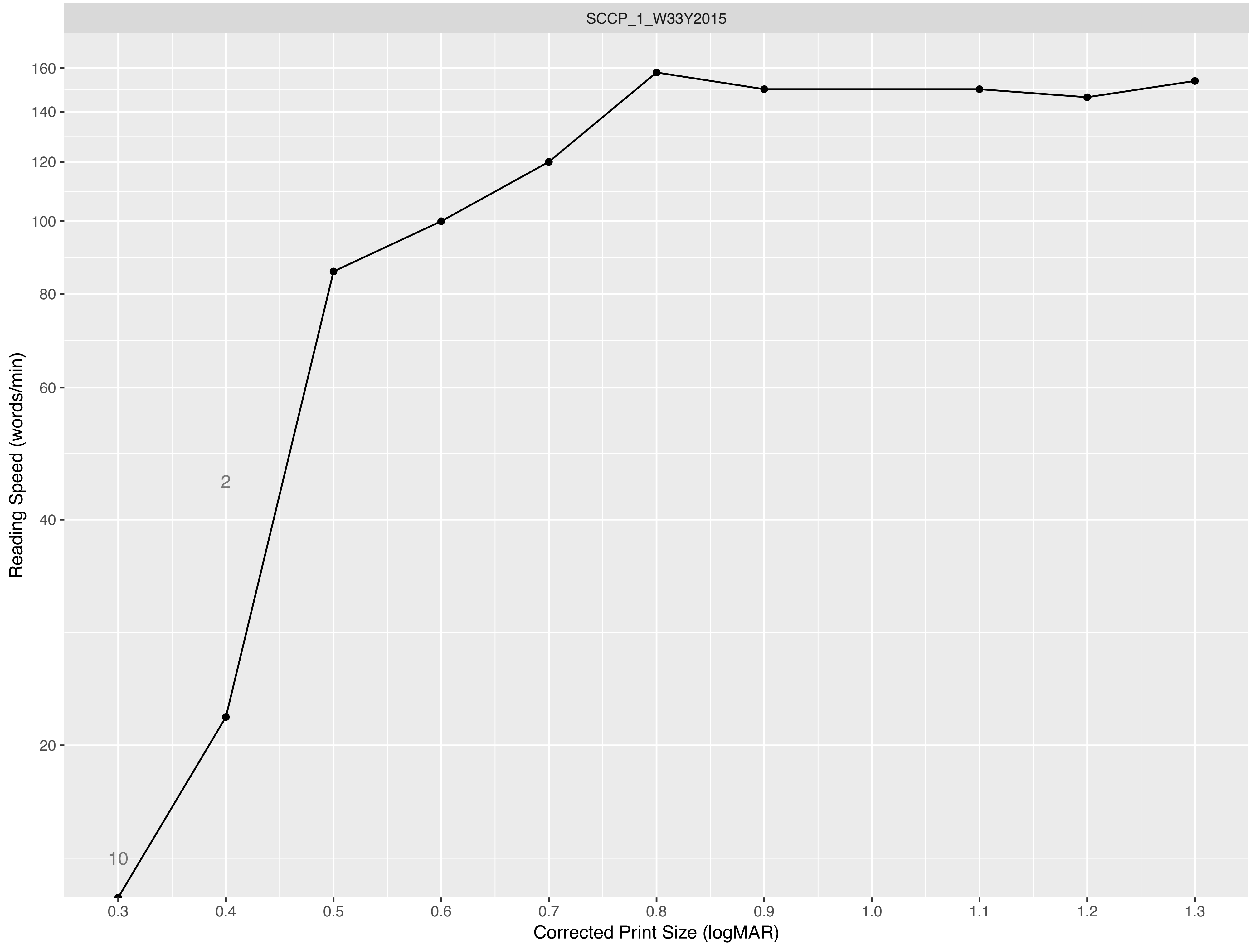
SCCP_1_W33Y2015
Reading Speed (words/min)
160
140
120
100
80
60
40
20
2
10
0.3
0.4
0.5
0.6
0.7
0.8
0.9
1.0
1.1
1.2
1.3
Corrected Print Size (logMAR)

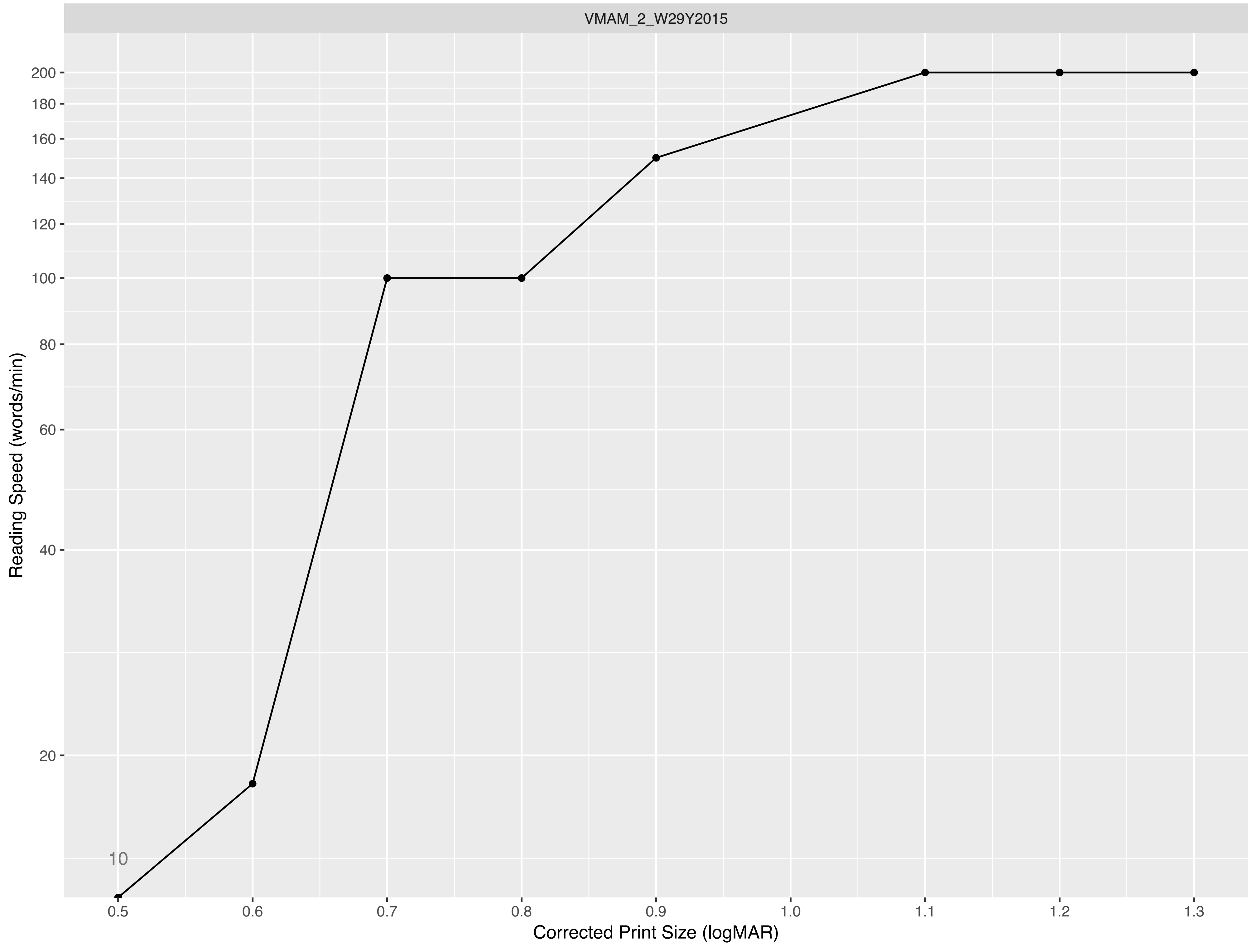

VMAM_2_W29Y2015
Reading Speed (words/min)
200
180
160
140
120
100
80
60
40
20
10
0.5
0.6
0.7
0.8
0.9
1.0
1.1
1.2
1.3
Corrected Print Size (logMAR)
