# Supplementary material for: Scoring reading parameters: An inter-rater reliability study using the MNREAD chart
Source: PLoS One. 2019 Jun 7;14(6):e0216775. doi: 10.1371/journal.pone.0216775 (PMC6555504; doi:10.1371/journal.pone.0216775)
Supplement: S2 Appendix — (PDF) [file pone.0216775.s002.pdf]

Visual estimation of the

Maximum Reading Speed (MRS) and Critical Print Size (CPS)

from individual MNREAD curves

Instructions to raters

The MNREAD acuity charts are continuous-text reading-acuity charts suitable for measuring the reading performance of normal and low-vision individuals. This particular test allows to measure how reading performance changes when print size decreases.

Values of reading speed collected with the MNREAD can be plotted as a function of print size to display an MNREAD curve.

From this curve, two estimates of reading performance can be extracted:

- Critical Print Size (CPS) - The smallest print that can be read with maximum speed.
- Maximum Reading Speed (MRS) - The fastest reading speed that can be achieved when reading is not limited by print size.

The present document provides guidelines to estimate the Maximum Reading Speed and Critical Print Size from MNREAD curves.

Instructions comes in two steps:

1. The standard definition of each reading parameter, as provided with the original MNREAD chart instructions (for more details, visit http://gellab.dl.umn.edu/mnread-speed) and illustrated with a standard MNREAD curve example.
2. Some extra tips and tricks to help users estimate the reading parameters for ambiguous curves (e.g. sparse data, non-standard curve shape, etc.)

1. PARAMETERS DEFINITION 2

EXAMPLE 1: STANDARD CURVE 2

2. EXTRA TIPS TO HELP DEAL WITH AMBIGUOUS CASES 3

EXAMPLE 2: BELL-SHAPED CURVE 3

EXAMPLE 3: THE CURVE DOES NOT REACH A PLATEAU 4

EXAMPLE 4: GRADUAL INCREASE IN READING SPEED 5

EXAMPLE 5: STAIRCASE-SHAPED CURVE 6

1. PARAMETERS DEFINITION

EXAMPLE 1: STANDARD CURVE

The Critical Print Size (CPS) is the smallest print size at which patients can read with their maximum reading speed. This is an important measure that indicates the minimum magnification required for effortless reading. Typically, reading time remains fairly constant for large print sizes. But as the acuity limit is approached there comes a print size where reading starts to slow down. This is the critical print size.

The reading speed with print larger than the critical print size is the Maximum Reading Speed (MRS). This is the reading speed that can be achieved by the patient when print size is not a limiting factor.

In a typical MNREAD curve (Figure 1), the MRS corresponds to the plateau of the curve, *i.e.* a region with high and sustained reading speed (represented here by the red data points). The characteristics of the plateau will help determine both MRS (in orange) and CPS (in blue). In this example, the plateau ranges from 1.4 logMAR to 1.2 logMAR and lies at 80 words/minute (wpm). Therefore, the **MRS is 80 wpm, the average reading speed on the plateau** and the **CPS is 1.2 logMAR, the smallest print on the plateau**.

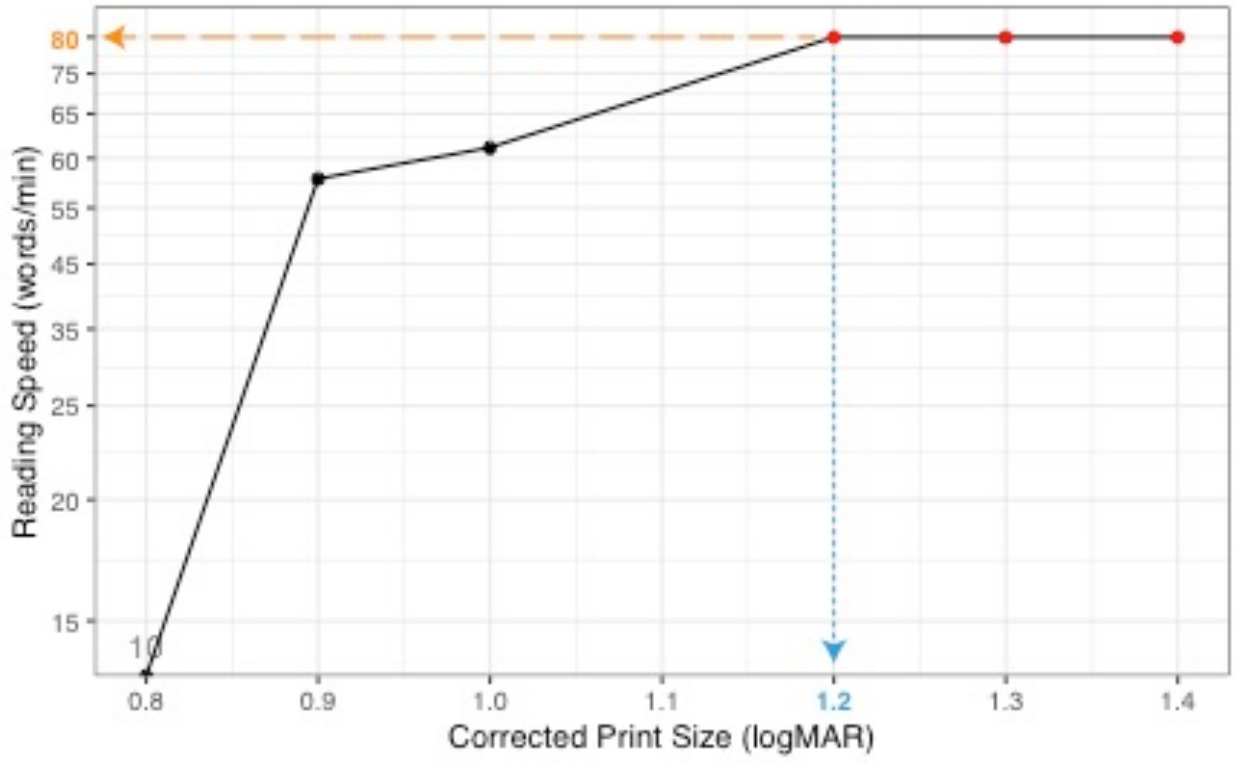

Figure 1: Typical MNREAD curve. In this example, MRS is 80 words/minute and CPS is 1.2 logMAR. Red data points correspond to the plateau.

2. EXTRA TIPS TO HELP DEAL WITH AMBIGUOUS CASES

Example 1 is a typical MRNEAD curve for which parameters' estimation is quite straightforward. However, individuals with low vision will usually have poor reading abilities, resulting in ambiguous curves. In such cases, MRS and CPS might be less obvious. We have included here 4 examples of ambiguous curves.

EXAMPLE 2: BELL-SHAPED CURVE

This type of curve usually reflects the presence of a ring scotoma in the center of the visual field. Here the scotoma occludes large print text, leading to poor reading speed from 1.6 to 1.4 logMAR. However, once print size decreases, text fits within the ring scotoma, enabling smaller print to be read faster (from 1.2 to 0.9 logMAR). This portion of the curve can be considered as the plateau with an MRS of about **130 words/minute**. Once print becomes smaller than **0.9 logMAR** though, reading speed starts dropping: this is the CPS.

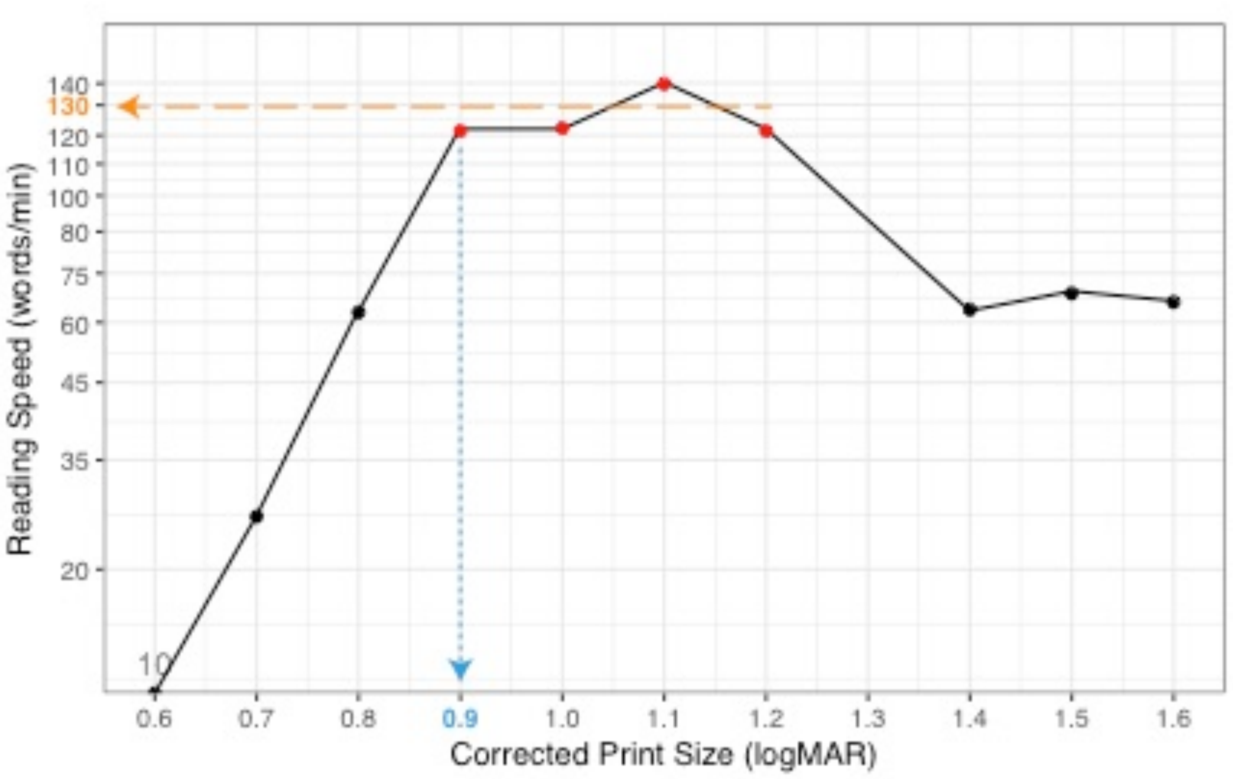

Figure 2: Bell-shaped MNREAD curve. In this example, MRS is 130 words/minute and CPS is 0.9 logMAR. Red data points correspond to the plateau.

EXAMPLE 3: THE CURVE DOES NOT REACH A PLATEAU

Figure 2 shows a curve with a gradual decrease in reading speed and no clear plateau. In this case, the larger print presented (1.3 logMAR) is probably not big enough to reach a reading speed plateau. The fastest reading speed achieved can be considered as the MRS (**80 words/minute** here) with a corresponding CPS of **1.3 logMAR**. In order to obtain more accurate measurements of MRS and CPS, the MRNEAD test should be run again at a closer viewing distance, which will increase the print size range on the larger end of the chart. For example, bringing the chart at 20 cm (instead of the recommended 40 cm) will move the range of tested print sizes from 1.3 / -0.5 logMAR to 1.6 / -0.1 logMAR. Increasing print size on the larger end by 0.3 logMAR.

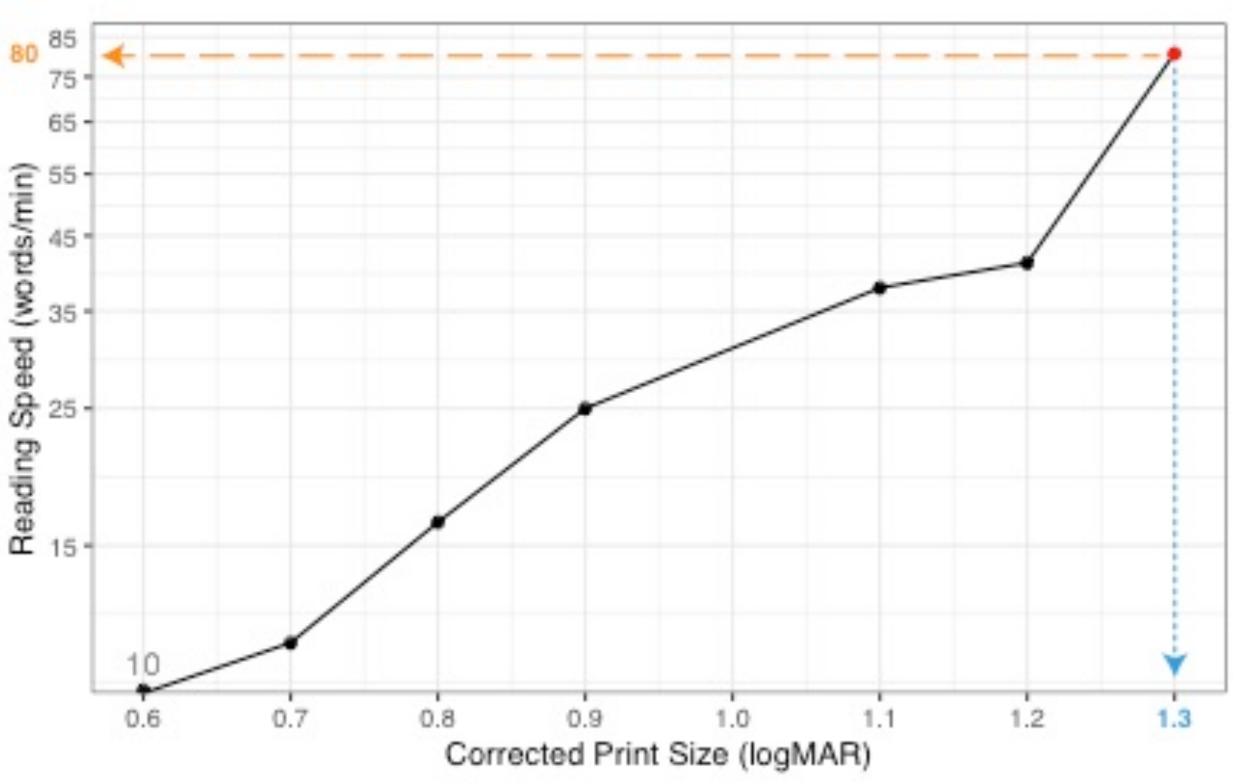

Figure 3: MNREAD curve that does not reach a plateau. In this example, MRS is 80 words/minute and CPS is 1.3 logMAR. Red data points correspond to the plateau.

EXAMPLE 4: GRADUAL INCREASE IN READING SPEED

The curve in Figure 4 shows a gradual increase in reading speed as print size increases, up to a critical print size, where speed starts dropping. In such cases, CPS is quite easy to estimate. It corresponds to this dropping point (here at **0.6 logMAR**). However, since there is not strict plateau MRS might be little harder to derive. To help estimate MRS, the range of reading speed measured (on the vertical axis) becomes very informative because it will help decide which data points to include in the plateau. Even though they look apart, data points from 1.1 to 0.6 logMAR (in red) range from 27 to 37 words / minute, which is actually quite a narrow range. Here CPS is set to **0.6 logMAR** and MRS is set to **32 words/minute**.

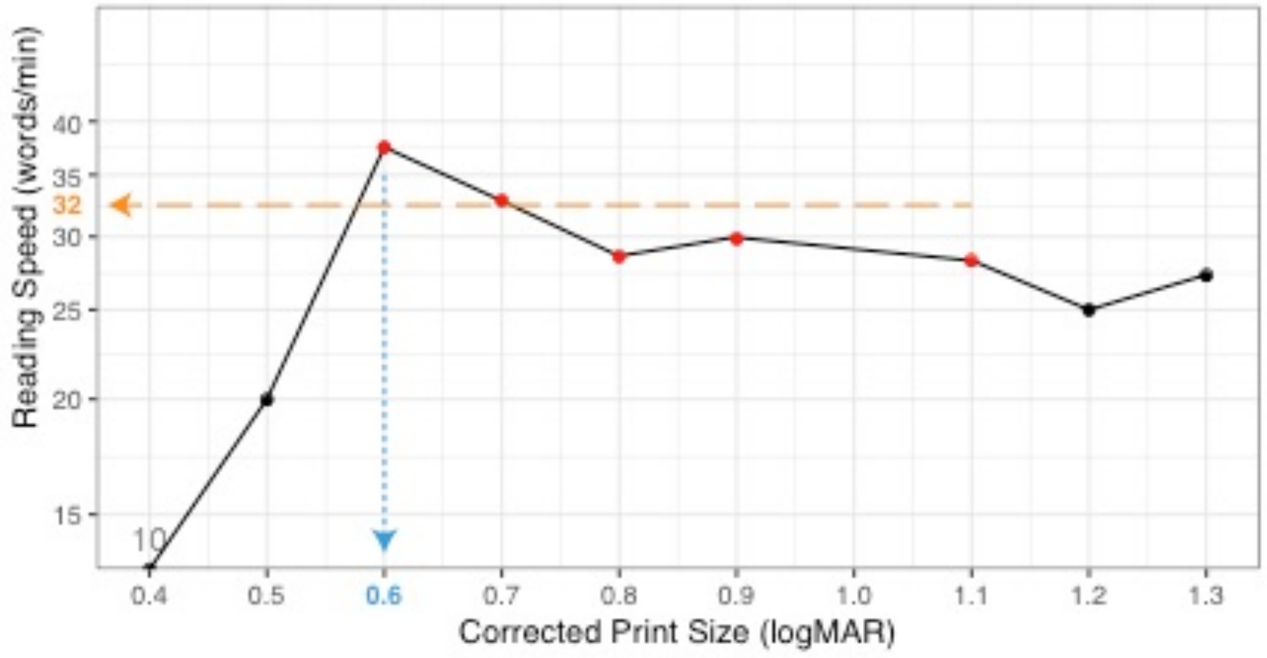

Figure 4: MNREAD curve with a gradual increase in reading speed. In this example, MRS is 32 words/minute and CPS is 0.6 logMAR. Red data points correspond to the plateau.

EXAMPLE 5: STAIRCASE-SHAPED CURVE

Figure 5 shows a very common MNREAD curve with the presence of two plateaus: one from 1.3 to 1.2 logMAR, and a second one from 1.1 to 0.7 logMAR. Although this second plateau encompass most of the data points with an average speed of 80 words/ minute, it does not represent the Maximum Reading Speed, which achieves **120 words/minute** until a Critical Print Size of **1.2 logMAR** is reached.

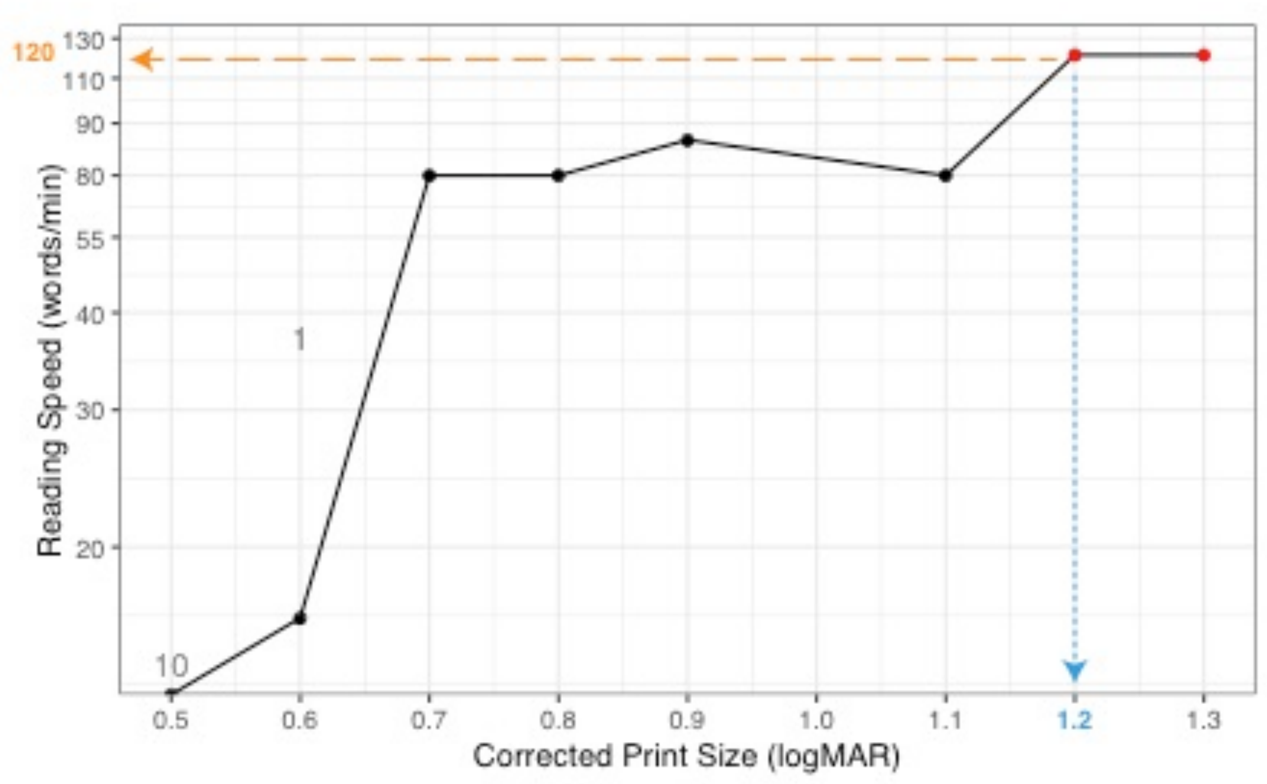

Figure 5: Staircase-shaped MNREAD curve. In this example, MRS is 120 words/minute and CPS is 1.2 logMAR. Red data points correspond to the plateau.
