# Supplementary figures and images for: Scoring reading parameters: An inter-rater reliability study using the MNREAD chart
Source: PLoS One. 2019 Jun 7;14(6):e0216775. doi: 10.1371/journal.pone.0216775 (PMC6555504; doi:10.1371/journal.pone.0216775)

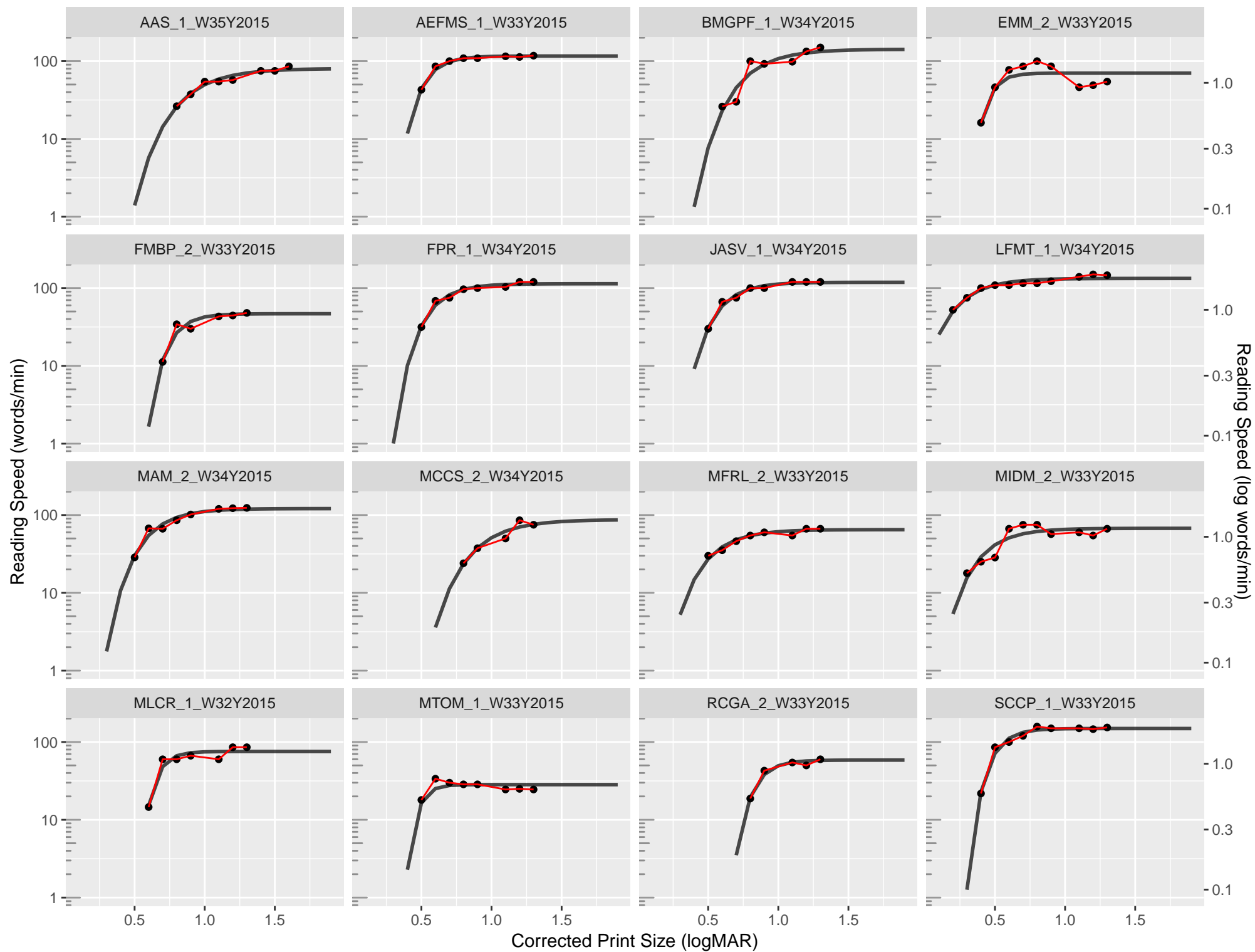

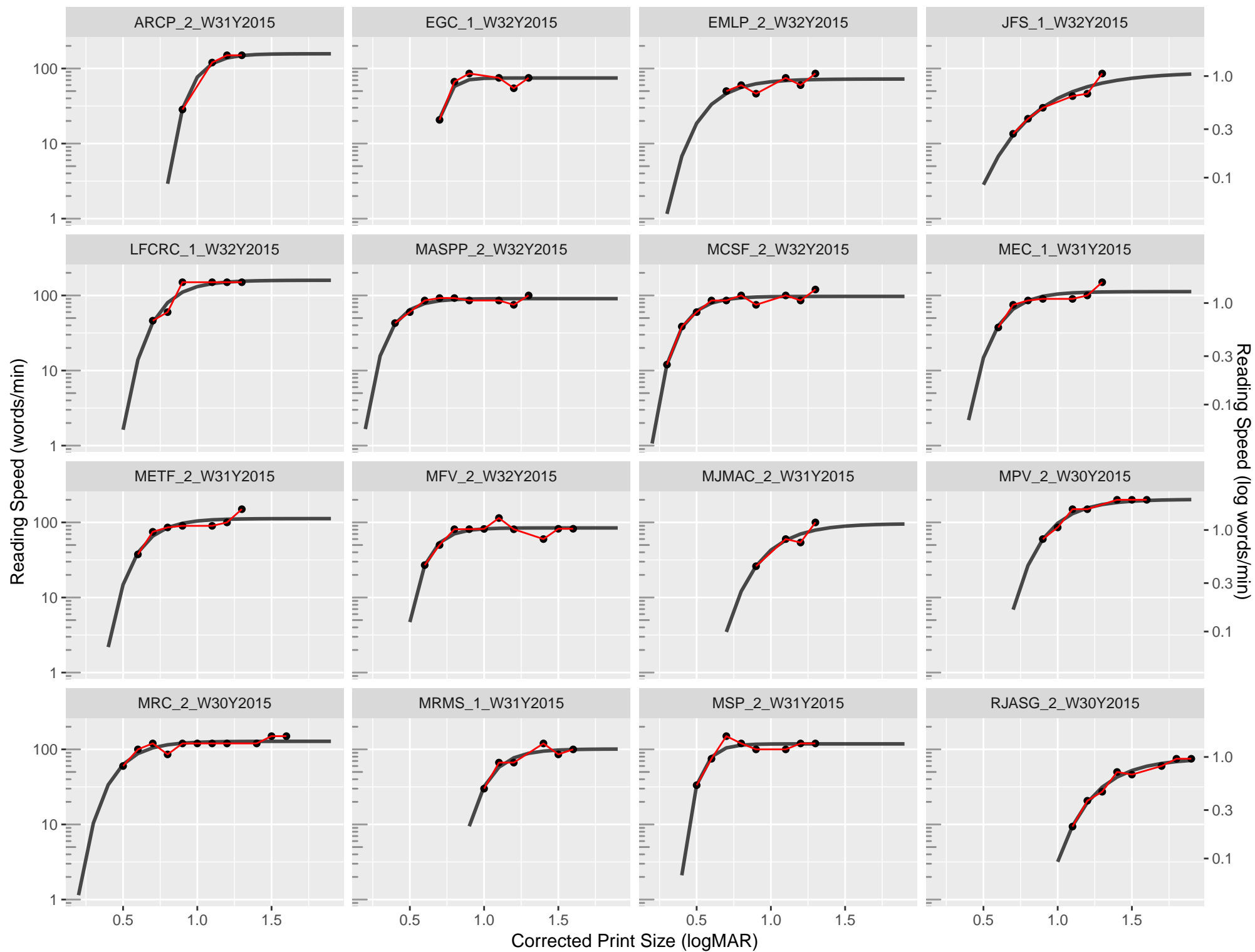

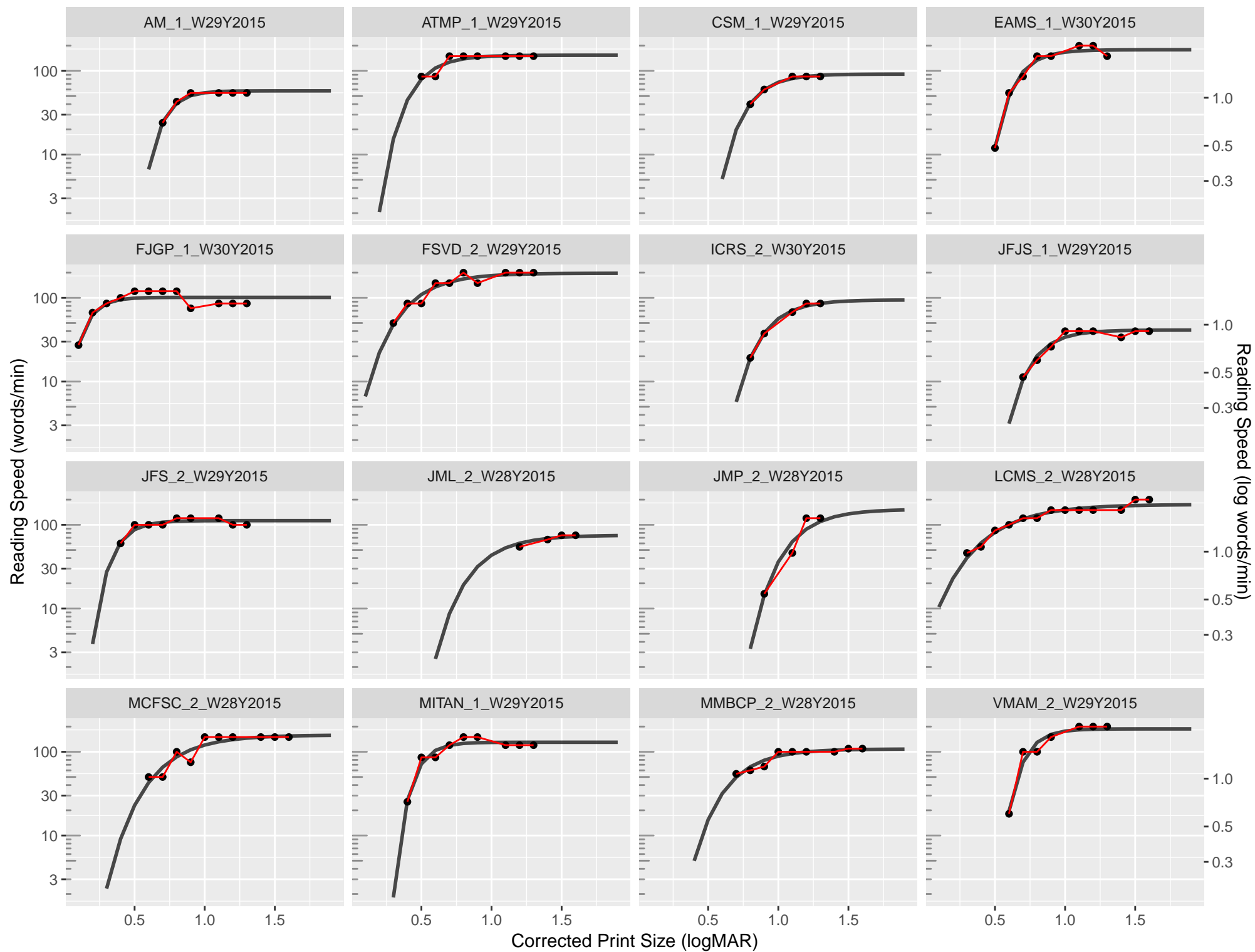

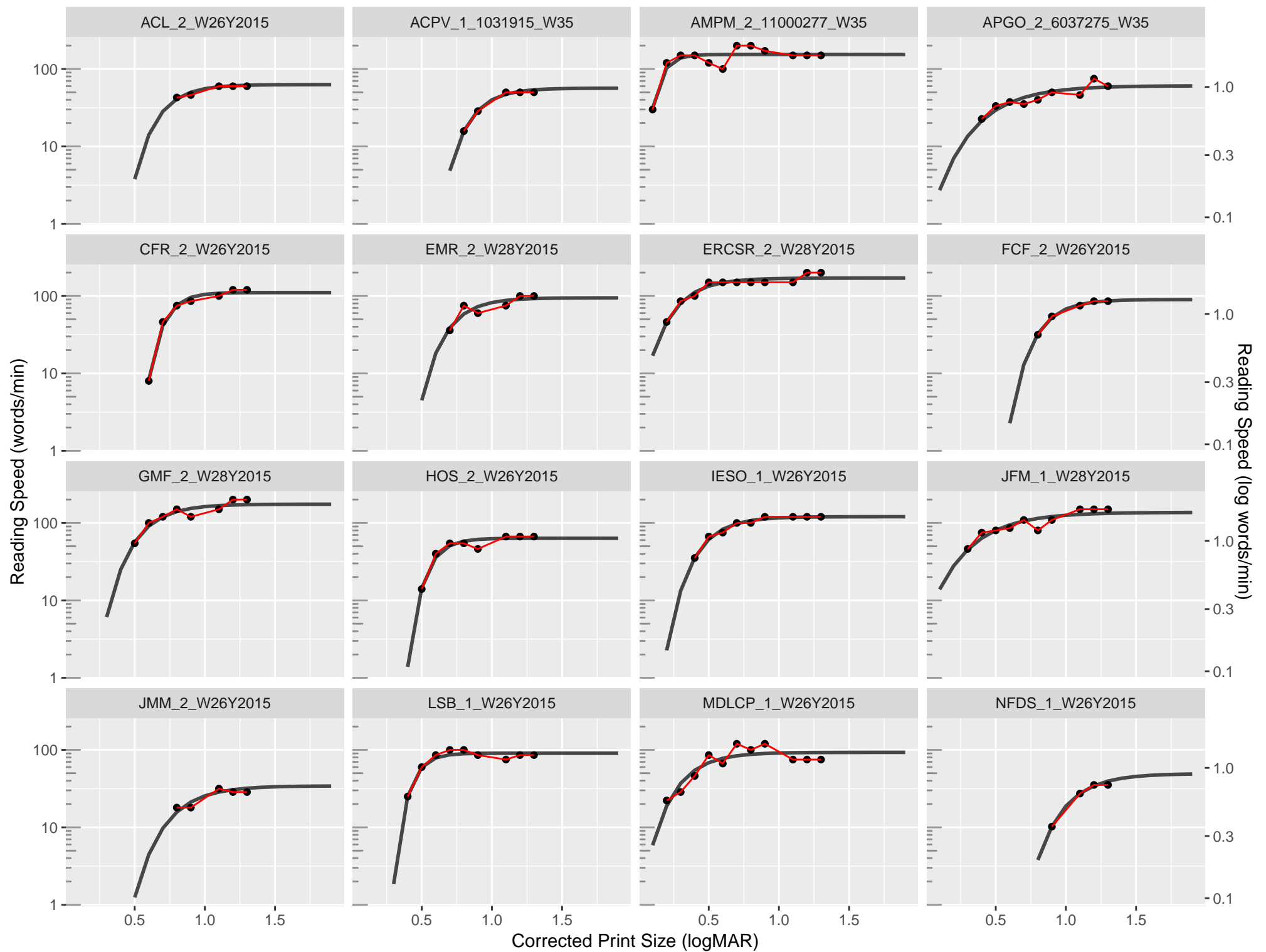

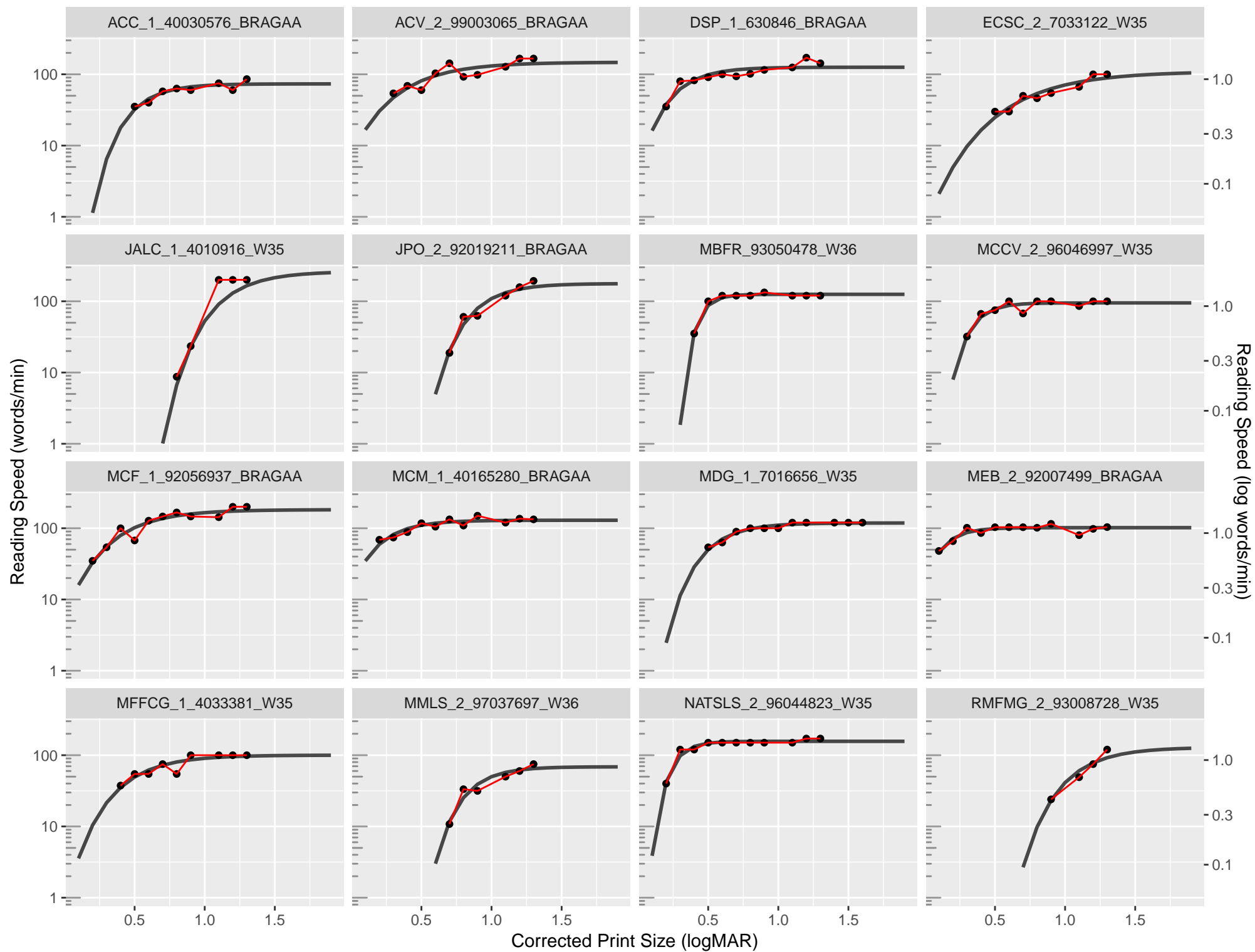

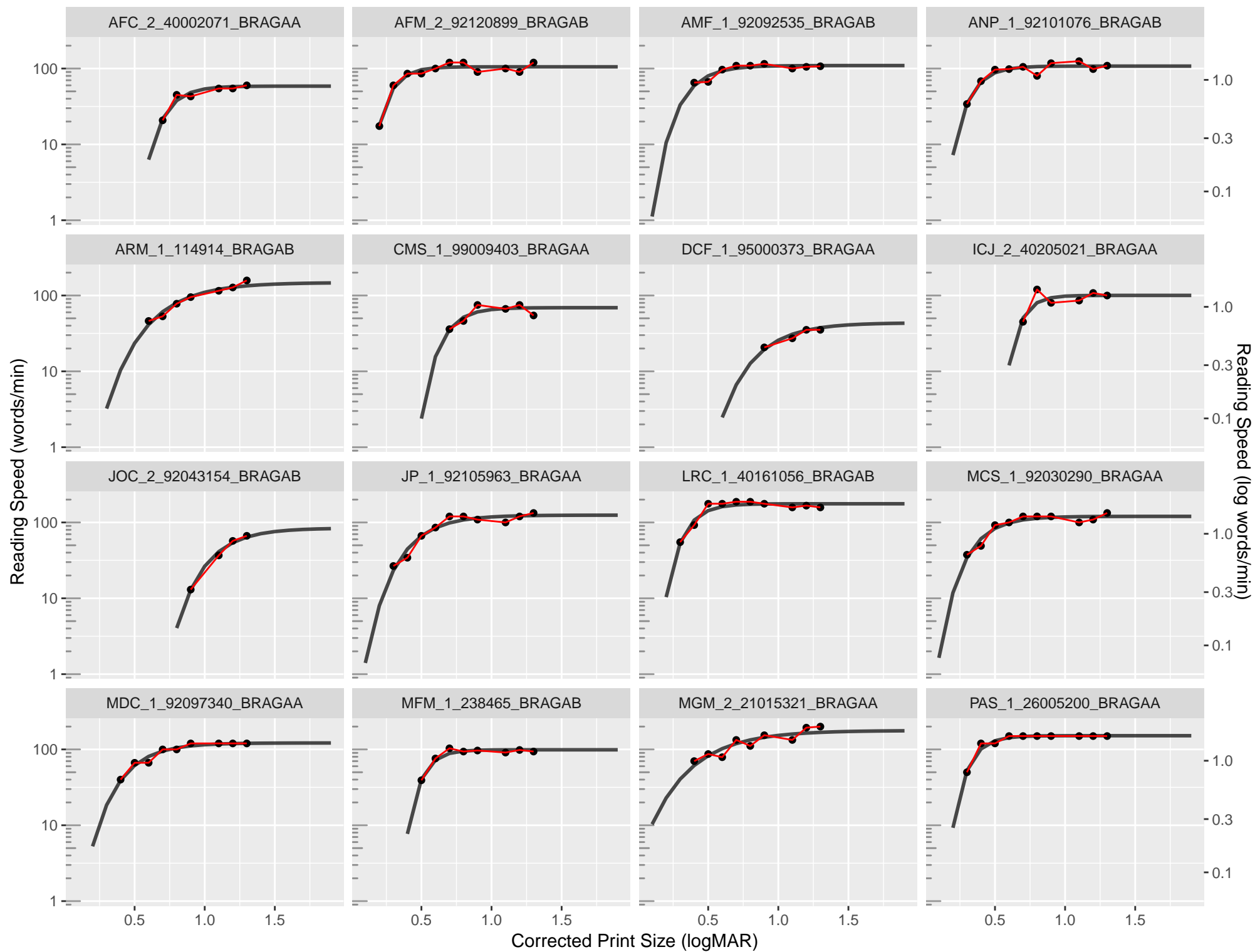

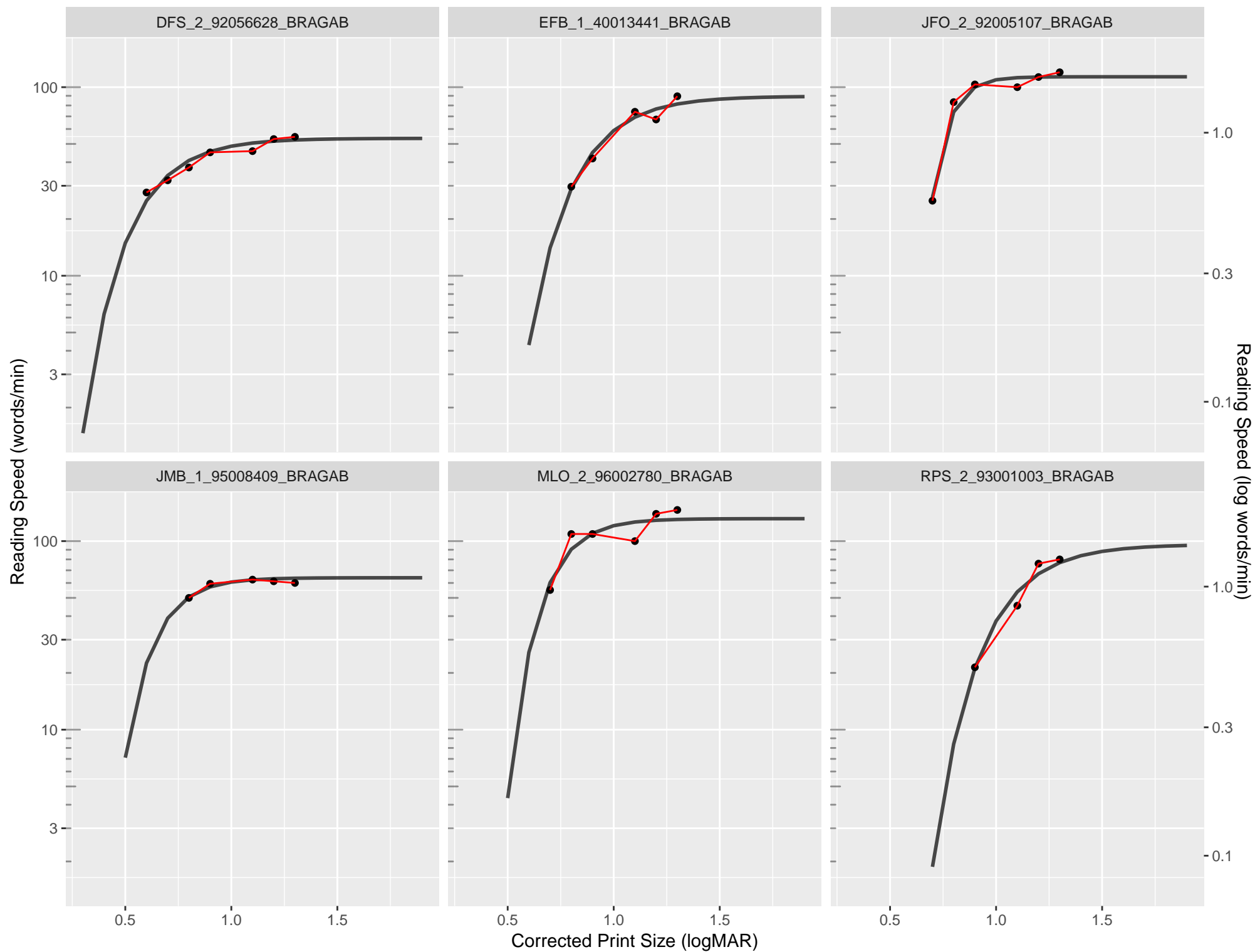

Supplement: S3 Appendix — (PDF) [file pone.0216775.s003.pdf]
